# Delineation guidelines for the lymphatic target volumes in ‘prone crawl’ radiotherapy treatment position for breast cancer patients

**DOI:** 10.1038/s41598-021-01841-y

**Published:** 2021-11-18

**Authors:** Michael E. J. Stouthandel, Françoise Kayser, Vincent Vakaet, Ralph Khoury, Pieter Deseyne, Chris Monten, Max Schoepen, Vincent Remouchamps, Alex De Caluwé, Guillaume Janoray, Wilfried De Neve, Stephane Mazy, Liv Veldeman, Tom Van Hoof

**Affiliations:** 1 Department of Human Structure and Repair, Ghent University, C. Heymanslaan 10, Radiotherapy park, entrance 98, 9000 Ghent, Belgium; 2 Department of Radiology, Université Catholique de Louvain, CHU UCL Namur, Yvoir, Belgium; 3 Department of Radiation Oncology, Ghent University Hospital, C. Heymanslaan 10, Radiotherapy park, entrance 98, 9000 Ghent, Belgium; 4 Department of Radiotherapy, CHU UCL Namur, site Ste Elisabeth, Place Louise Godin 15, 5000 Namur, Belgium; 5 Department of Radiation Oncology, Institut Jules Bordet – Université Libre de Bruxelles (ULB), Brussels, Belgium; 6 Department of Radiology, CHU-UCL Namur, site Ste Elisabeth, Place Louise Godin 15, 5000 Namur, Belgium

**Keywords:** Anatomy, Breast cancer, Radiotherapy

## Abstract

Our recently developed prone crawl position (PCP) for radiotherapy of breast cancer patients with lymphatic involvement showed promising preliminary data and it is being optimized for clinical use. An important aspect in this process is making new, position specific delineation guidelines to ensure delineation (for treatment planning) is uniform across different centers. The existing ESTRO and PROCAB guidelines for supine position (SP) were adapted for PCP. Nine volunteers were MRI scanned in both SP and PCP. Lymph node regions were delineated in SP using the existing ESTRO and PROCAB guidelines and were then translated to PCP, based on the observed changes in reference structure position. Nine PCP patient CT scans were used to verify if the new reference structures were consistently identified and easily applicable on different patient CT scans. Based on these data, a team of specialists in anatomy, CT- and MRI radiology and radiation oncology postulated the final guidelines. By taking the ESTRO and PROCAB guidelines for SP into account and by using a relatively big number of datasets, these new PCP specific guidelines incorporate anatomical variability between patients. The guidelines are easily and consistently applicable, even for people with limited previous experience with delineations in PCP.

## Introduction

Radiotherapy of the regional lymph nodes, i.e. nodal irradiation, is an effective treatment option for patients with early stage breast cancer and lymphatic involvement. It was shown to improve overall survival and it lowers the risk of local recurrence^1–4^. However, radiotherapy can cause serious side effects like ischemic heart disease^5^ and secondary lung cancer^6^. To prevent these side effects, it is important to reduce radiation exposure of the organs at risk, like the heart and the lungs^7,8^. By placing the patient in prone position and allowing the breast to hang down, the breast is further removed from the chest cavity, providing an opportunity to spare the heart and lungs^9^. We recently introduced a new patient position with complementary positioning device to allow nodal radiotherapy in prone position^10^. The arm on the treated side is placed alongside the body, providing good access to the regional lymph nodes. Treating the regional lymph nodes with good coverage is challenging in prone ‘dive’ position set ups, because both arms are elevated above the head^9–11^. In our new position, only the contralateral arm is elevated above the head, making it look like the patient is in a phase of the crawl swimming movement. Hence, the new position is referred to as the prone crawl position (PCP)^10^. Preliminary research confirmed the potential of the PCP for nodal radiotherapy, showing reduced radiation dose to the lungs, the contralateral breast, the thyroid and the esophagus, compared to supine position with arms in elevation (SP). Good nodal coverage was also achieved, making PCP a promising candidate for widespread clinical use^12^.

SP is most commonly used for breast cancer radiotherapy^9^. Different consensus guidelines have been published for delineation of the nodal areas in SP^13–18^. The RTOG (radiation therapy oncology group) and ESTRO (European society for therapeutic radiology and oncology) guidelines are most frequently used^19^. To our knowledge, no official delineation guidelines for prone breast radiotherapy are yet available. The existing guidelines cannot simply be applied in PCP, because the reference structures will have a different spatial orientation, due to the change in patient position. A similar uniform consensus guideline is needed for PCP to facilitate clinical application in different centers. The European ESTRO^14^ and PROCAB (project on cancer of the breast)^13^ guidelines differ only slightly from one another and have been shown to provide better nodal coverage and include less healthy tissue than the RTOG guidelines^19,20^. Therefore, these guidelines were chosen as a starting point for PCP guideline development.

The aim of this study was to create new PCP specific delineation guidelines, to provide a uniform standard for centers that wish to implement the technique in the future.

## Materials and methods

### Delineation guideline development

**Figure 1:**
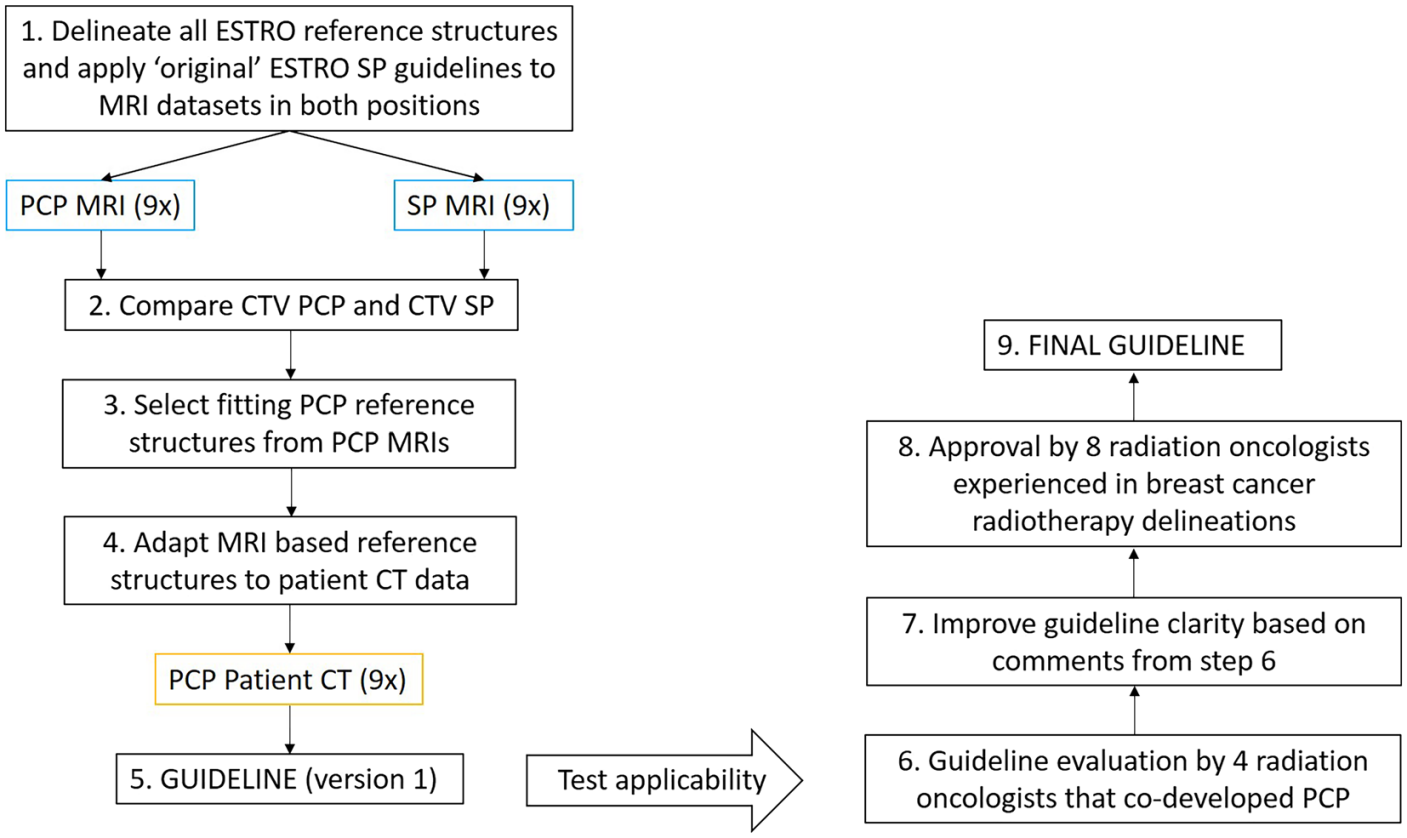
Schematic representation of the guideline development process. *SP* supine position with arms in elevation, *MRI* magnetic resonance imaging dataset, *CTV* clinical target volume, *PCP* prone crawl position, *CT* computed tomography dataset. 9 volunteers were MRI scanned in PCP, the same 9 volunteers were also MRI scanned in SP to obtain complementary datasets. 9 (different) patients were scanned to obtain the PCP patient CT data.

The existing ESTRO^14^ and PROCAB^13^ SP guidelines were used as a basis for PCP guideline development, taking into account the anatomical differences between both positions. The ESTRO and PROCAB target volumes were respected where possible, because they were previously endorsed by the European radiotherapy society^13,14^. 18 MRI and 9 CT datasets were evaluated to include interpatient anatomical variability. The protocol consists of 9 steps that are depicted in Fig. 1 and further clarified below. All methods were carried out in accordance with the declaration of Helsinki and according to institutional guidelines and regulations.

To facilitate initial guideline development, 9 volunteers were MRI scanned in PCP (PCP MRI) and SP (SP MRI), providing 9 complementary datasets for intrapersonal comparison between SP and PCP. The MRI scans in SP and PCP were used to indicate reference structures and delineate nodal targets according to the ESTRO and PROCAB SP guidelines (step 1). From this comparison between the two positions, it was recorded which ESTRO and PROCAB clinical target volume (CTV) borders needed to be adapted for PCP, due to the position shift of the reference structures. During the process of choosing new reference structures, the resulting PCP CTVs were routinely compared to the SP CTVs, to ensure that similar structures (mainly the veins) were contained within the CTVs (step 2 and 3). To confirm that new MRI based reference structures were also applicable in a clinical setting, they were subsequently applied on 9 patient CT scans (PCP patient CT) with lower contrast detail. MRI reference structures that were not consistently easily identified, or not consistently applicable on all 9 patient CT scans were replaced with CT appropriate reference structures (step 4). This process provided a first set of guidelines that were tested for applicability by 4 radiation oncologists involved in developing radiotherapy in PCP (VV, PD, CM, LV) (step 5 and 6). The radiation oncologists were instructed to strictly apply the guidelines and report any unclarities encountered during guideline application. By taking the reported unclarities into account, the guidelines were further refined (step 7). After review and approval by 8 radiation oncologists experienced in breast cancer radiotherapy delineations (VV, PD, CM, VR, ADC, GJ, LV, WDN) the final set of guidelines, presented in the results section, was obtained (step 8 and 9). Mimics Innovation Suite software, version 20.0 (Materialise, Leuven, Belgium, www.materialise.com/en/medical/mimics-innovation-suite) was used for delineations and 3D comparison between datasets.

### CT data acquisition

PCP patient CT data was available from patients treated for breast cancer with lymphatic involvement at the Ghent University Hospital radiotherapy department. Patients signed informed consent for the use of their medical imaging data and the study was approved by the institutional ethics committee (UZ Gent, NCT03280719). Patients were scanned using a Toshiba Aquilion CT-simulator (Toshiba, Tokyo, Japan). A slice thickness of 5 mm was used, since treatment planning is routinely performed on datasets with 5 mm slice thickness at our center. A total of 9 PCP patient CT scans was used to set up the guidelines.

### MRI data acquisition

MRI datasets were acquired from volunteers, after they signed informed consent. This study was approved by the medical ethics committee of CHU UCL Namur, site Godinne (CHU UCL Namur, site Godinne, B039201941613). Volunteers were scanned in both PCP and SP to obtain corresponding MRI datasets to compare target volumes and reference structure positions. Volunteers were scanned on a 3T Verio MRI scanner (Siemens, Erlangen, Germany), using a T1 3D spin echo sequence. A slice thickness of 1.7 mm was used to include as much anatomical detail as possible. A total of 18 MRI scans was used to set up the guidelines (9 PCP scans and 9 SP scans).

## Results

**Figure 2:**
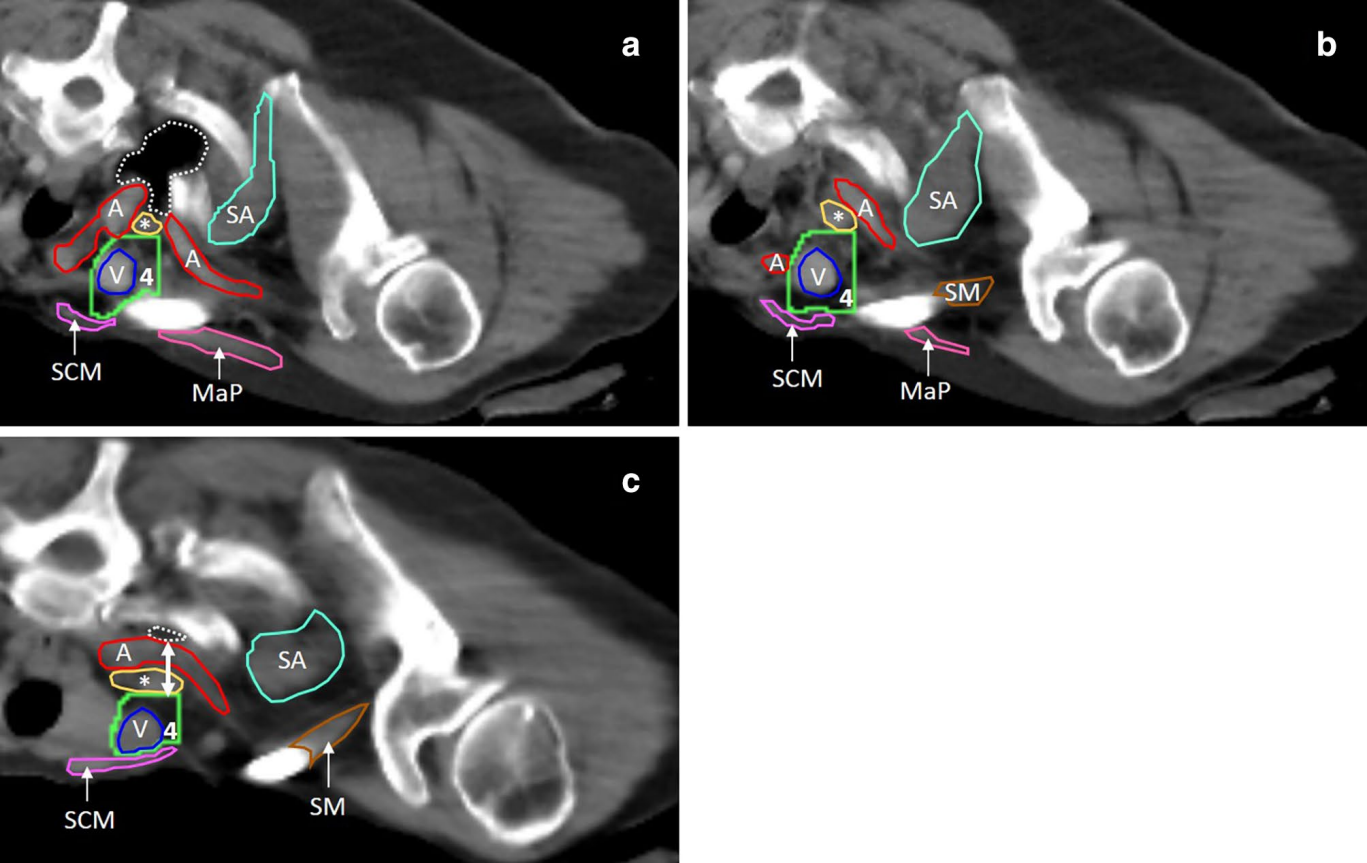
Adaptations to level IV borders indicated on patient CT scans taken in PCP. (**a**) Shows the first slice where the pleura is visible (dotted white line). (**b**), (taken from the same patient) shows the top of the subclavian artery after moving 5 mm (1 slice) in cranial direction (cranial border). (**a**) and (**b**) illustrate the new cranial border of level IV. Panel c shows the first cranial slice containing pleura (dotted white lines) on a different patient. (**c**) Illustrates the need for an adapted dorsal border in level IV. The ventral edge of the anterior scalene muscle is still located 20 mm more ventral from the pleura in this patient (double arrow), while the ventral edge of the anterior scalene muscle falls within the 5 mm dorsal limit from the dorsal edge of the vein. Turquoise (SA) = serratus anterior muscle, red (A) = common carotid artery, or subclavian artery, orange (*) = anterior scalene muscle, bright green (4) = level IV, blue (V) = internal jugular vein, purple (SCM) = sternocleidomastoid muscle, brown (SM) = subclavius muscle, pink (MaP) = major pectoral muscle.

**Figure 3:**
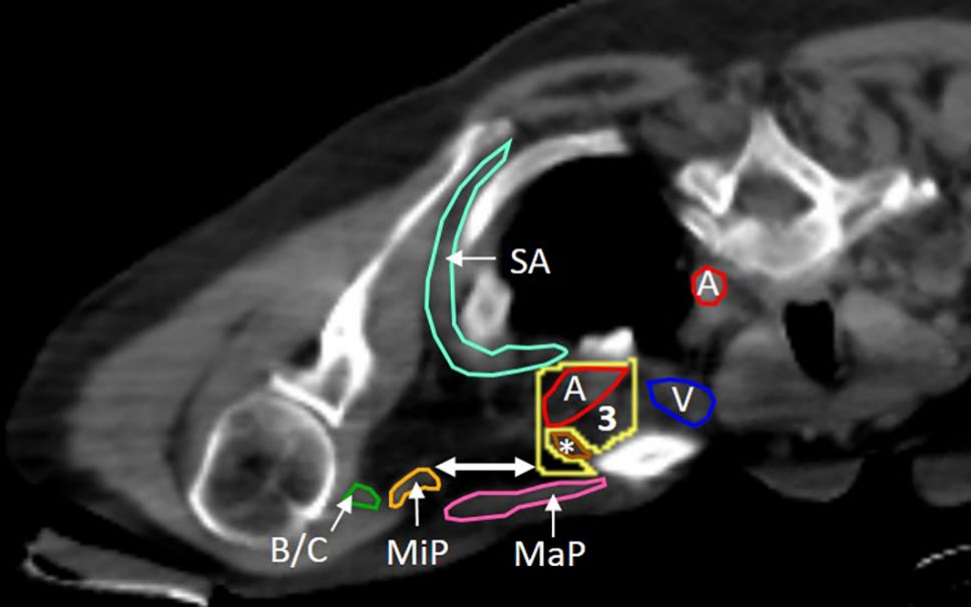
Adaptations to level III borders indicated on patient CT scan taken in PCP. The figure shows the first cranial slice where the axillary/subclavian artery first crosses both the lateral border of the first rib and the clavicle. In the most cranial slices of level III, the minor pectoral muscle can still be located (very laterally) close to its insertion on the coracoid process of the scapula. To spare the (up to 30 mm) margin indicated by the double arrow, the lateral border is taken at the lateral edge of the axillary artery until the artery crosses the medial edge of the minor pectoral muscle. Also note that the CTV excludes the subclavius muscle. Turquoise (SA) = serratus anterior muscle, red (A) = common carotid artery, or axillary/subclavian artery, yellow (3) = level III, blue (V) = subclavian vein, brown (*) = subclavius muscle, orange (MiP) = minor pectoral muscle, dark green (B/C) = biceps/coracobrachial muscle bundle, pink (MaP) = major pectoral muscle.

**Figure 4:**
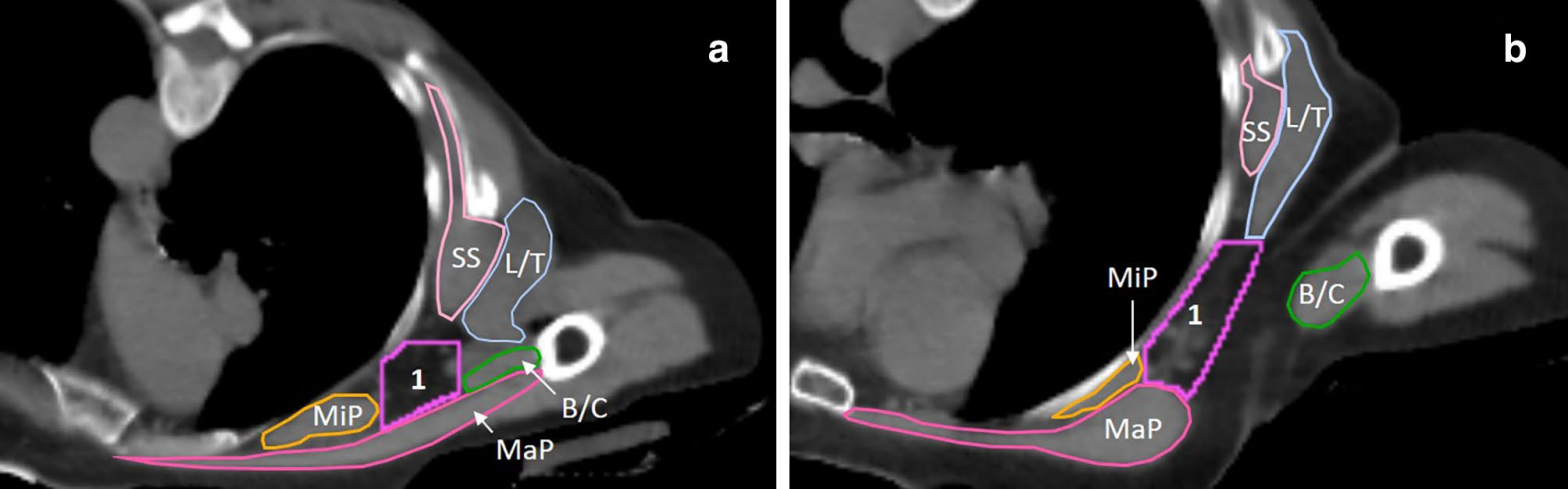
Adaptations to level I borders indicated on patient CT scan taken in PCP. (**a**) Shows a representation of the cranial slices, where the lateral border is defined by the medial edge of the biceps/coracobrachial muscle bundle, because the major pectoral muscle is still connected to the arm. Note that the dorsal border stops at the ventral edge of the latissimus dorsi/teres major muscle bundle here, because this reaches more ventrally than the ventral edge of the subscapular muscle at this level. (**b**) Shows a representation of the more caudal slices in level I  (of the same patient) where the major pectoral muscle is no longer connected to the arm and the lateral border is formed by connecting the lateral edge of the major  pectoral muscle to the ventral edge of the latissimus dorsi/teres major  muscle bundle. Light pink (SS) = subscapular muscle, light blue (L/T) = latissimus dorsi/teres major muscle bundle, purple (1) = level I, green (B/C) = biceps/coracobrachial muscle bundle, orange (MiP) = minor pectoral muscle, dark pink (MaP) = major pectoral muscle.

The final PCP guidelines are presented in Table 1 and described below. Table 1 also indicates which borders were adapted from ESTRO^14^ and PROCAB^13^. Figure 2, 3 and 4 clarify these adaptations. The reasoning behind the CTV border changes is explained in the discussion section.

**Table 1:** Prone crawl position specific delineation guidelines for the CTV of the lymph node regions.

Border per region	Level IV	Level III	Level II	Level I	Inter-pectoral nodes	Internal mammary nodes
Cranial	*5 mm cranial from the first cranial slice containing pleura*	First cranial slice the subclavian artery crosses the lateral border of both the first rib and the clavicle	First cranial slice where the axillary artery crosses the medial edge of the pec minor	*5 mm cranial from 1st cranial slice where the axillary vein crosses the lateral edge of the pec minor*	Same as level II	5 mm caudal to caudal border of level IV
Caudal	5 mm caudal from the first caudal slice where the subclavian vein and internal jugular vein fuse	5 mm caudal from the first cranial slice where the subclavian vein crosses the medial edge of the pec minor	Last slice with fatty tissue between ribs and pec minor (attachment of pec minor to ribs)	Point where the 4th rib attaches to the sternum (stop at 1st caudal slice without 4th rib next to sternum)	Same as level II	1st caudal slice without 4th rib next to sternum (same as level I)
Medial	Medial edge of internal jugular vein, or medial edge of subclavian vein	Lateral border of the clavicle, or the lateral border of level IV	Medial edge of pec minor, or lateral border of level III	Lateral edge of pec minor, or lateral border of level II	Same as level II	5 mm medial from IMV, or sternum/clavicle
Lateral	Lateral edge of anterior scalene muscle (cranial) Lateral edge of clavicle (caudal)	*Lateral edge of subclavian artery until it crosses medial edge of pec minor (cranial)* *Medial edge of pec minor (caudal)*	Lateral edge of pec minor, or medial border of level I	*Medial edge of B/C until pec major no longer connects to arm (cranial)* *Connect lateral edge of pec major to ventral edge of L/T (caudal)*	Same as level II	5 mm lateral from the IMV, or 1st rib/pleura
Ventral	Dorsal edge of sternocleido-mastoid muscle and/or dorsal edge of clavicle	Dorsal edge of clavicle and/or dorsal edge of pec major	Dorsal edge of pec minor	*Dorsal edge of pec major*	Dorsal edge of pec major	Dorsal edge of sternum/clavicle/1st rib/ICM
Dorsal	*5 mm dorsal margin from most dorsal point of the vein in CTV, or ventral edge of subclavian artery/anterior scalene muscle/1st rib/ICM/pleura*	5 mm dorsal margin from most dorsal part of the vein in CTV, or ribs/ICM/ anterior serratus muscle	5 mm dorsal margin from most dorsal part of the vein in CTV, or ribs/ICM/serratus anterior muscle (cranial) Dorsal border of level I (caudal)	*Connect the ventral edge of the subscapular muscle, or L/T (most ventral) to the ribs and medial border (along thoracic wall)* *Exclude thoracodorsal vessels*	Ventral edge of pec minor	5 mm dorsal margin from IMV, or brachiocephalic vein/pleura/ pericardium

Borders that had to be changed from the ESTRO (and PROCAB) guideline are written in italics.

*B/C* biceps/coracobrachial muscle bundle, *ICM* intercostal muscles, *IMV* internal mammary vein, *L/T* latissimus dorsi/teres major muscle bundle, *pec major* major pectoral muscle, *pec minor* minor pectoral muscle.

The supplementary materials offer a detailed description on how to apply the guidelines, a guide to quickly recognise anatomical structures relevant for the delineation process, a contouring atlas, 3D representations of the CTVs and explanations related to rare findings.

As indicated in the ESTRO and PROCAB guidelines^13,14^, these guidelines are specifically created for elective nodal radiotherapy in early stage breast cancer patients. In advanced breast cancer cases, the guidelines can be used as a starting point, but patient specific adaptations should be made based on the available clinical and imaging information. When present, clips, seroma, or surgical parameters should be taken into account.

### Supraclavicular volume: level IV (Fig. 2)

This is the most medially located CTV, covering the supraclavicular volume. The cranial border is located 5 mm cranially from the last cranial slice that still shows the pleura. This level will contain the top of the subclavian artery arch. The caudal border starts 5 mm caudally from the first caudal slice where the subclavian vein and internal jugular vein fuse. The medial border is formed by the medial edge of the internal jugular vein (without margin) and more caudally, the medial edge of the brachiocephalic vein, or subclavian vein (without margin). The thyroid gland and the carotid artery are excluded from the CTV. The lateral border is the lateral edge of the anterior scalene muscle, until it is no longer visible, for these more caudal slices, the lateral border becomes the lateral edge of the clavicle. The ventral border is formed by the dorsal edge of the sternocleidomastoid muscle and the dorsal edge of the clavicle. For the dorsal border a 5 mm dorsal margin is respected, starting from the most dorsal part of the brachiocephalic, or subclavian vein. The anterior scalene muscle, the first rib, the intercostal muscles, the pleura and the subclavian artery (if visible) are excluded.

### Internal mammary nodes

This CTV contains the internal mammary nodes. The cranial border connects to the caudal border of level IV. The caudal border is located 5 mm caudal from the sternocostal joint of rib 4. Medially and laterally, a 5 mm margin around the internal mammary vein (IMV) is respected. Medially, the sternum and clavicle are excluded and laterally the ribs and pleura are excluded, if located within the 5 mm margin. The IMV is always located medially from the internal mammary artery. The ventral border is formed by the sternum, 1st rib, clavicle, or the intercostal muscles. For the dorsal border, a 5 mm margin dorsal from the IMV is respected, unless the ventral edge of the brachiocephalic vein (cranial), pleura, or pericardium is crossed. In this case these structures form the dorsal border.

### Infraclavicular volume: level III (Fig. 3)

This volume is located medially from the minor pectoral muscle and laterally from level IV. The cranial border is the first cranial slice where the subclavian artery crosses the lateral edge of both the first rib and the clavicle. The caudal border starts 5 mm caudal from the first cranial slice where the subclavian vein crosses the medial edge of the minor pectoral muscle. The medial border is the lateral border of level IV (if present), or the lateral edge of the clavicle. The lateral border follows the subclavian artery, until it crosses the medial edge of the minor pectoral muscle. At this point the medial edge of the minor pectoral muscle becomes the lateral border. Ventrally, this CTV is bordered by the dorsal edge of the clavicle and/or the major pectoral muscle. The subclavius muscle is excluded from the CTV. Dorsally, a 5 mm margin is respected, starting from the most dorsal part of the axillary vein. The serratus anterior muscle, the ribs and intercostal muscles are excluded.

### Axillary level 2: level II

This CTV is located dorsally from the minor pectoral muscle. The cranial limit starts on the first cranial slice where the axillary artery crosses the medial edge of the minor pectoral muscle. The caudal limit is reached when no more fatty tissue is observed between the minor pectoral muscle and the ribs/intercostal muscles. The medial border is the medial edge of the minor pectoral muscle, or the lateral edge of level III (if present). The lateral border is the lateral edge of the minor pectoral muscle, or the medial border of level I (if present). Ventrally this CTV is bordered by the dorsal edge of the minor pectoral muscle. For the dorsal border, a 5 mm margin from the most dorsal part of the axillary vein is respected. The ribs, intercostal muscles and the serratus anterior muscle are excluded. From the first cranial slice where the vein crosses the lateral border of the minor pectoral muscle (start of level I, caudal slices), the dorsal border becomes the same as the dorsal border of level I to ensure a smooth transition between the CTVs and to avoid missing lymphatic tissue between level I, level II and the ribs.

### Interpectoral space (Rotter space)

The interpectoral space is the area between the major and minor pectoral muscles, located ventrally from level II. The cranial, caudal, medial and lateral limit are the same as for level II. The ventral limit is the dorsal edge of the major pectoral muscle and the dorsal limit is the ventral edge of the minor pectoral muscle.

### Axillary level 1: level I (Fig. 4)

This CTV is located most laterally and caudally, covering the largest part of the axilla. The cranial border is located 5 mm in cranial direction from the first cranial slice where the axillary vein crosses the lateral border of the minor pectoral muscle. The caudal border is the point where the fourth rib is no longer visible at the sternum. The medial border is the lateral edge of the minor pectoral muscle, or the lateral border of level II. The lateral border is the medial edge of the biceps and coracobrachial muscle bundle (B/C) (cranial), until the major pectoral muscle is no longer connected to the arm. From this point forward, the lateral border is made by connecting the lateral edge of the major pectoral muscle directly to the ventral edge of the latissimus dorsi and teres major muscle bundle (L/T) (caudal). This line will become curved in the more caudal slices, because it follows the curvature of the ribs. The ventral border is the dorsal edge of the major pectoral muscle. The dorsal border is a connecting horizontal line from the ventral edge of the subscapular muscle, or the L/T (whichever is located more ventrally) (lateral border endpoint) towards the ribs, intercostal muscles, or serratus anterior muscle. The dorsal border continues along the surface of these structures, until reaching the medial border. If the thoracodorsal vessels are visible, they should be excluded from the CTV.

## Discussion

Like the ESTRO and PROCAB guidelines, our PCP specific guidelines are primarily based on the vasculature, because the lymphatics are located in close proximity to the veins^13,14,21^. Vessel based guidelines are less patient and position dependent, compared to bony landmarks as reference points^13,14,17^. However, some adaptations were still needed to obtain the final PCP specific guidelines. When choosing the alternative borders, the position of the vasculature was the main focus, to respect this ESTRO and PROCAB principle. Borders that had to be adapted from the ESTRO and PROCAB guidelines in order to obtain PCP specific guidelines were the following: the cranial and dorsal border of level IV, the lateral border of level III and the cranial, lateral, ventral and dorsal borders of level I.

The cranial border of level IV was adapted, because the subclavian artery arch was not easily recognizable on all patient CT scans. By taking the pleura as a reference point (easy to locate) and adding 5 mm in cranial direction, the top of the subclavian artery arch will be included, whether or not it can be located. The top of the subclavian artery remains the cranial border, using the pleura as reference simply offers an easier alternative to locate it (Fig. 2a,b). The 5 mm margin dorsal from the subclavian or brachiocephalic vein is chosen to resemble the PROCAB border (ventral edge of the subclavian artery, or the anterior scalene muscle). It describes the ventral edge of the subclavian artery for most patients and it can be applied even when the artery is not visible/located. The dorsal border was not indiscriminately extended to the pleura (like in ESTRO), because this can cause an increased lung dose in more obese patients in PCP (Fig. 2c).

The lateral border of level III was adapted to account for the more latero-lateral course of the vessels that results from keeping the arm next to the body, compared to elevating it. Additionally, unlike in SP, the cranial border of level III overlaps with, or is located closer to the (much more lateral) insertion of the minor pectoral muscle. By following the axillary artery, until it passes the medial edge of the minor pectoral muscle, the space between the lateral edge of the axillary artery and the insertion of the minor pectoral muscle is excluded. In the most cranial slices, this spares a lot of healthy tissue in PCP (Fig. 3).

Level I shows the most changes, because the change in arm position has the biggest impact on the spatial orientation of the muscles in this area. For the cranial border, 5 mm cranial to the first cranial slice where the axillary vein crosses the lateral edge of the minor pectoral muscle is chosen. The humeral head is not mentioned as a restriction, because it is no longer in close proximity to the target volume in PCP. The lateral border had to be adapted, because the latissimus dorsi muscle (and deltoid muscle), and major pectoral muscle are no longer adjacent in the cranial slices. As an alternative, the B/C was chosen, because it is the most adjacent structure to the major pectoral muscle and it resulted in a CTV most similar to the ESTRO and PROCAB CTV (Fig. 4a,b). Choosing the B/C as lateral border in the cranial slices in PCP spares the axillary vein dorsally located from the B/C, as it runs towards the arm. Excluding the vessels running towards the arm, similar to supine position delineations using ESTRO and PROCAB, could have a positive effect on lymphedema prevention. The ventral border now solely consists of the major pectoral muscle. Finally, the dorsal border was adapted to account for the slightly altered relation between the L/T and subscapular muscle in the new position. By taking either the ventral edge of the L/T, or the ventral edge of the subscapular muscle (whichever is located more ventrally), a stable target volume is obtained that is applicable for all patients and still resembles the ESTRO and PROCAB volume.

By taking the existing ESTRO and PROCAB guidelines for supine position into account and by using a relatively big number of datasets, new delineation guidelines for PCP were developed. These new PCP specific guidelines incorporate anatomical variability between patients and are easily and consistently applicable, even for people with limited previous experience with delineations in PCP.

## Supplementary Information

Supplementary Information 1.

Supplementary Information 2.

Supplementary Information 3.

Supplementary Information 4.
