# Supplementary material for: Delineation guidelines for the lymphatic target volumes in ‘prone crawl’ radiotherapy treatment position for breast cancer patients
Source: Sci Rep. 2021 Nov 18;11:22529. doi: 10.1038/s41598-021-01841-y (PMC8602302; doi:10.1038/s41598-021-01841-y)

Prone crawl delineation guideline, application guide

Linked to manuscript ‘Delineation guidelines for the lymphatic target volumes in ‘prone crawl’ radiotherapy treatment position for breast cancer patients ’

by

Michael E. J. Stouthandel, Françoise Kayser, Vincent Vakaet, Ralph Khoury, Pieter Deseyne, Chris Monten, Max Schoepen, Vincent Remouchamps, Alex De Caluwé, Guillaume Janoray, Wilfried De Neve, Stephane Mazy, Liv Veldeman, Tom Van Hoof.

Contour colour legend

vein (V)

artery (A)

Level IV (**4**)

Level III (**3**)

Level II (**2**)

Level I (**1**)

Interpectoral nodes (**IP**)

Internal mammary nodes (**IM**)

sternocleidomastoid muscle (SCM)

anterior scalene muscle (AS)

biceps/coracobrachial muscle (B/C)

serratus anterior muscle (SA)

major pectoral muscle (MaP)

lattissimus dorsi/teres major muscle (L/T)

subscapular muscle (SS)

subclavius muscle (SM)

bones (light grey)

minor pectoral muscle (MiP)

Level IV cranial border

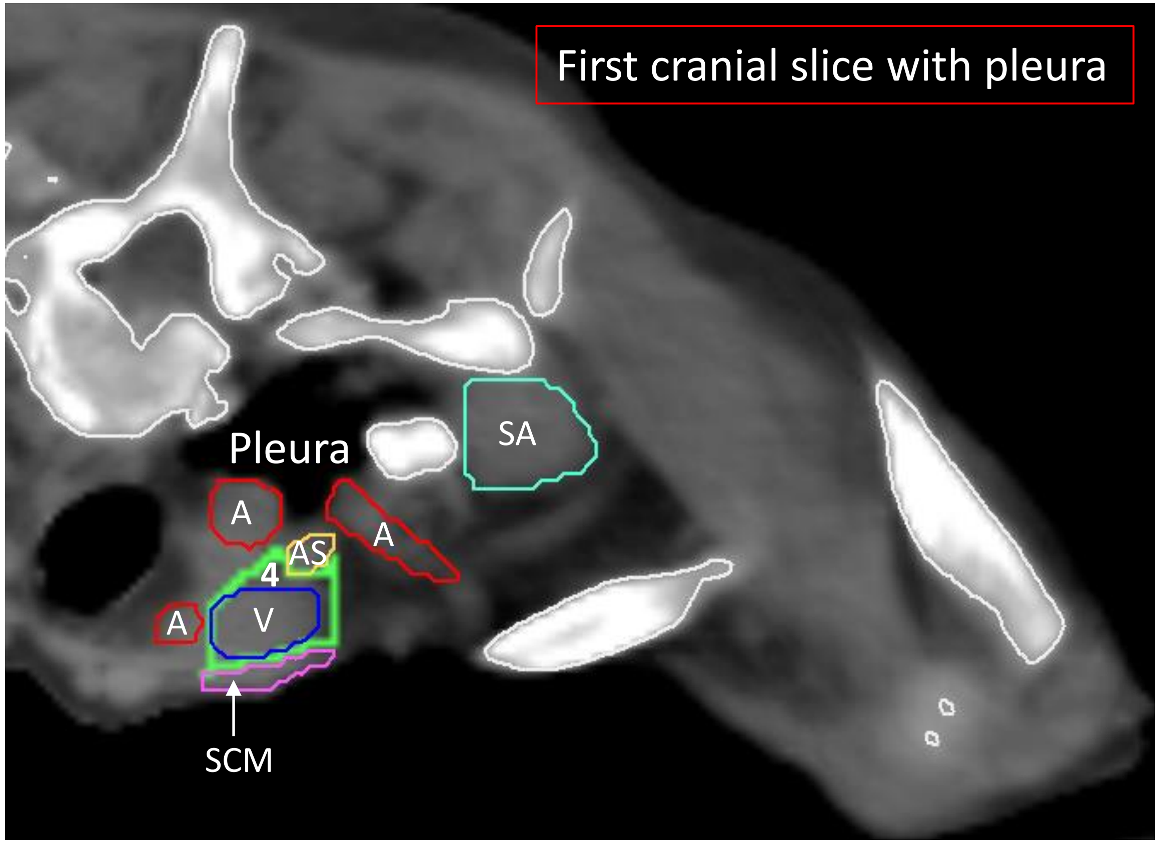

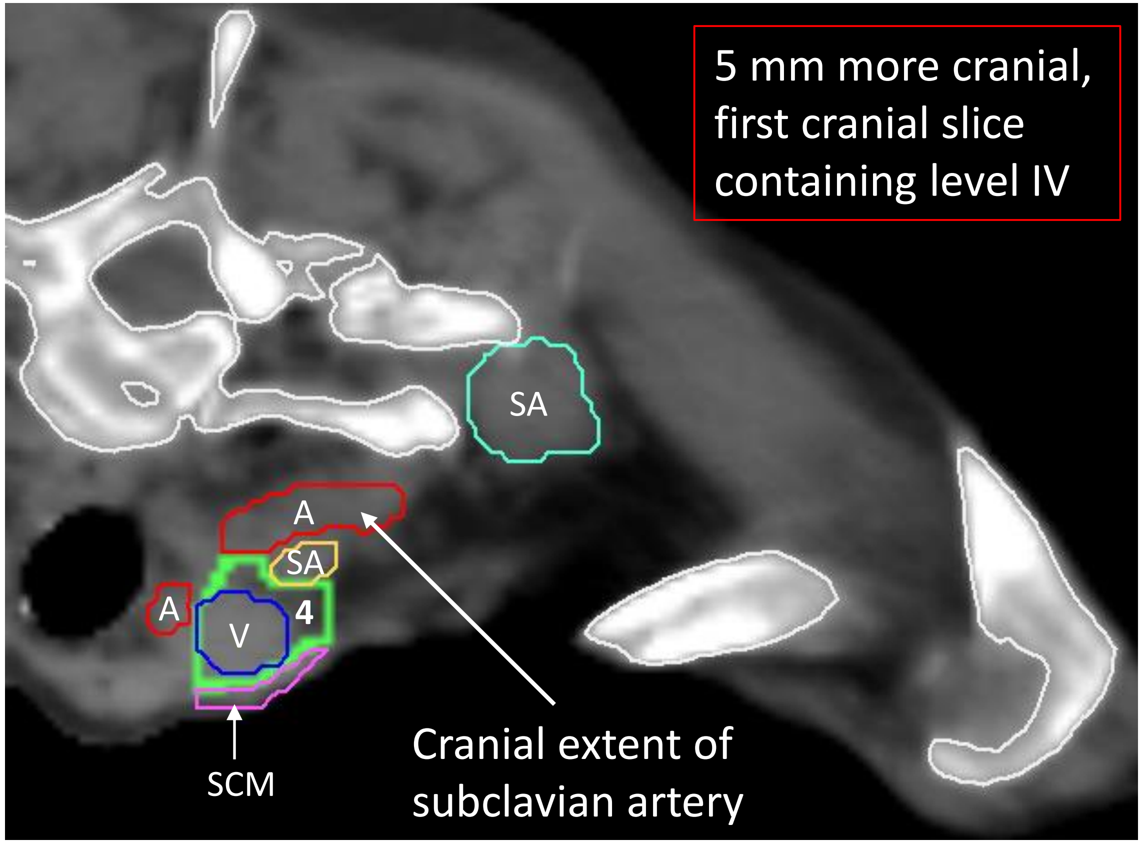

- Taking 1 slice more cranial from the pleura includes the cranial extent of the subclavian artery in all datasets.
- Using the pleura as reference point makes it easier to locate the cranial extent of the subclavian artery (ESTRO border) in datasets where it is not easily recognised.

Level IV caudal border

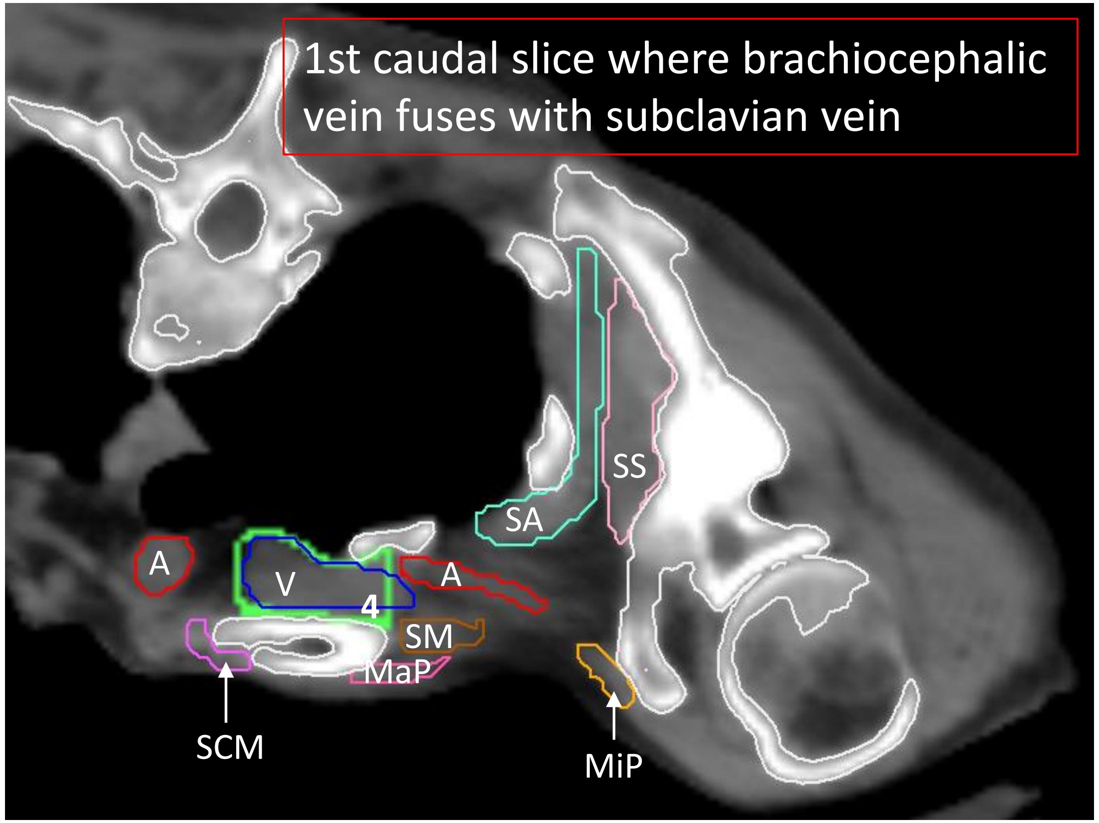

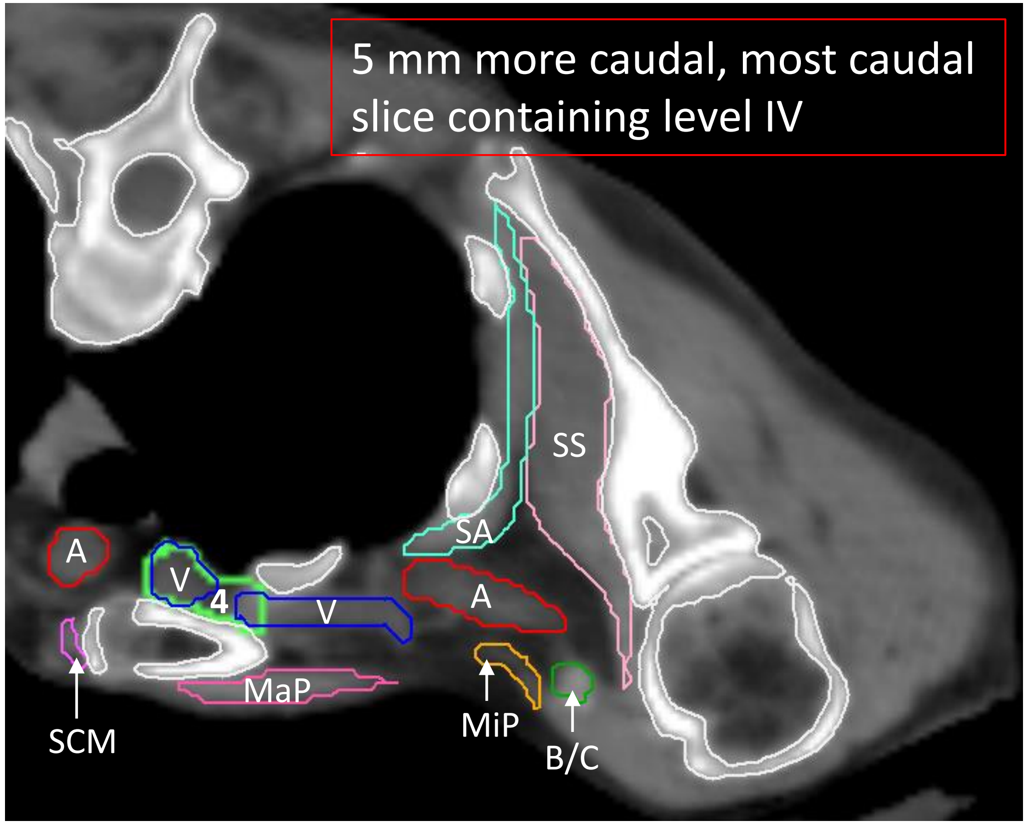

Explanation of first cranial, or first caudal slice for cranial or caudal border

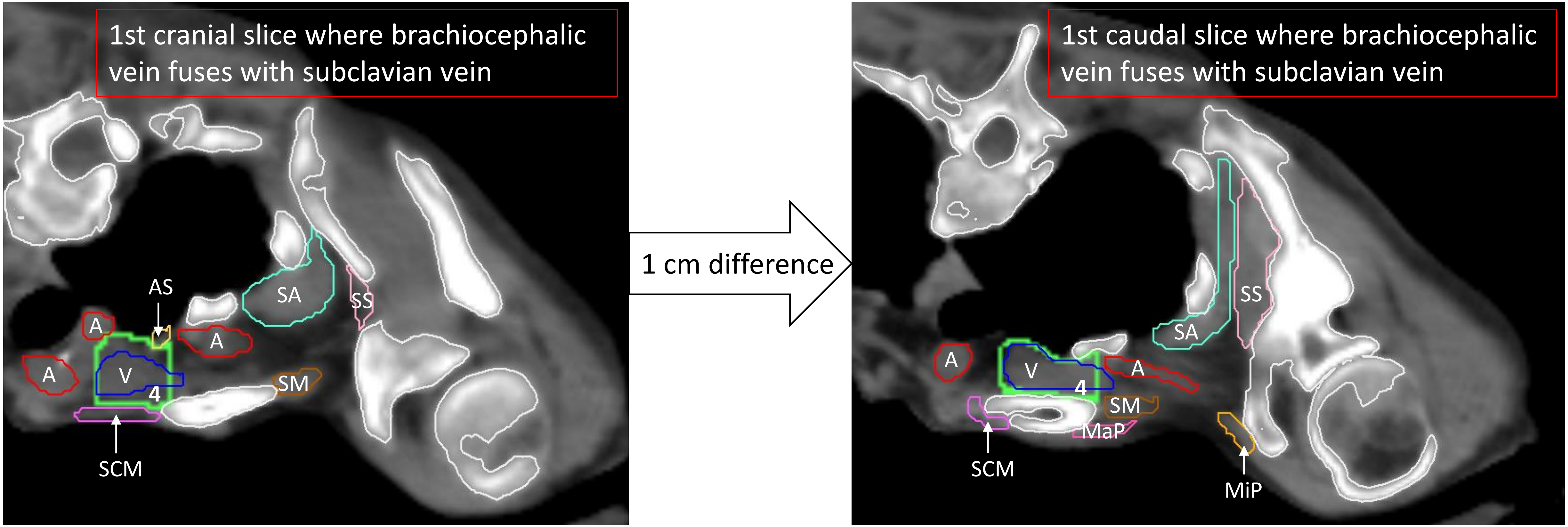

* There is a necessity to specify if it is the first cranial or the first caudal slice where a structure is encountered, to differentiate between the direction you scroll through the slices (cranio-caudal, or caudo-cranial)

Level IV medial border

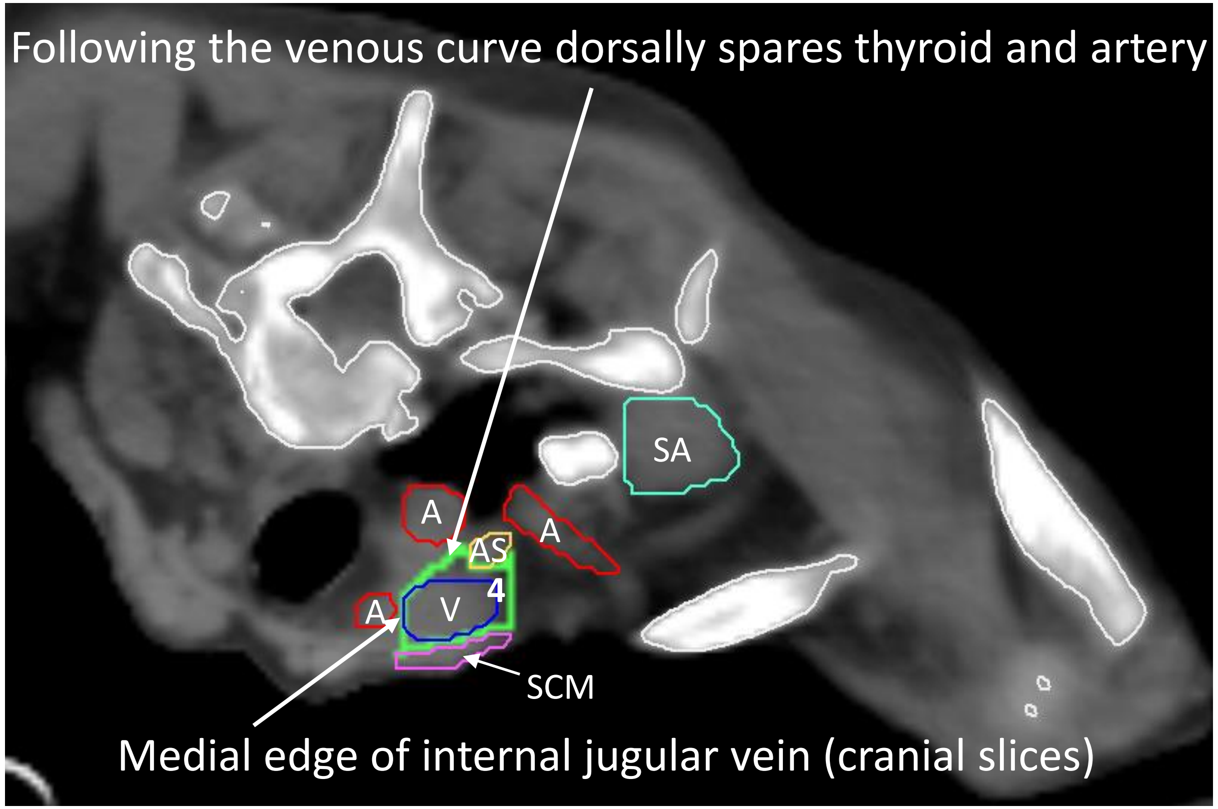

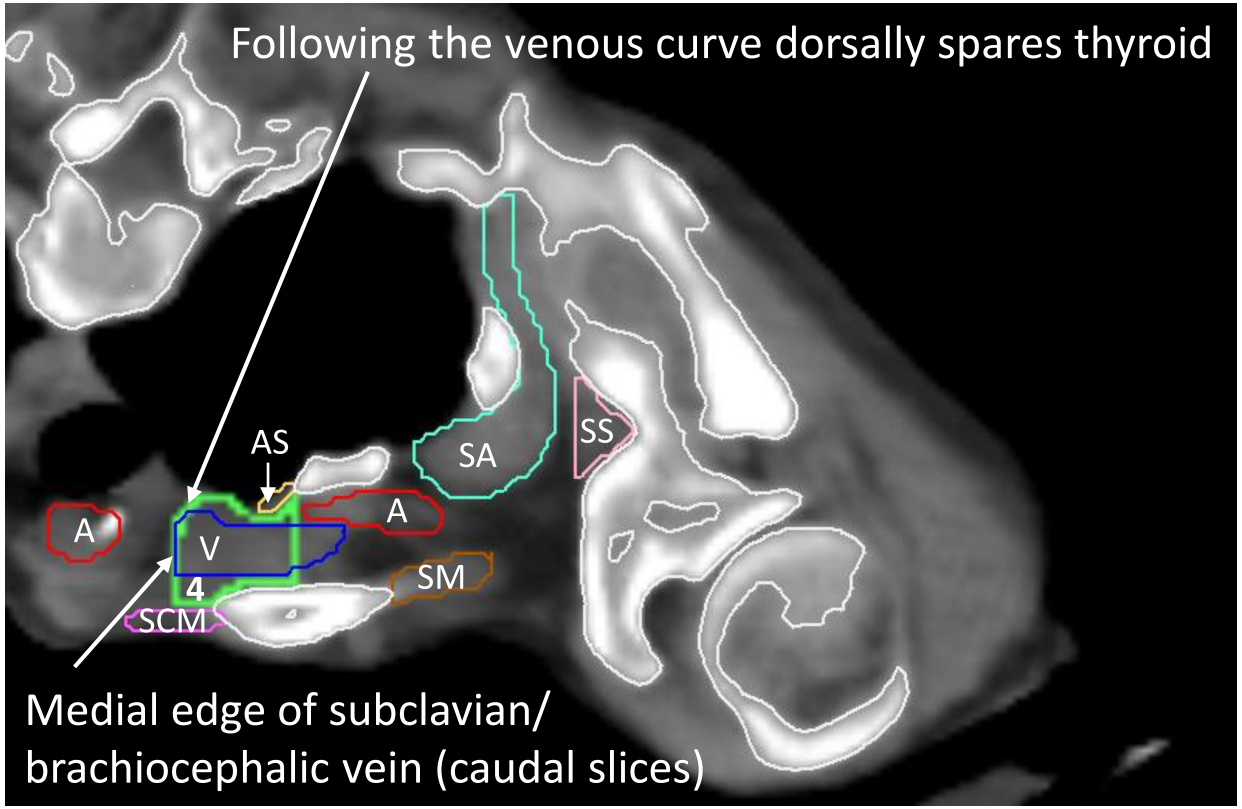

Level IV lateral border

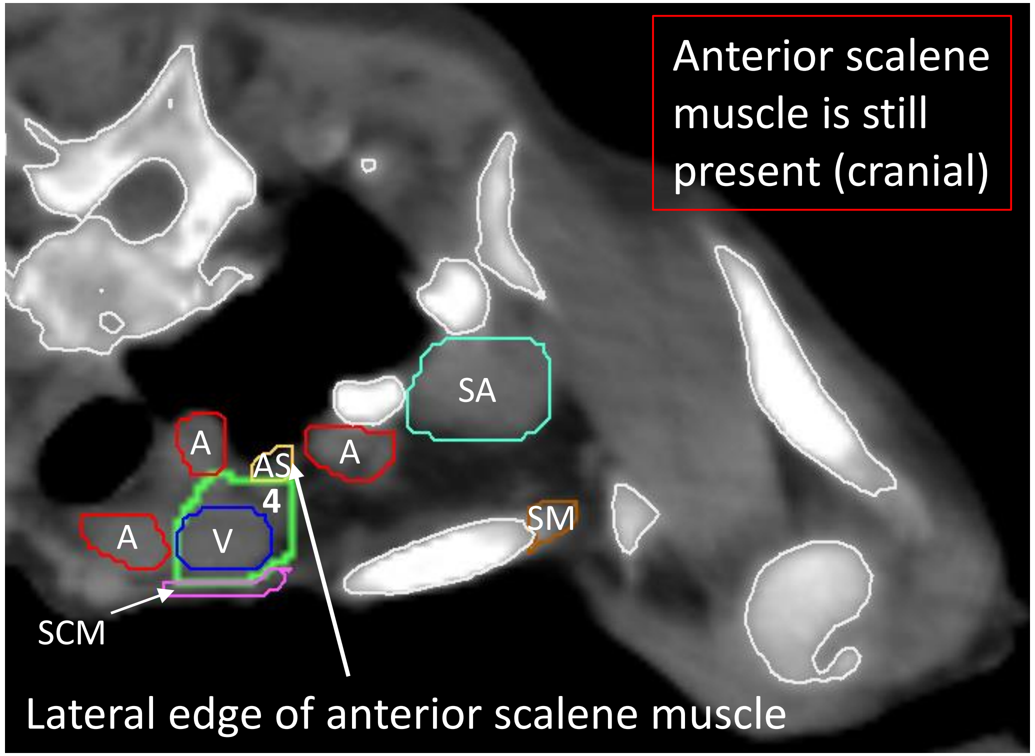

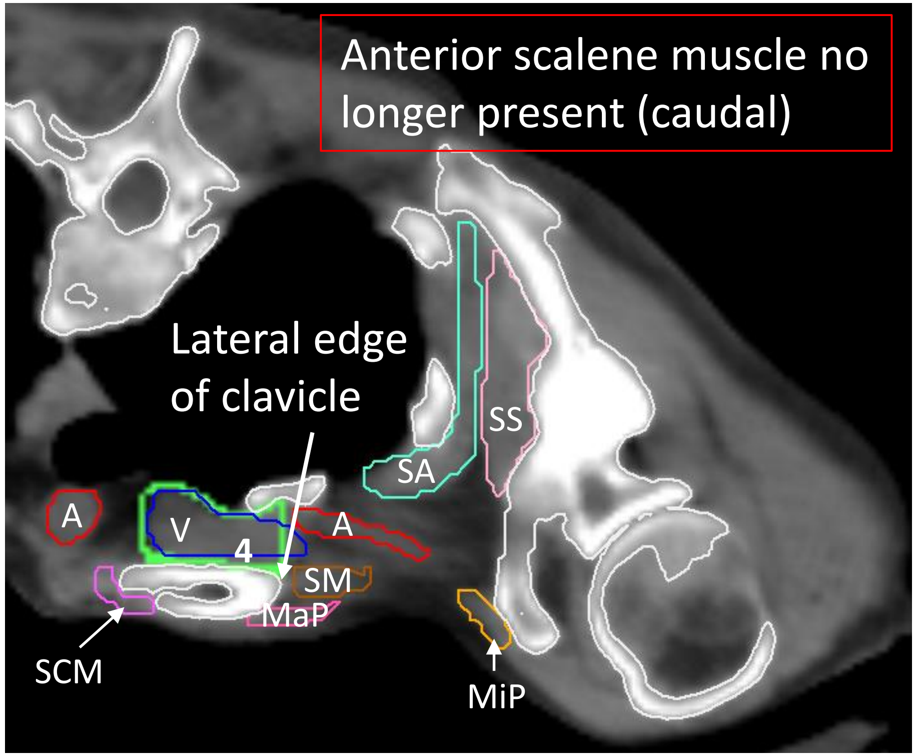

Level IV ventral border

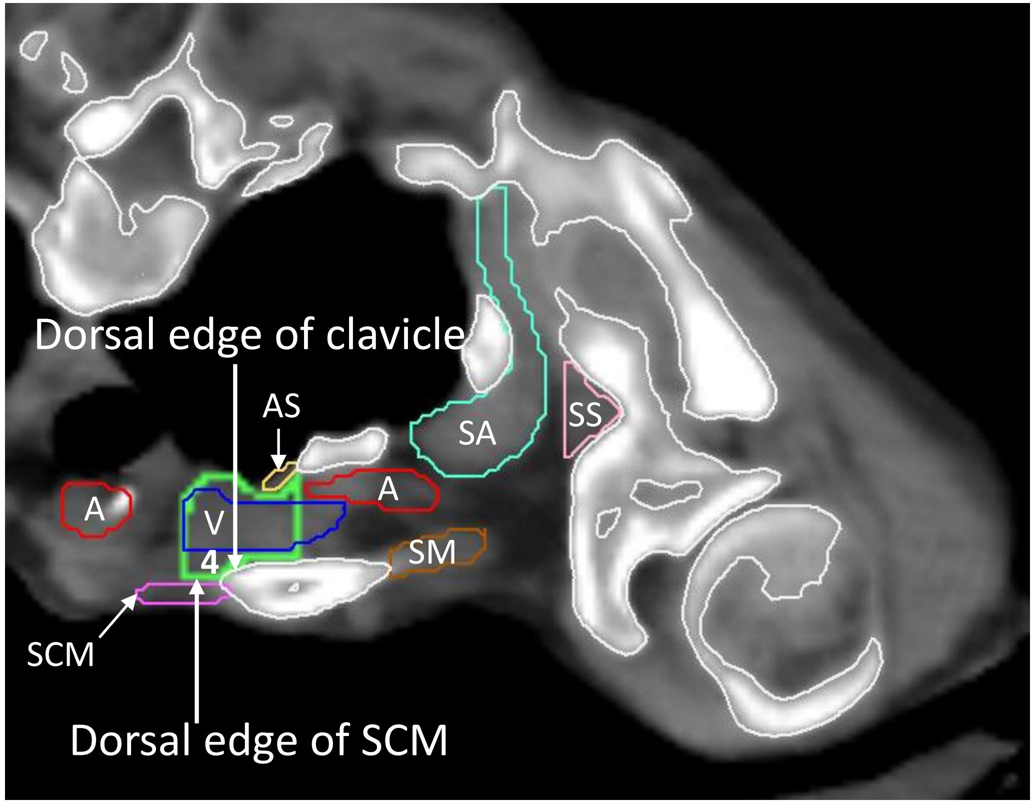

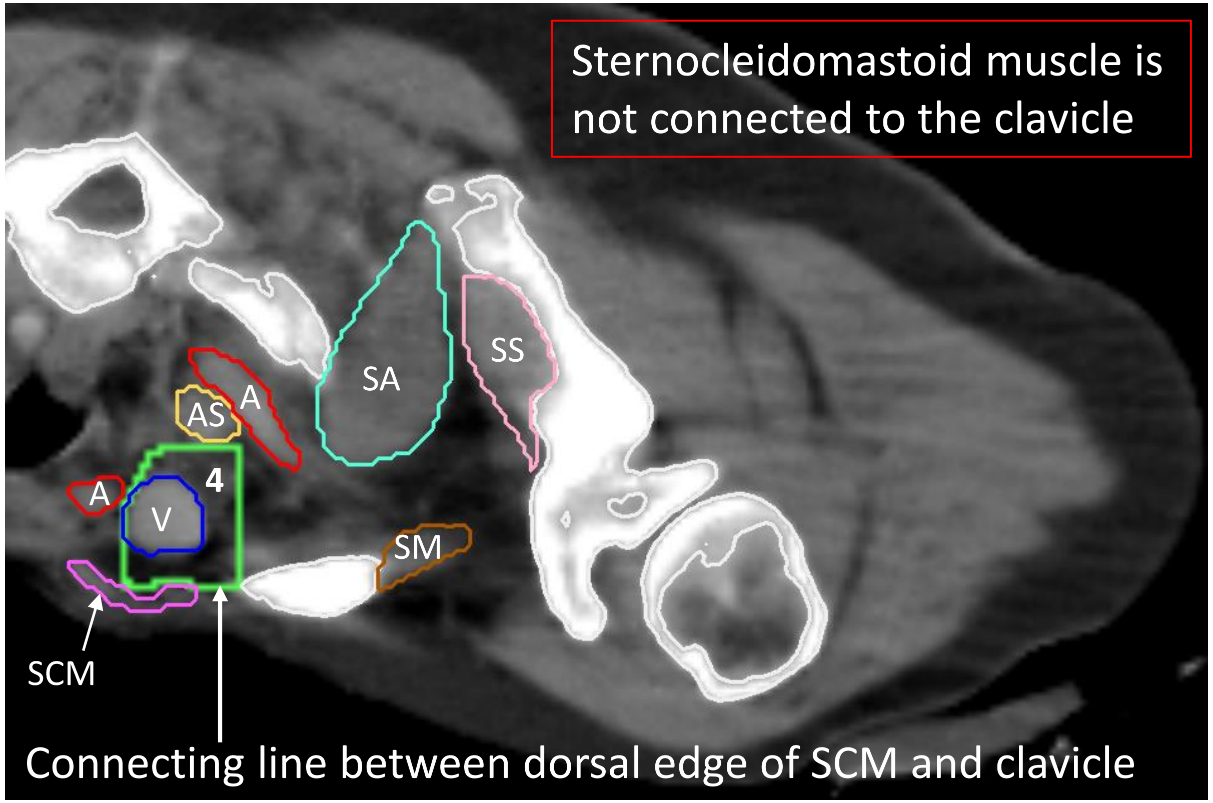

Level IV dorsal border

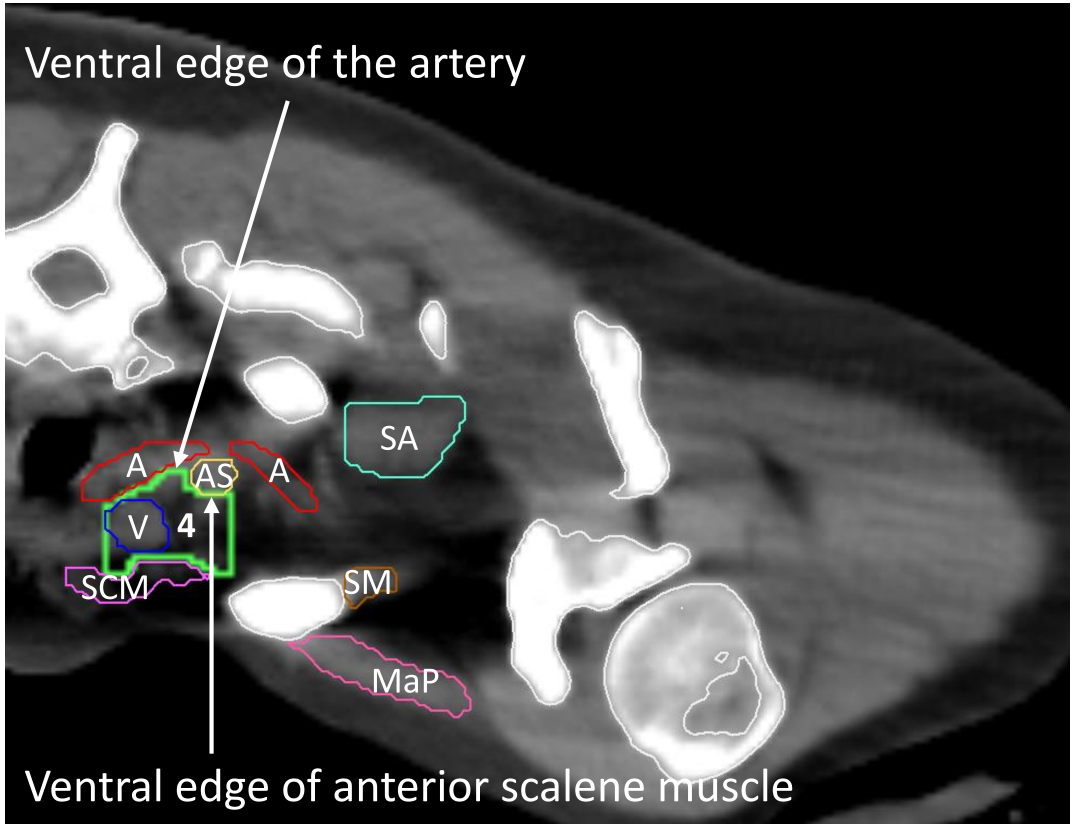

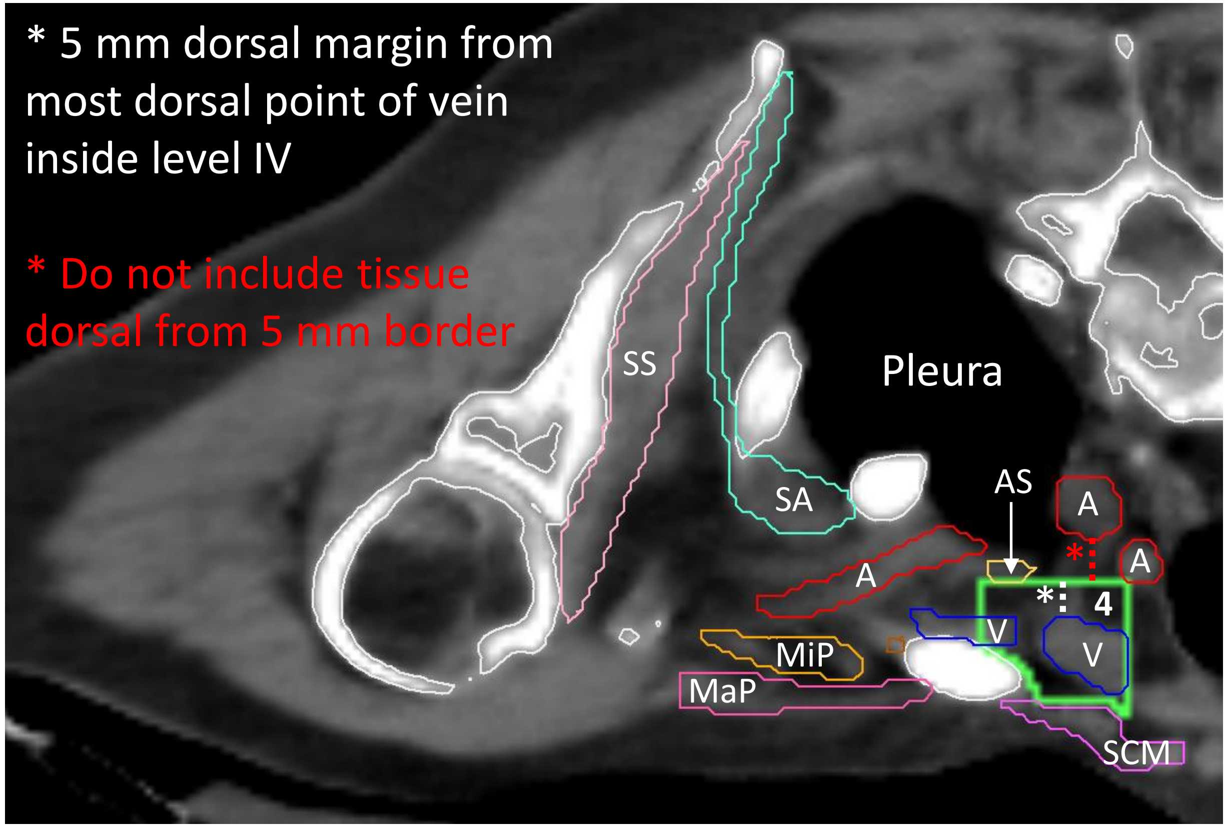

Level III cranial border

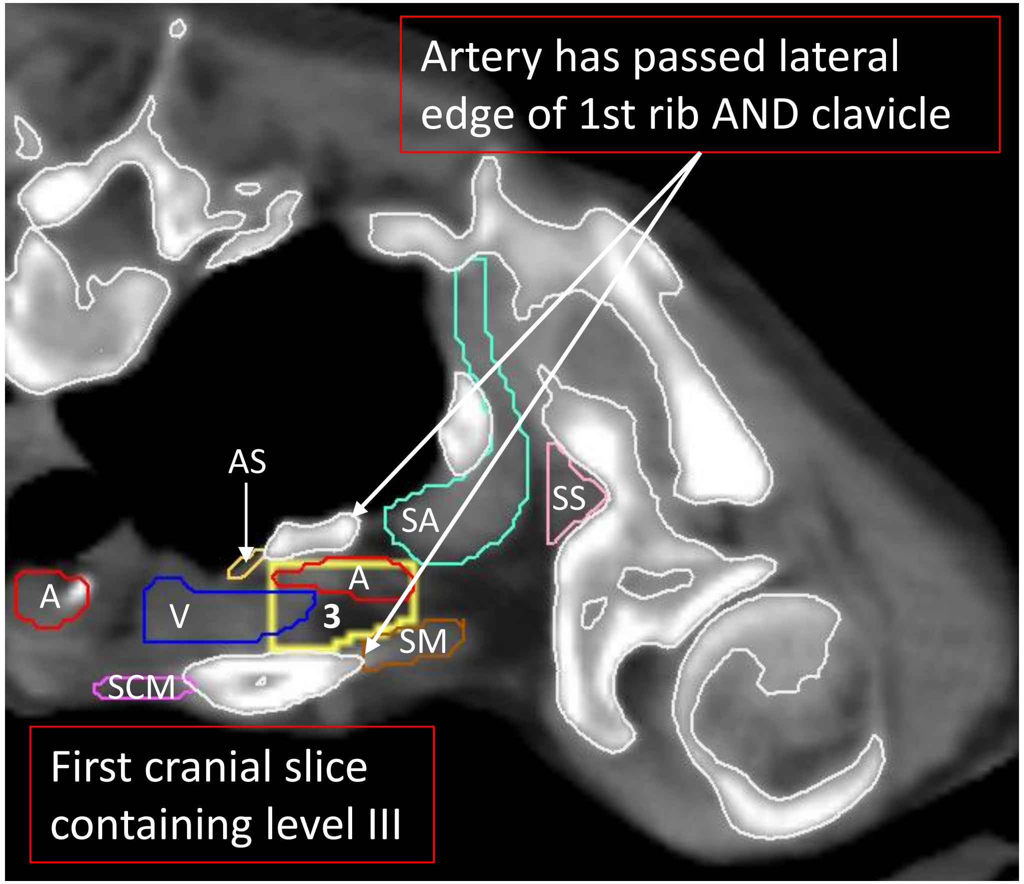

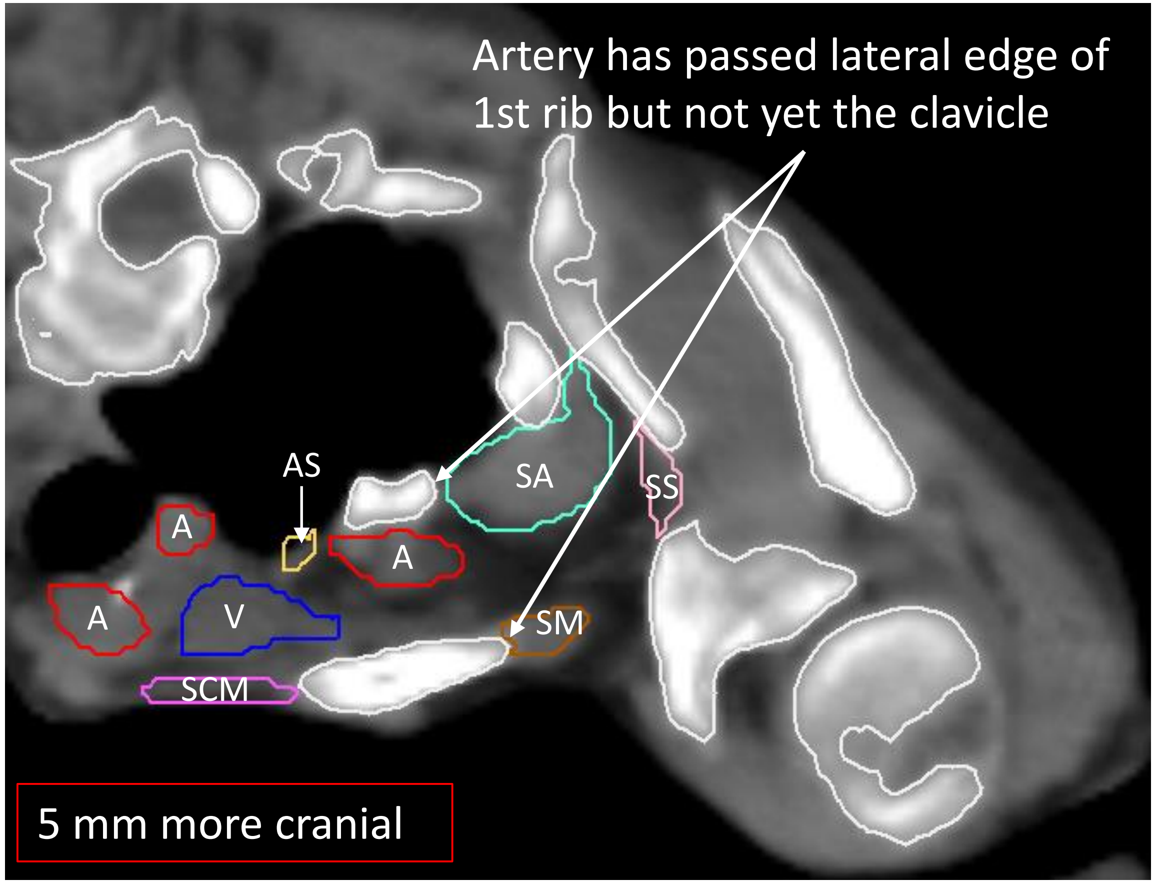

Level III caudal border

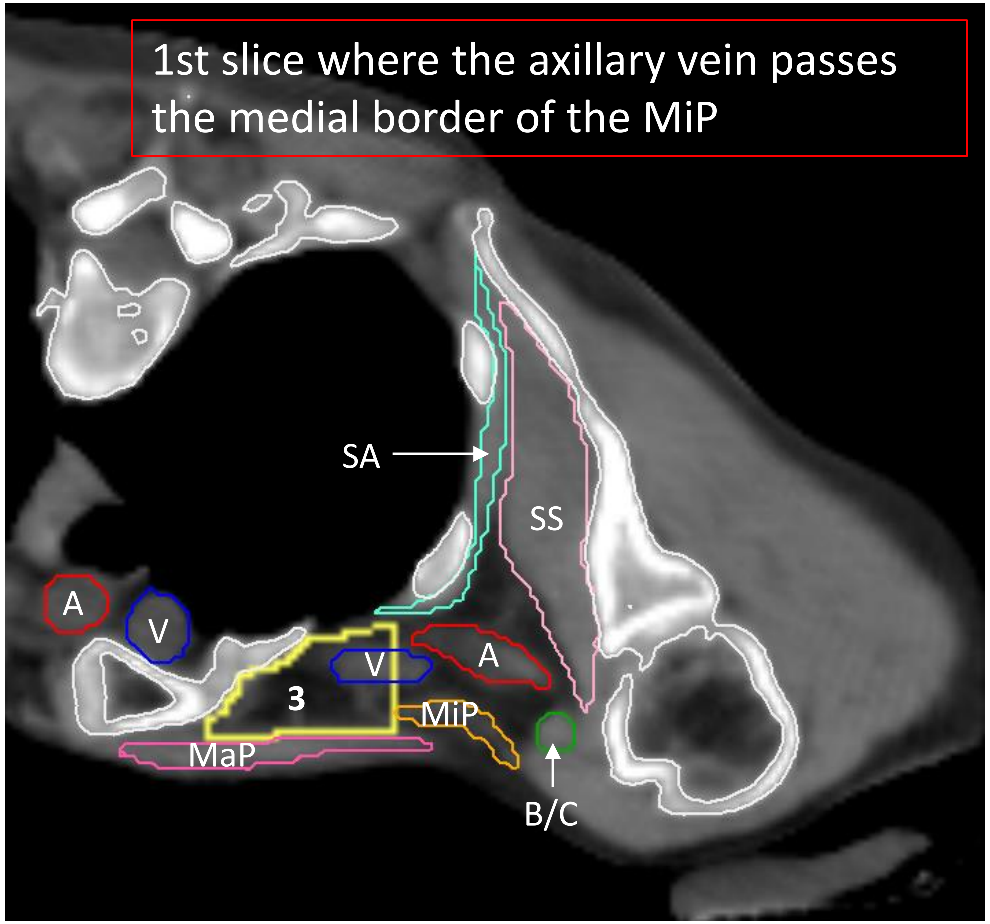

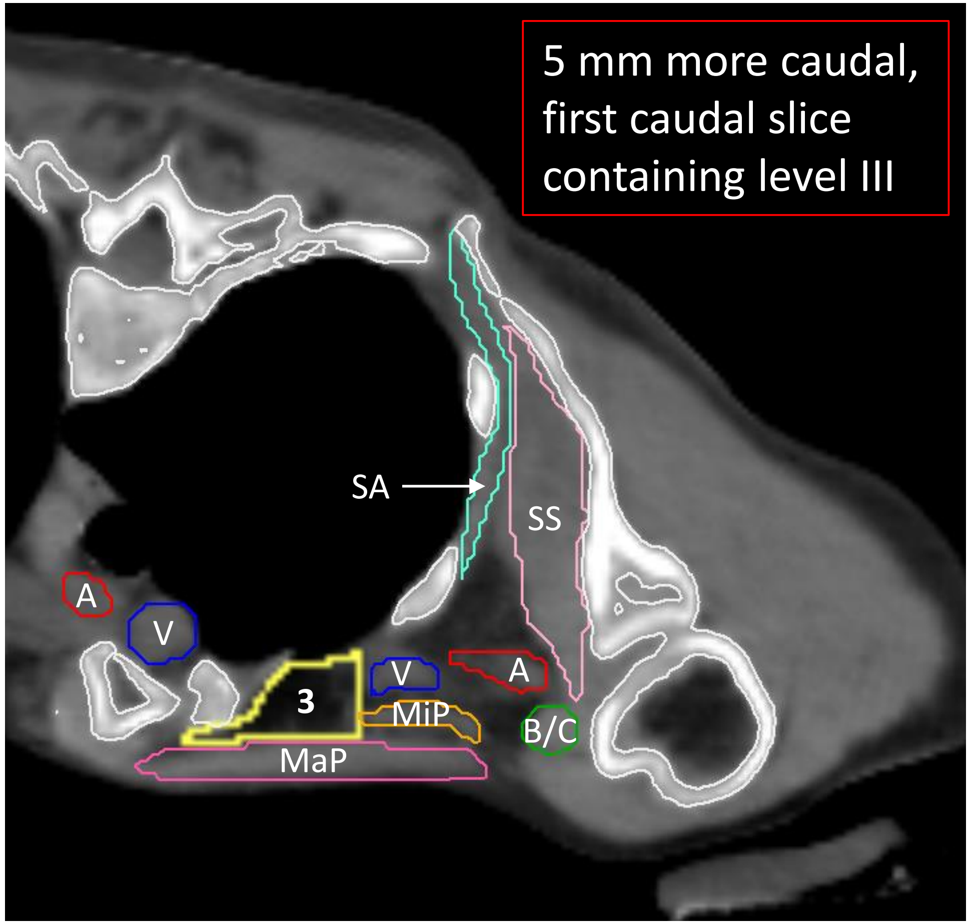

* The additional 5 mm more caudal always covers the remaining venous volume between the medial edge of the minor pectoral muscle and the lateral edge of the clavicle in level III.

Level III caudal border, rare finding

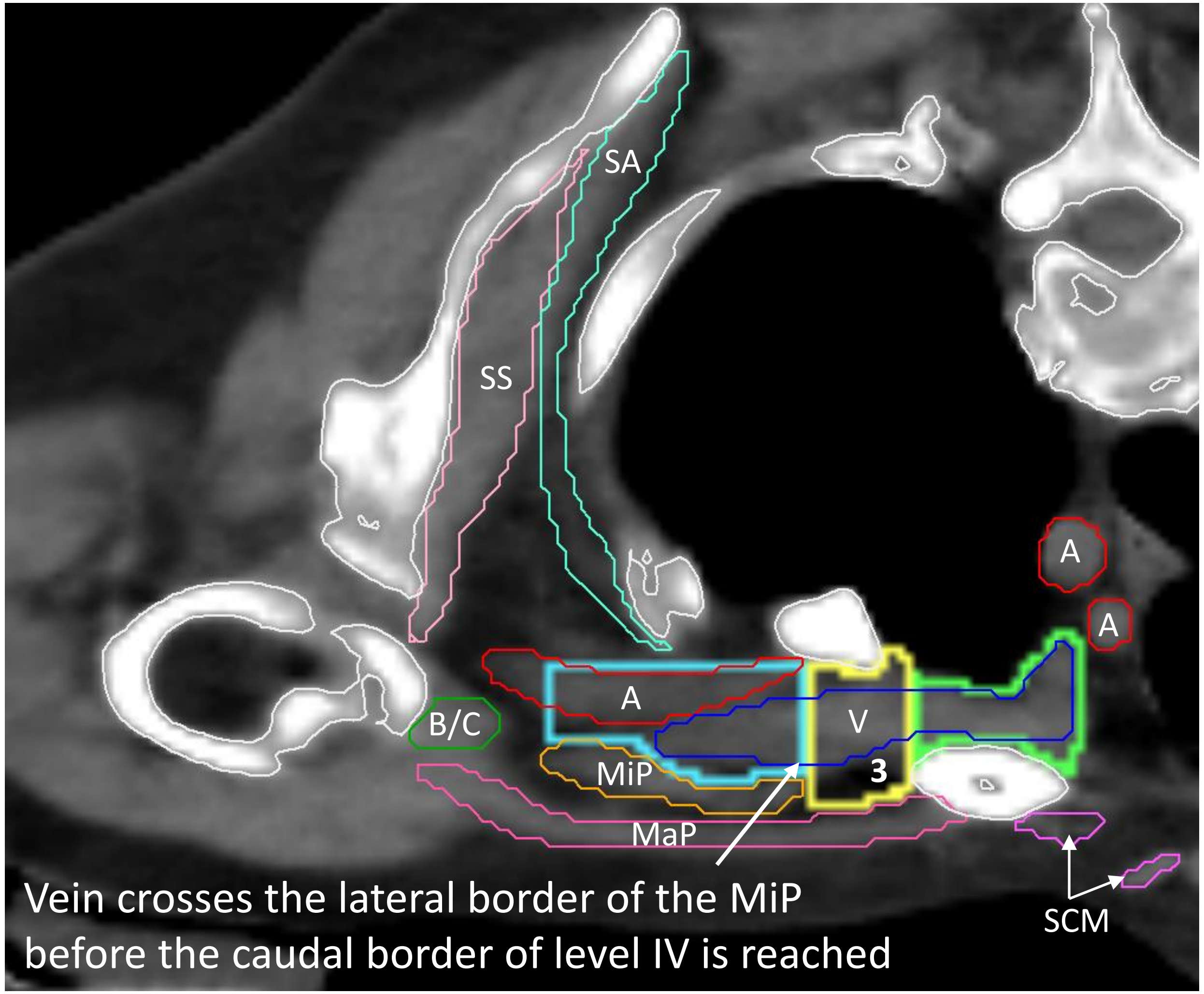

To make sure that no target tissue is missed when this rare finding occurs, the caudal border of level III can be extended to the caudal border of level IV. In other words, in case level III stops before the most caudal slice containing level IV, extend level III until the final slice containing level IV is reached.

The adaptation for this rare finding added 2 additional slices until the caudal border of level IV was reached in the 1 CT dataset that this occured. Adding these 2 slices in caudal direction succesfully included the entire part of the axillary vein that was located between level IV and the medial edge of the minor pectoral muscle

Level III medial border

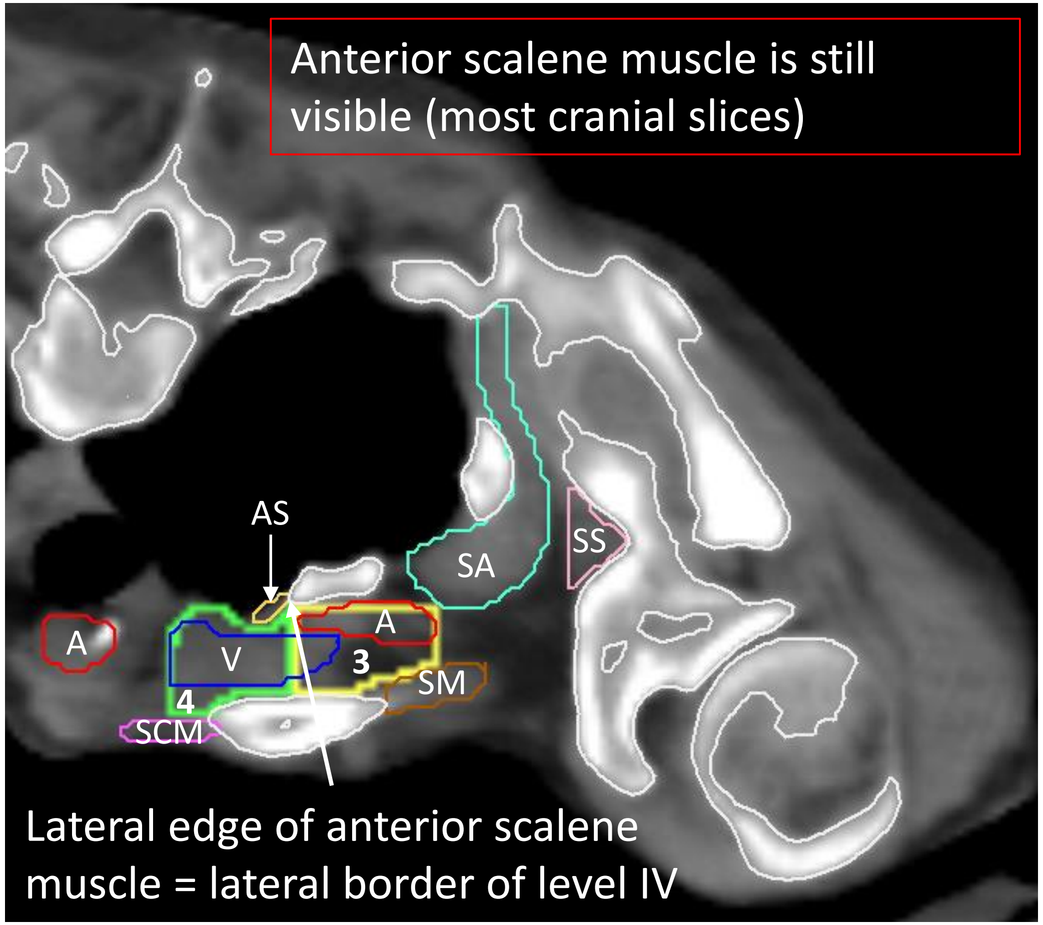

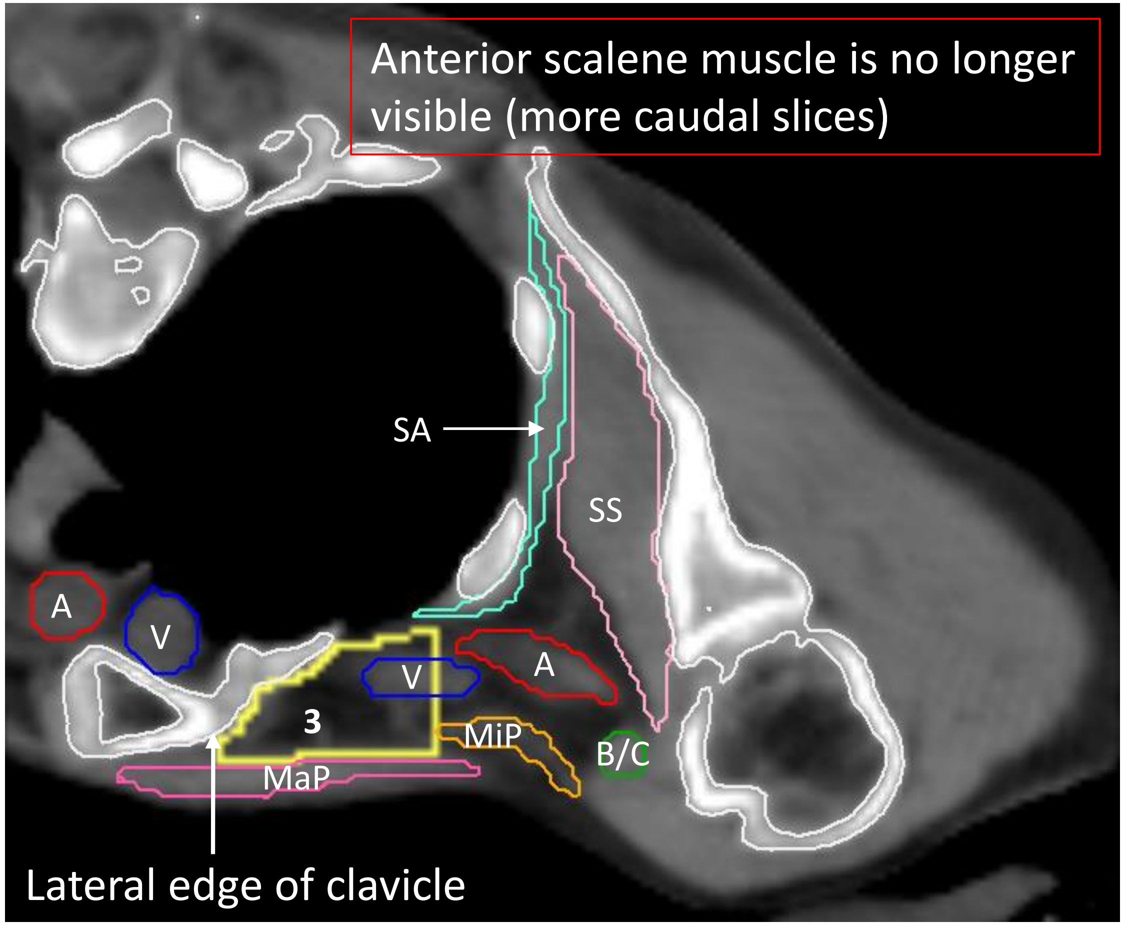

Level III lateral border

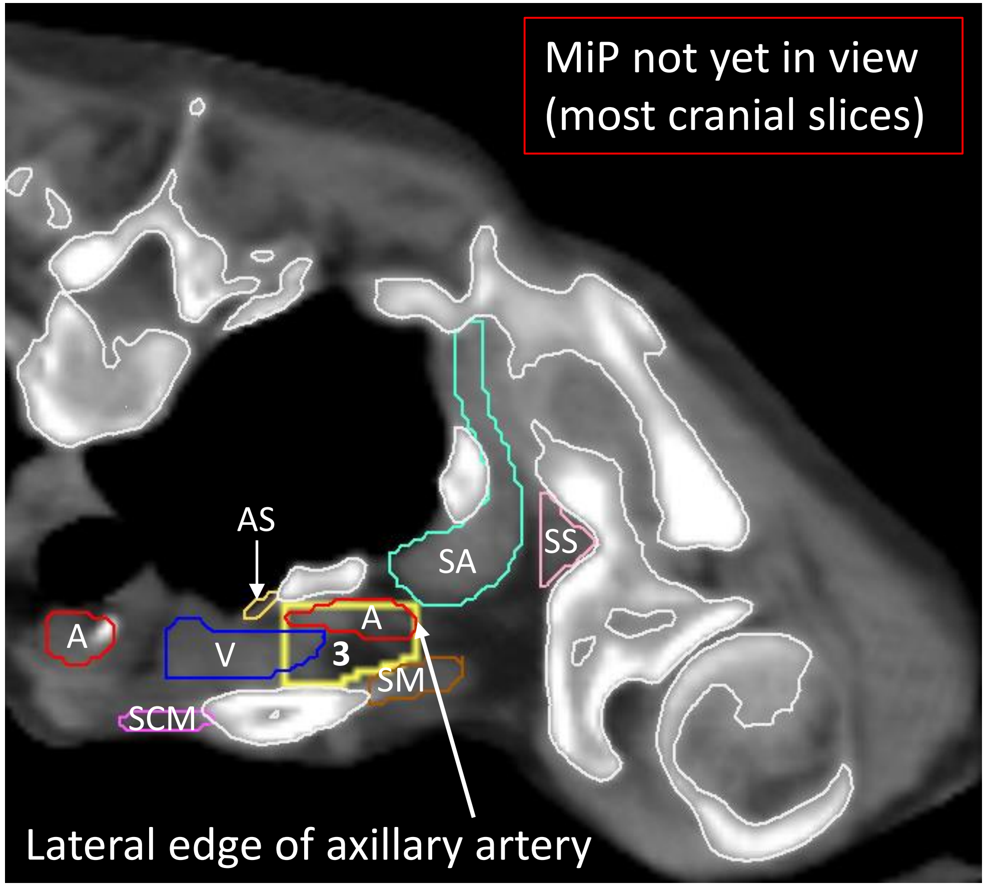

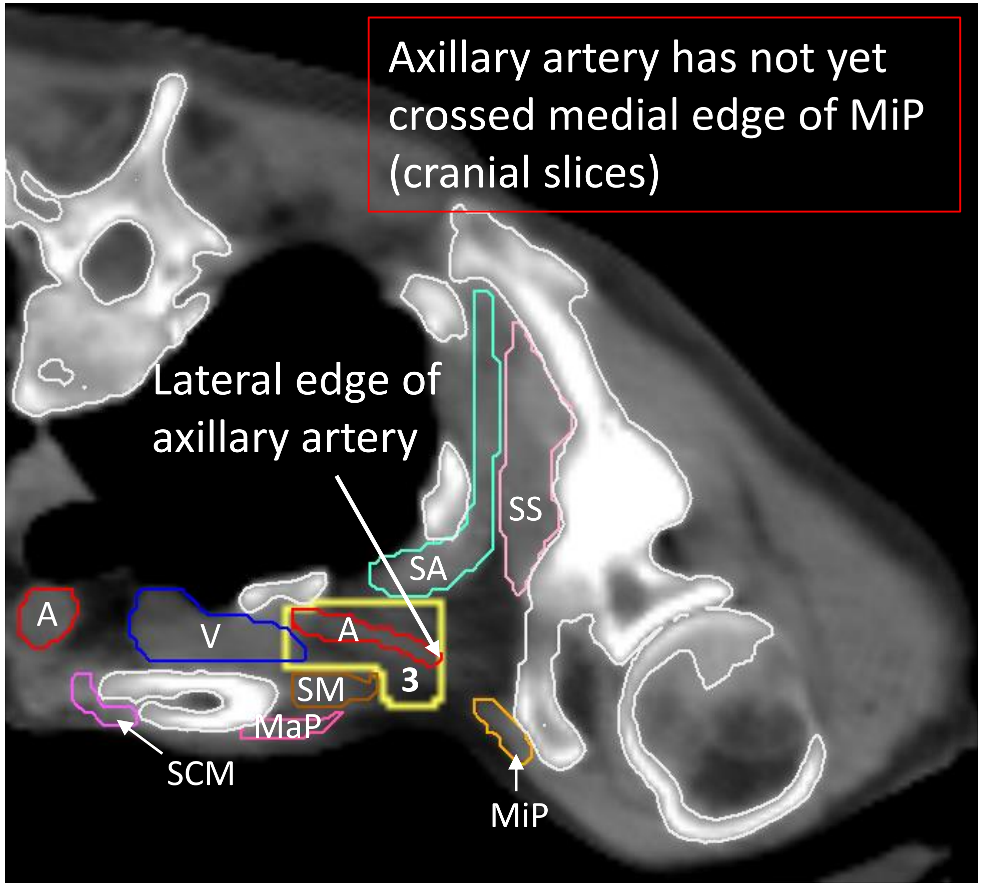

Level III lateral border

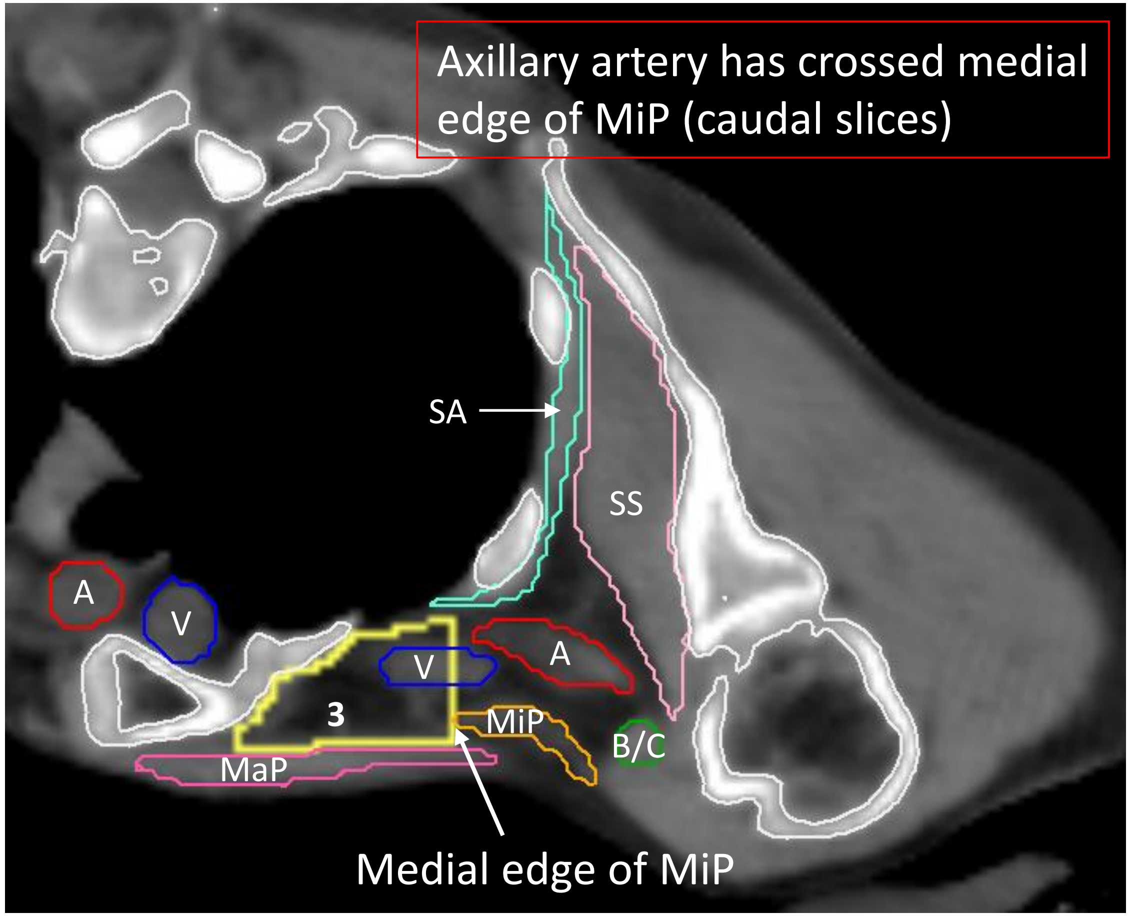

Level III ventral border

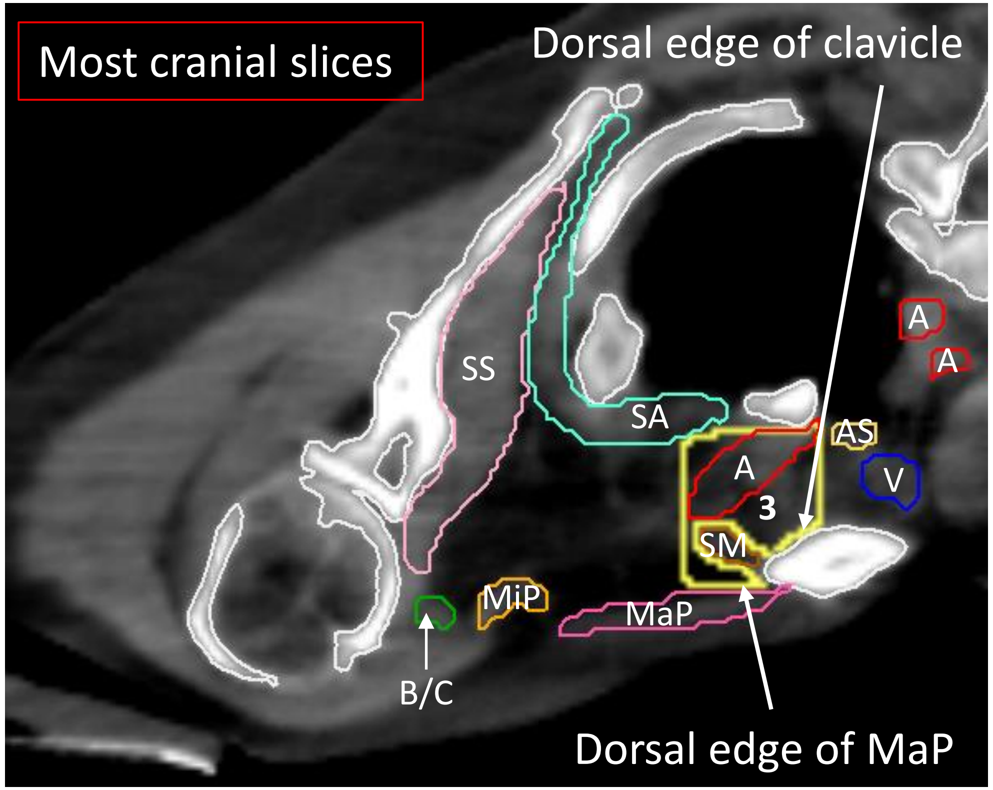

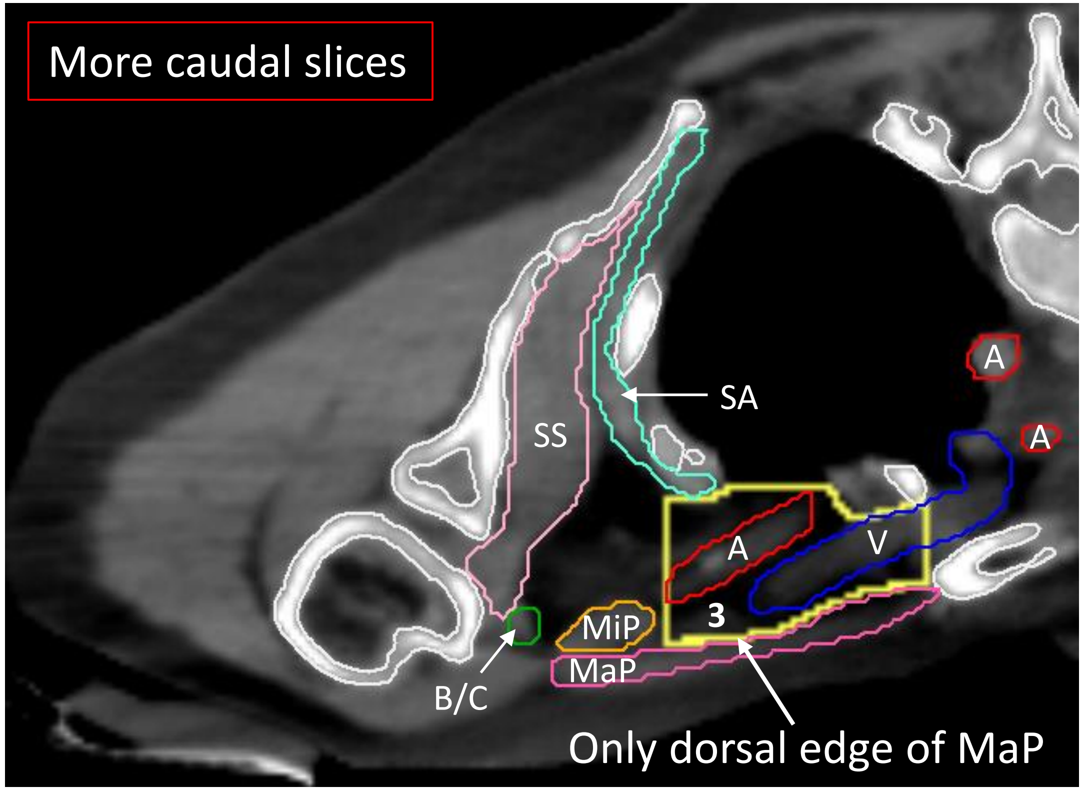

Level III ventral, rare findings

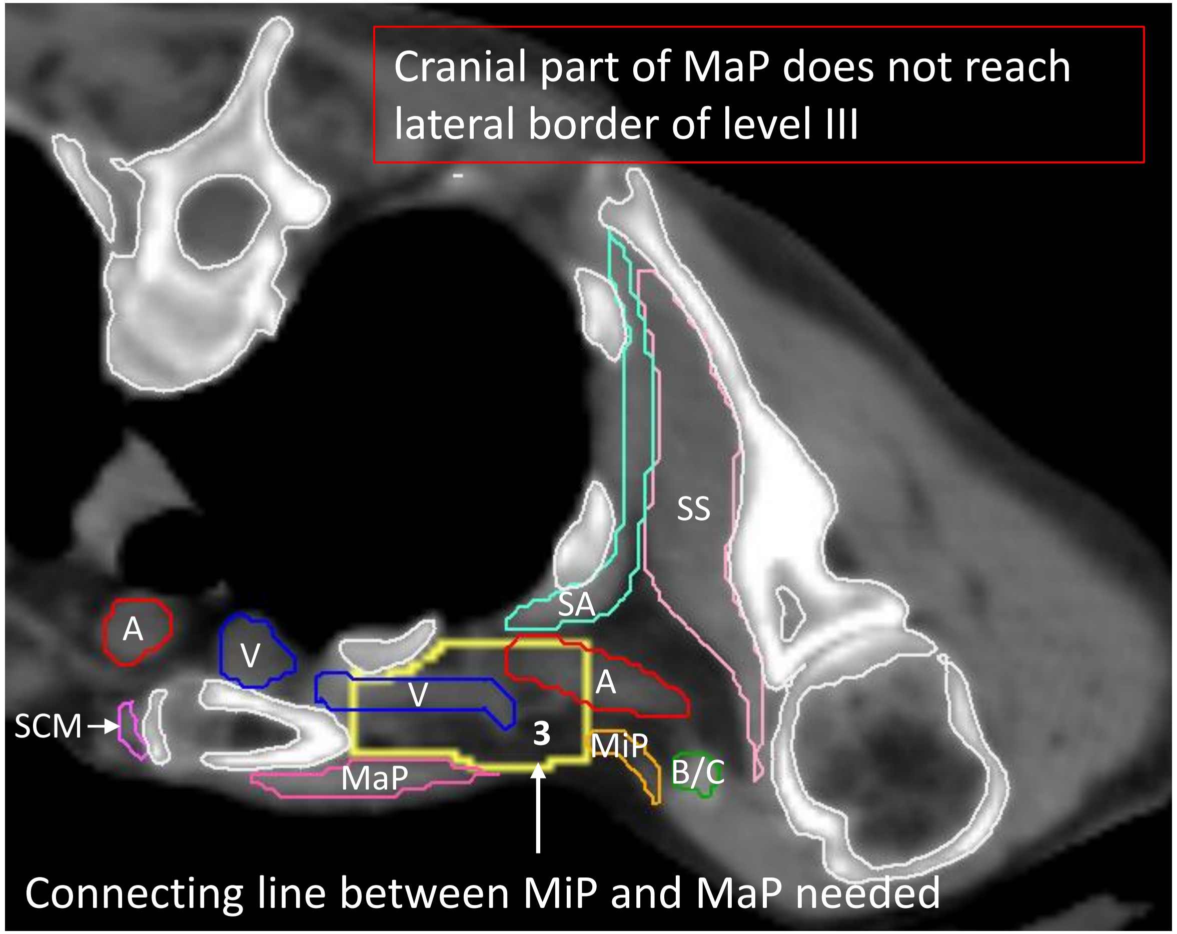

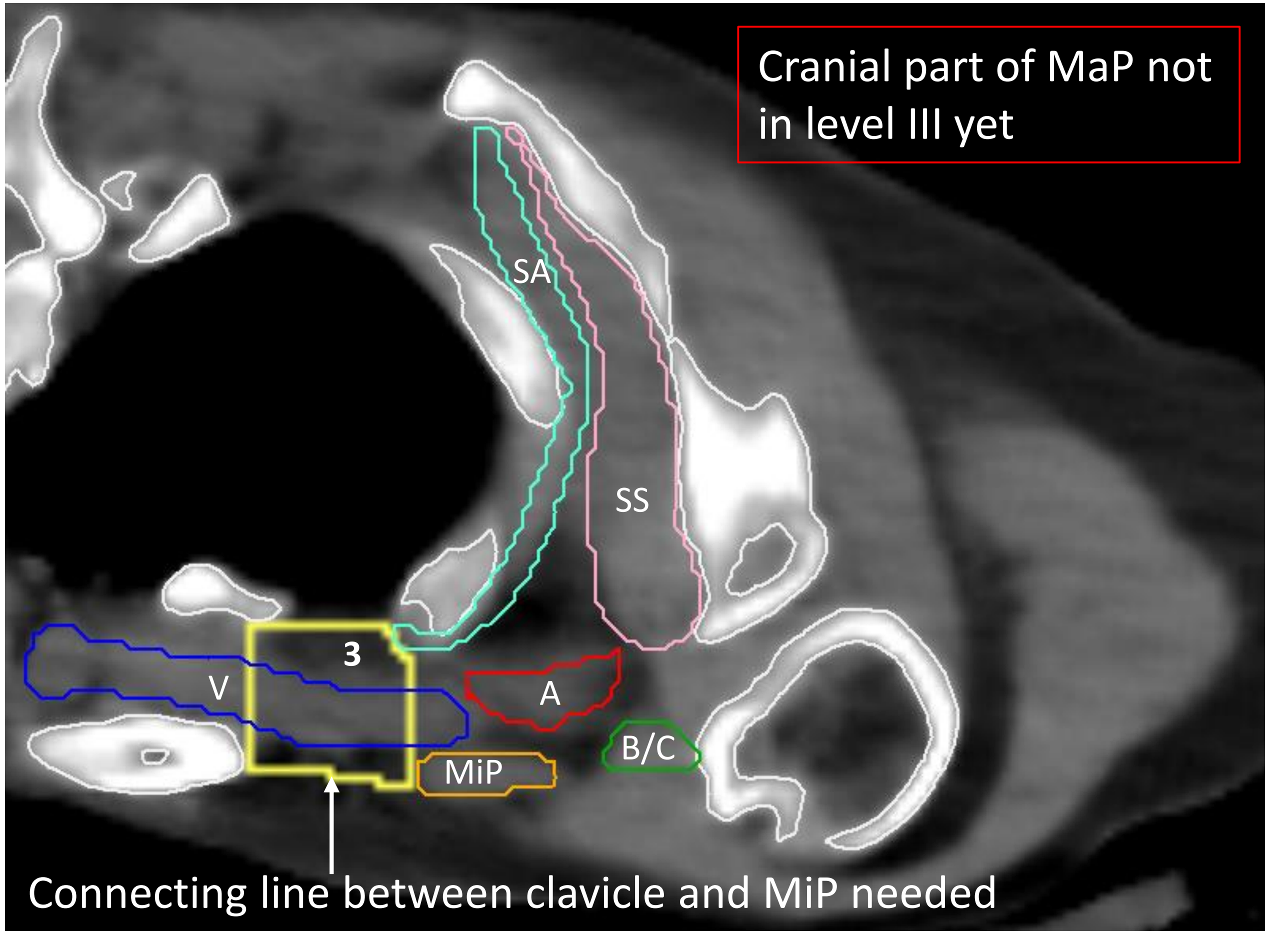

Level III ventral border (excluding subclavius muscle)

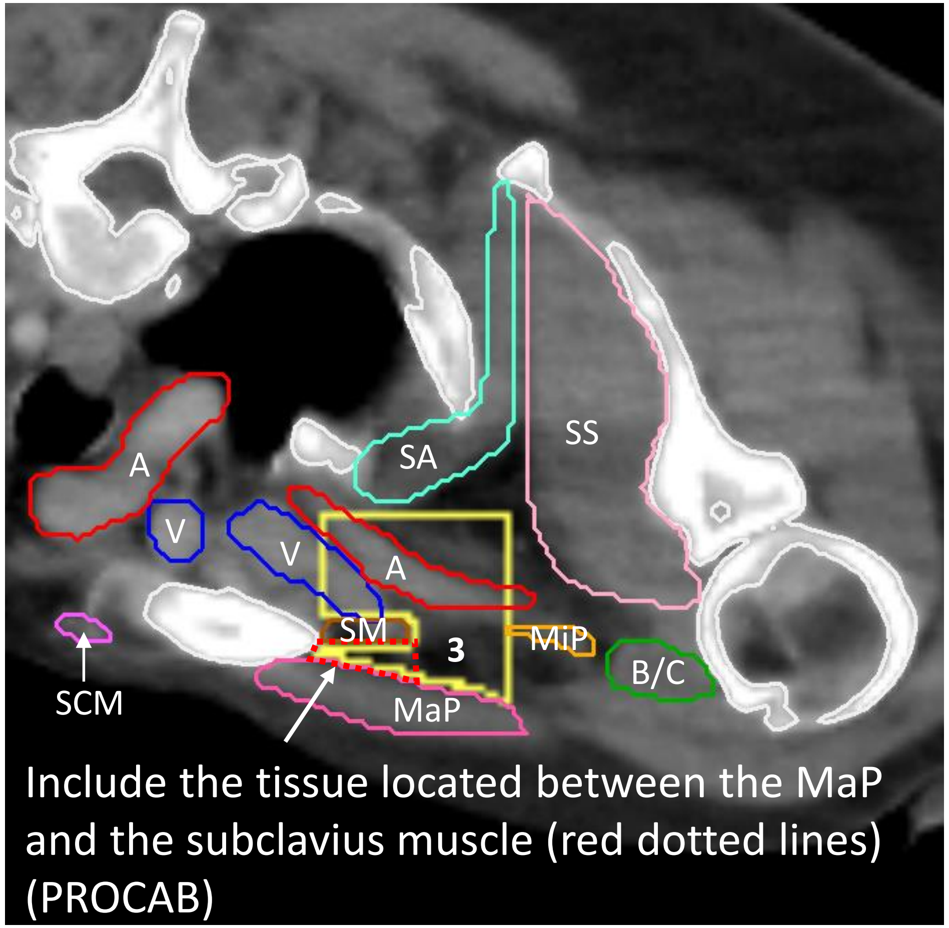

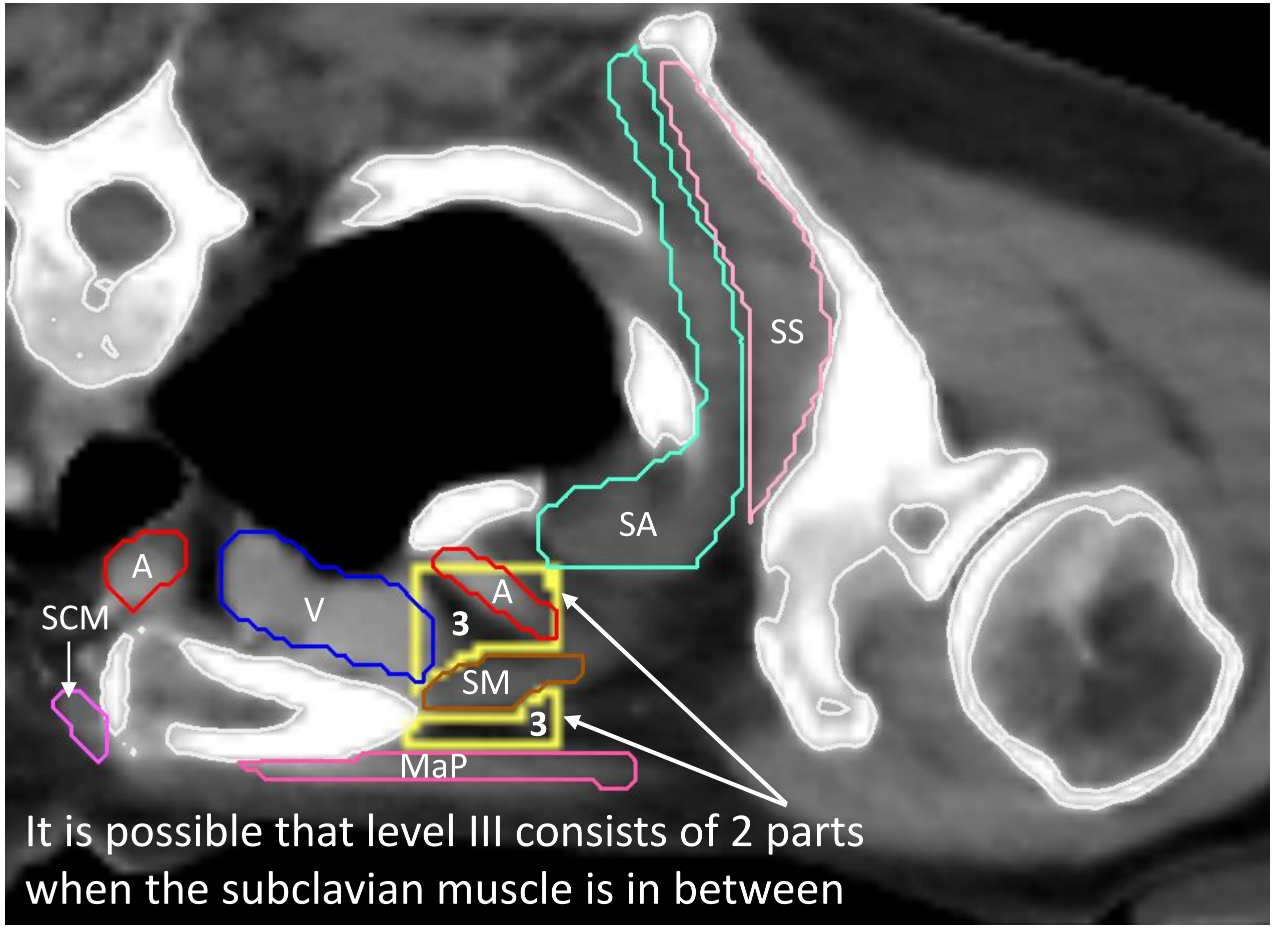

Level III dorsal border

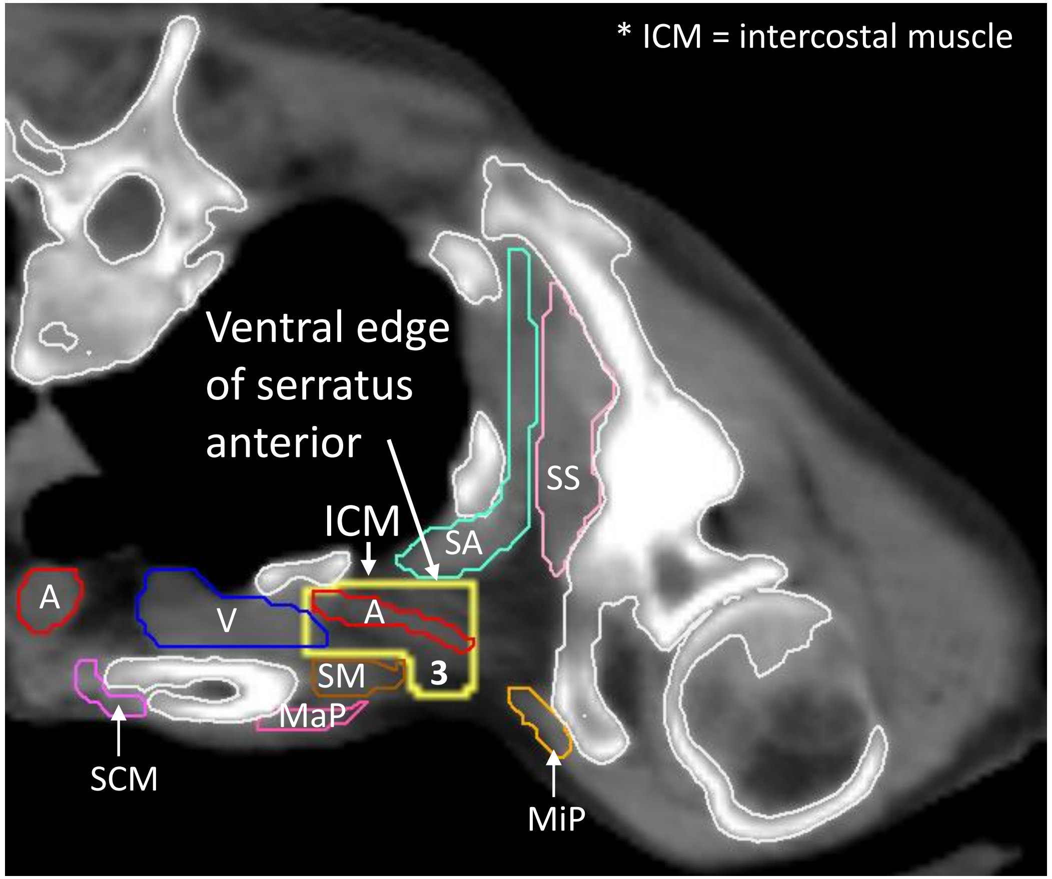

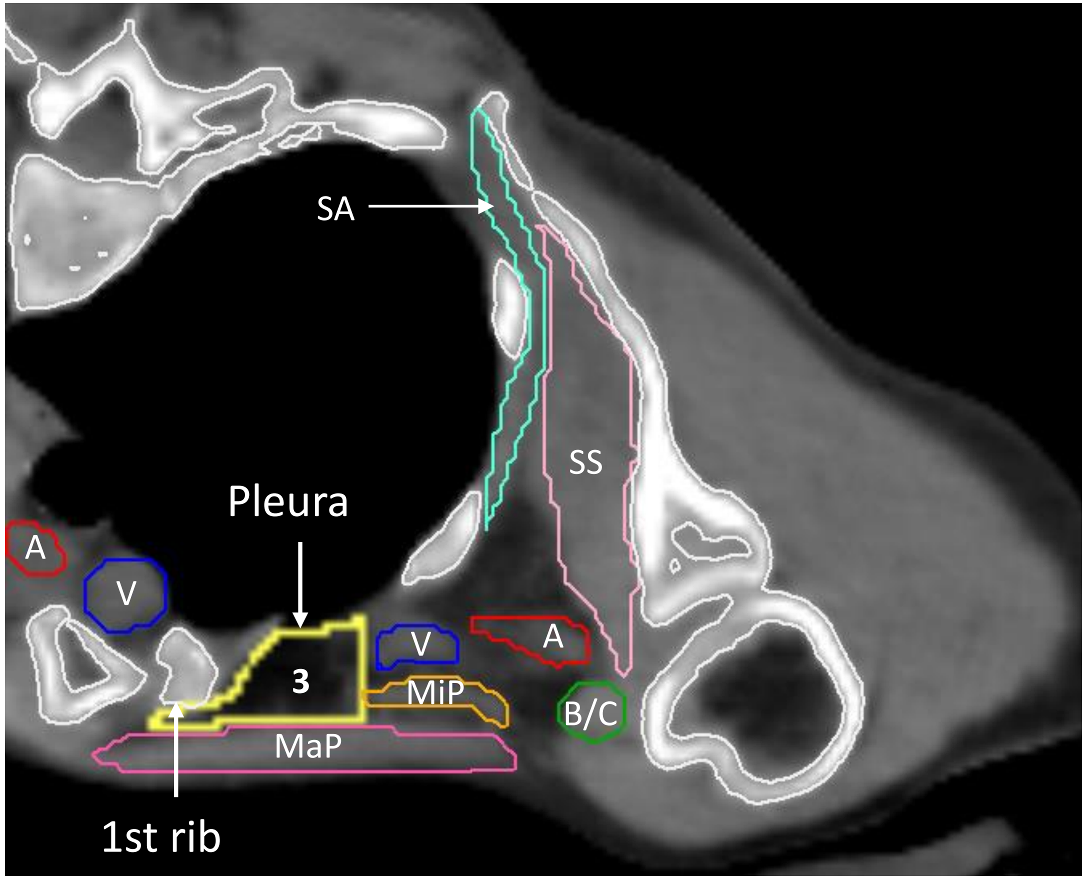

Level III dorsal (5 mm dorsal margin)

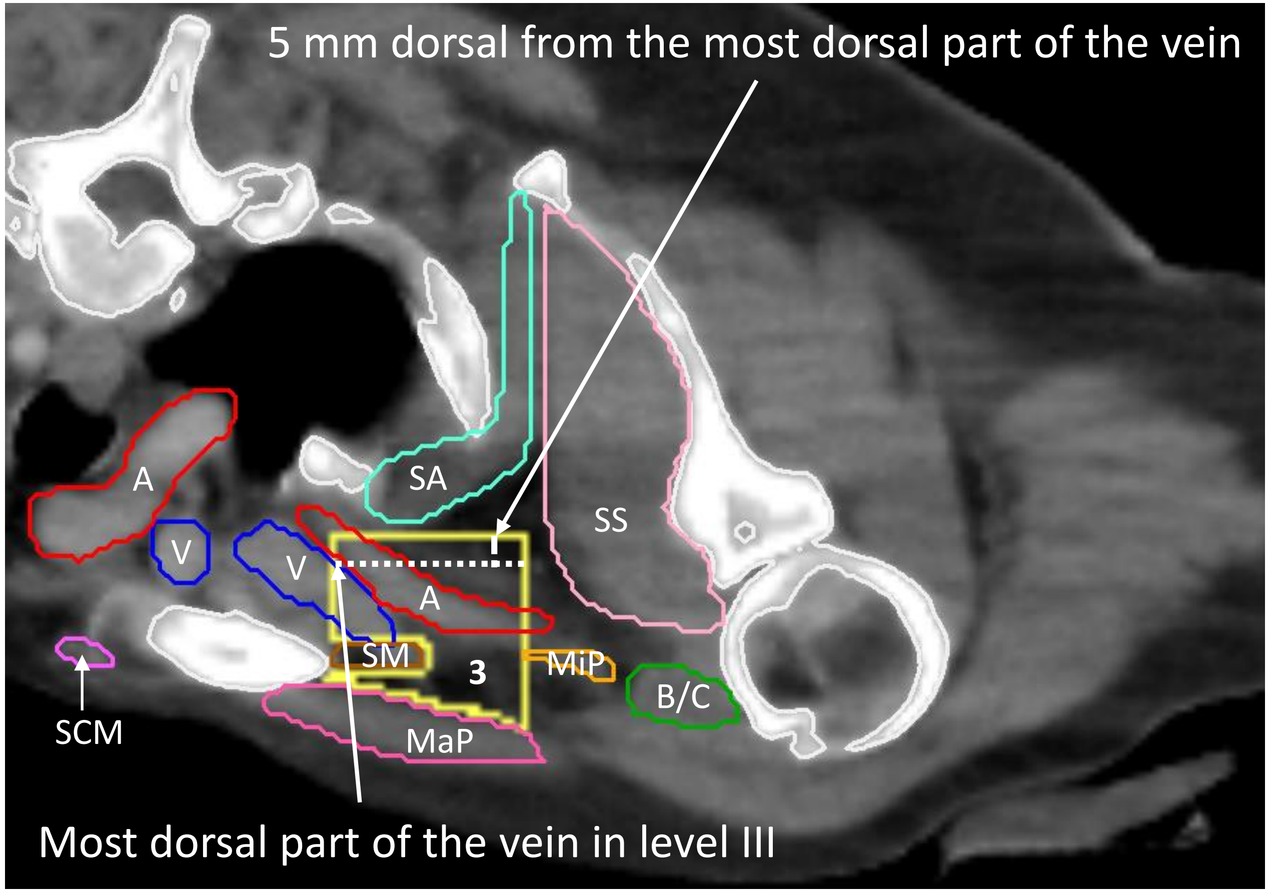

To determine the 5 mm dorsal margin from the vein, find the most dorsal point of the vein within level III.

Measure 5 mm in dorsal direction from this point and connect the medial and lateral border at this level.

Exclude the ribs, the serratus anterior muscle, the intercostal muscles and the pleura if they are located within the 5 mm margin
(not the case in this example, the serratus anterior muscle is not reached)

Level III dorsal border (5 mm margin)

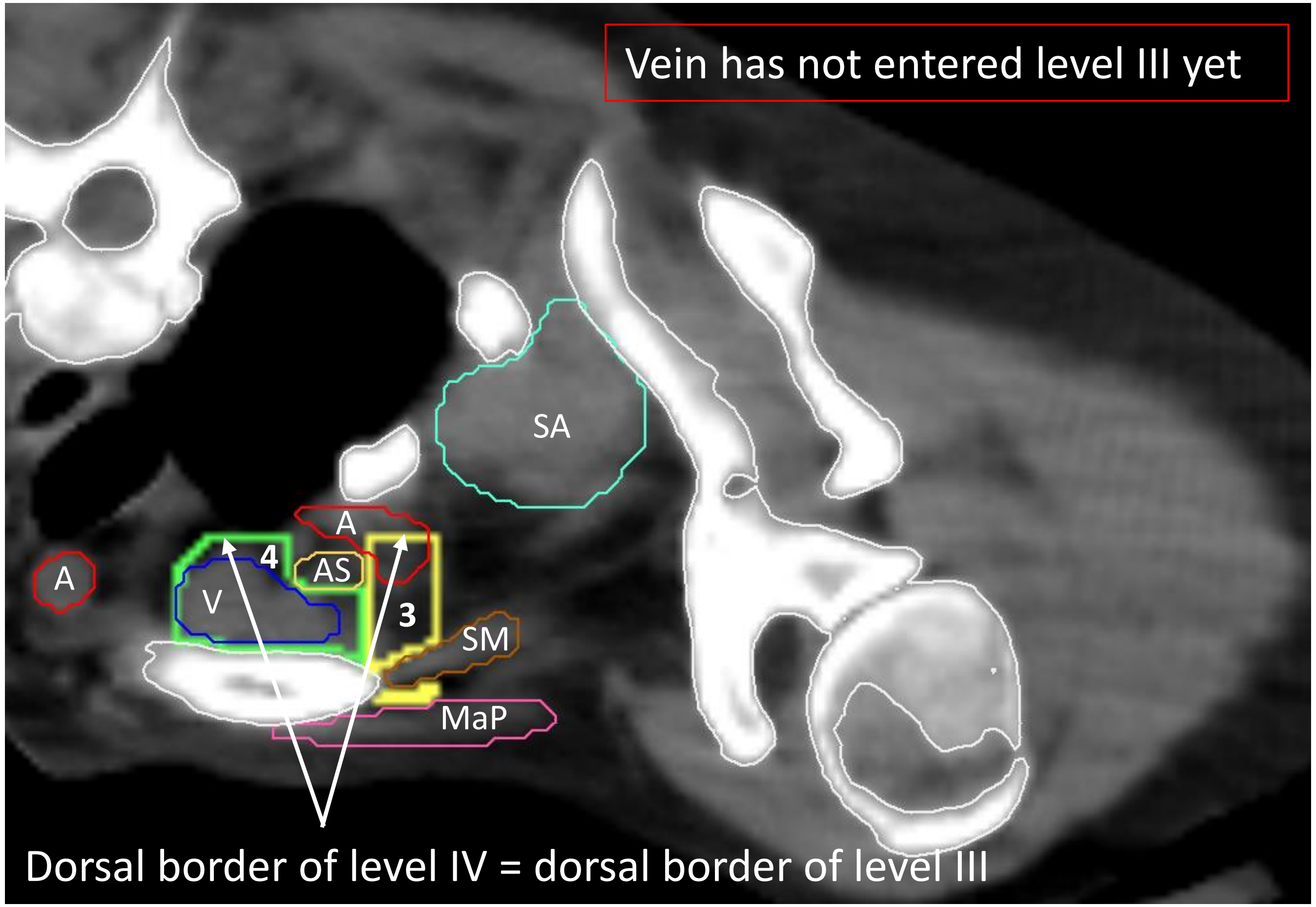

In the most cranial slices, where the vein has sometimes not entered level III yet, the 5 mm dorsal margin from the most dorsal part of the vein can not be taken from inside level III

In this case, refer to the dorsal border of level IV, that marks 5 mm dorsal from the dorsal edge of the vein in level IV.

Level II cranial border

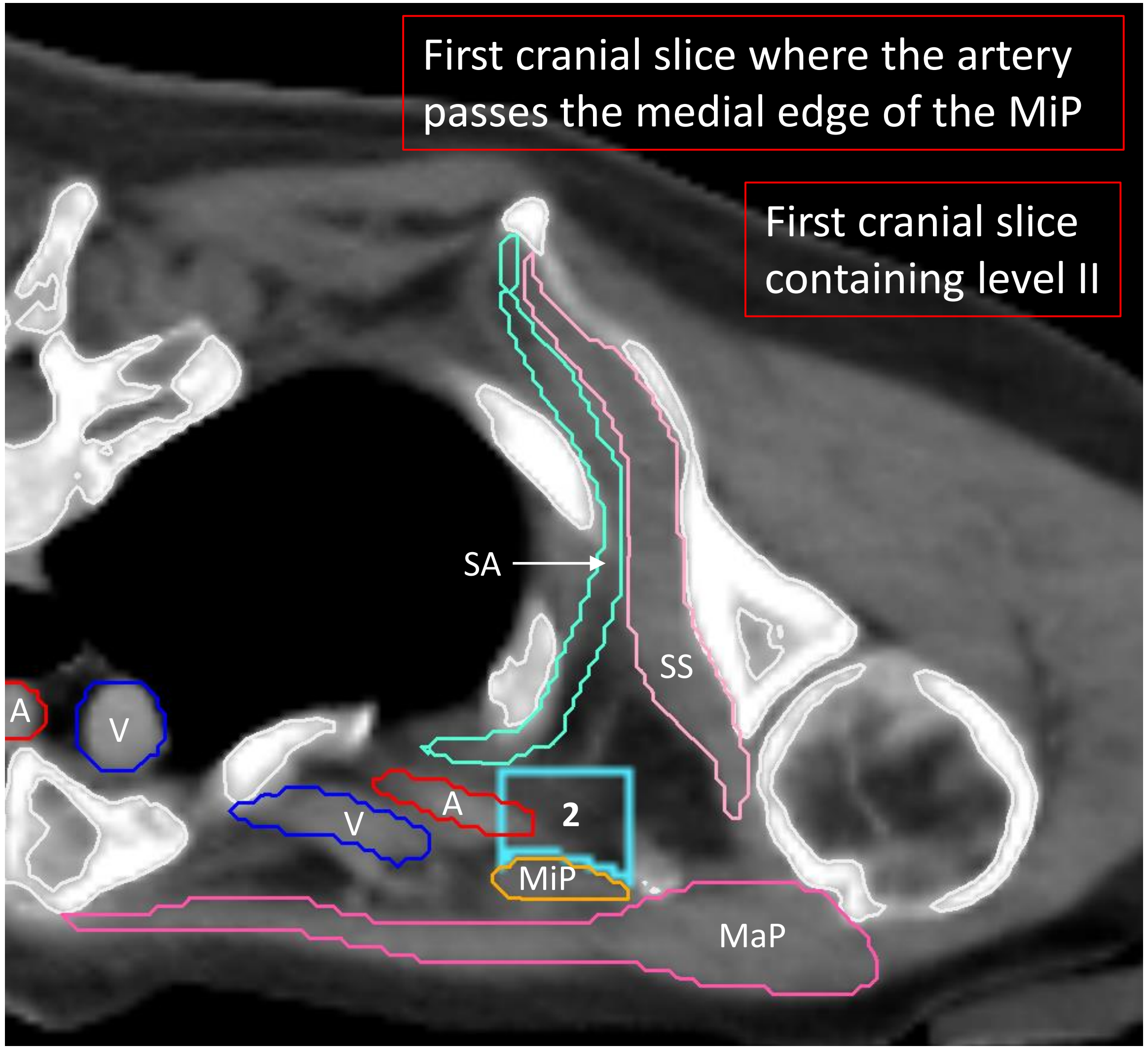

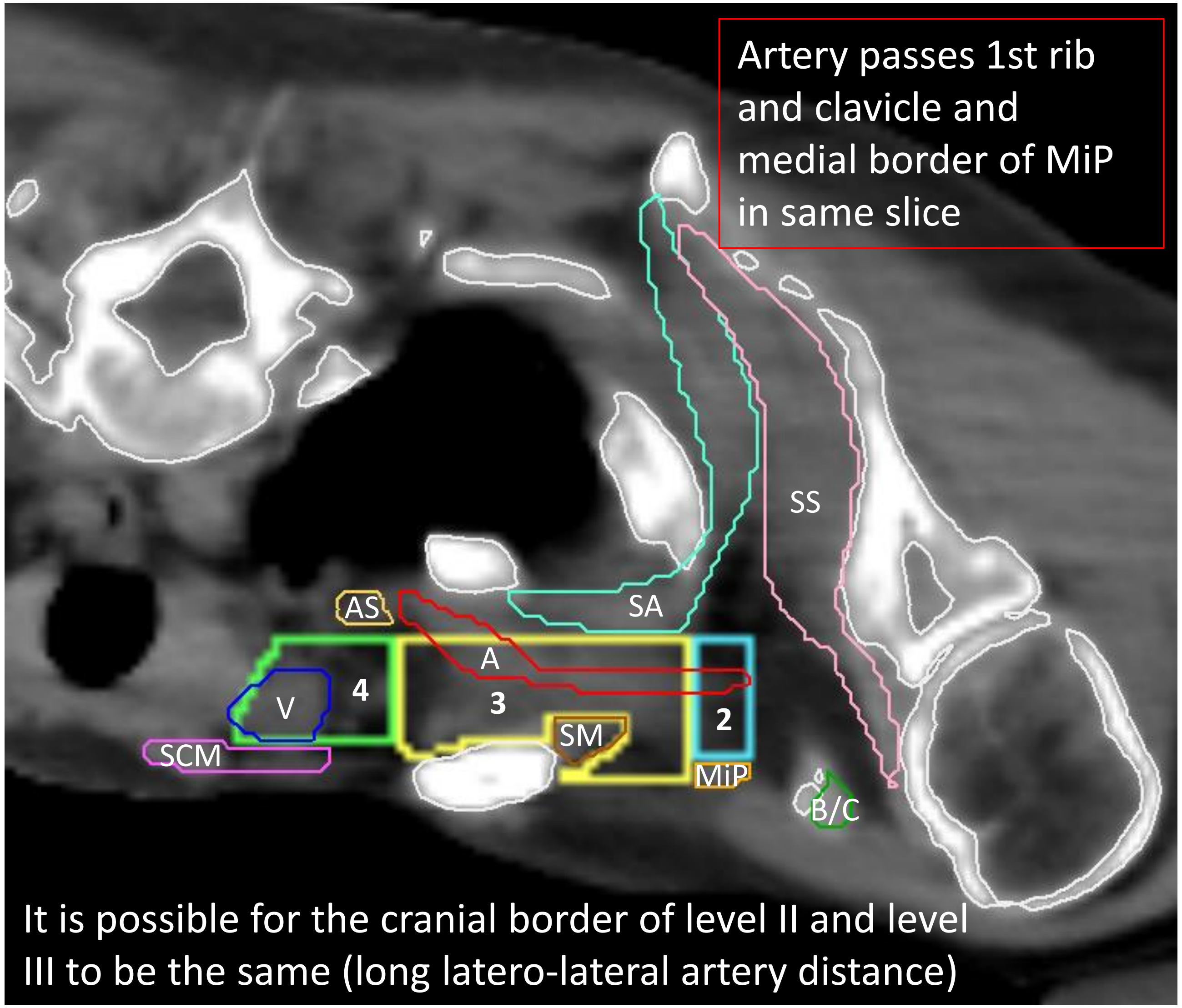

Level II caudal border

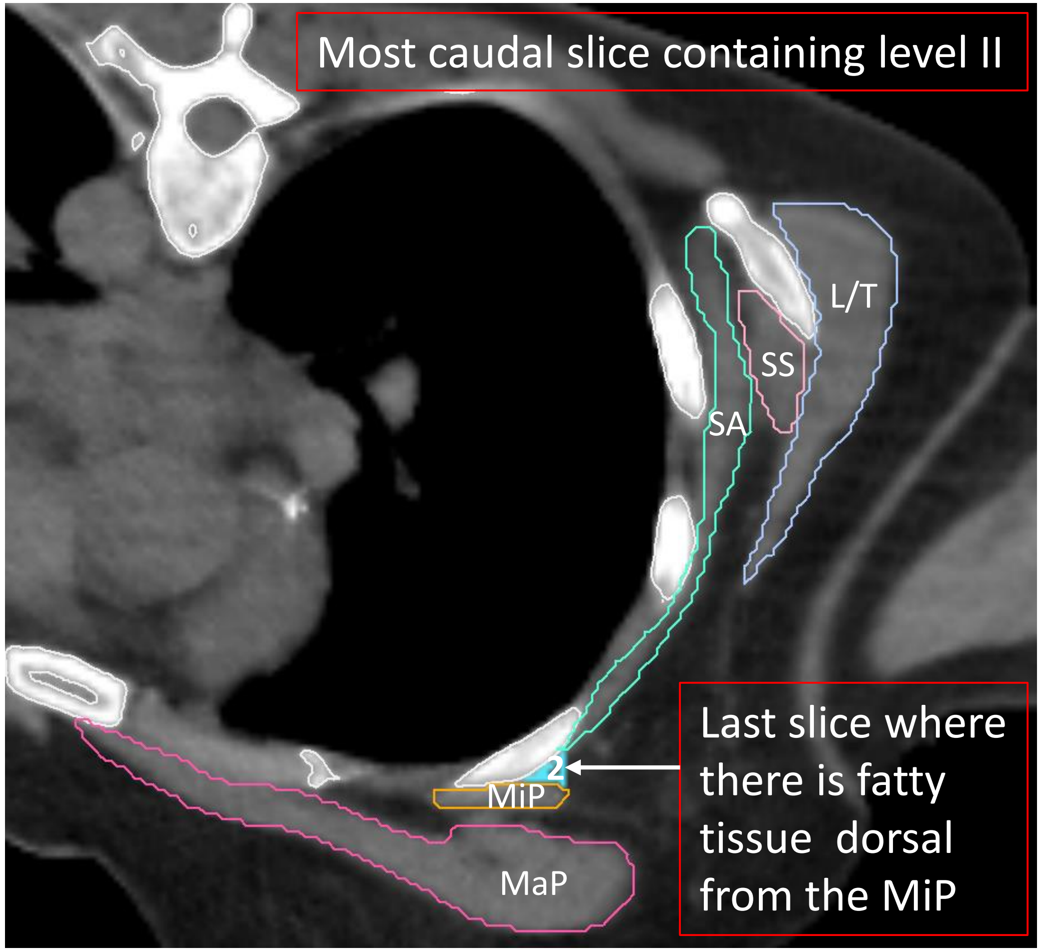

Level II medial border

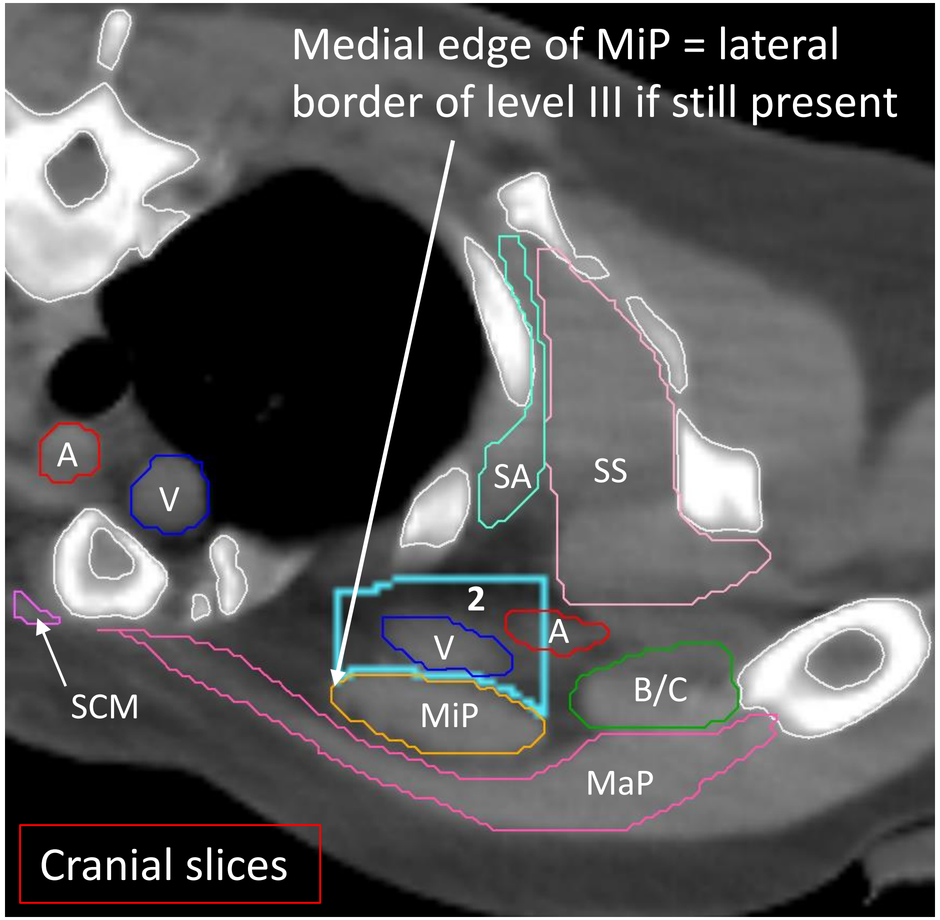

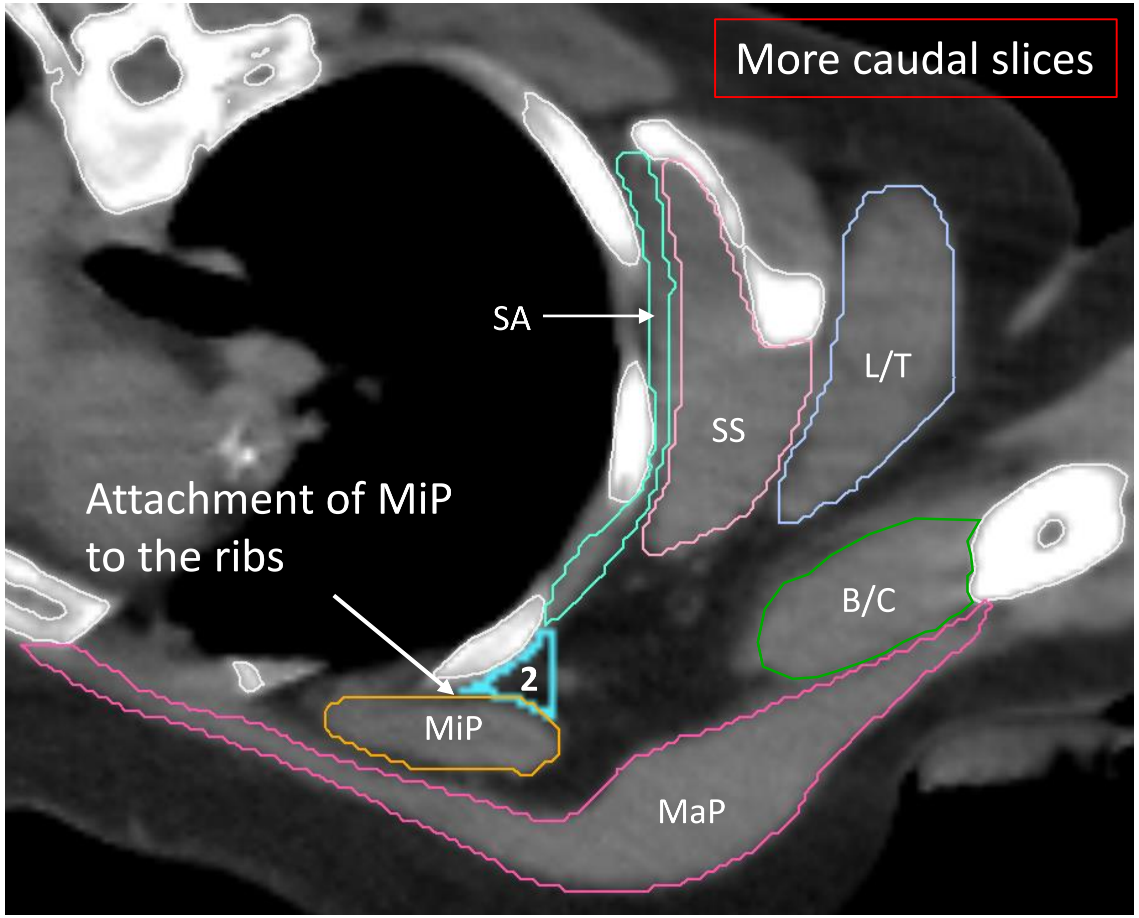

Level II lateral border

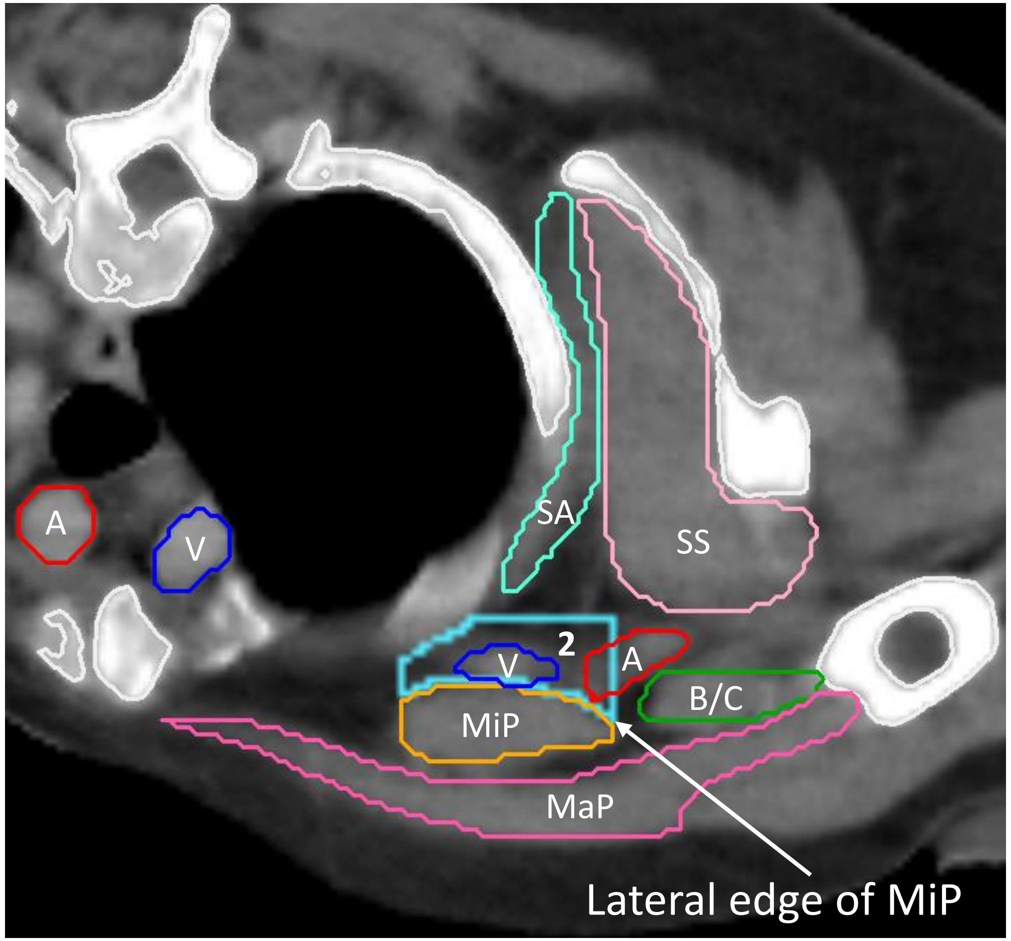

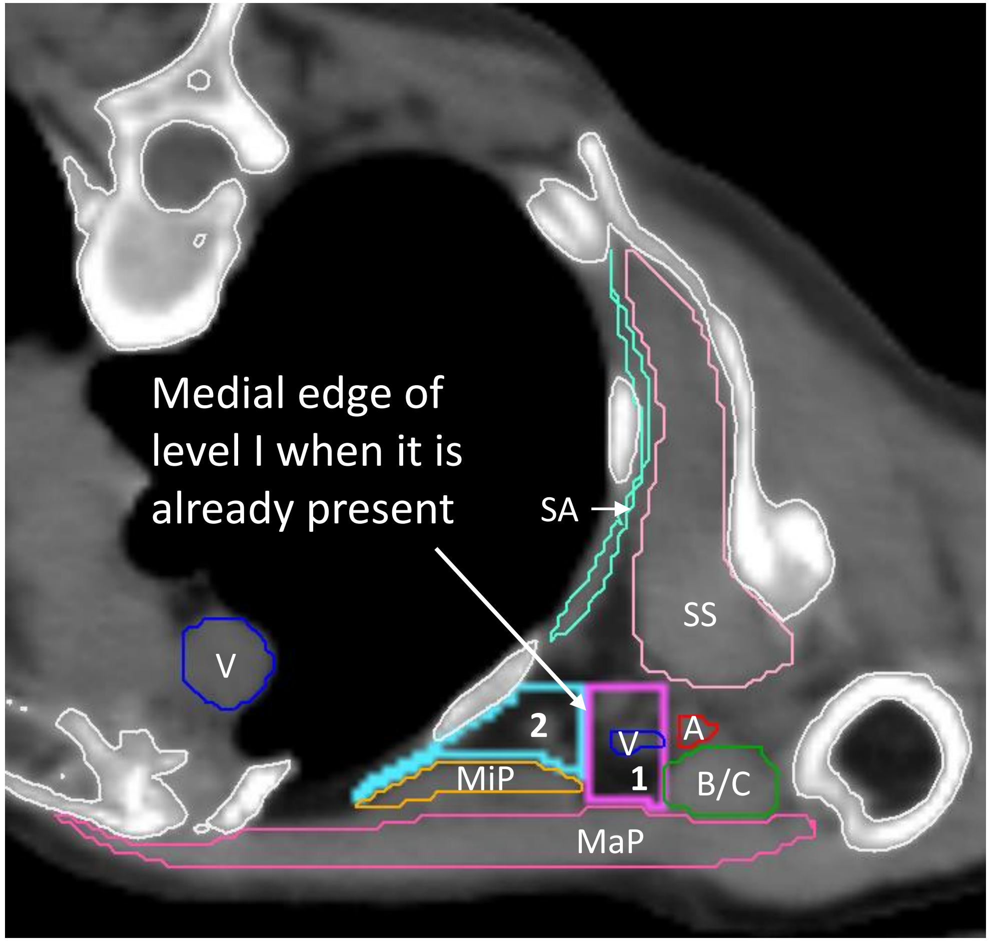

Level II ventral border

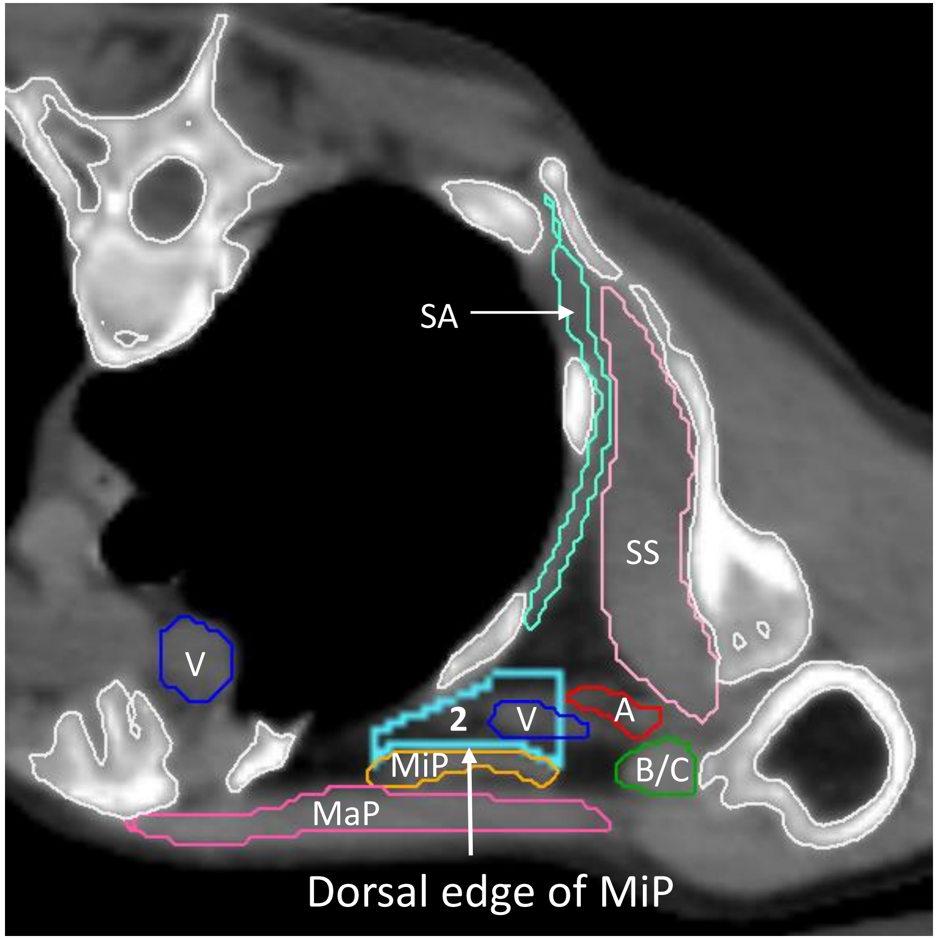

Level II dorsal border

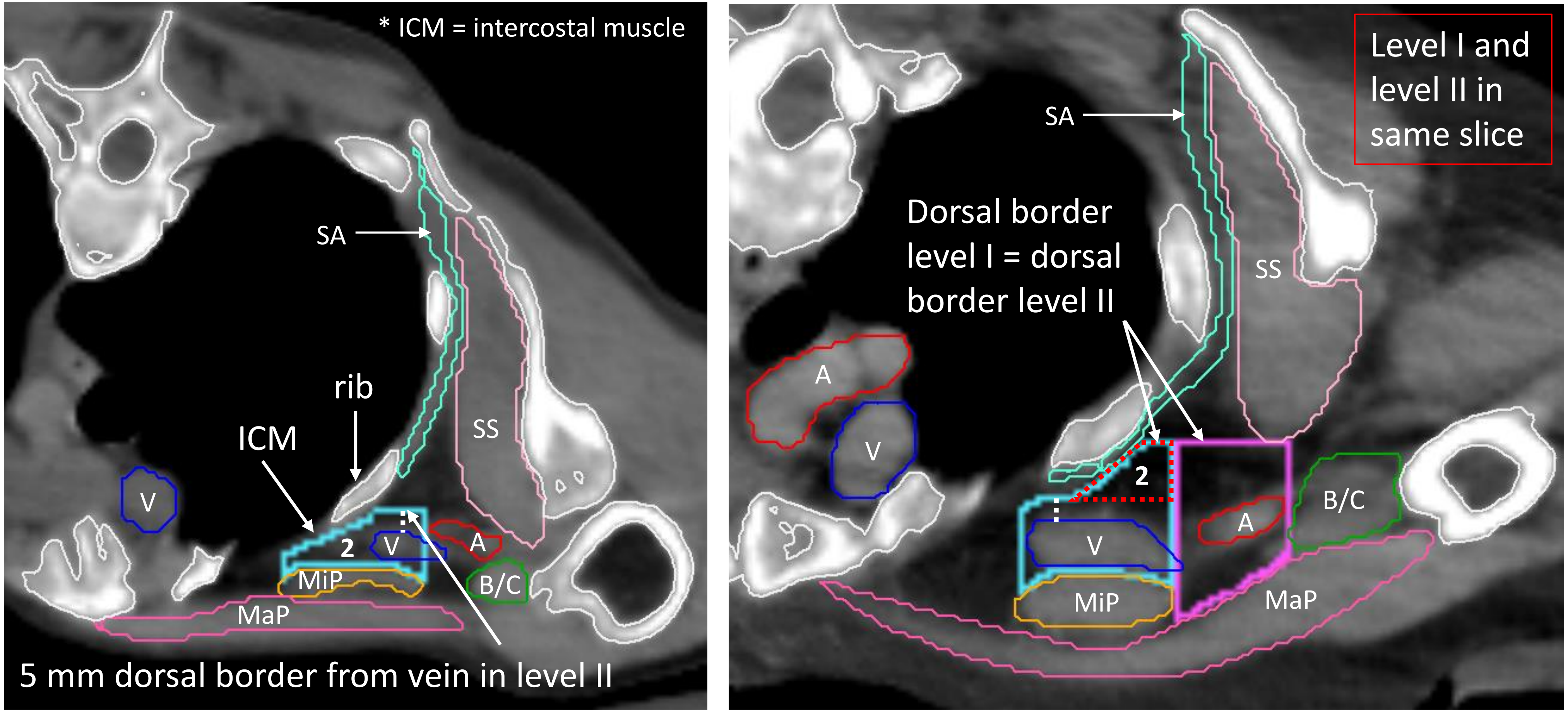

Dorsal border follows dorsal border of level I, once level I starts.

This makes sure that level I and II connect smoothly and there is no gap in the target volume between level I, level II and the ribs (red dotted lines).

Level I cranial border

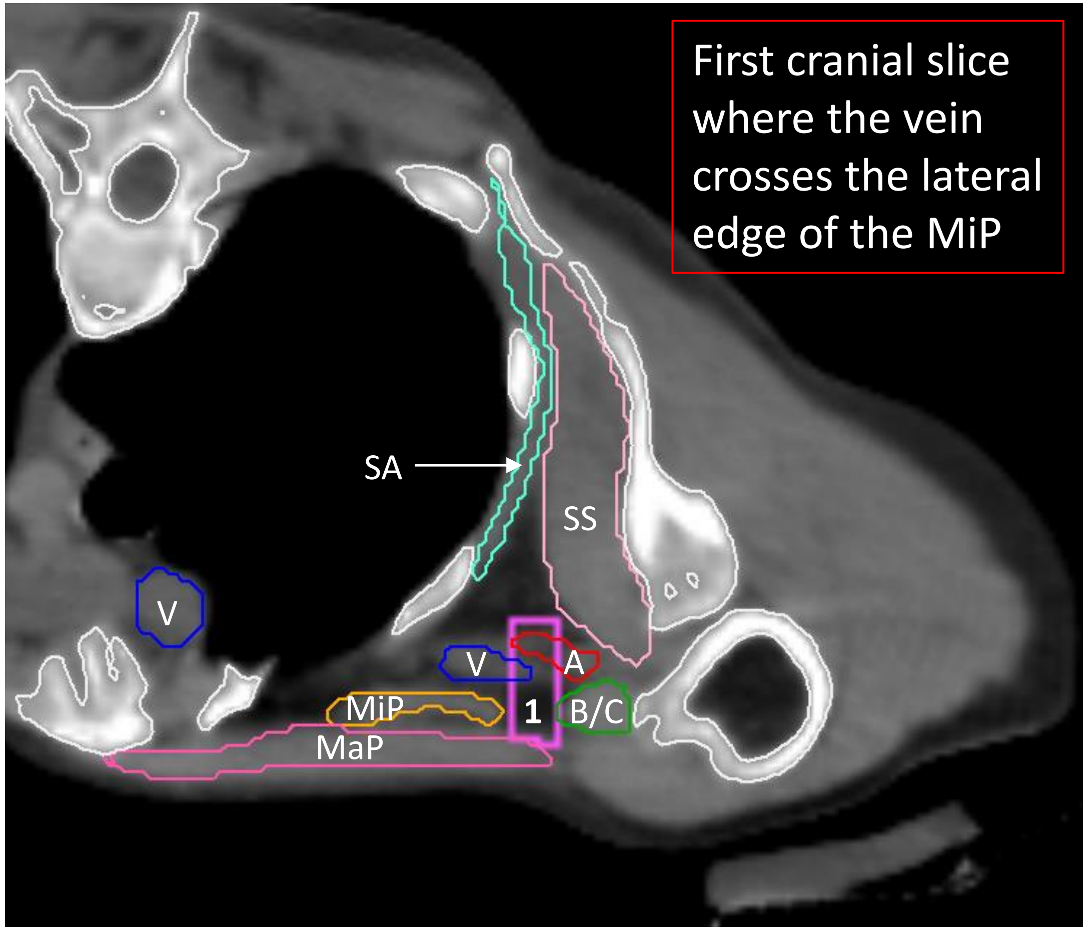

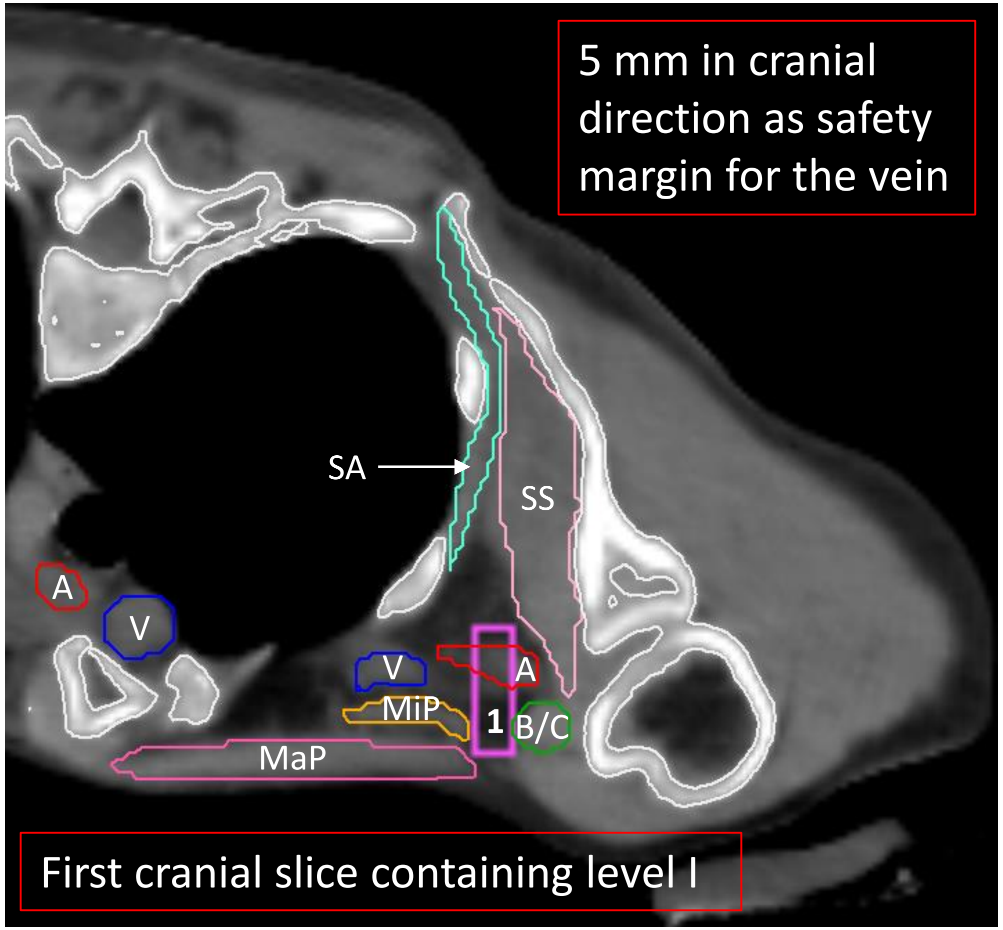

Level I caudal border

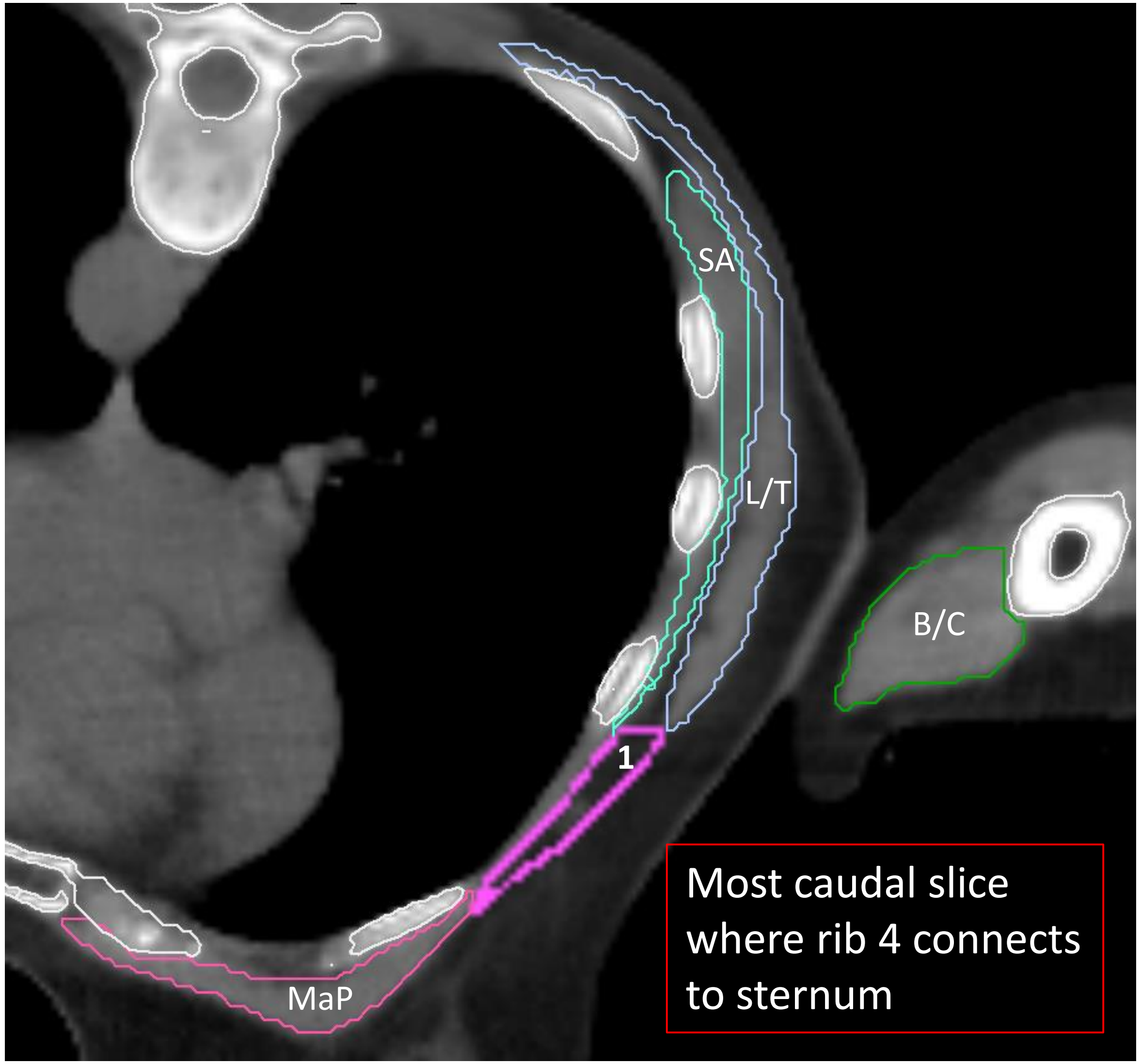

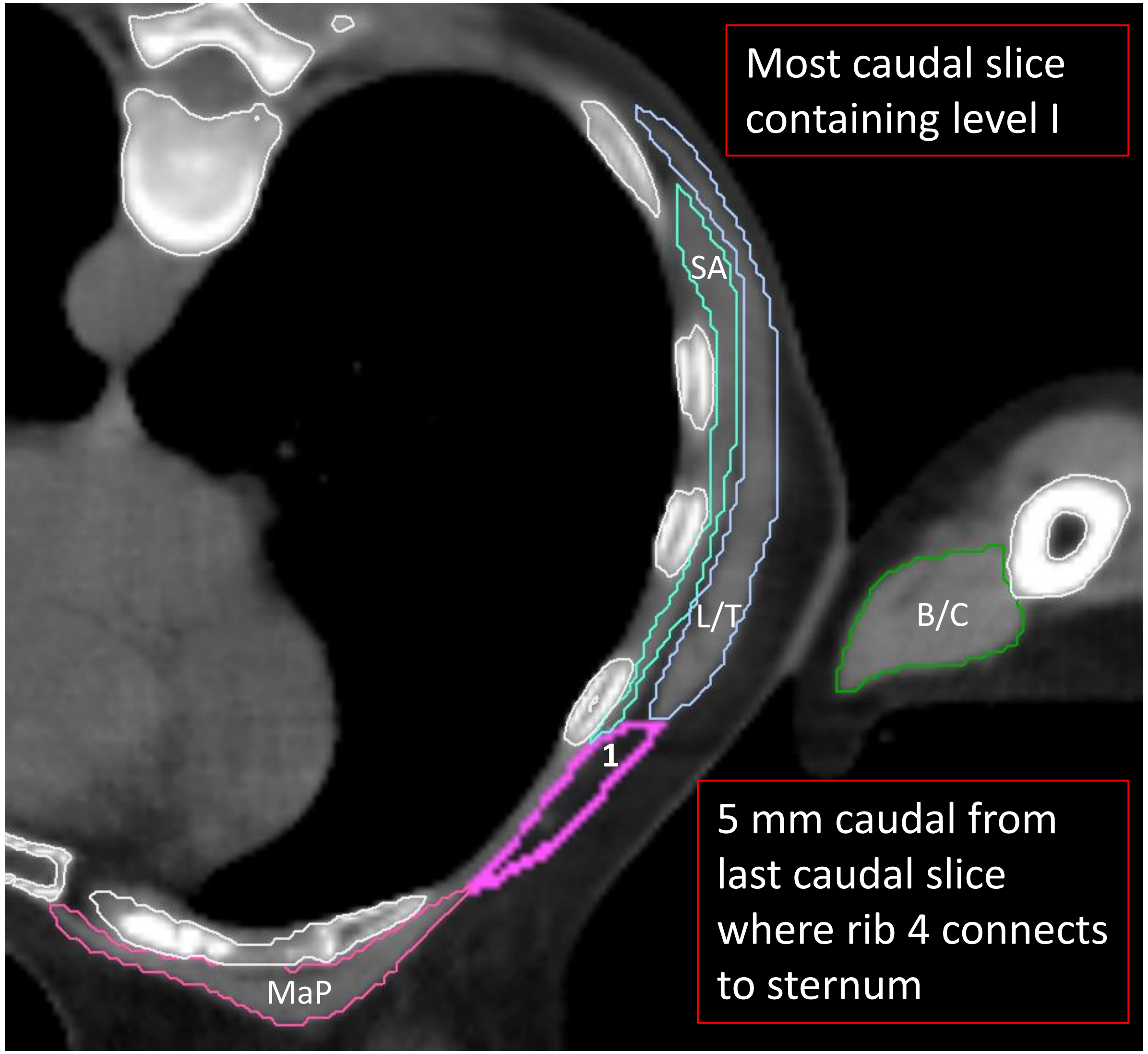

Level I (dorso)medial border

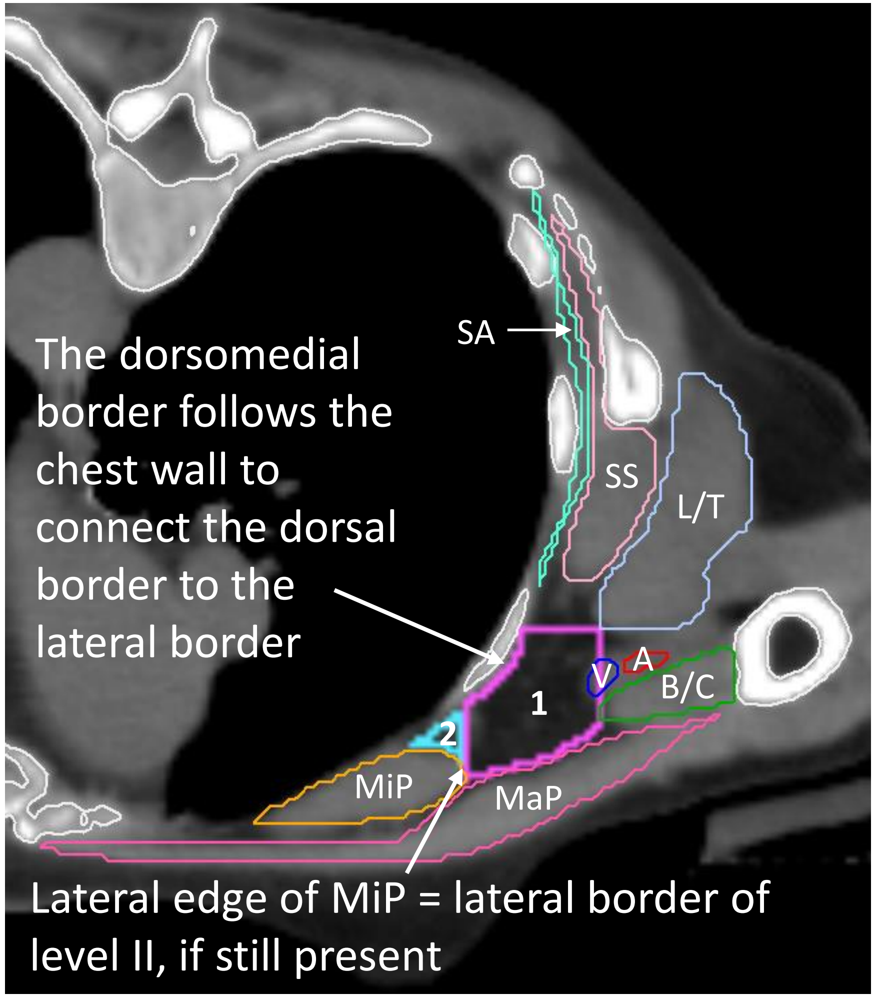

Level I lateral border

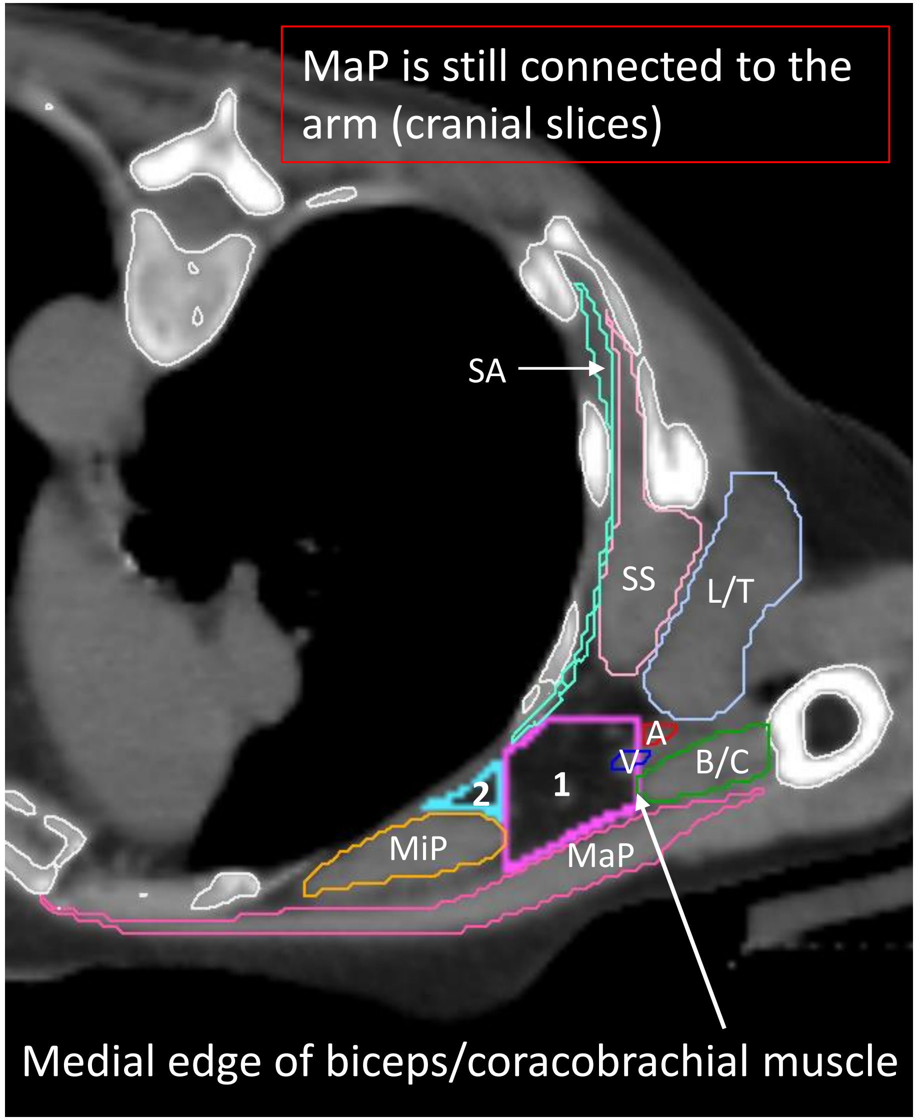

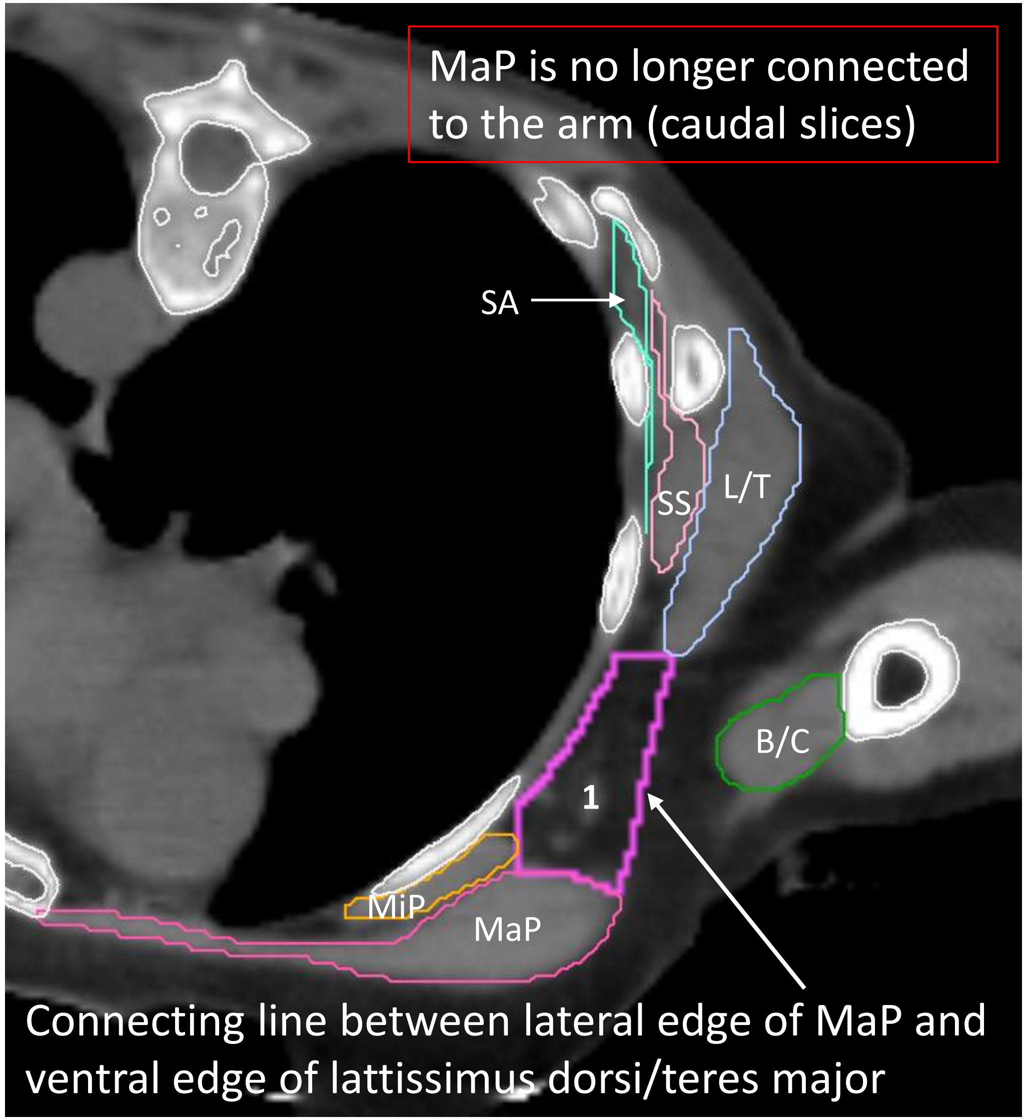

Level I ventral border

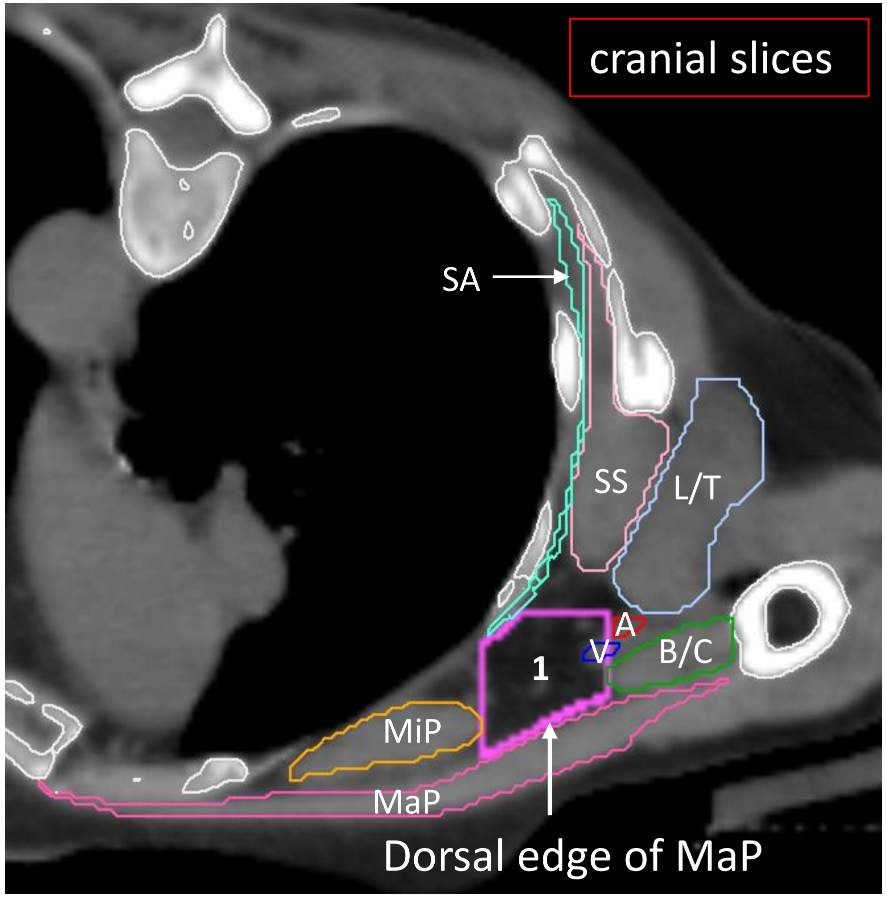

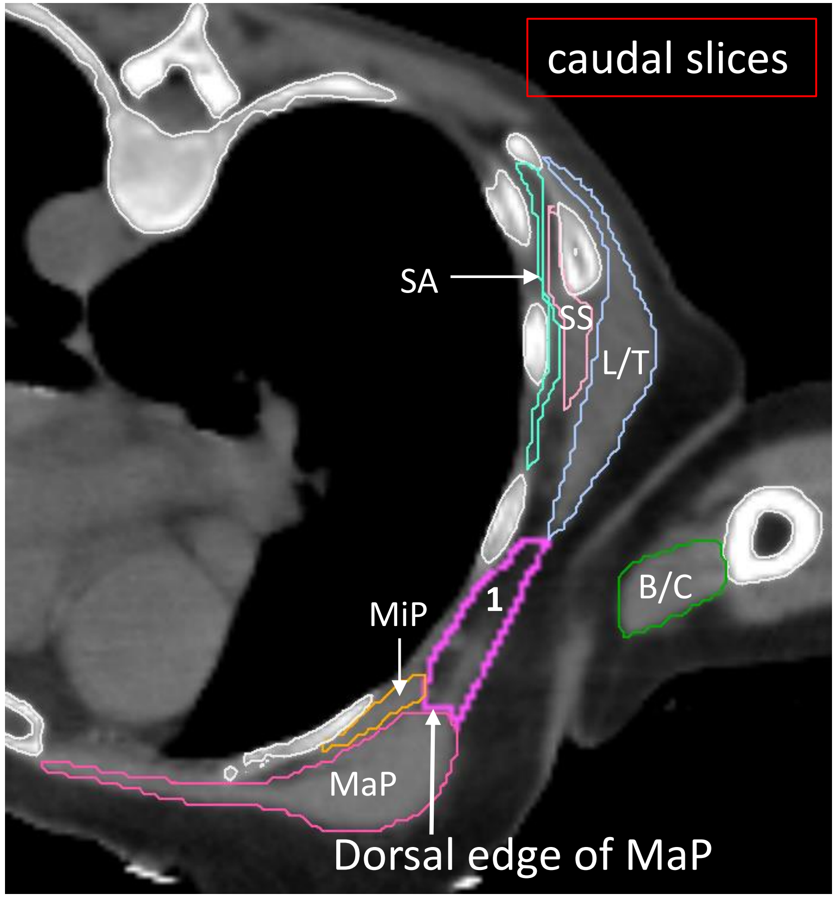

Level I dorsal border (subscapular muscle)

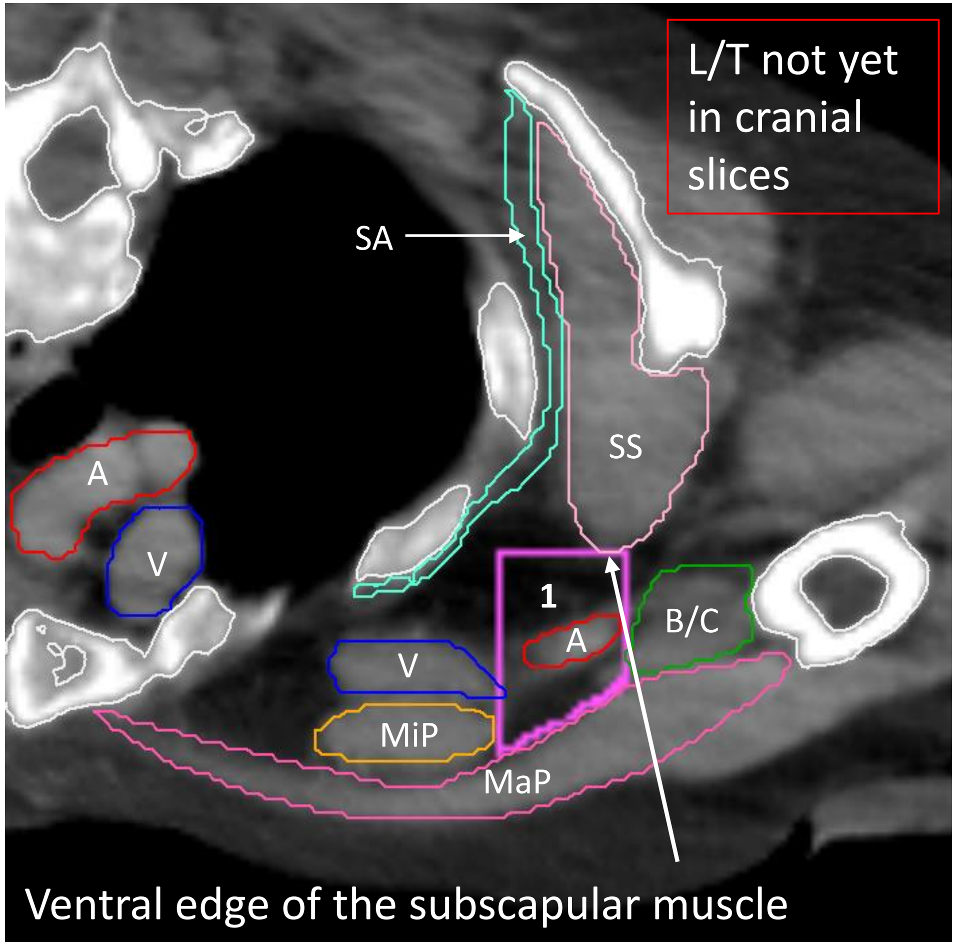

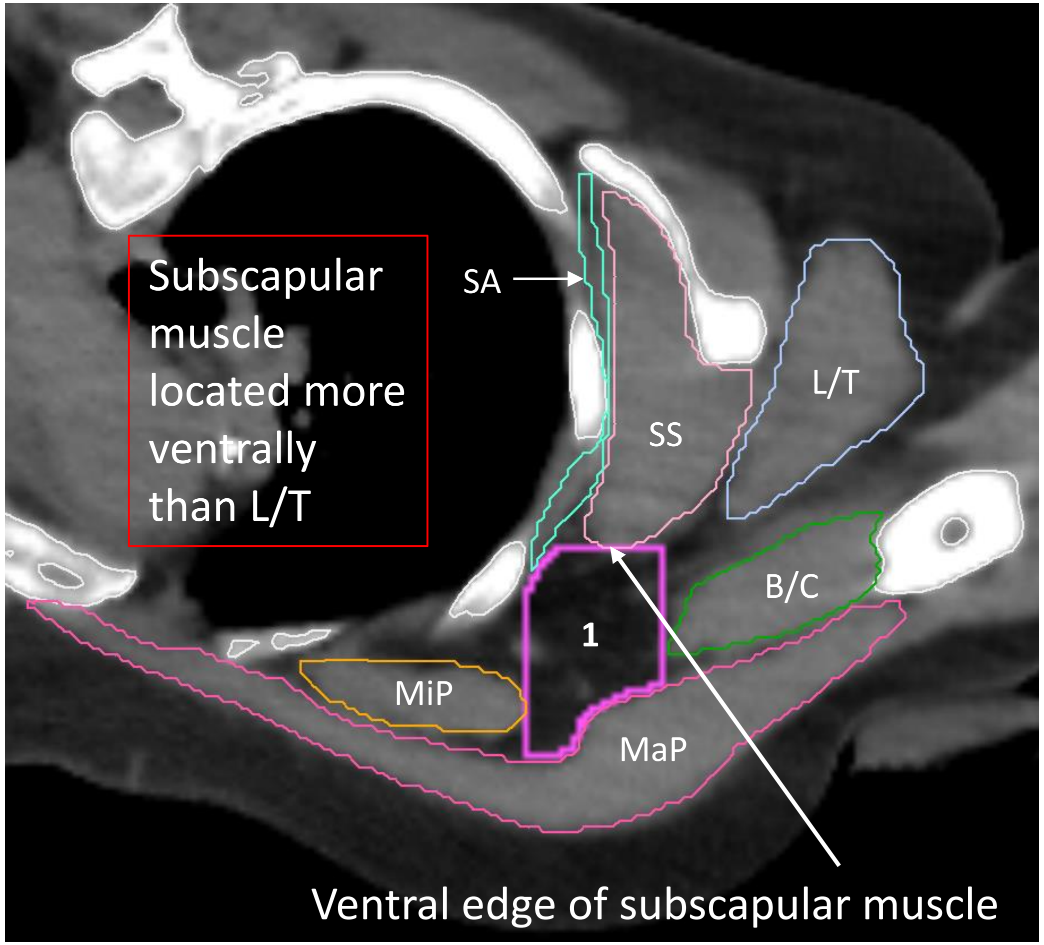

Level I dorsal border (lattissimus dorsi)

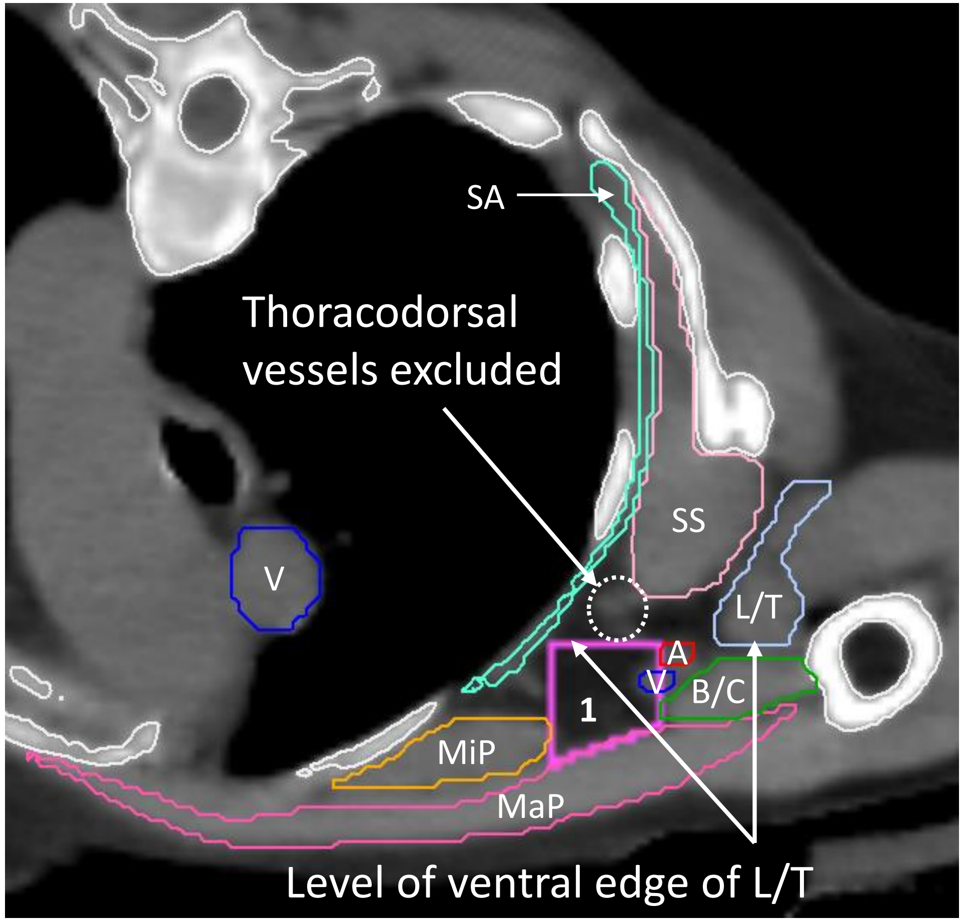

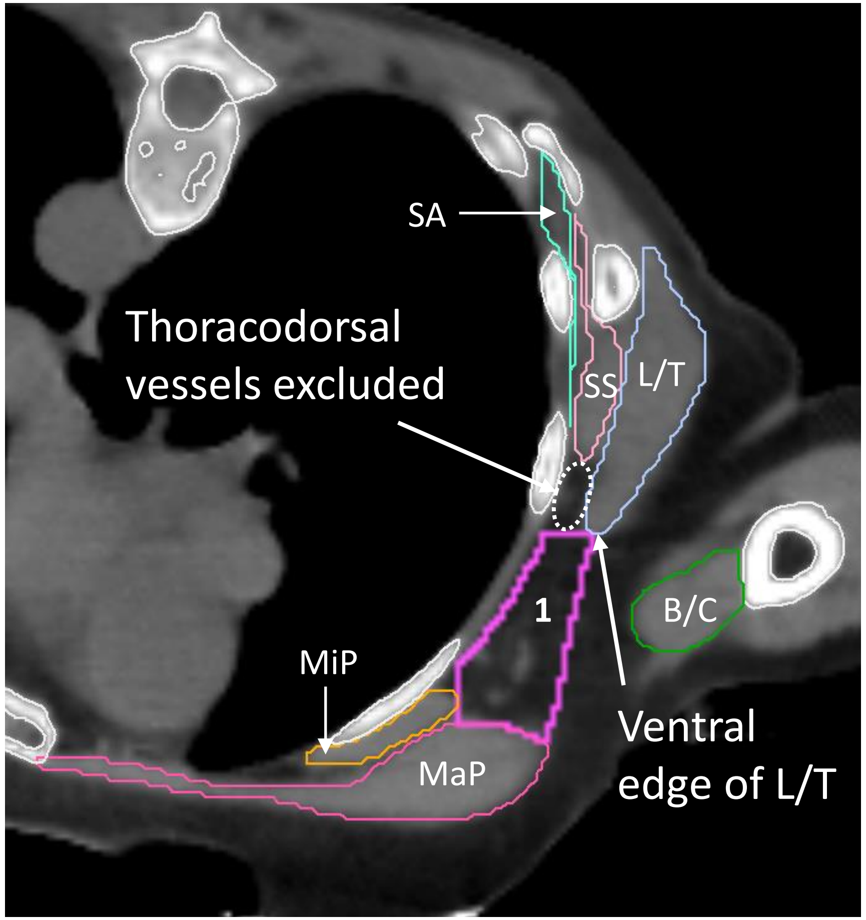

In the majority of cases, the lattissimus dorsi/ teres major muscle bundle will be located more ventrally than the subscapular muscle and it will be used as dorsal edge of level I

Level I (thoracodorsal vessels)

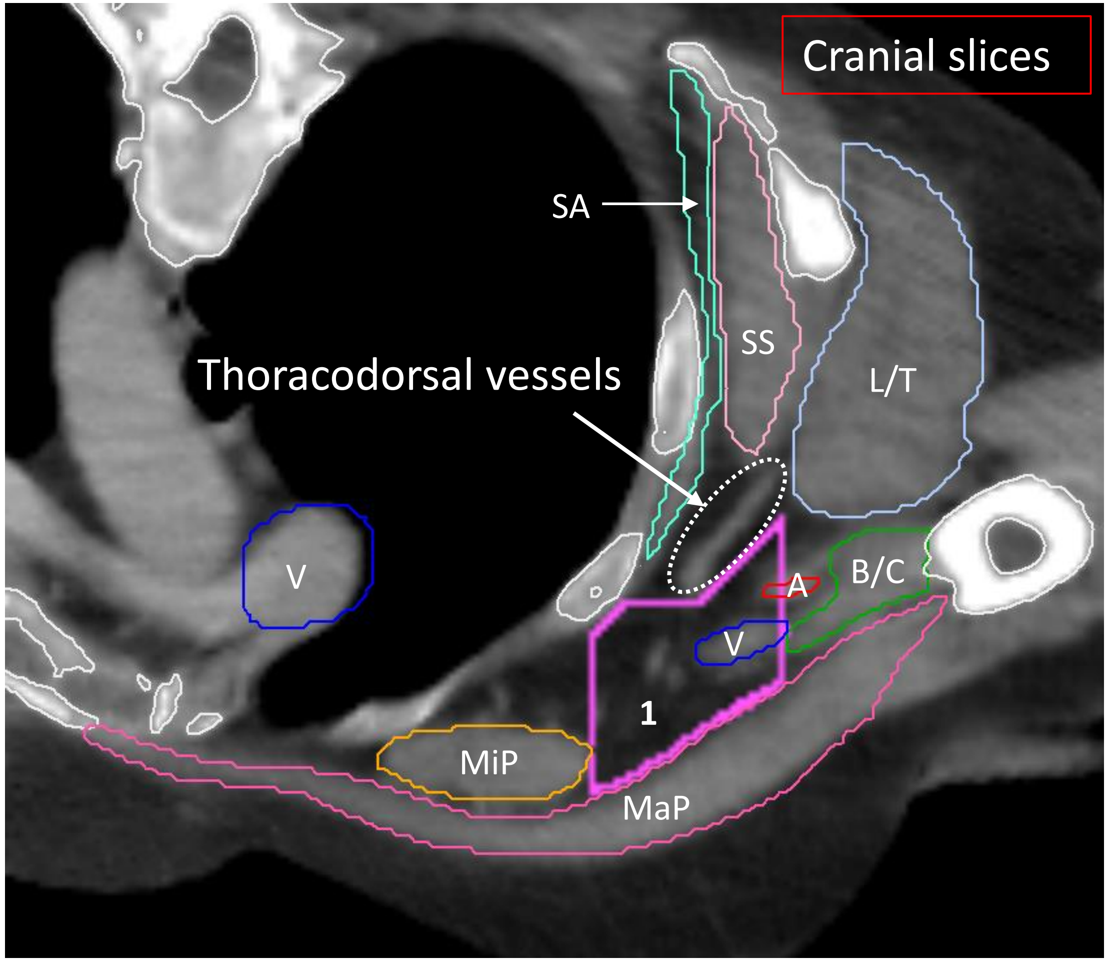

When the thoracodorsal vessels branch off from the axillary vessels (in the more cranial slices of level I) they can occasionally be included in level I, instead of being located dorsally from this level.

When the thoracodorsal vessels are included in level I, the dorsolateral border can be adapted to exclude them from the target volume as shown in this example.

Level I (thoracodorsal vessels)

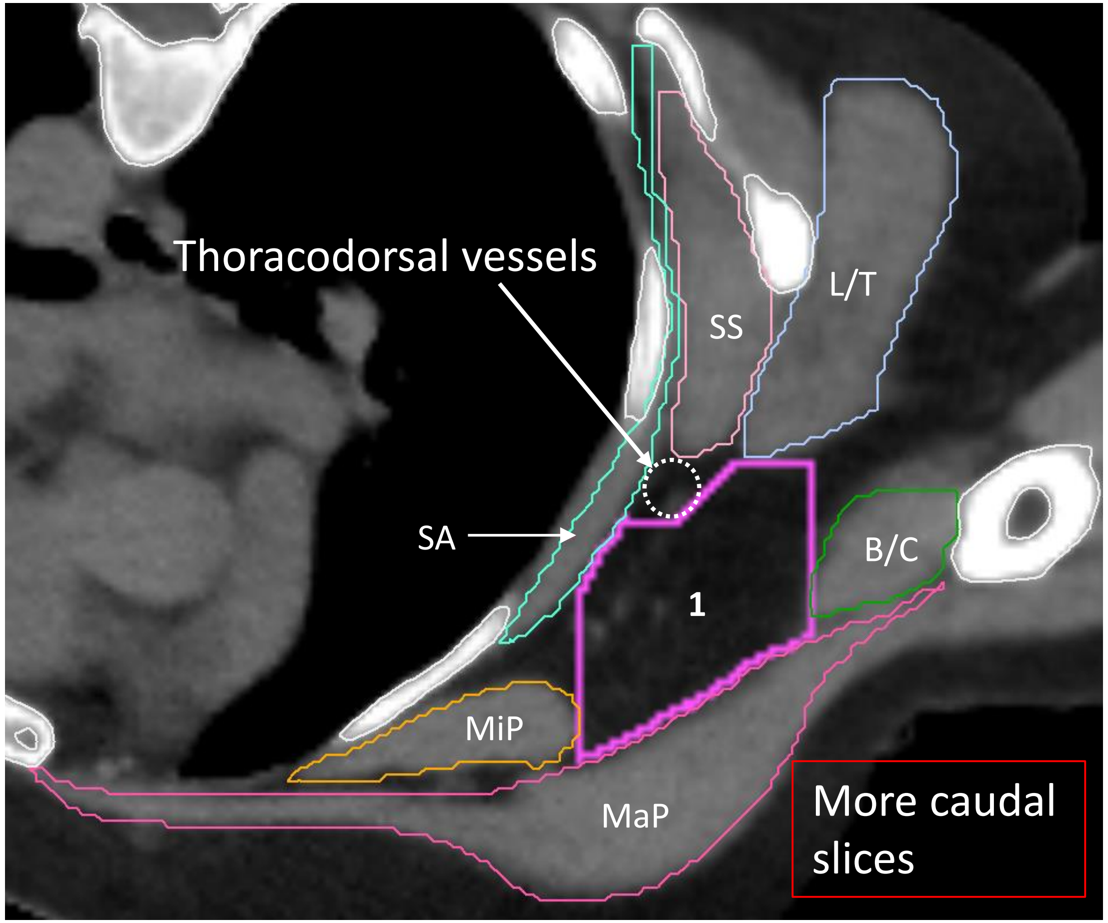

When the ventral edge of the subscapular muscle and the ventral edge of the lattissimus dorsi/teres major muscle bundle are located in close proximity to each other, excluding the thoracodorsal vessels may in rare cases require an adaptation of the dorsolateral border of level I in the more caudal slices, as shown here.

Internal mammary nodes cranial border

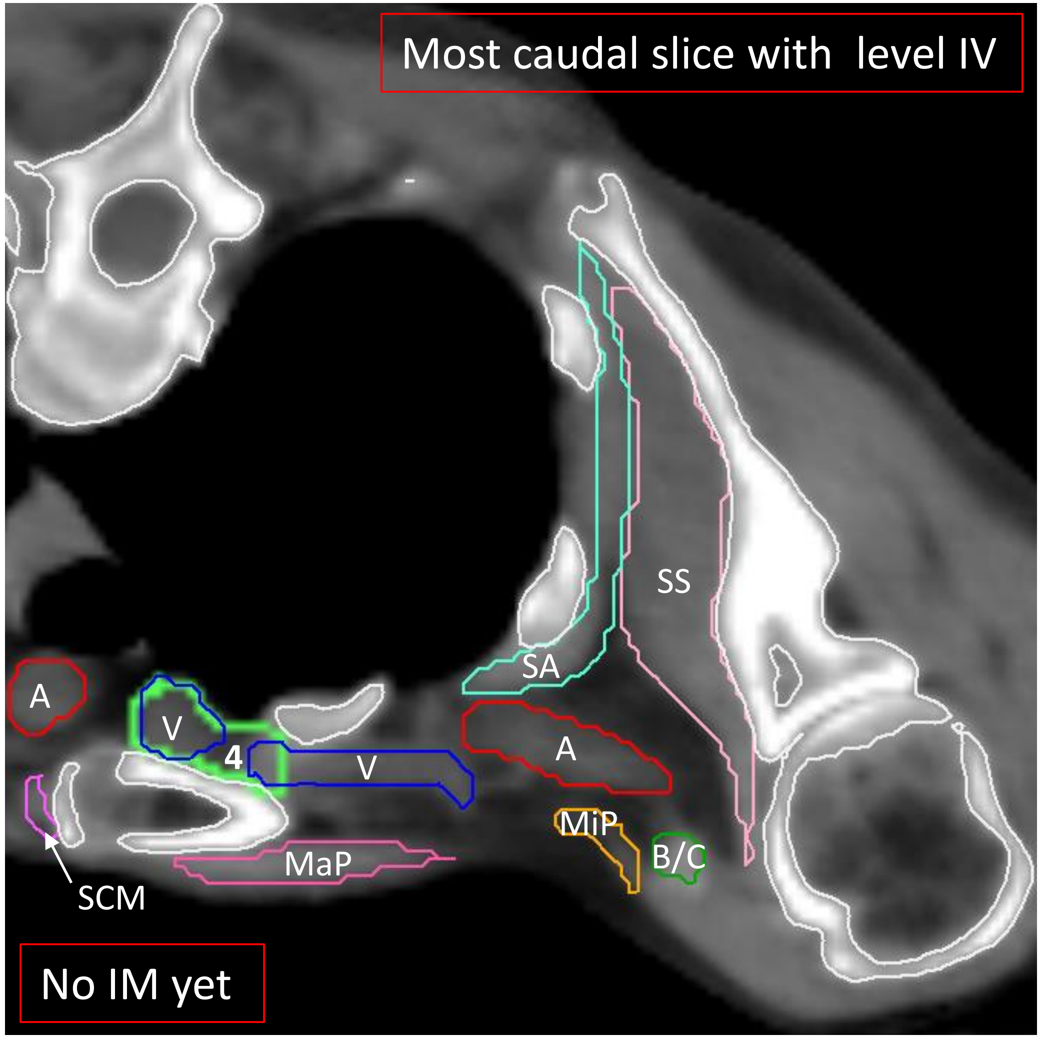

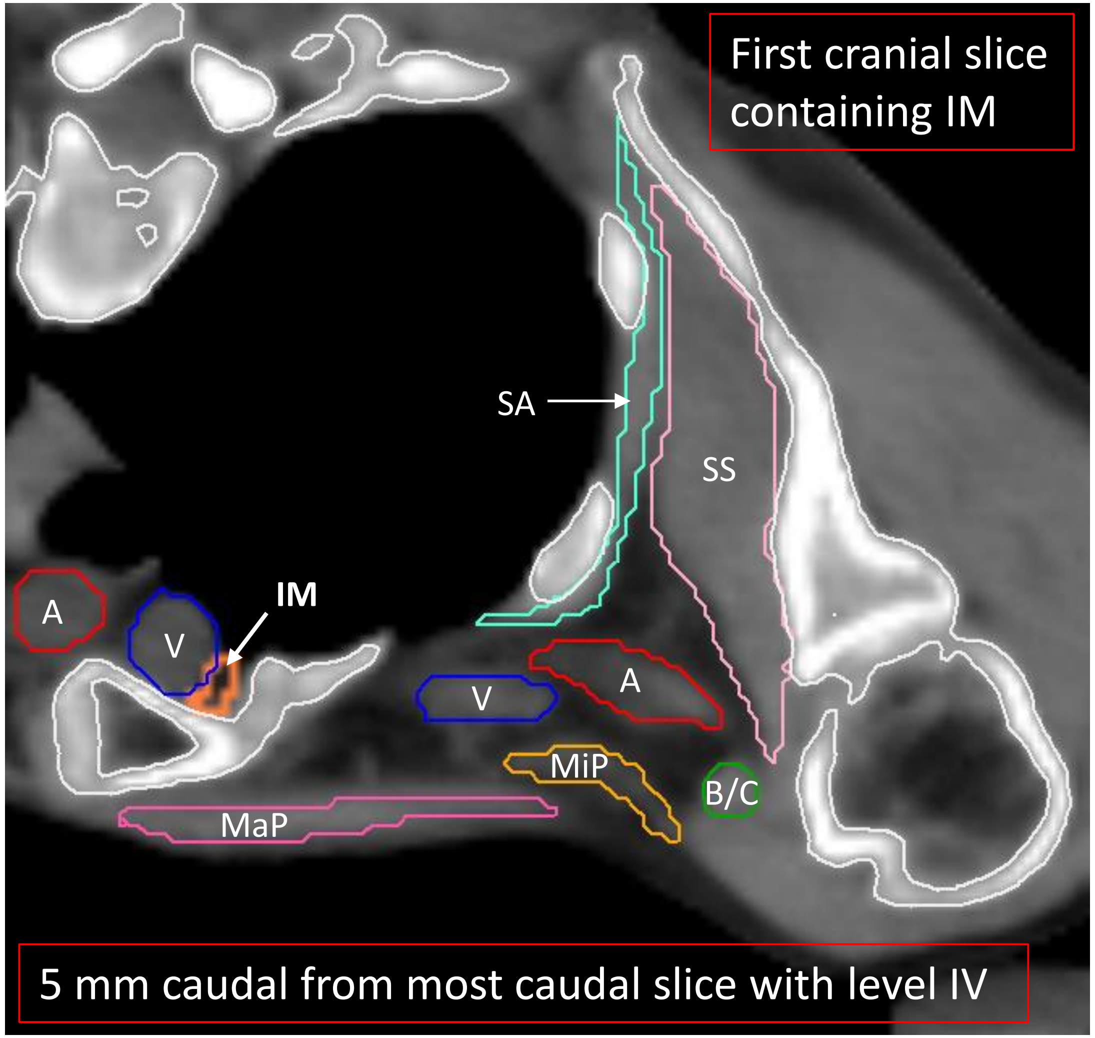

Internal mammary nodes, rare finding

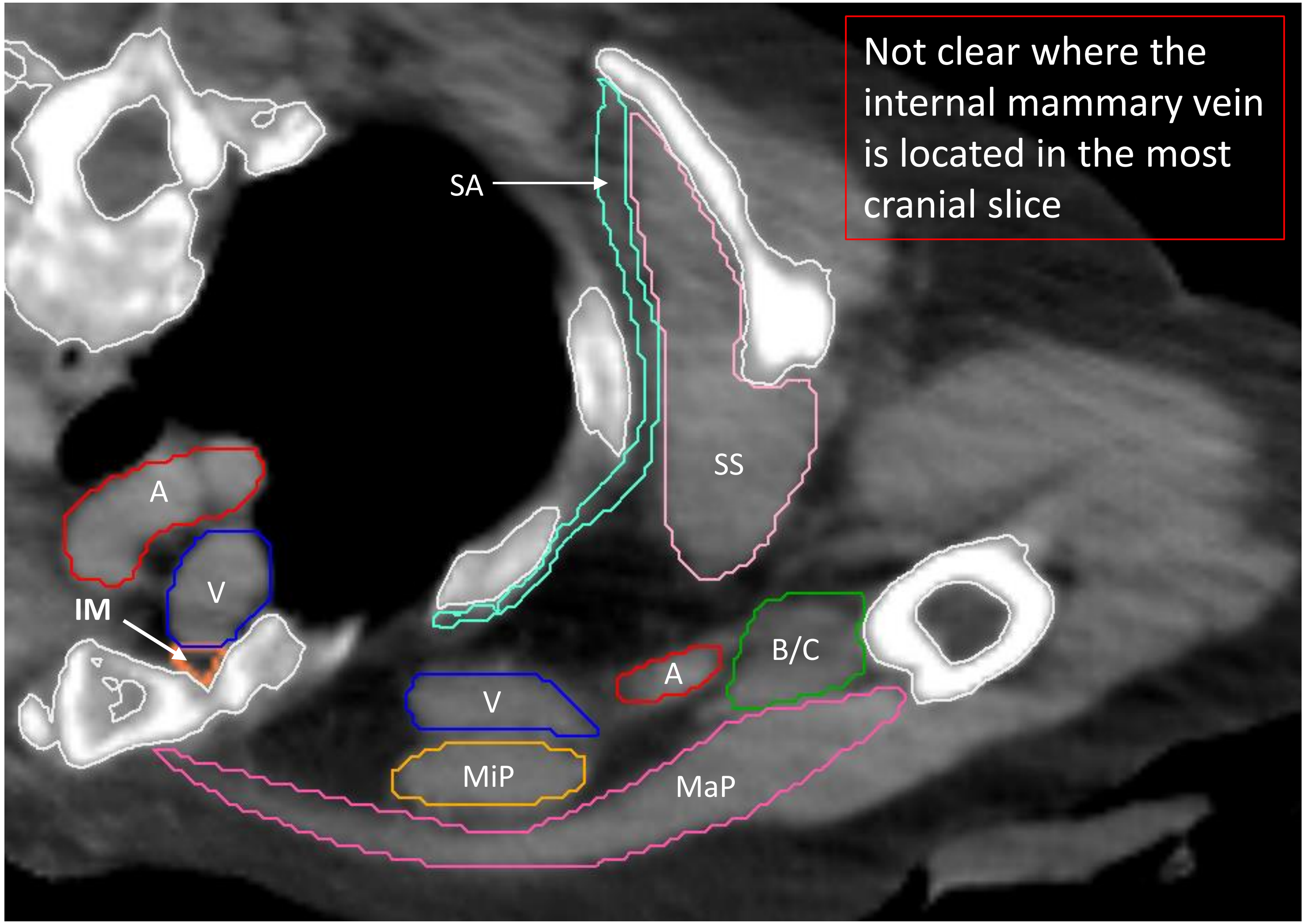

If the point where the internal mammary vein joins the brachiocephalic vein cannot clearly be distinguished in the most cranial slices, or it is not present yet, the CTV is defined by the area between the dorsal edge of the clavicle, the dorsal edge of the 1st rib and the ventral edge of the brachiocephalic vein.

This ensures that the internal mammary vein is always included, even if it cannot be distinguished and it provides a safety margin in the most cranial slices.

Internal mammary nodes caudal border

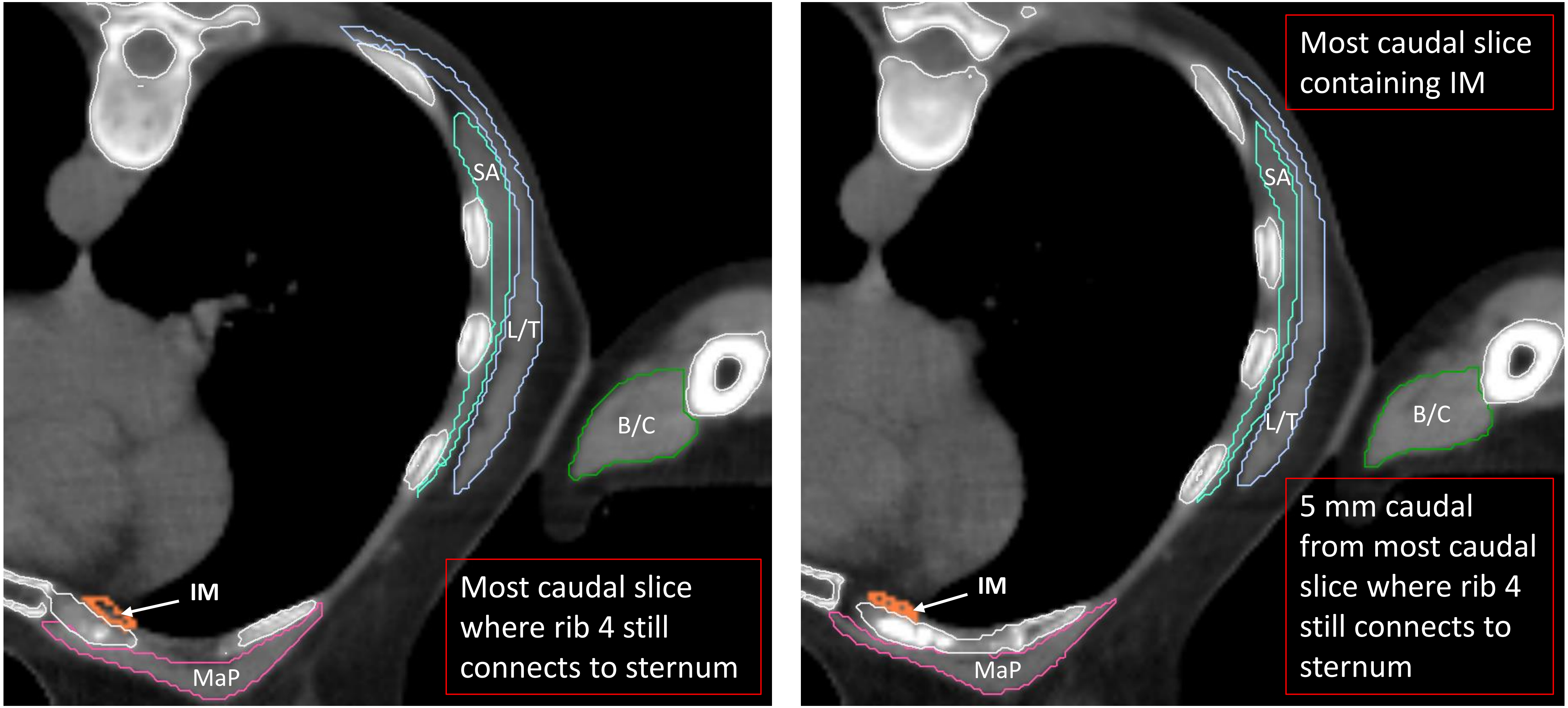

Internal mammary nodes medial border

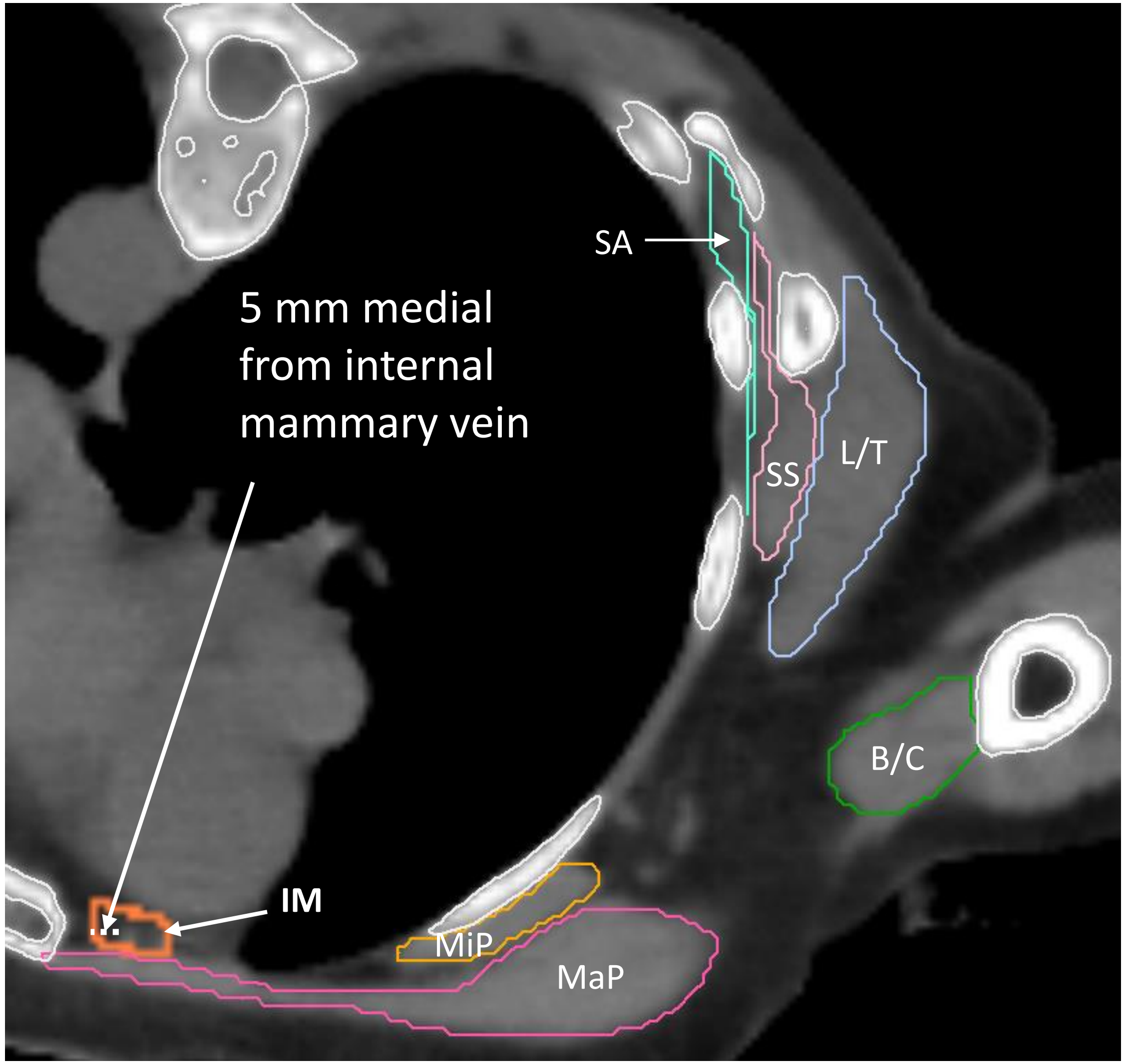

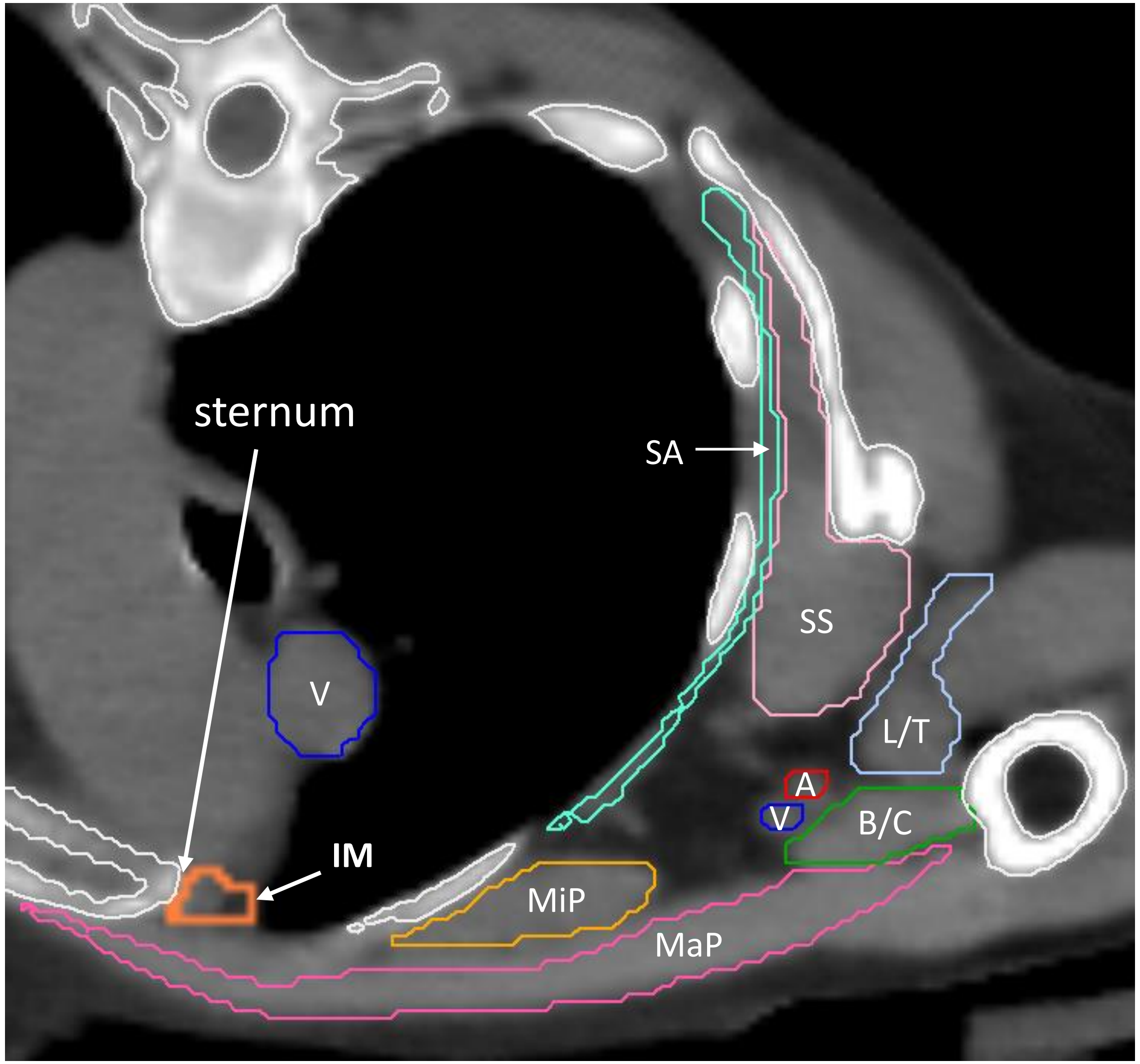

Internal mammary nodes lateral border

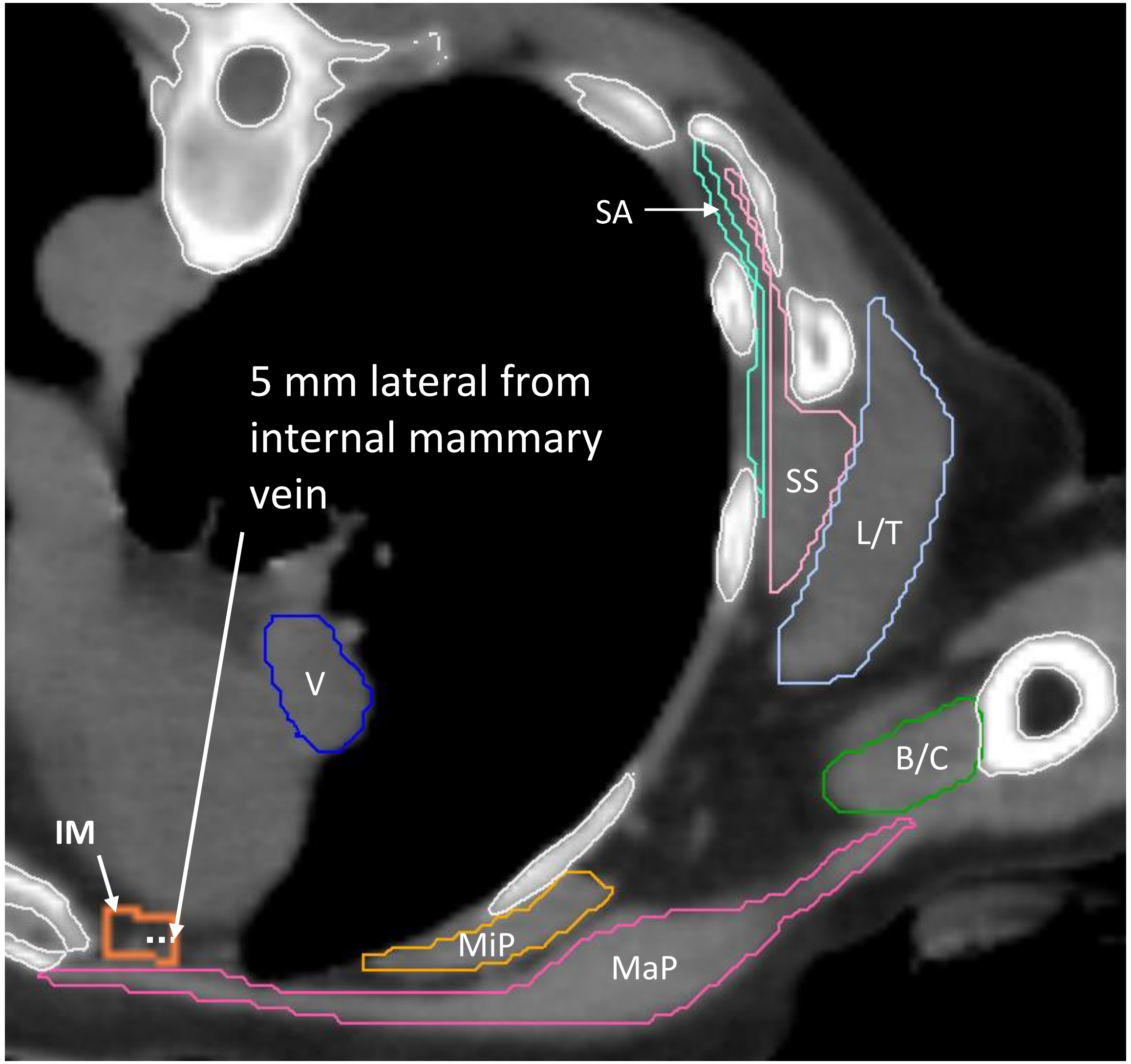

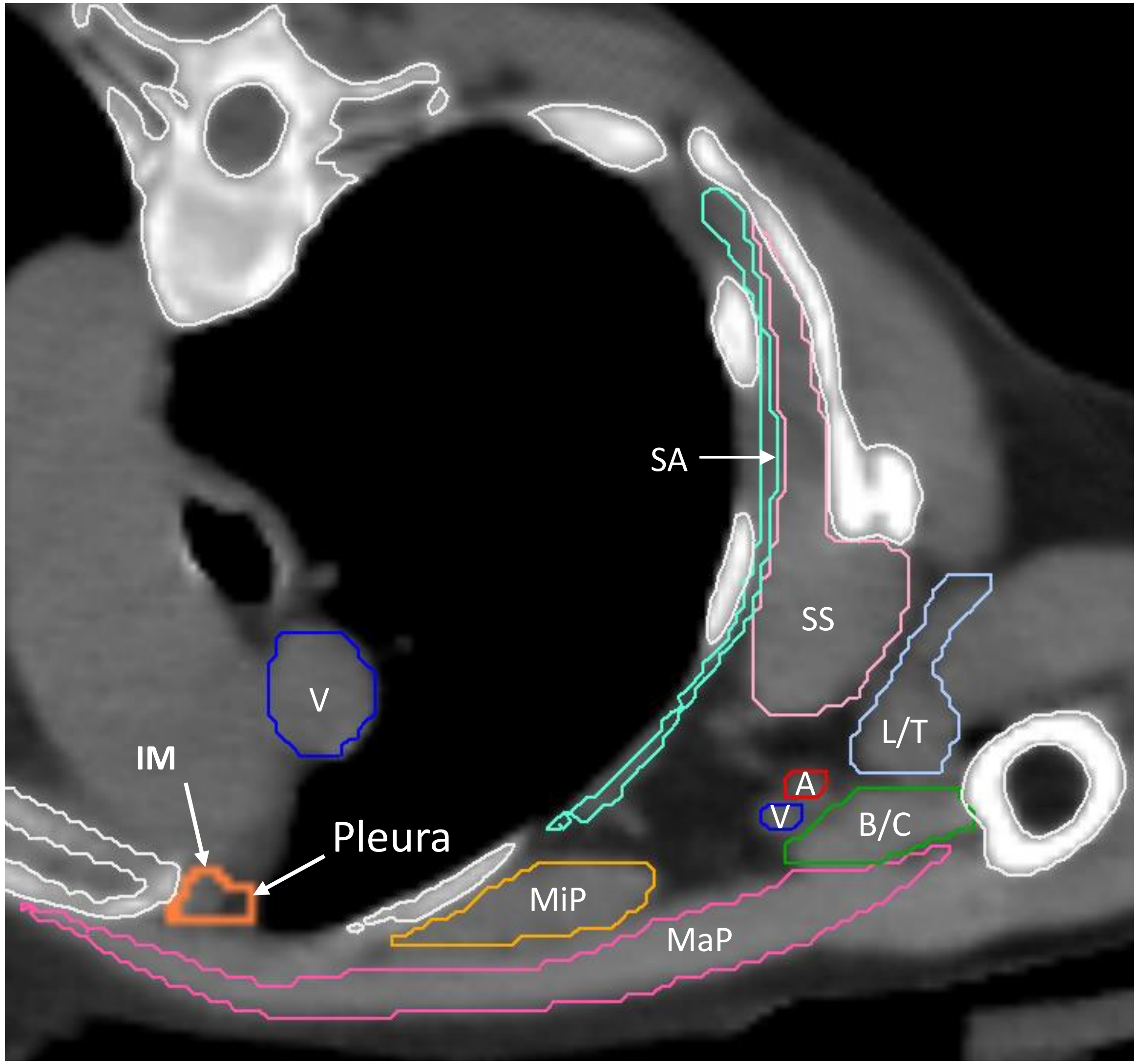

Internal mammary nodes ventral border

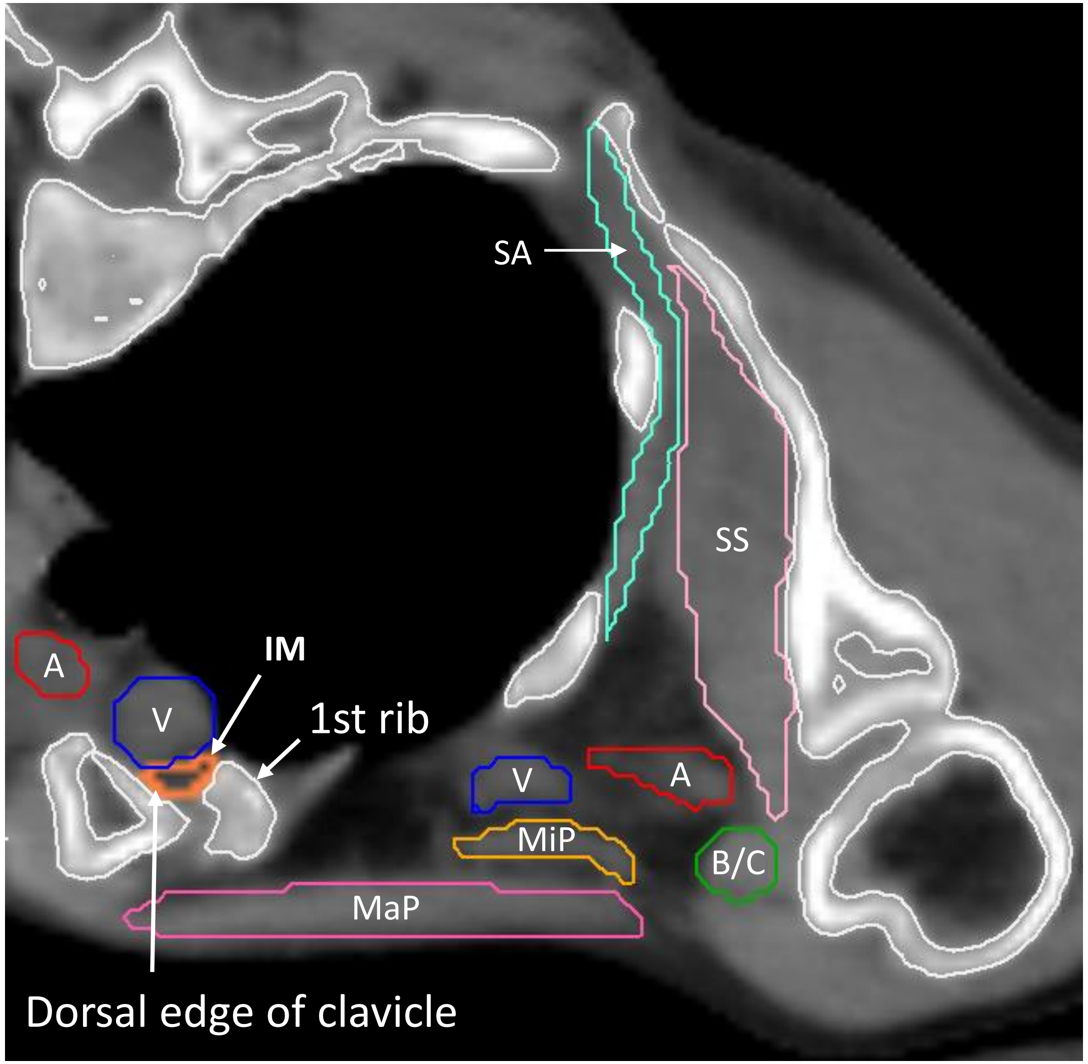

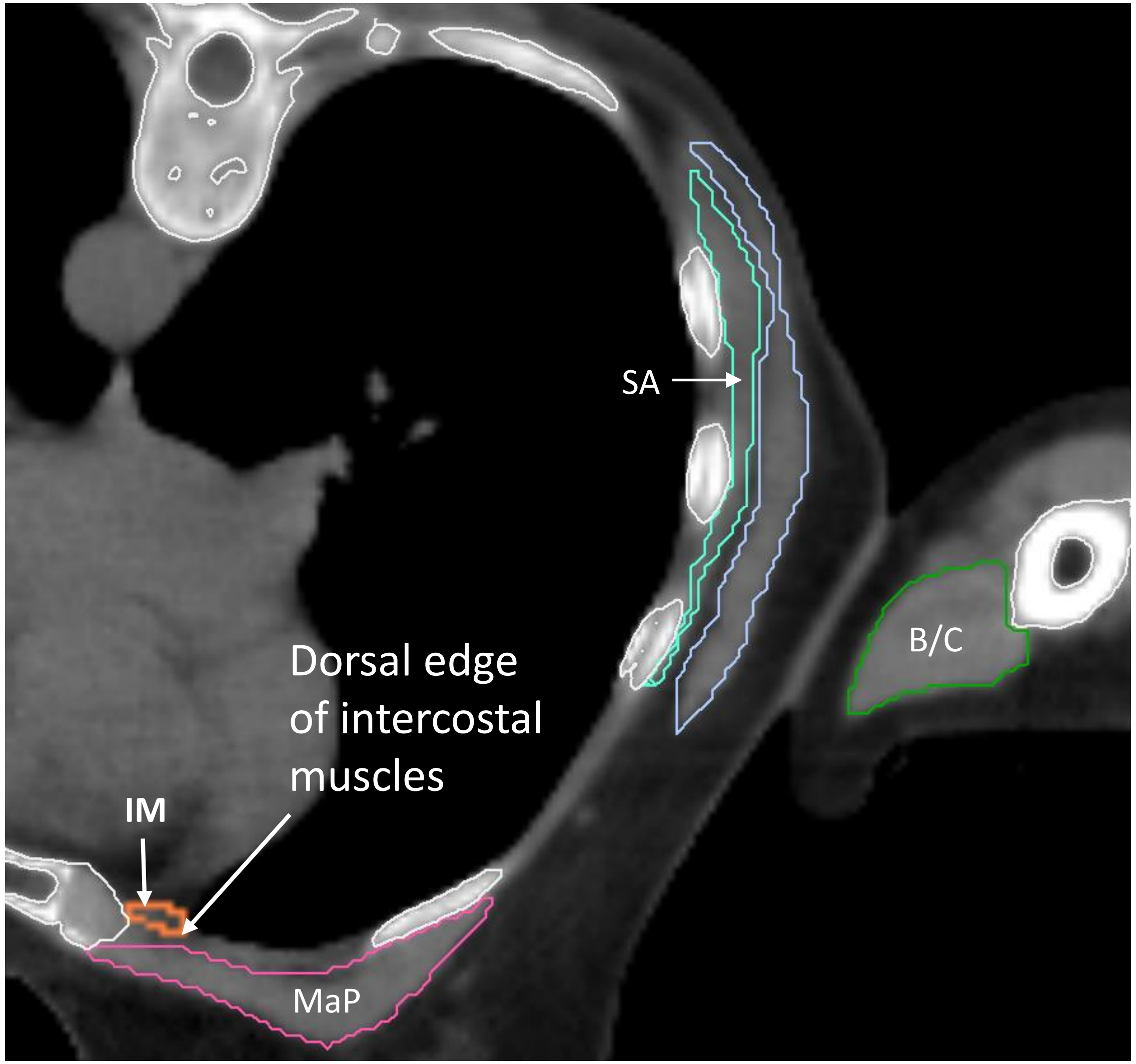

Internal mammary nodes dorsal border

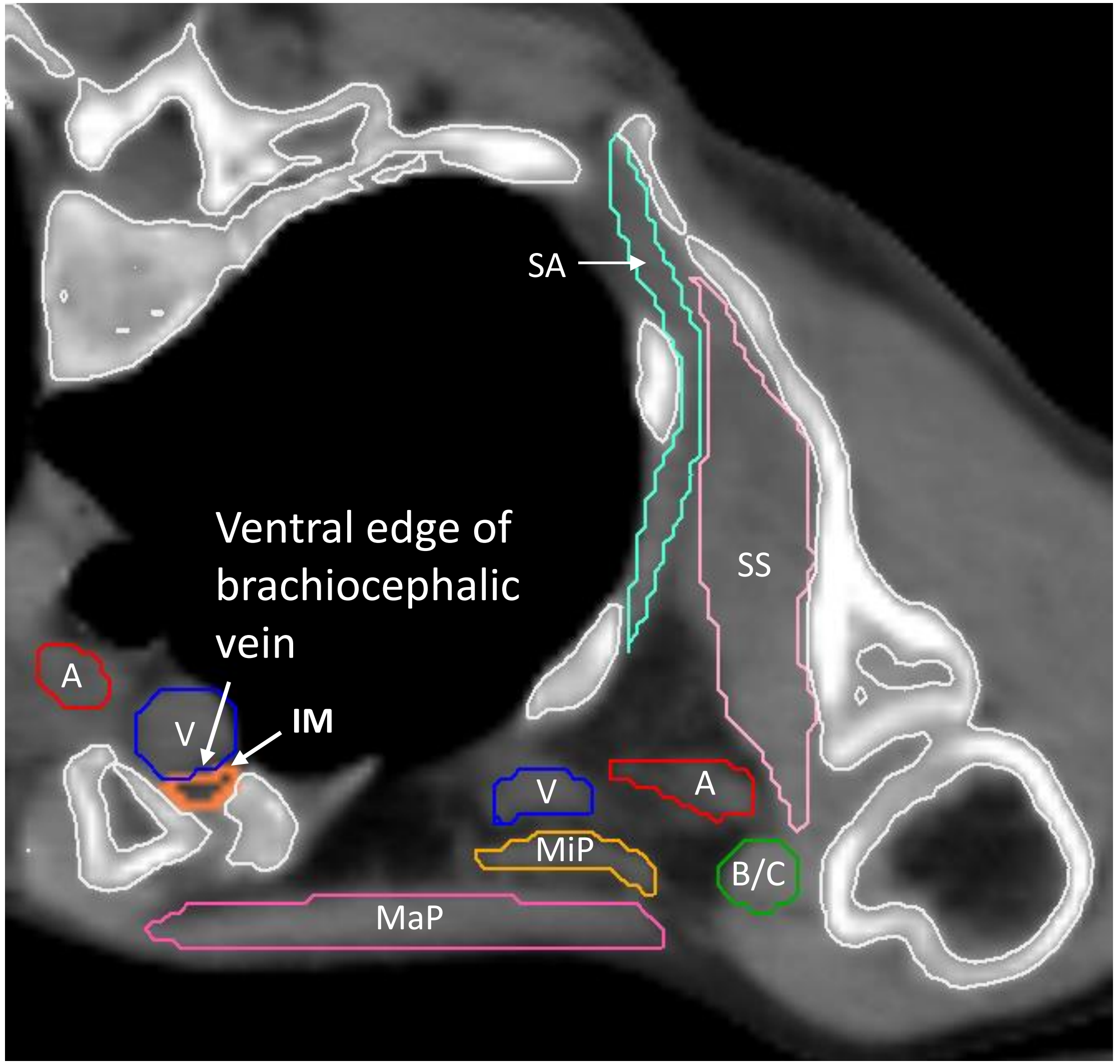

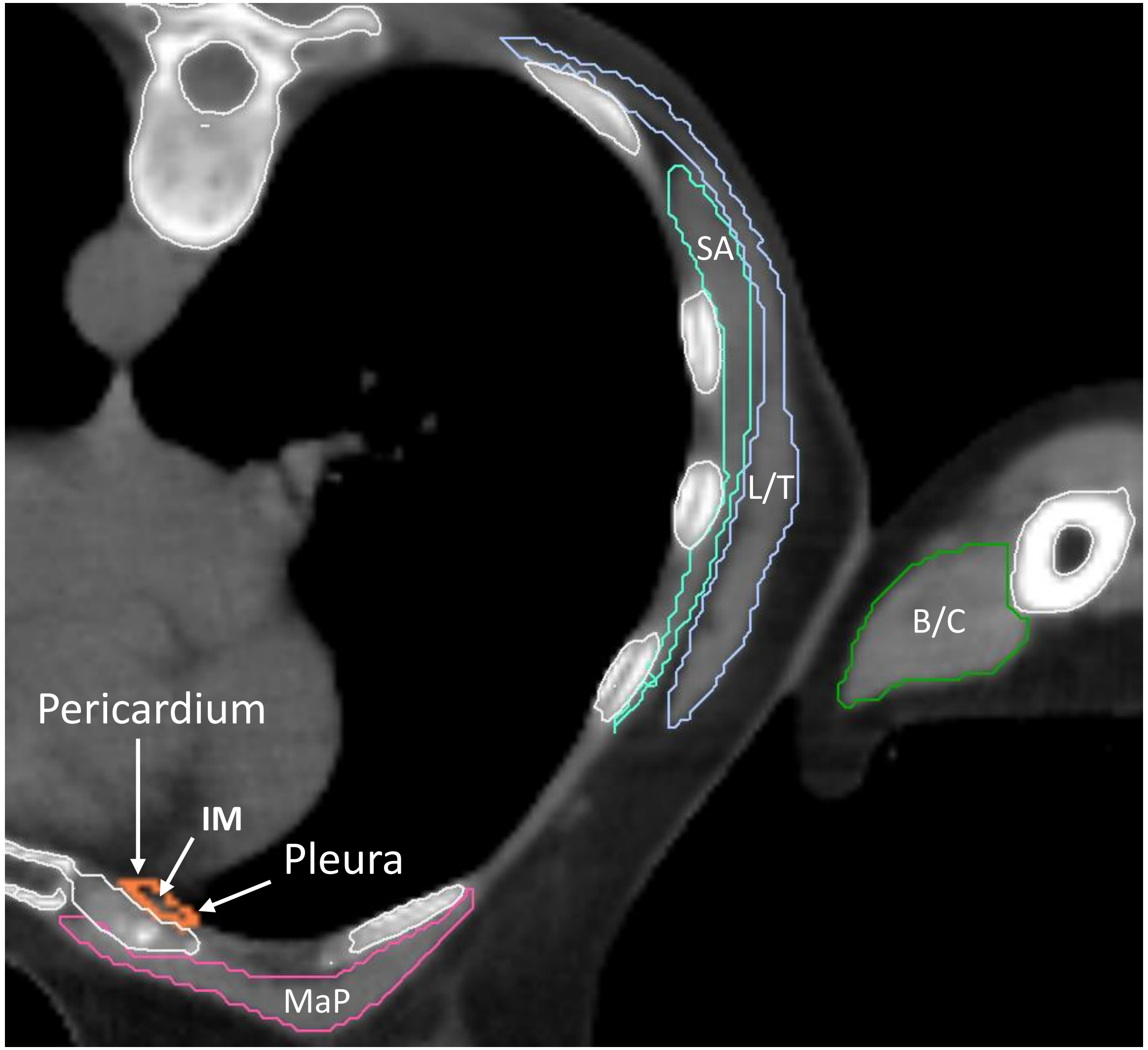

Interpectoral nodes cranial border

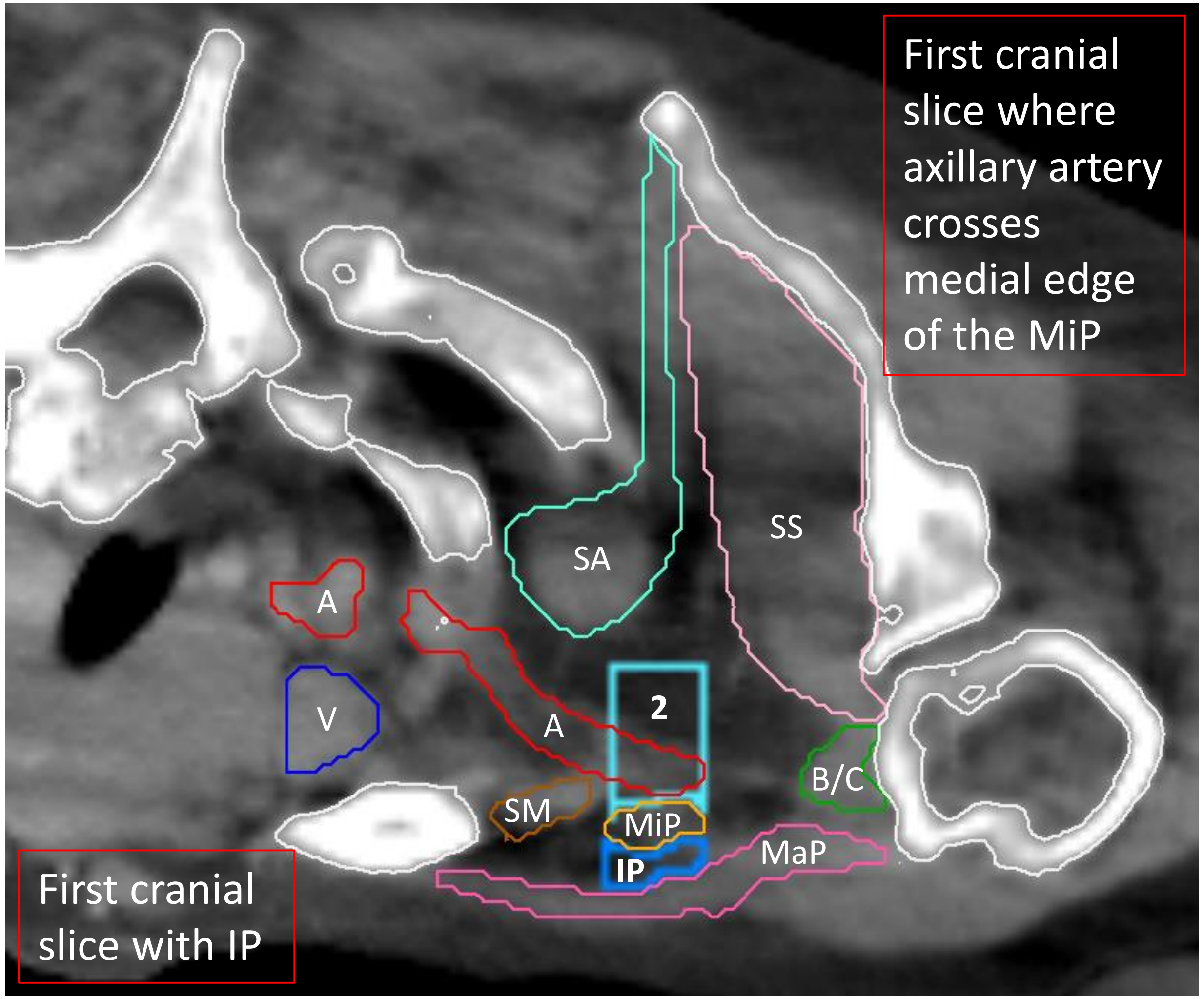

The cranial border of the interpectoral nodes starts at the first cranial slice where the axillary artery first crosses the medial border of the minor pectoral muscle.

This is the same cranial border as level II.

Interpectoral nodes (cranial alternative)

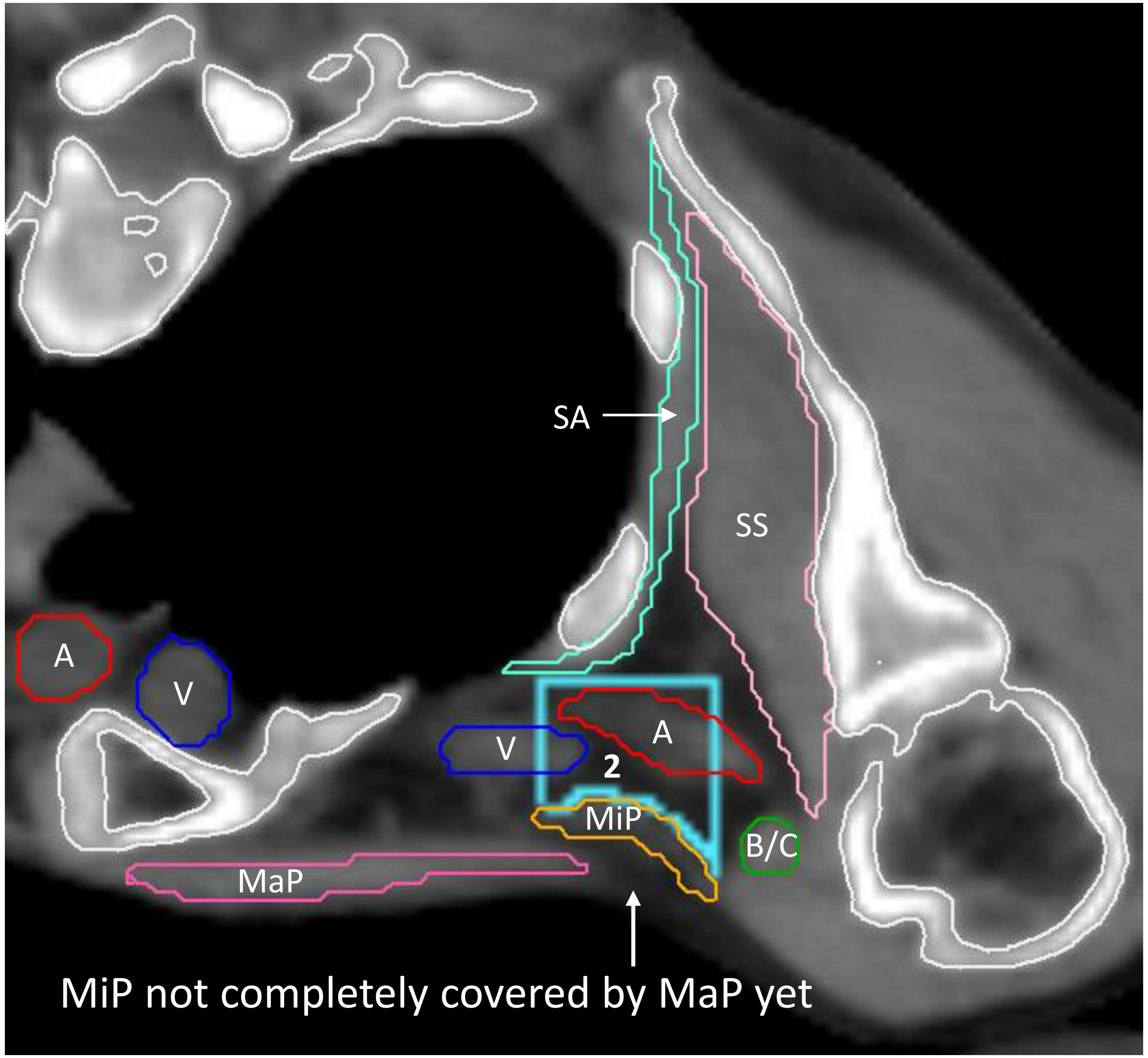

It is possible that in the most cranial slices the major pectoral muscle does not completely cover the minor pectoral muscle yet on the ventral aspect when the axillary artery has crossed the medial edge of the minor pectoral muscle.

If this occurs, the cranial border is moved caudally until the major pectoral muscle completely covers the minor pectoral muscle ventrally.

Interpectoral nodes caudal border

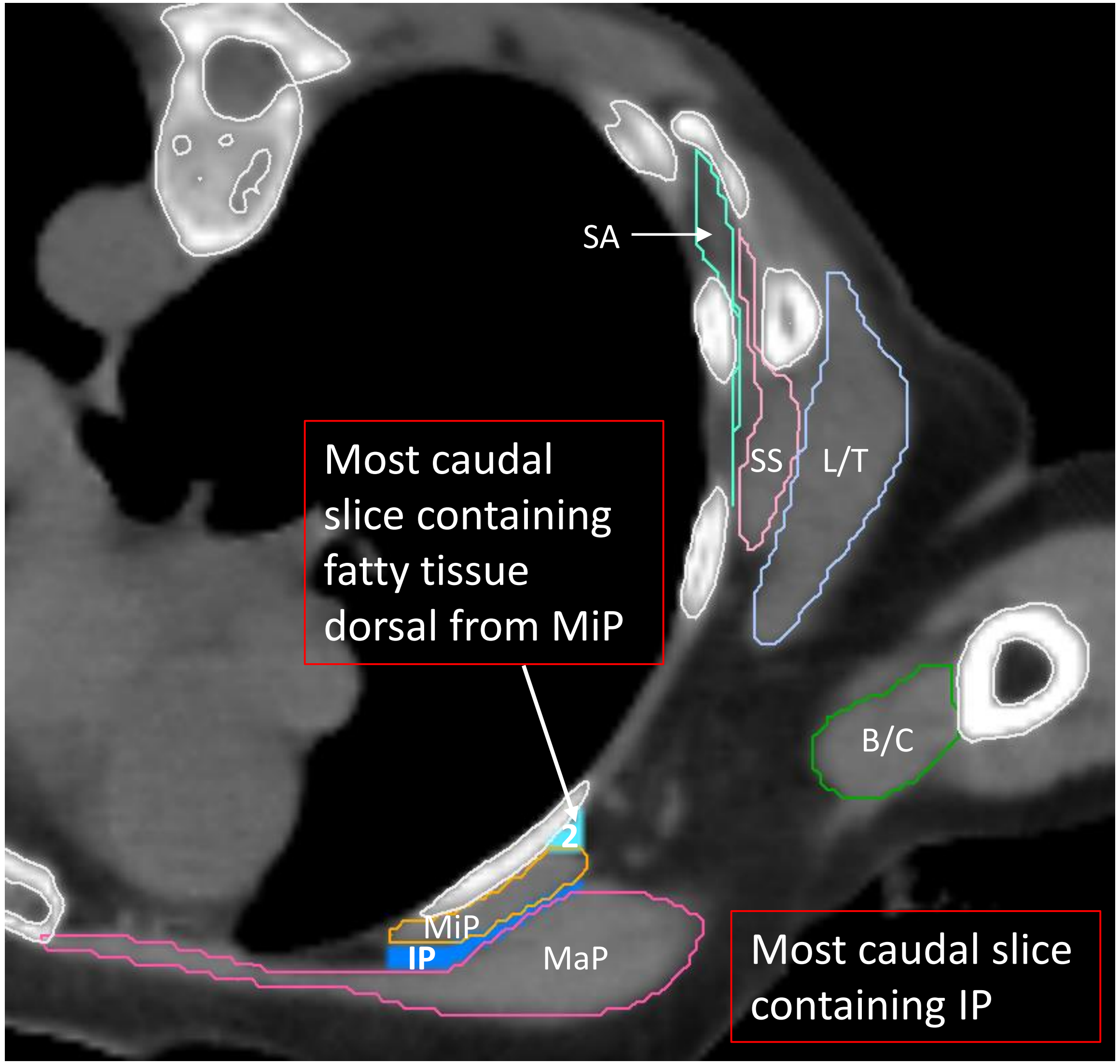

The caudal border is the most caudal slice that still contains fatty tissue dorsally from the minor pectoral muscle.

This is the same caudal border as for level II.

Interpectoral nodes medial border

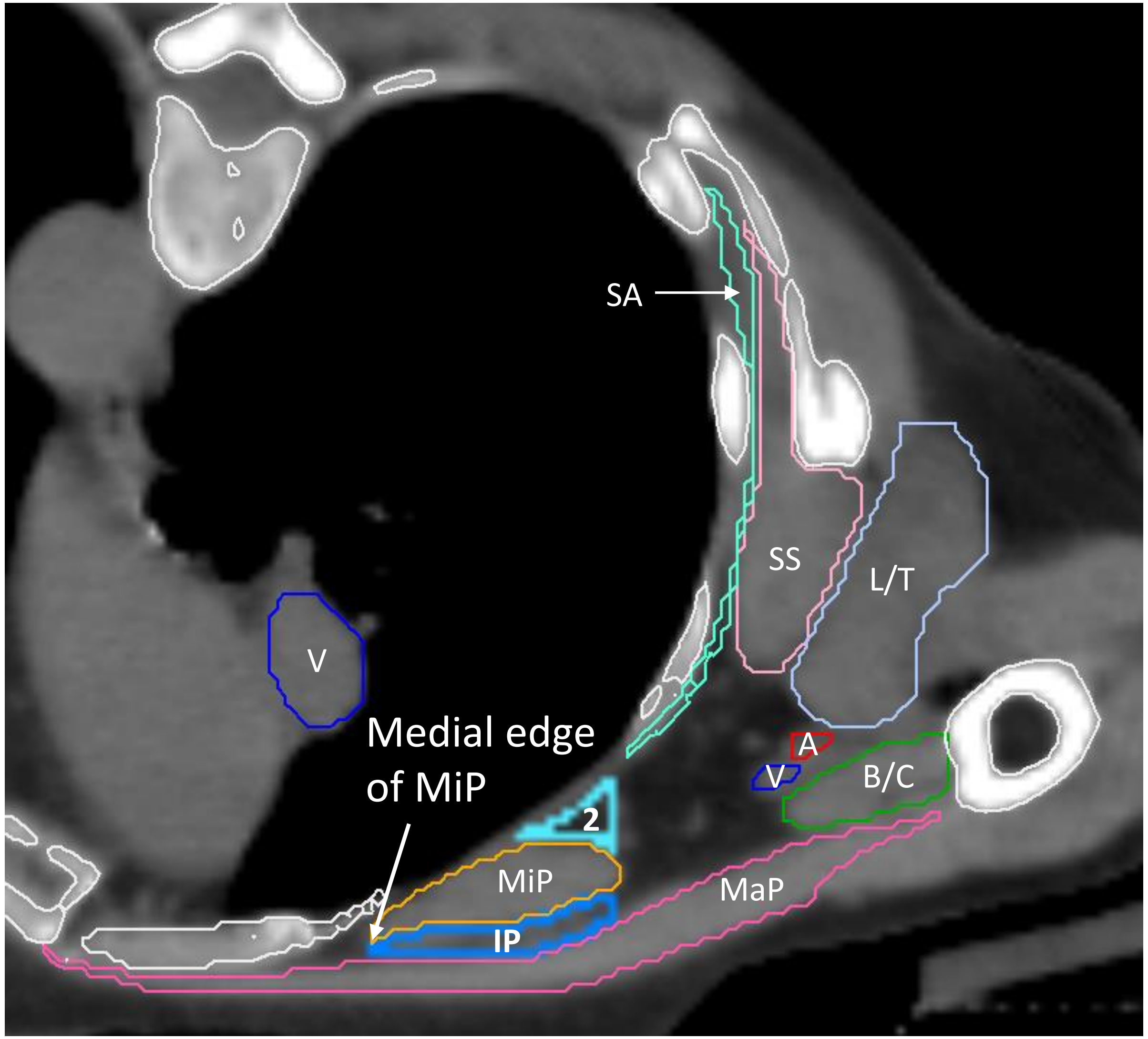

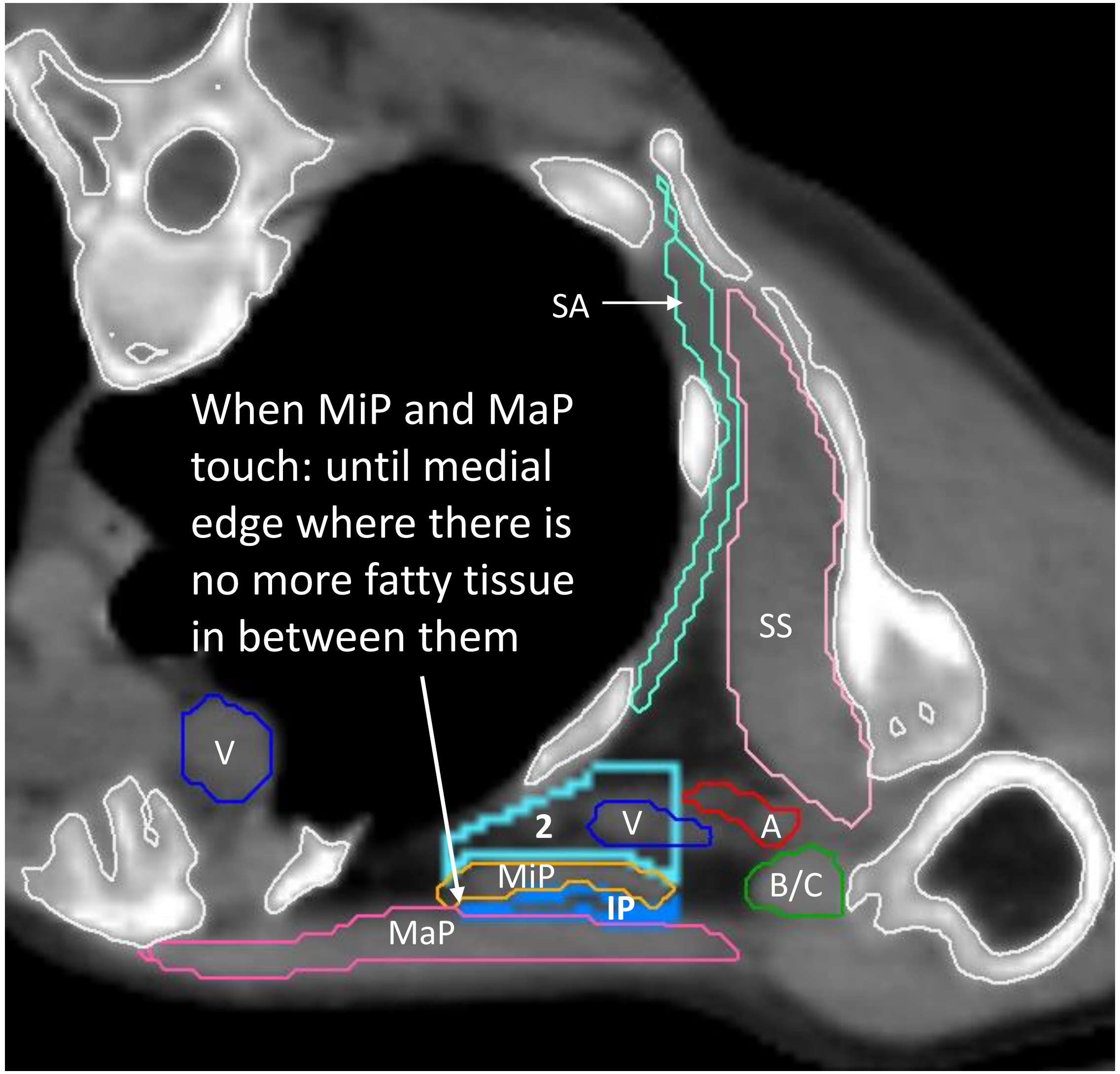

Interpectoral nodes lateral border

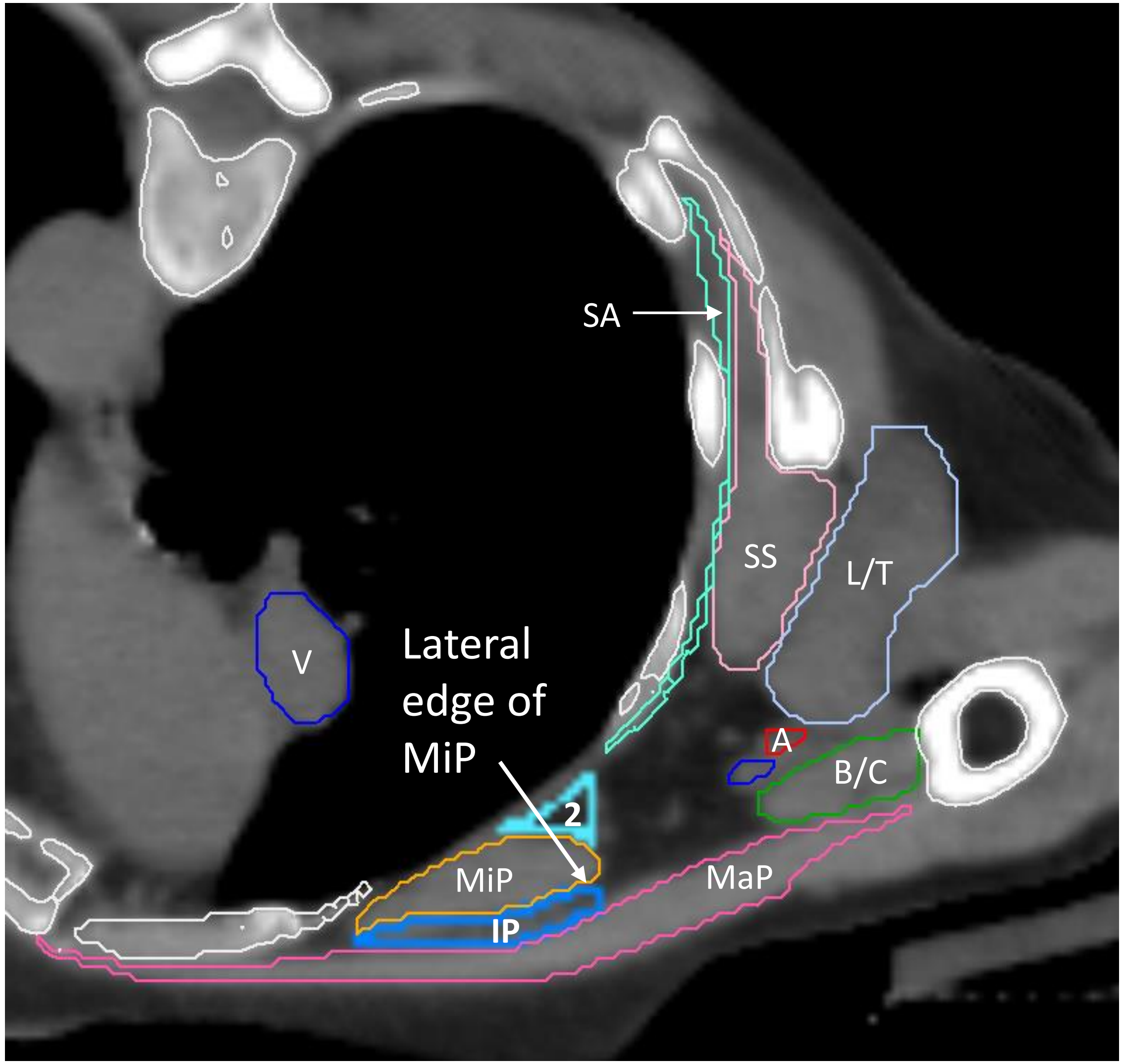

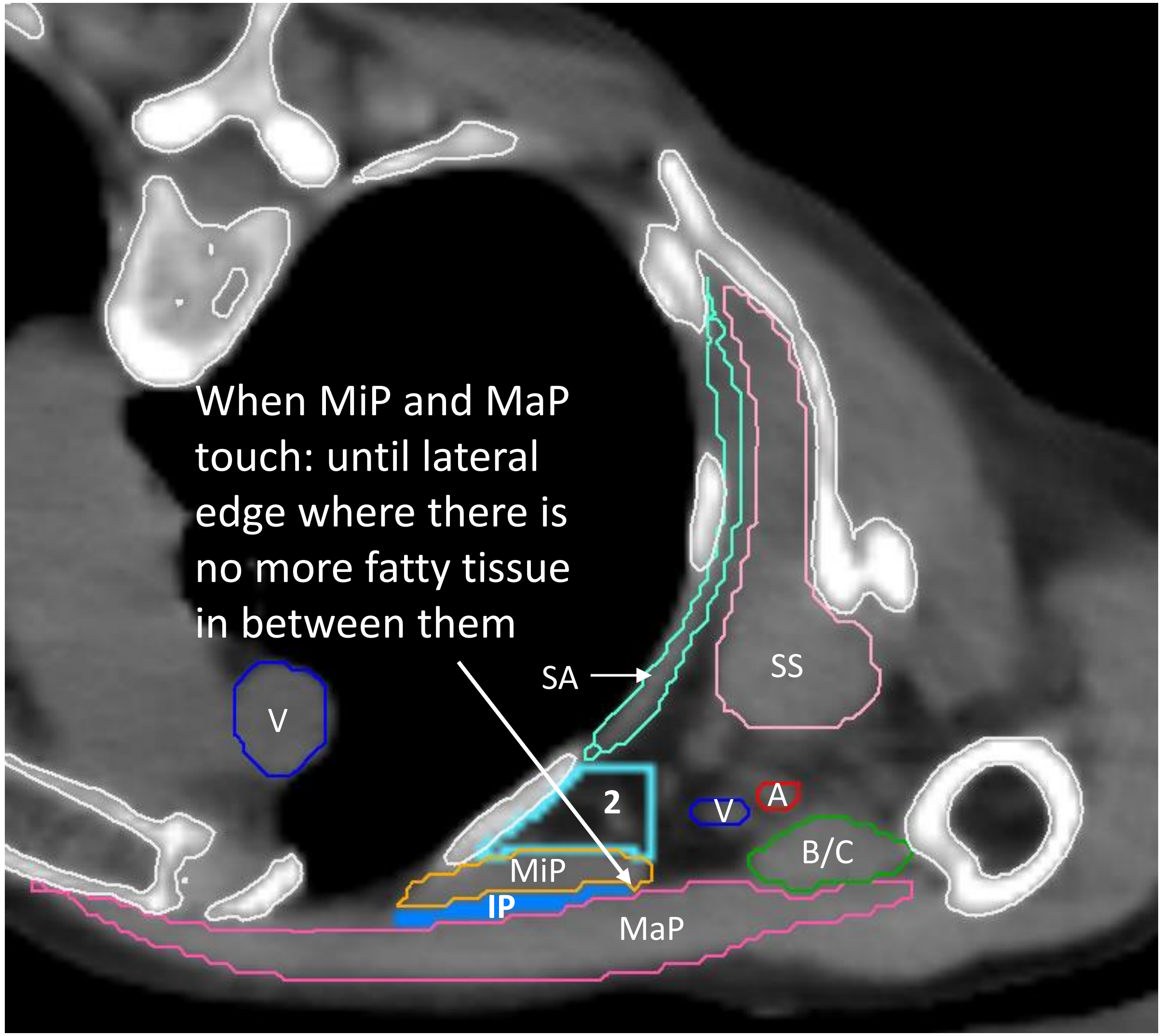

Interpectoral nodes ventral border

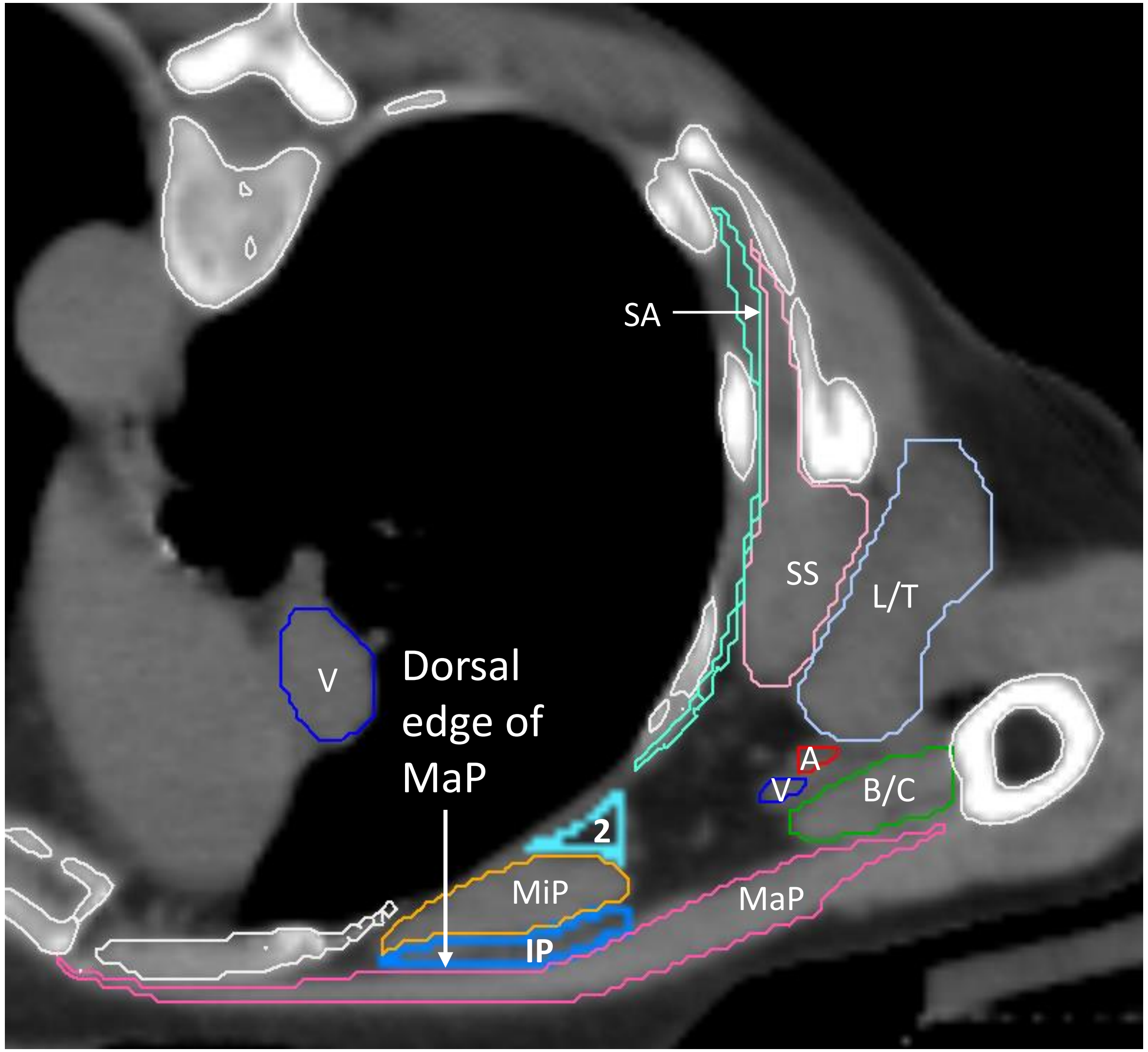

Interpectoral nodes dorsal border

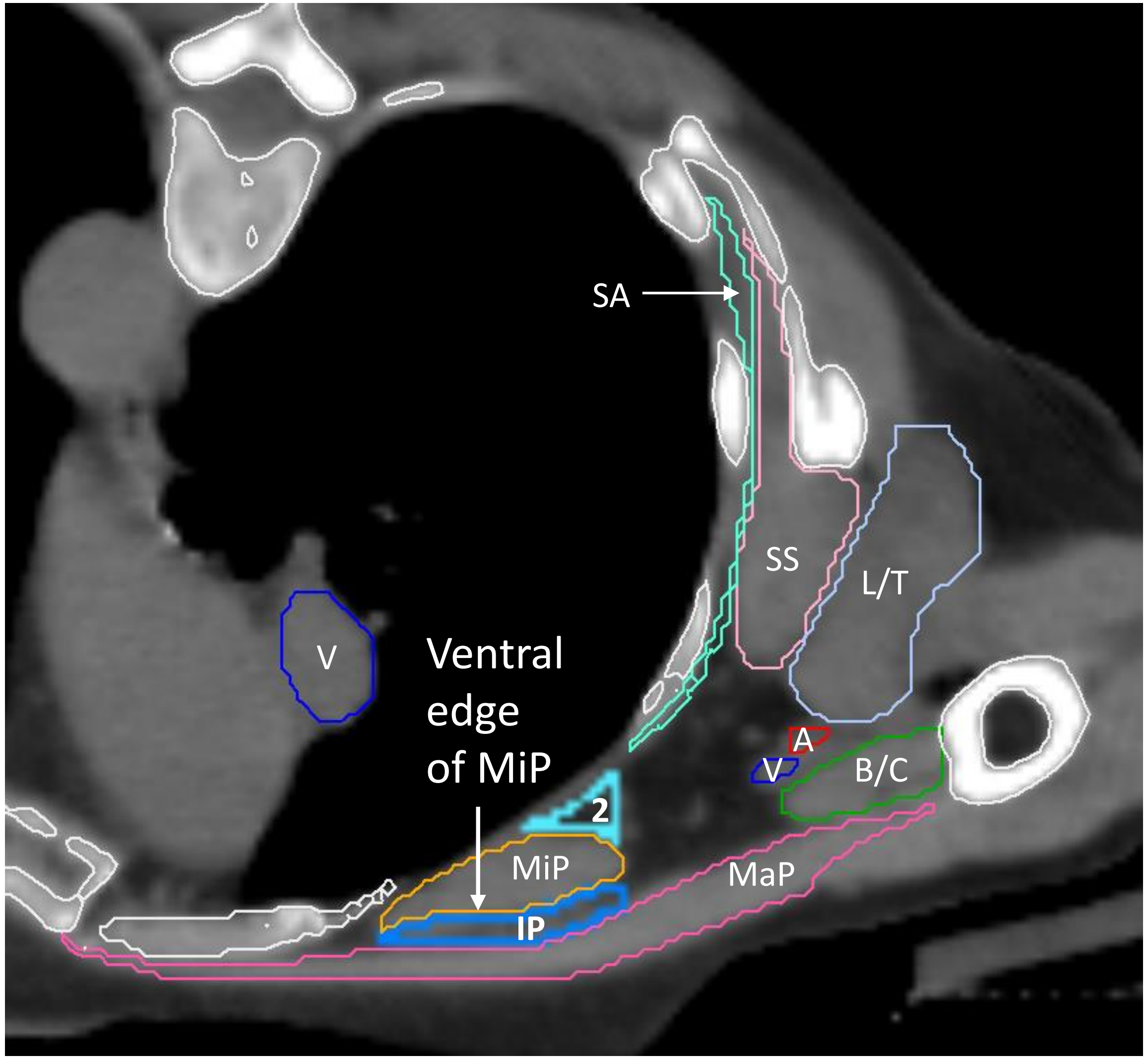

3D representation of the CTVs for all the patient CT scans used for guideline development

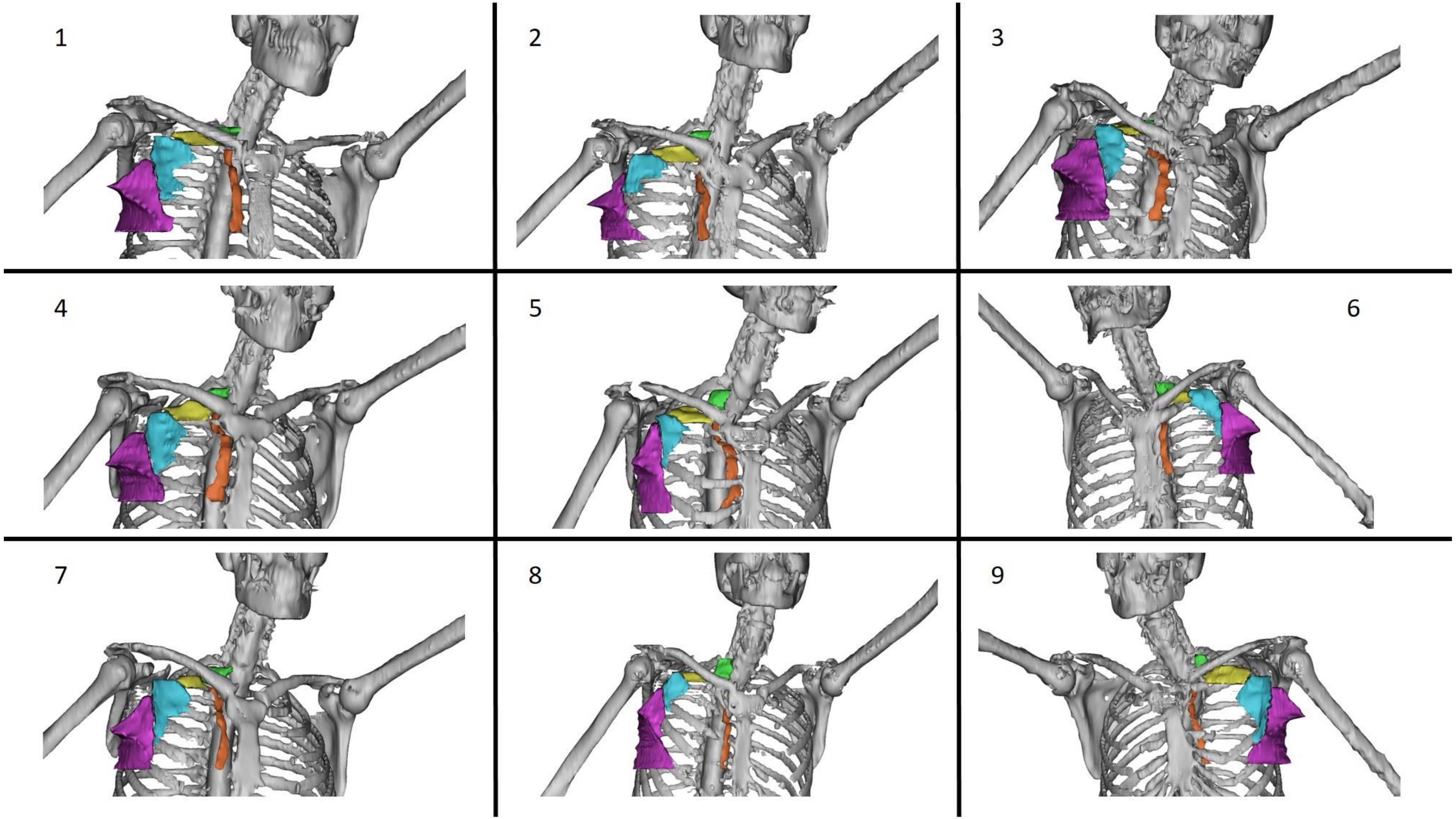

Supplement: Supplementary file 1 — Supplementary Information 1. [file 41598_2021_1841_MOESM1_ESM.pdf]
