# Supplementary material for: Delineation guidelines for the lymphatic target volumes in ‘prone crawl’ radiotherapy treatment position for breast cancer patients
Source: Sci Rep. 2021 Nov 18;11:22529. doi: 10.1038/s41598-021-01841-y (PMC8602302; doi:10.1038/s41598-021-01841-y)
Supplement: Supplementary file 2 — Supplementary Information 2. [file 41598_2021_1841_MOESM2_ESM.pdf]

Completely delineated dataset PCP

Linked to manuscript ‘Delineation guidelines for the lymphatic target volumes in ‘prone crawl’ radiotherapy treatment position for breast cancer patients ’

by

Michael E. J. Stouthandel, Françoise Kayser, Vincent Vakaet, Ralph Khoury, Pieter Deseyne, Chris Monten, Max Schoepen, Vincent Remouchamps, Alex De Caluwé, Guillaume Janoray, Wilfried De Neve, Stephane Mazy, Liv Veldeman, Tom Van Hoof.

Contour colour legend

vein (V)

artery (A)

Level IV (4)

Level III (3)

Level II (2)

Level I (1)

Interpectoral nodes (IP)

Internal mammary nodes (IM)

sternocleidomastoid muscle (SCM)

anterior scalene muscle (AS)

biceps/coracobrachial muscle (B/C)

serratus anterior muscle (SA)

major pectoral muscle (MaP)

lattissimus dorsi/teres major muscle (L/T)

subscapular muscle (SS)

triceps muscle (T)

bones (light grey)

subclavius muscle (SM)

minor pectoral muscle (MiP)

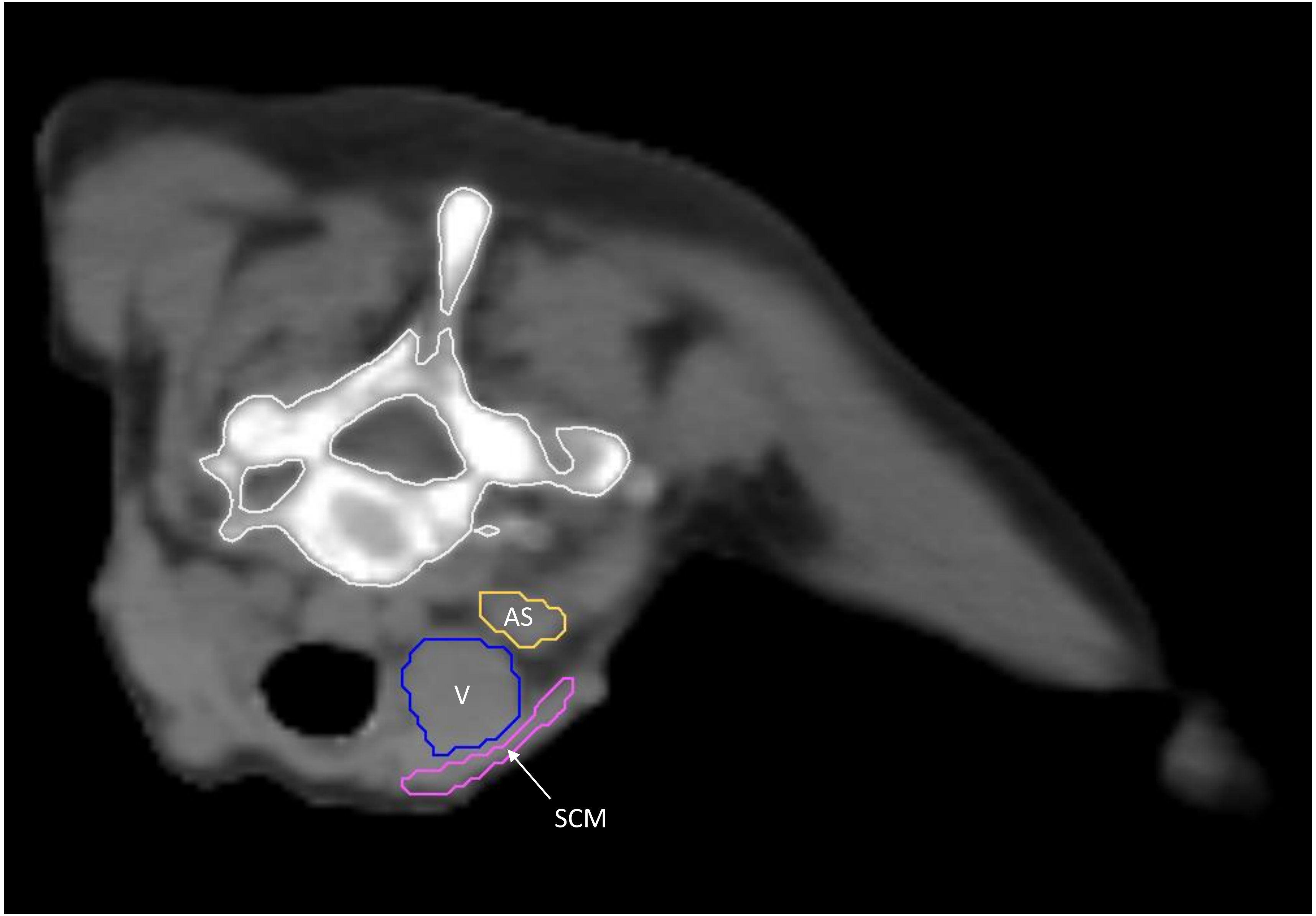
AS
V
SCM

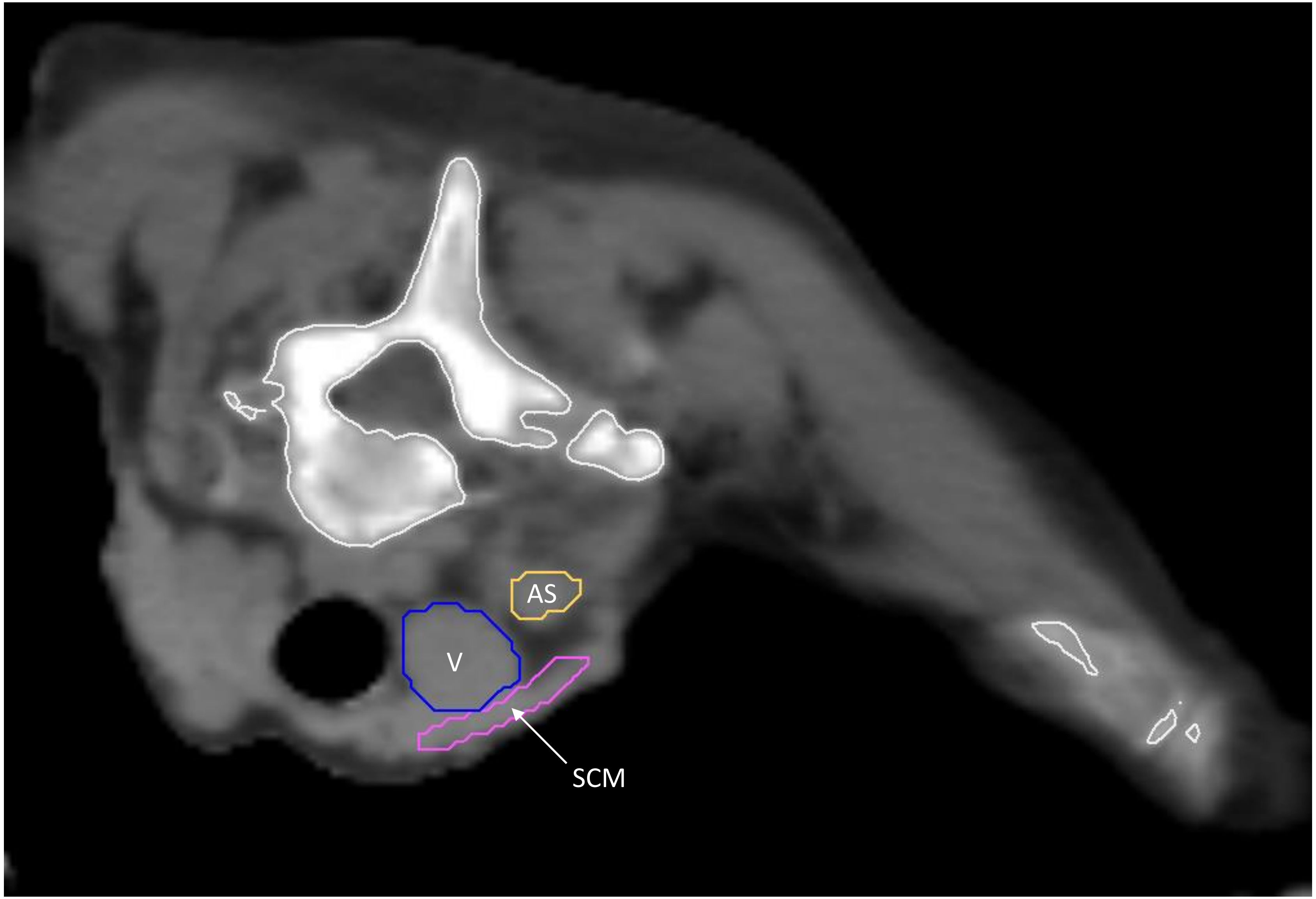
AS
V
SCM

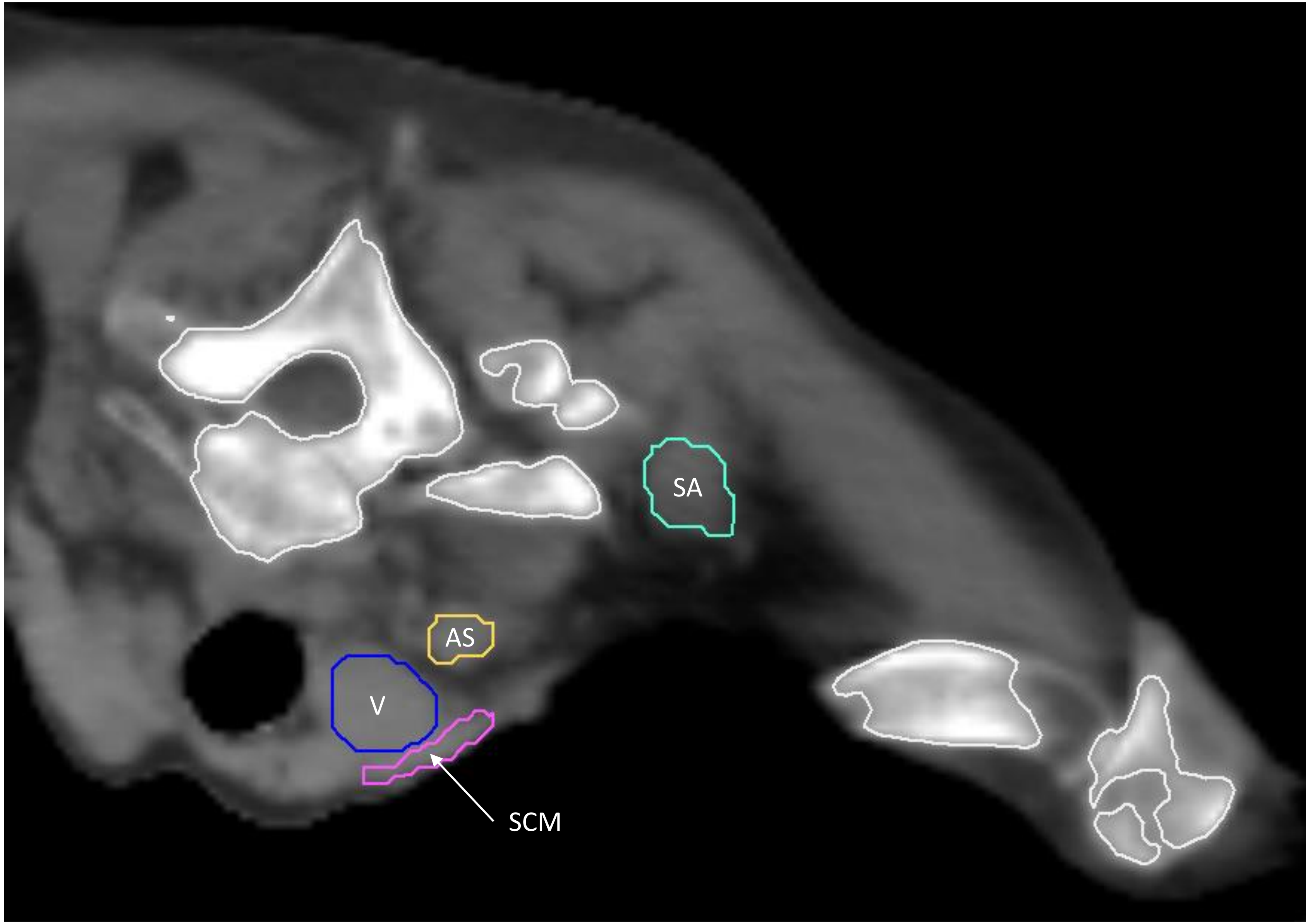
SA
AS
V
SCM

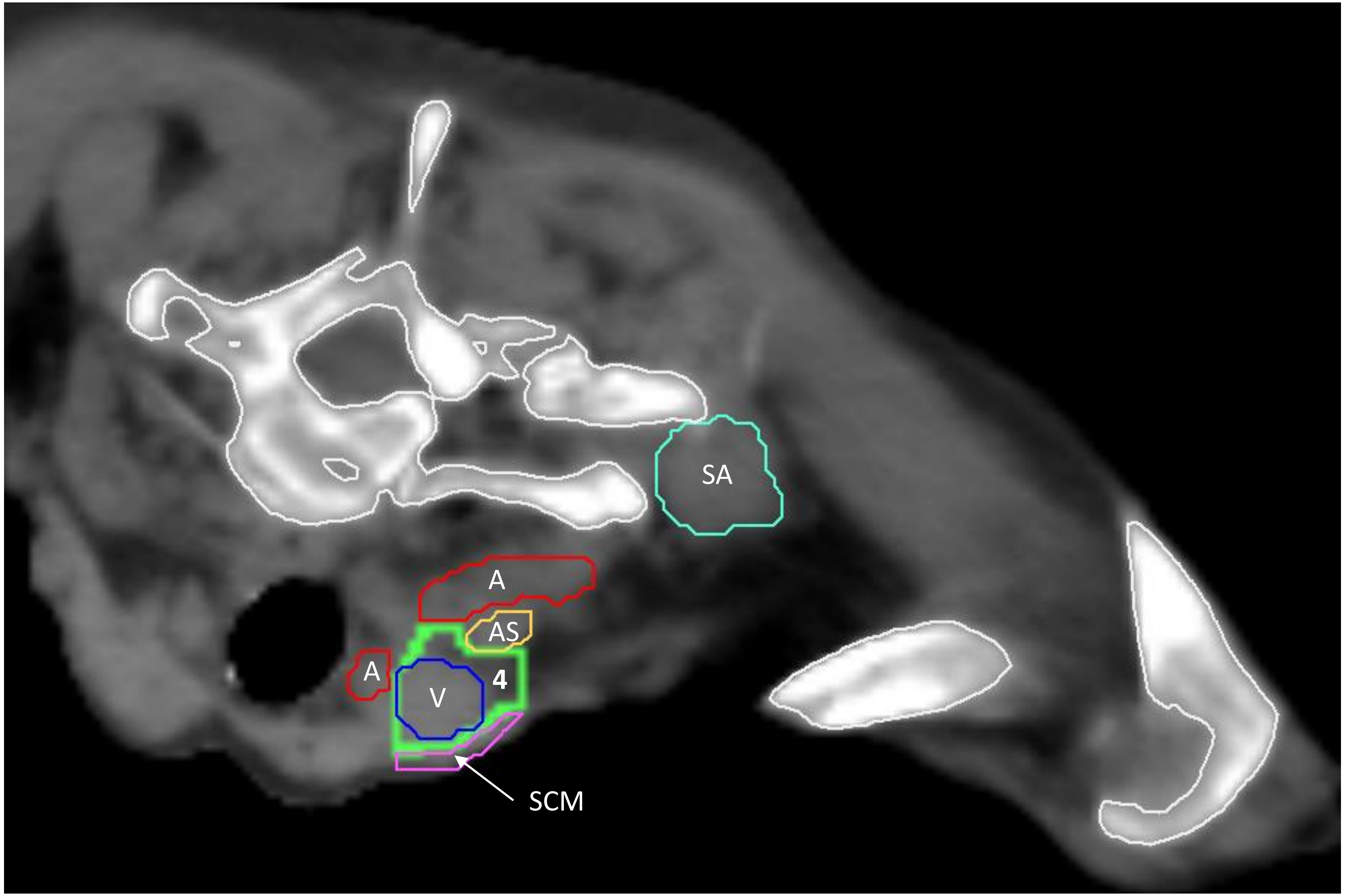
SA
A
AS
A
4
V
SCM

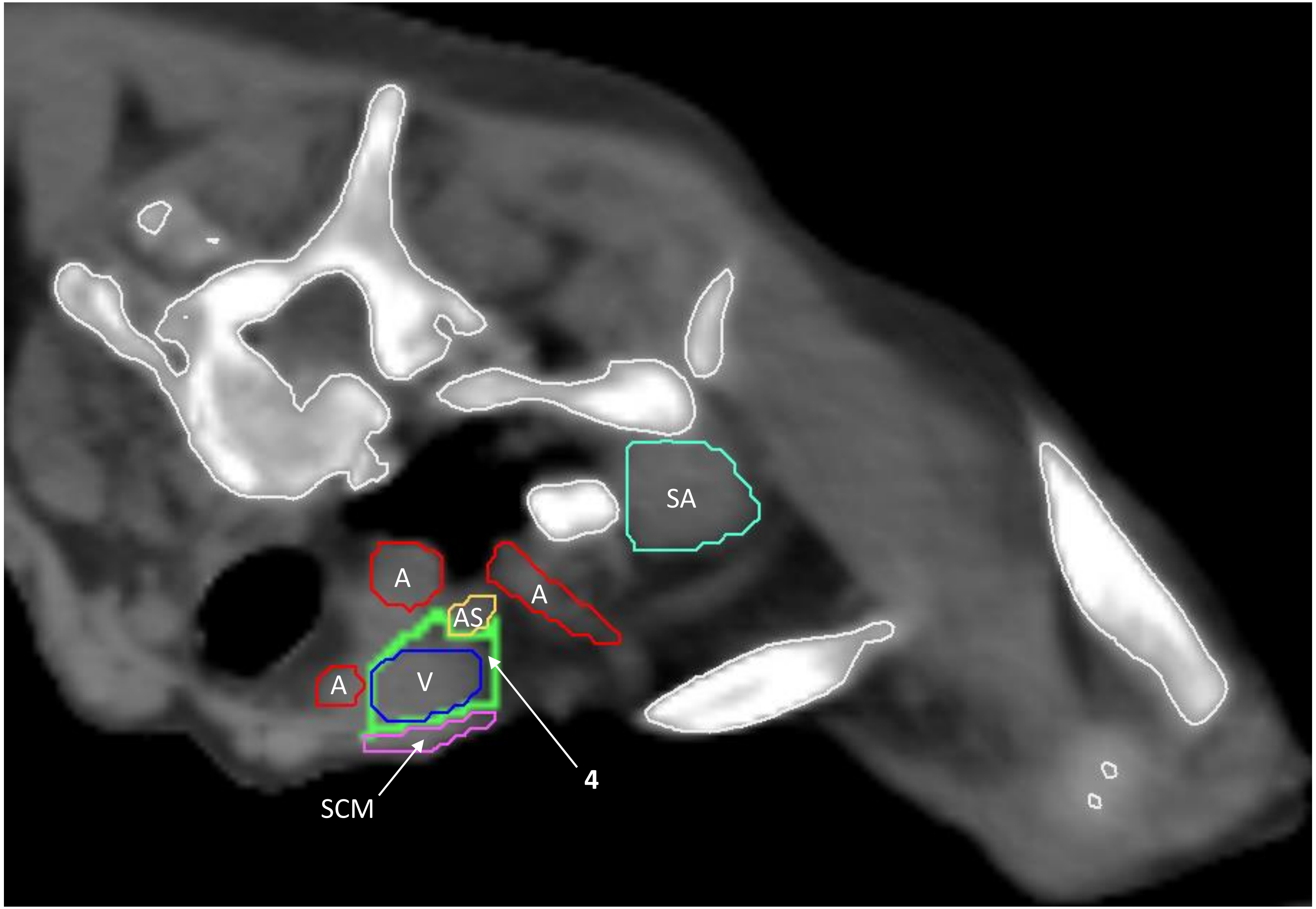
SA
A
A
AS
V
A
SCM
4

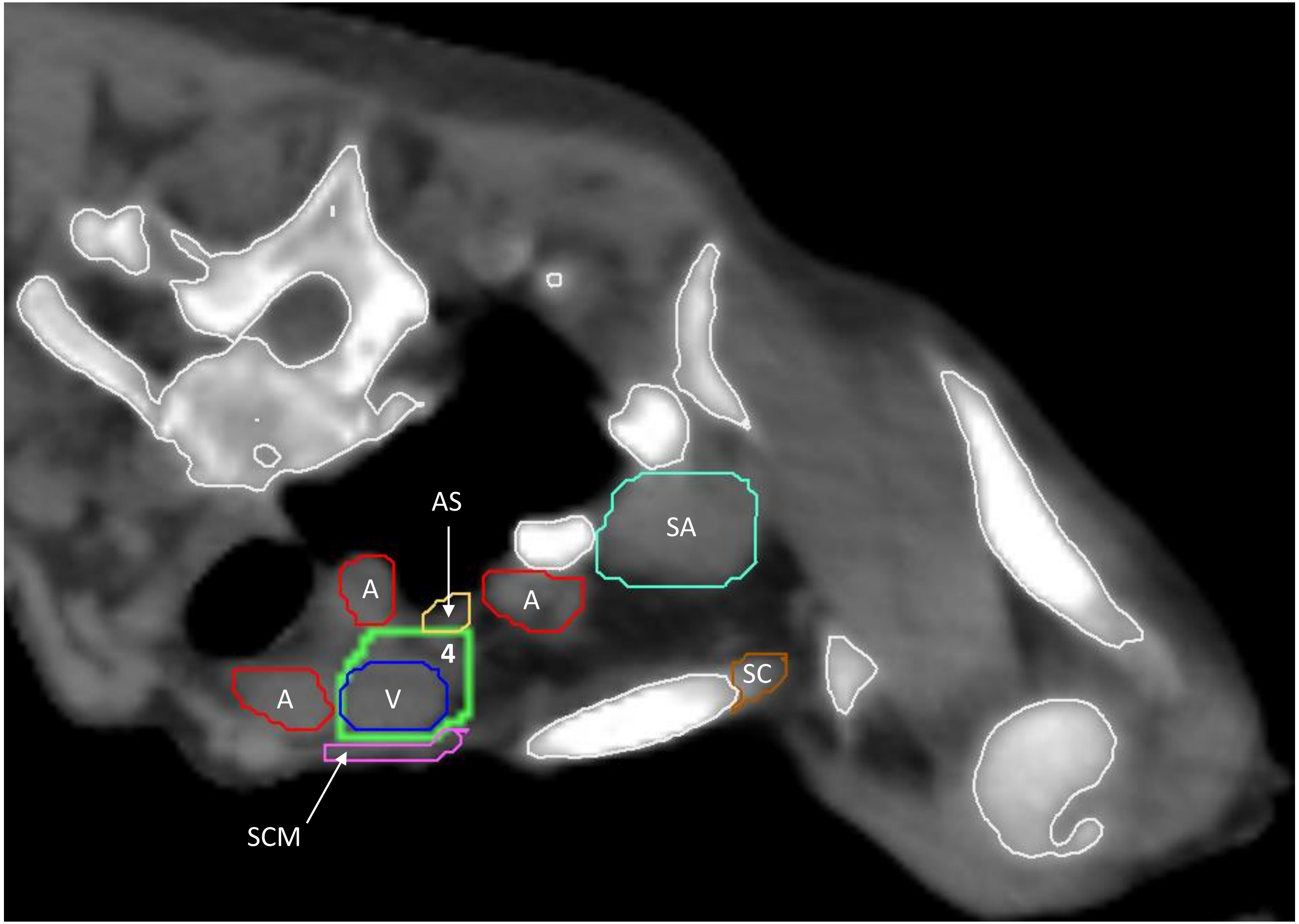
AS
SA
A
A
4
A
V
SC
SCM

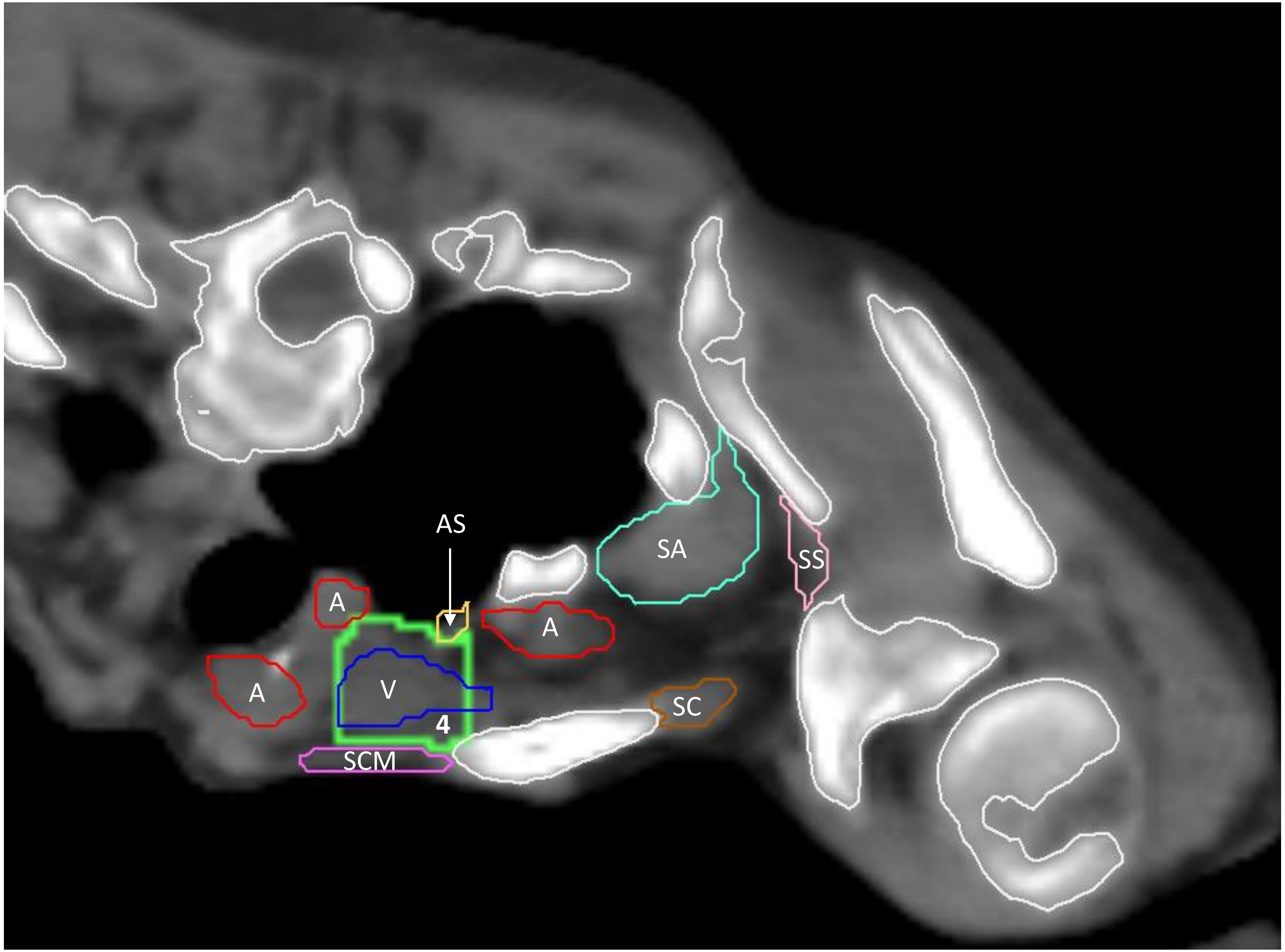
AS
A
A
A
SA
SS
V
4
SC
SCM

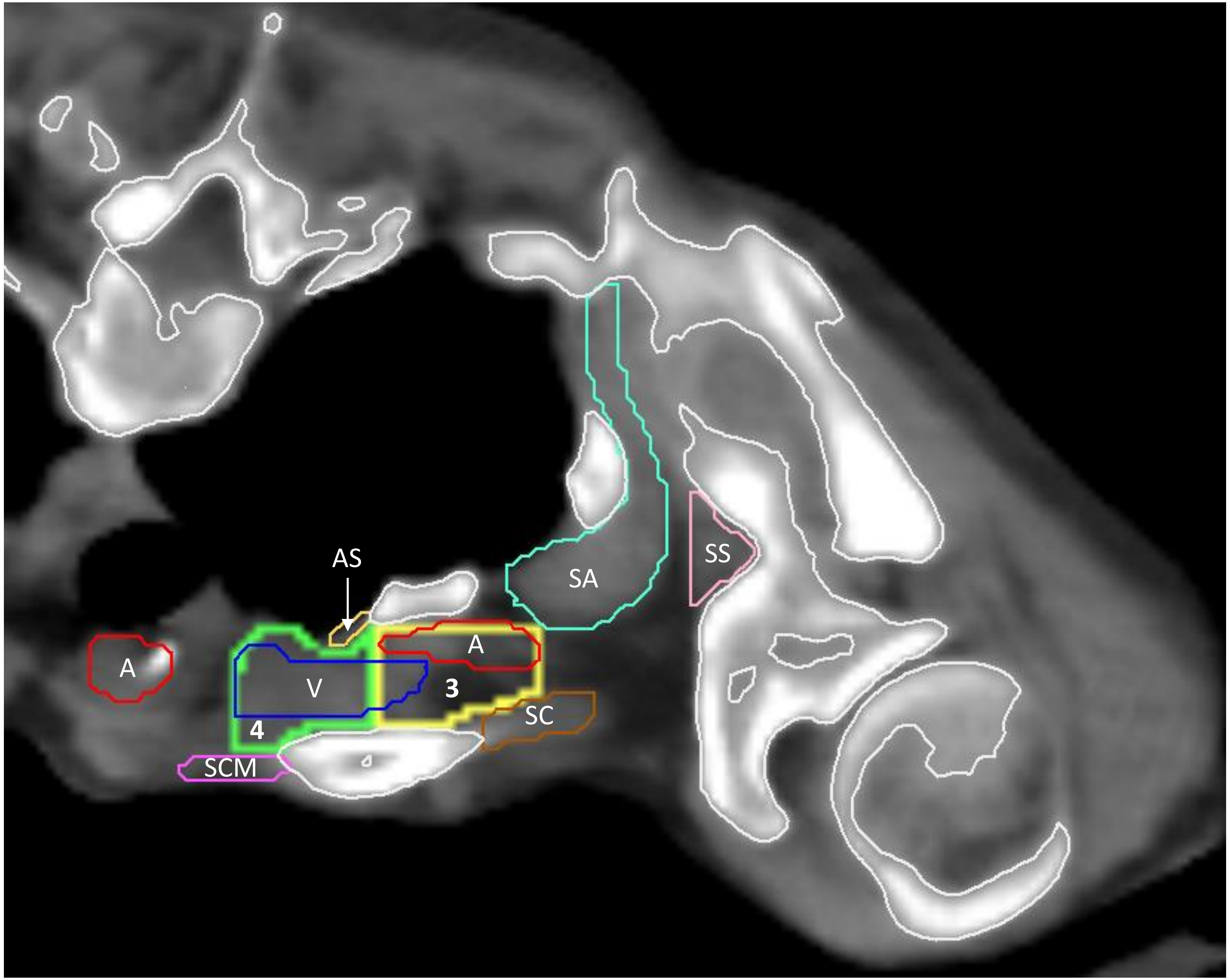
AS
SA
SS
A
A
V
3
4
SC
SCM

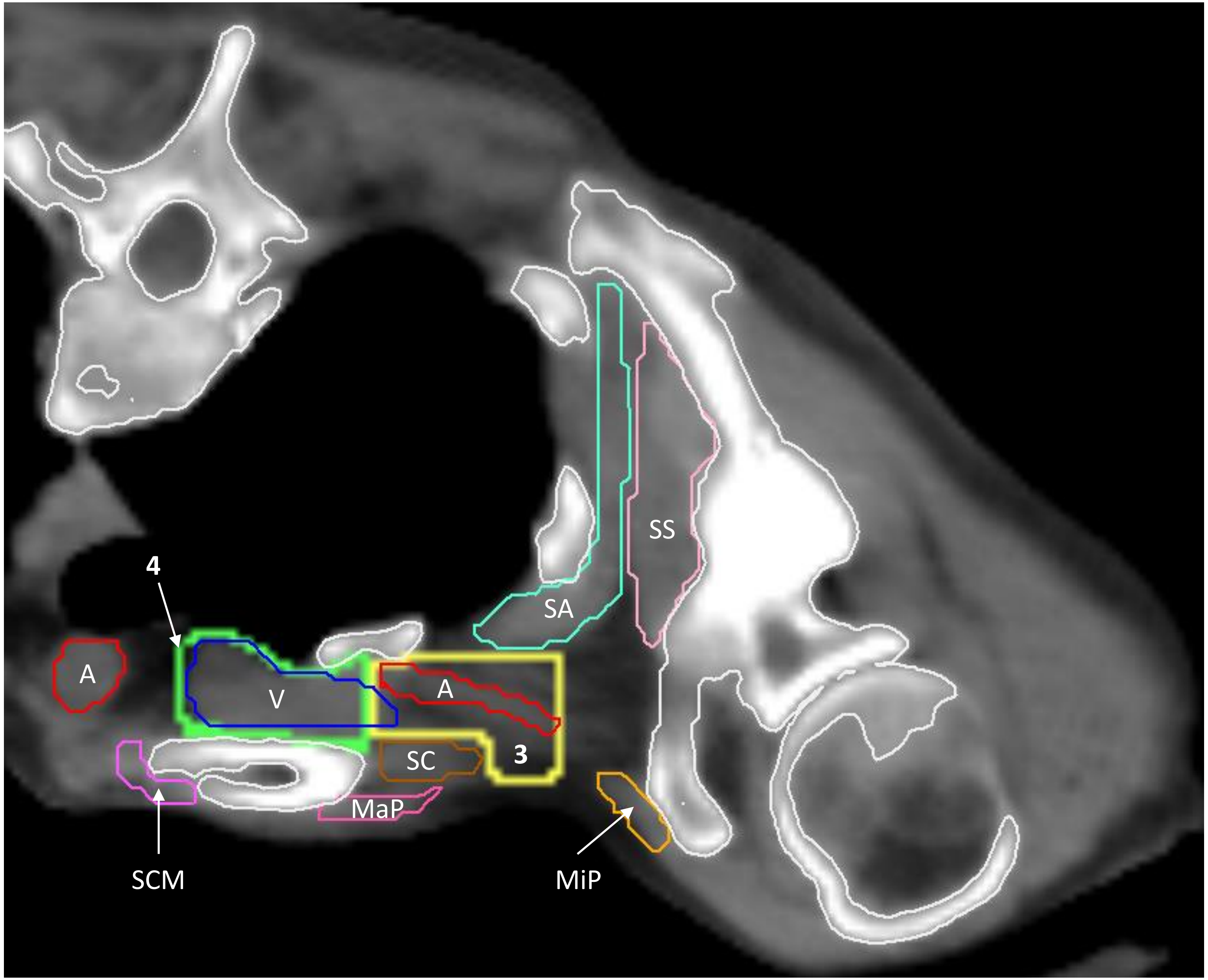
4
SA
SS
A
V
A
3
SC
MaP
SCM
MiP

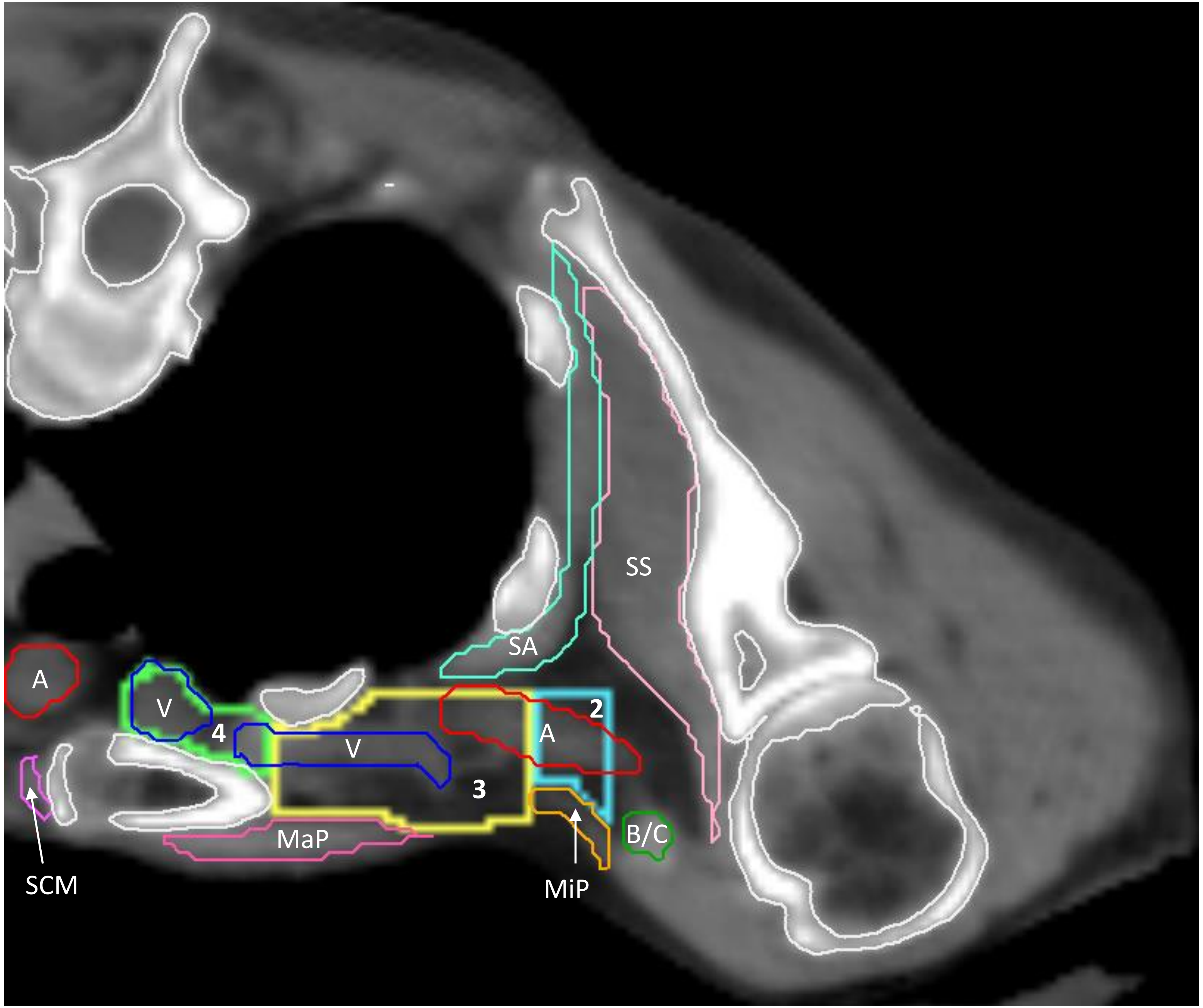
SS
SA
A
V
4
V
A
2
3
MaP
SCM
MiP
B/C

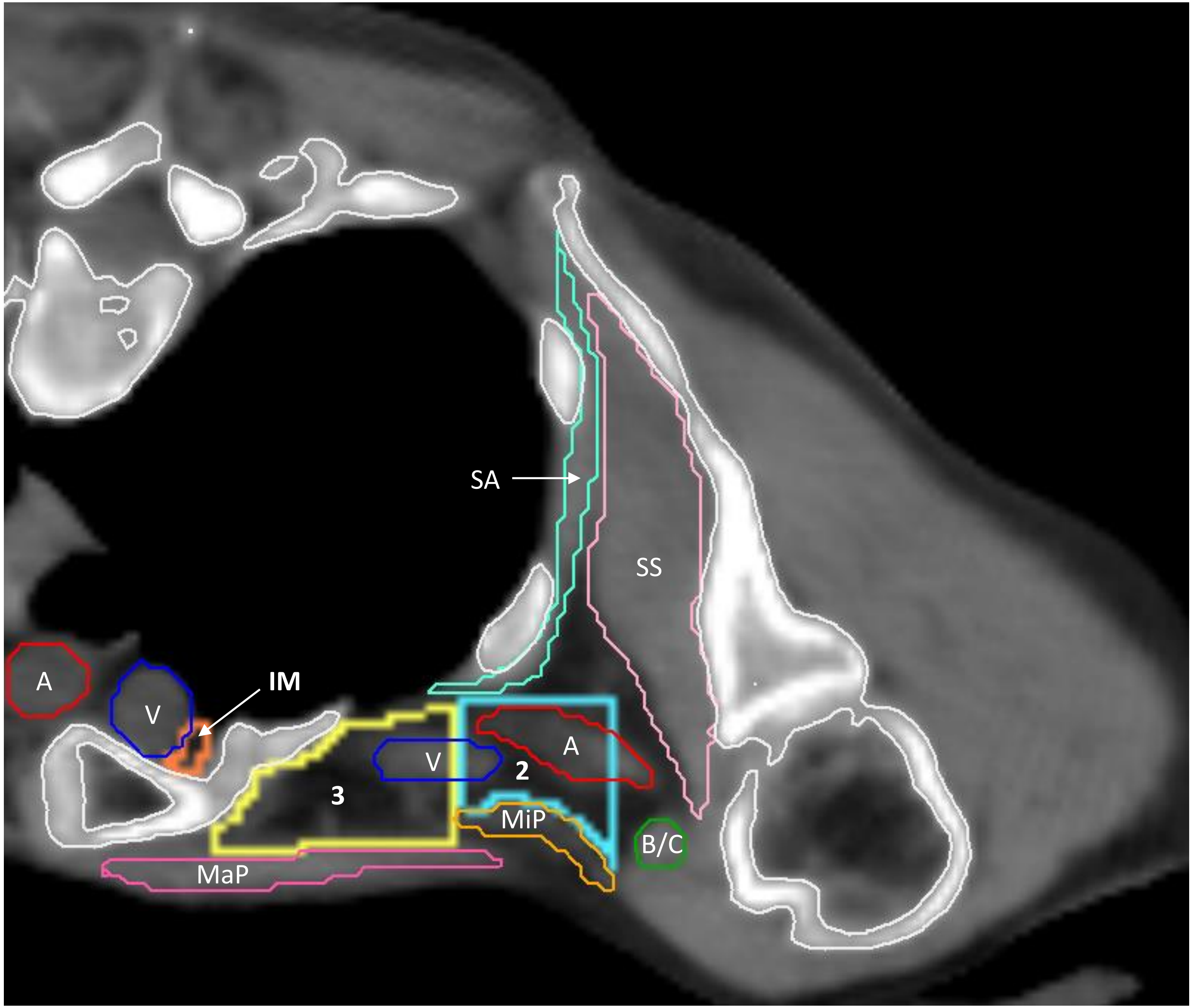
SA
SS
A
V
IM
V
A
2
3
MiP
B/C
MaP

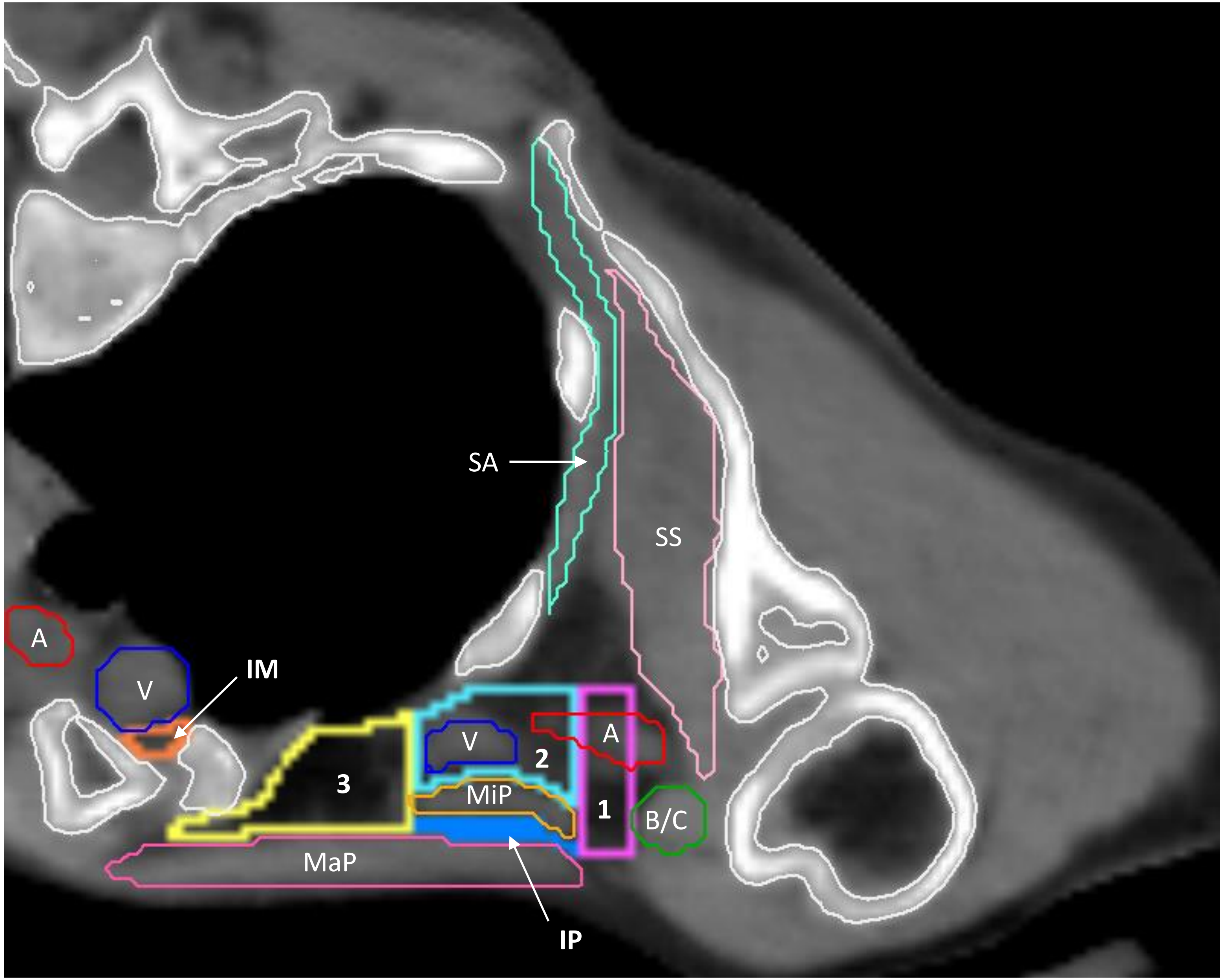

SA
SS
A
V
IM
3
V
2
A
MiP
1
B/C
MaP
IP

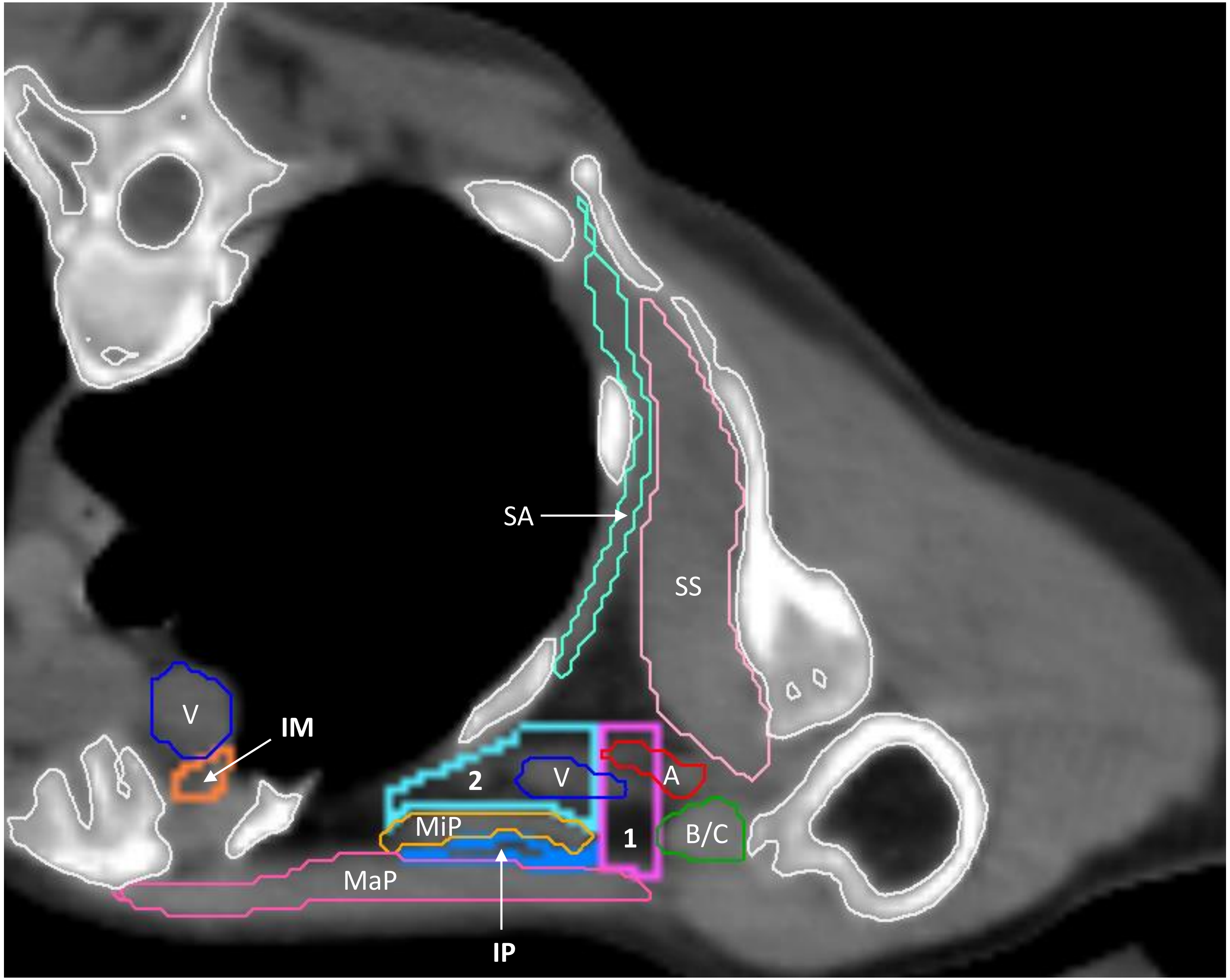
SA
SS
V
IM
2
V
A
1
B/C
MiP
MaP
IP

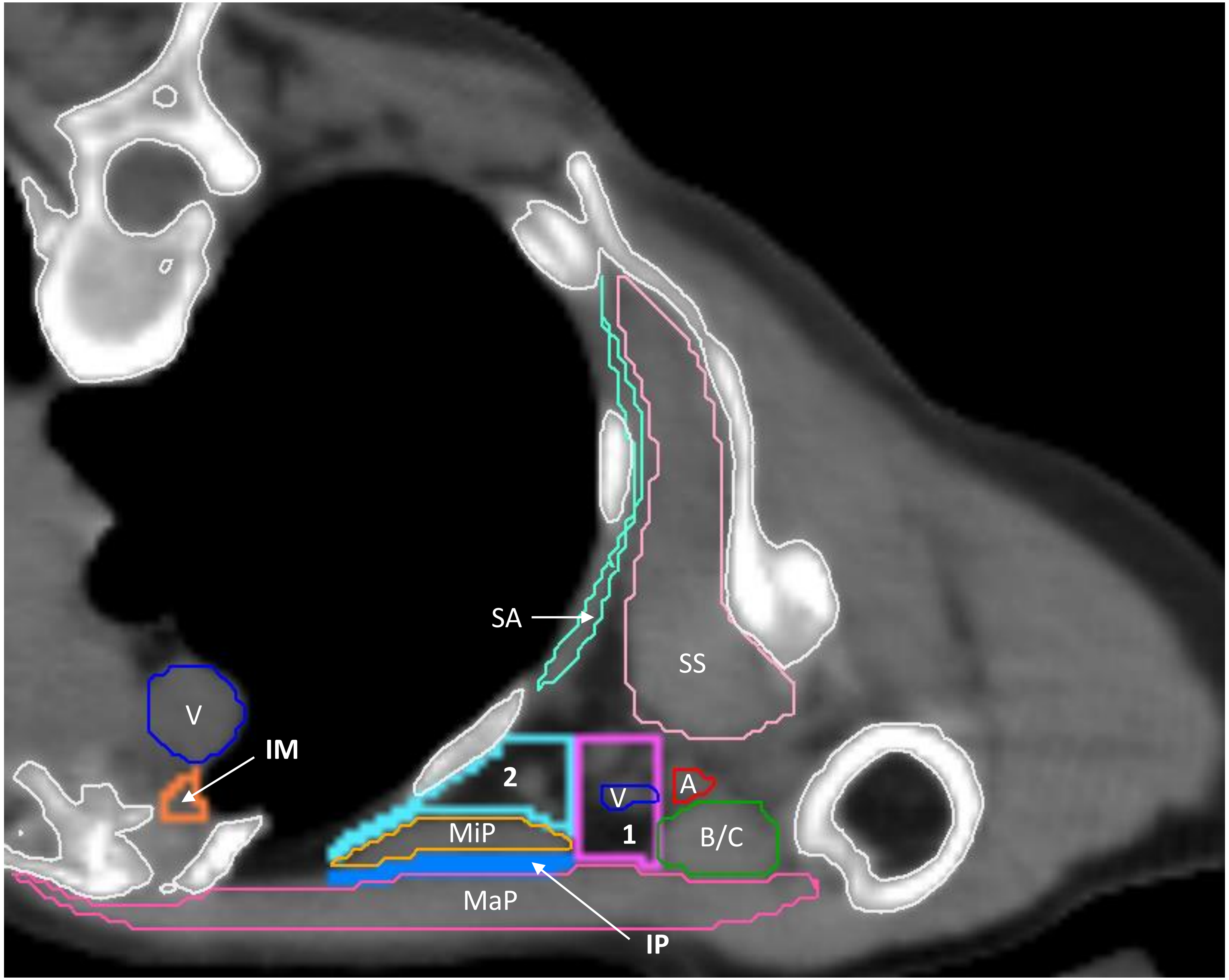
SA
SS
V
IM
2
V
A
MiP
1
B/C
MaP
IP

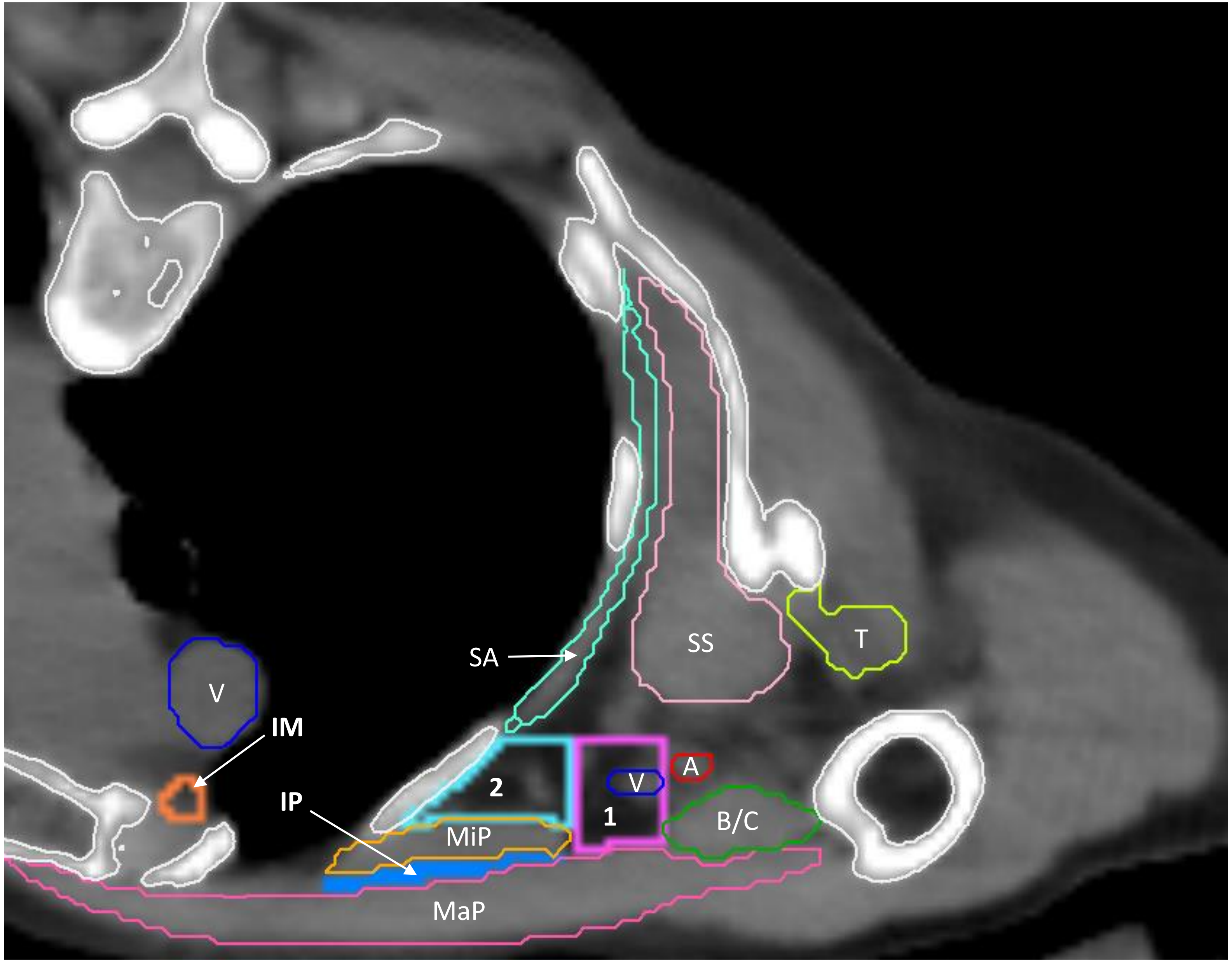
SA
SS
T
V
IM
A
V
2
1
B/C
IP
MiP
MaP

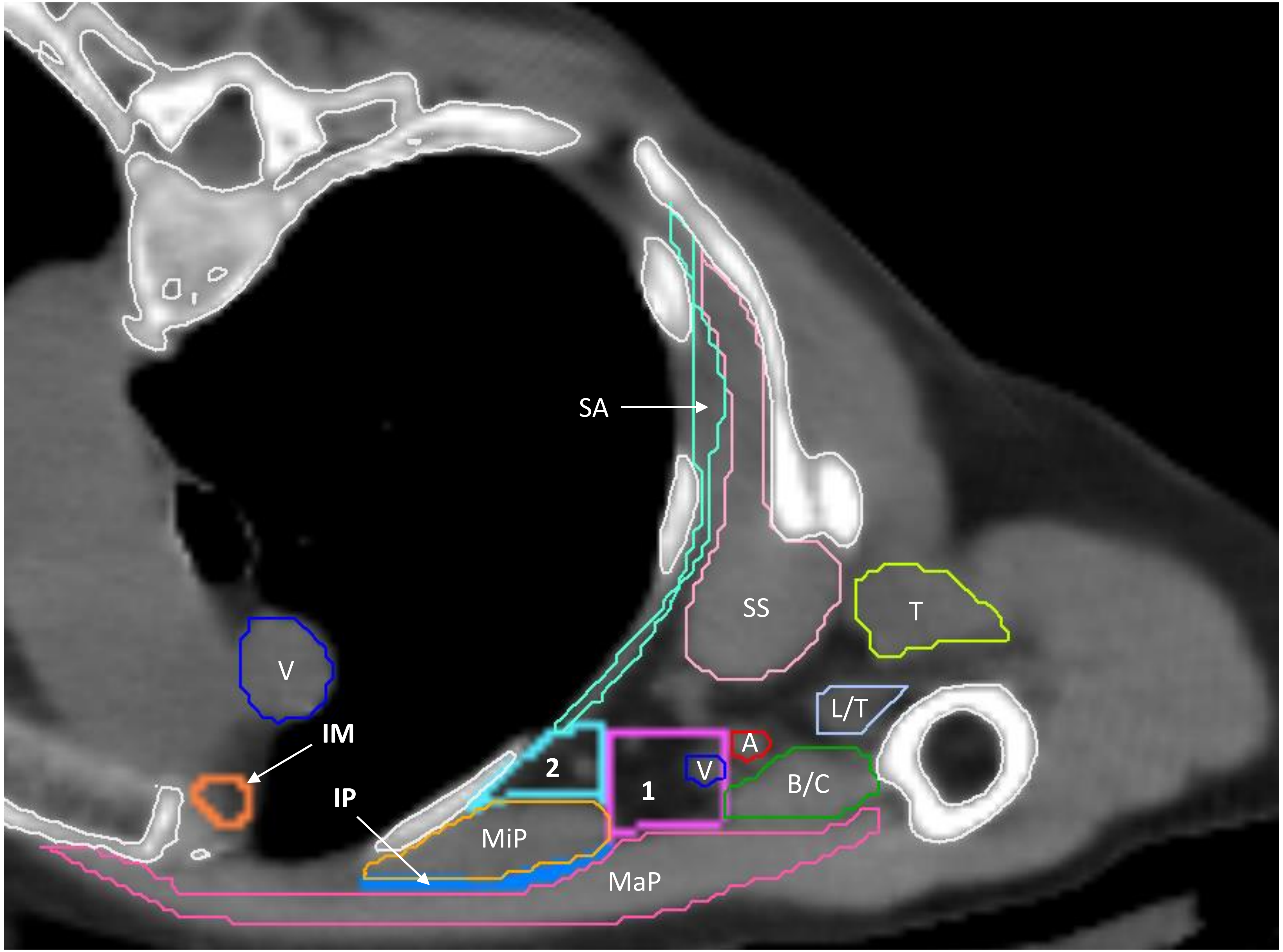
SA
SS
T
V
L/T
IM
A
2
V
B/C
1
IP
MiP
MaP

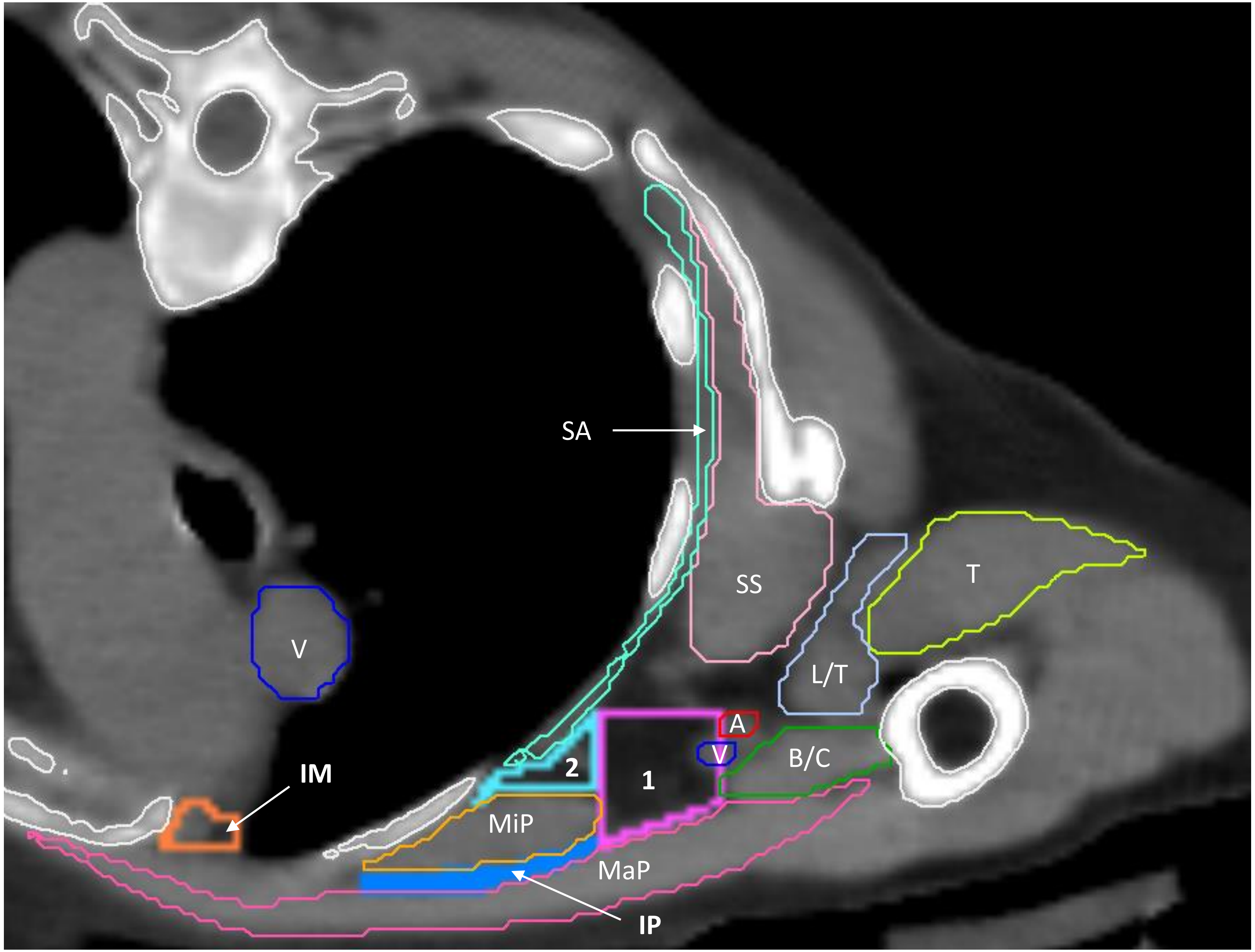
SA
SS
T
L/T
V
A
V
B/C
2
1
IM
MiP
MaP
IP

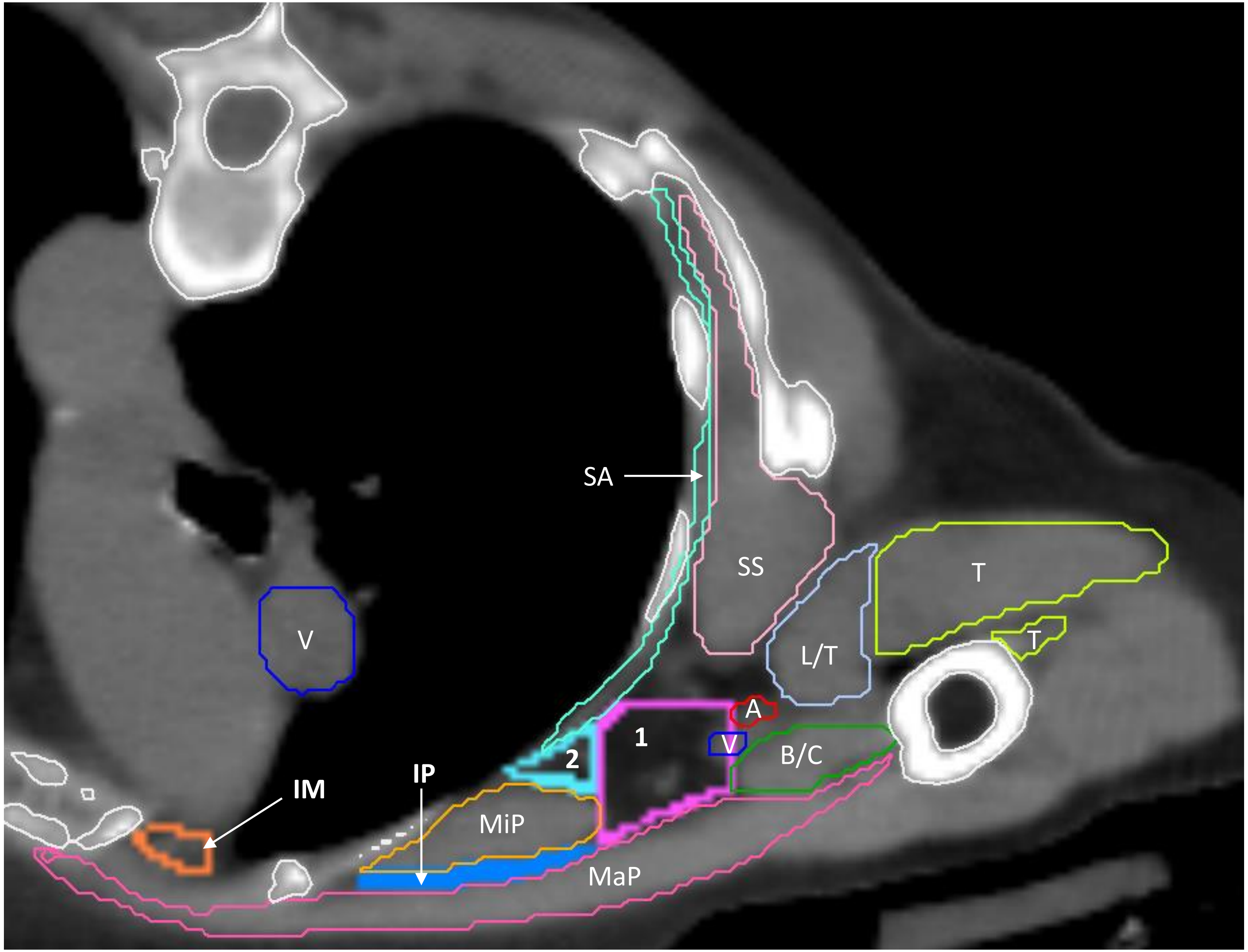
SA
SS
T
T
L/T
V
A
1
V
2
B/C
IM
IP
MiP
MaP

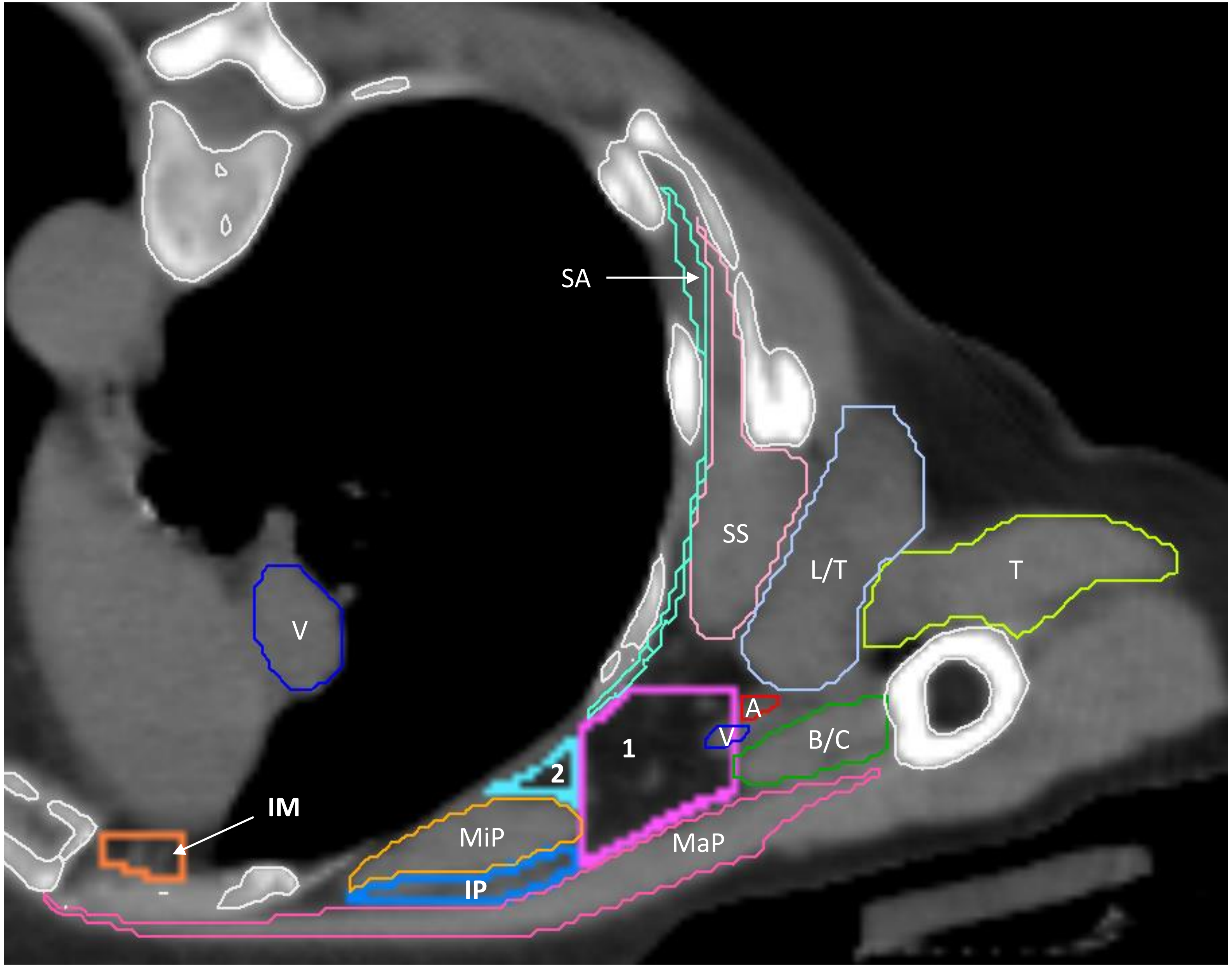
SA
SS
L/T
T
V
A
V
1
2
B/C
IM
MiP
MaP
IP

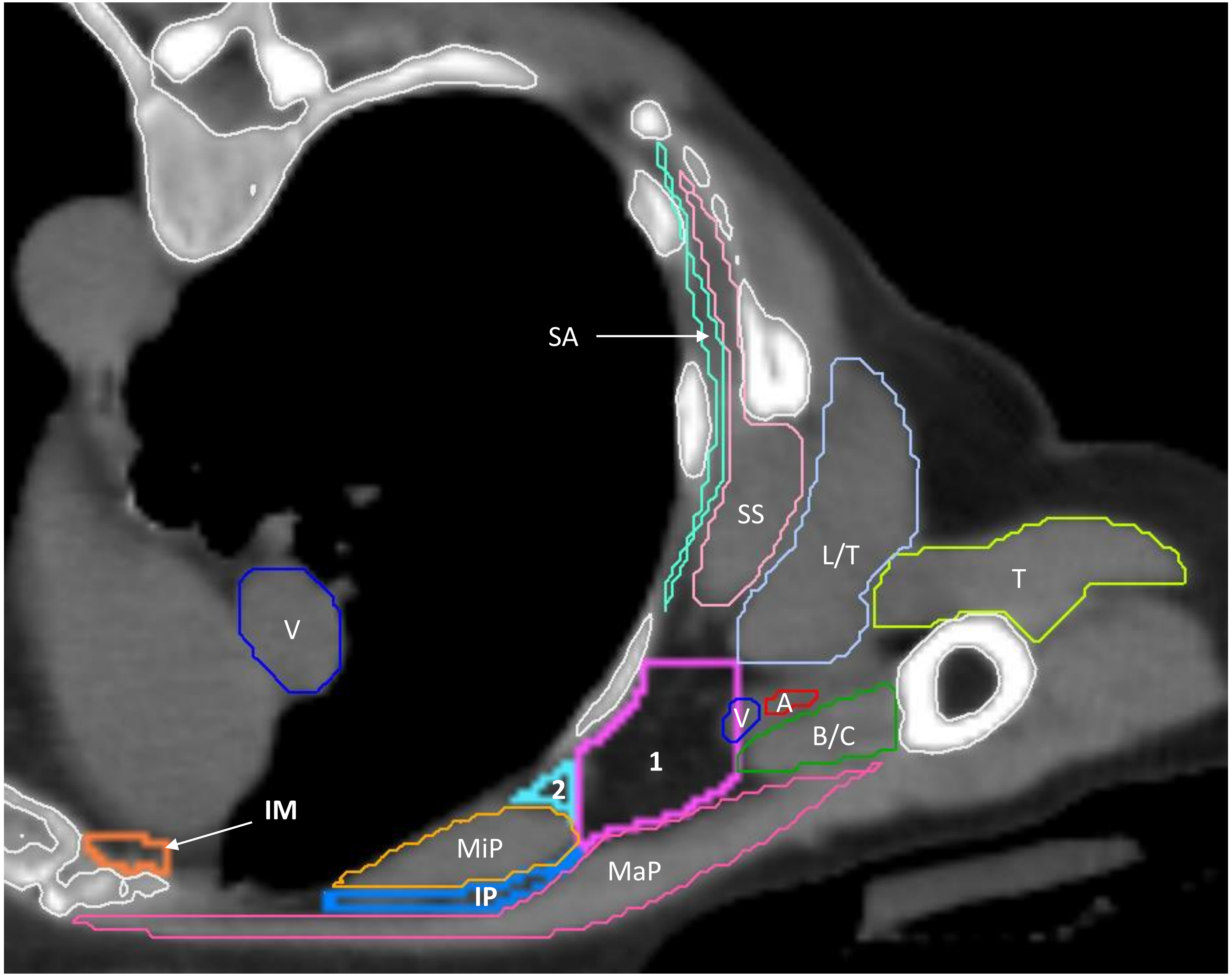
SA
SS
L/T
T
V
V
A
B/C
1
2
IM
MiP
MaP
IP

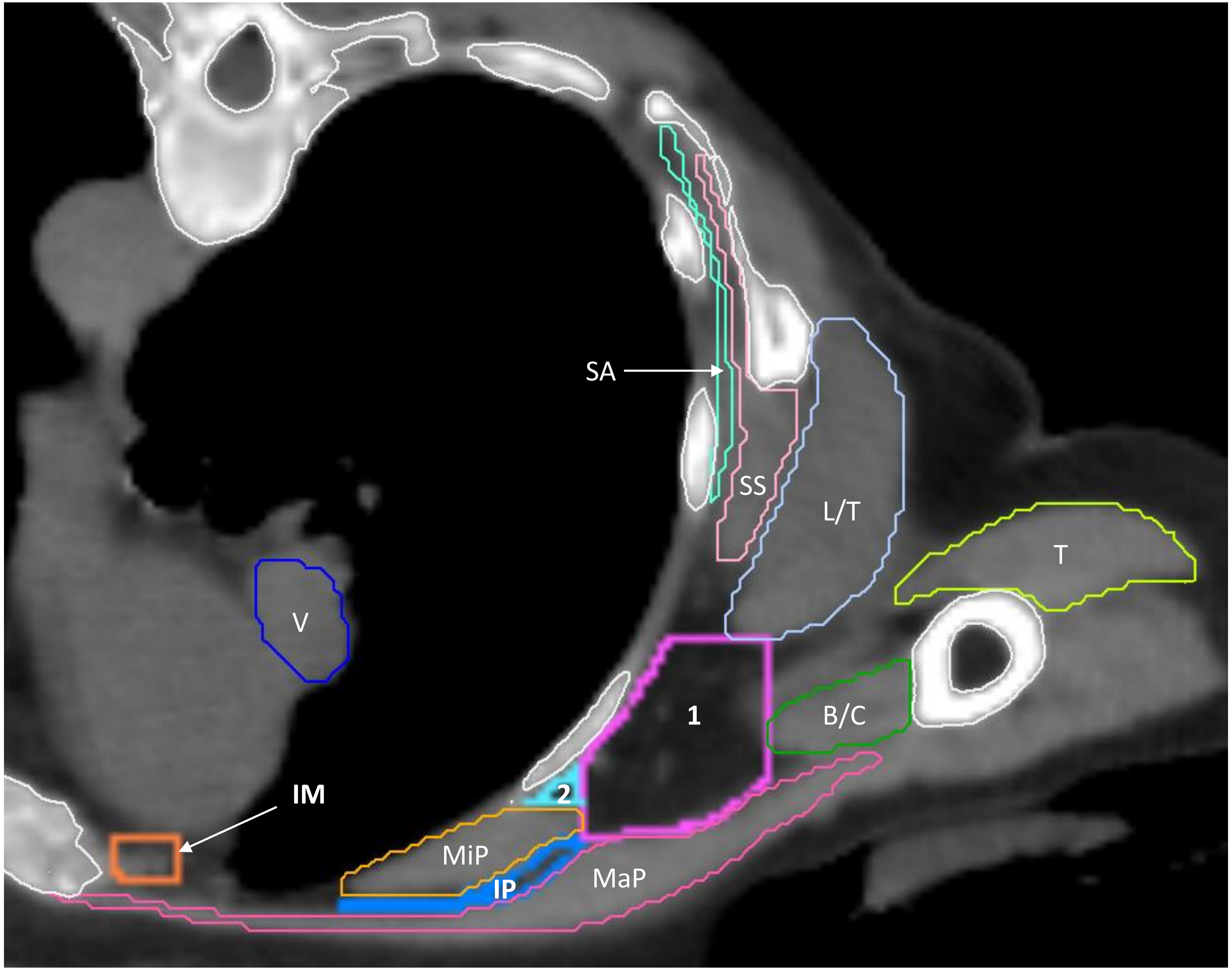

SA
SS
L/T
T
V
1
B/C
2
IM
MiP
IP
MaP

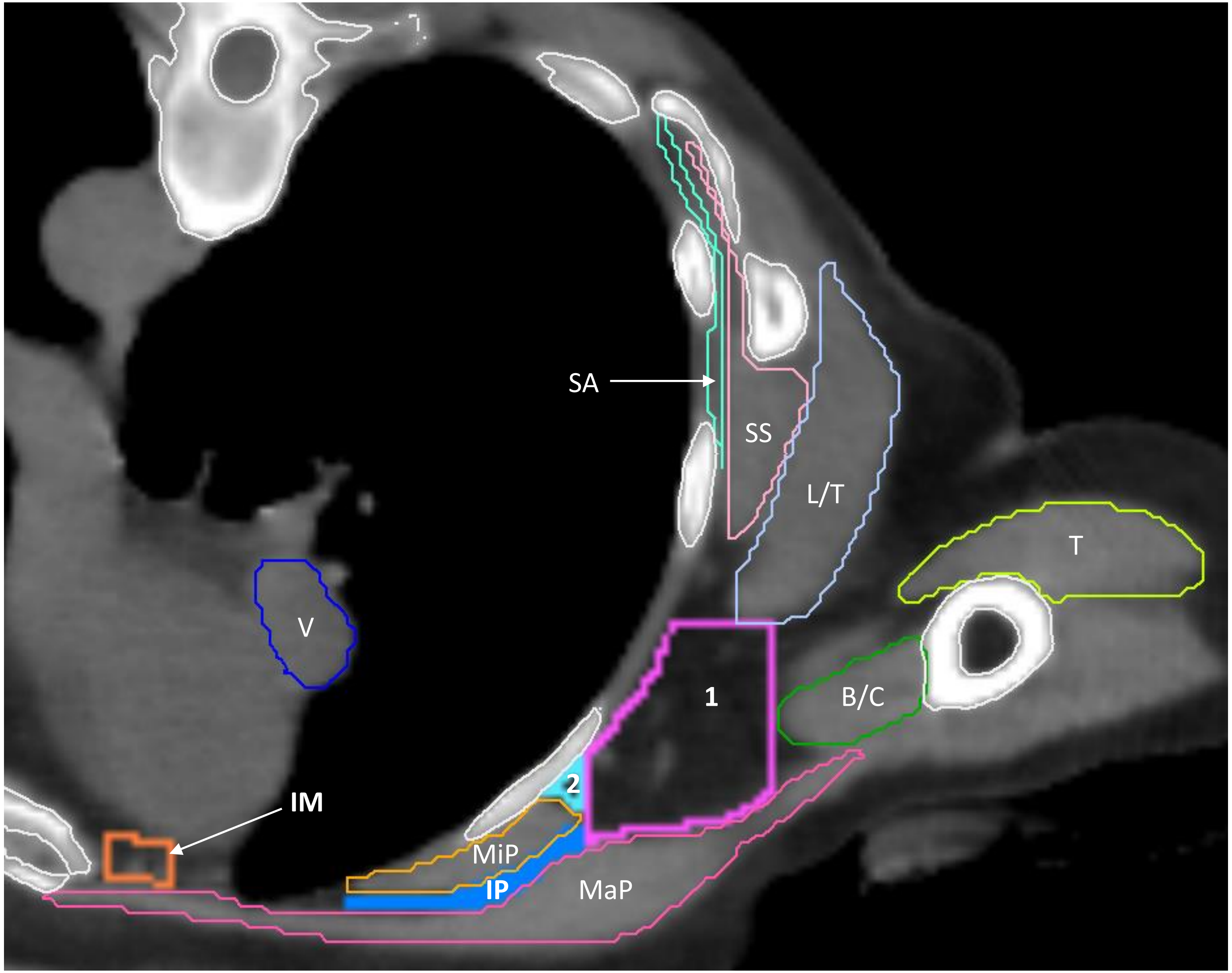
SA
SS
L/T
T
V
1
B/C
2
IM
MiP
IP
MaP

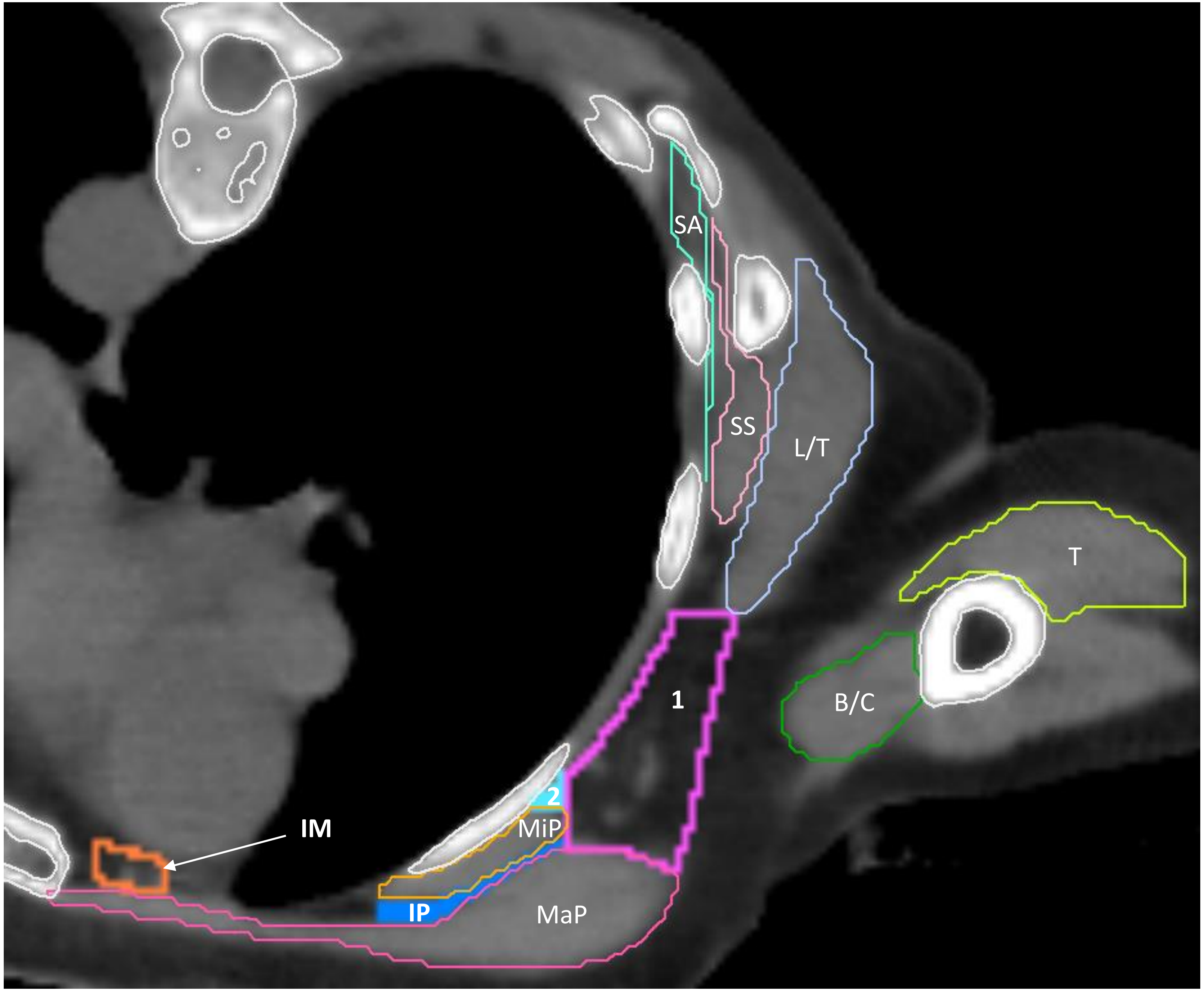
SA
SS
L/T
T
B/C
1
2
MiP
IM
IP
MaP

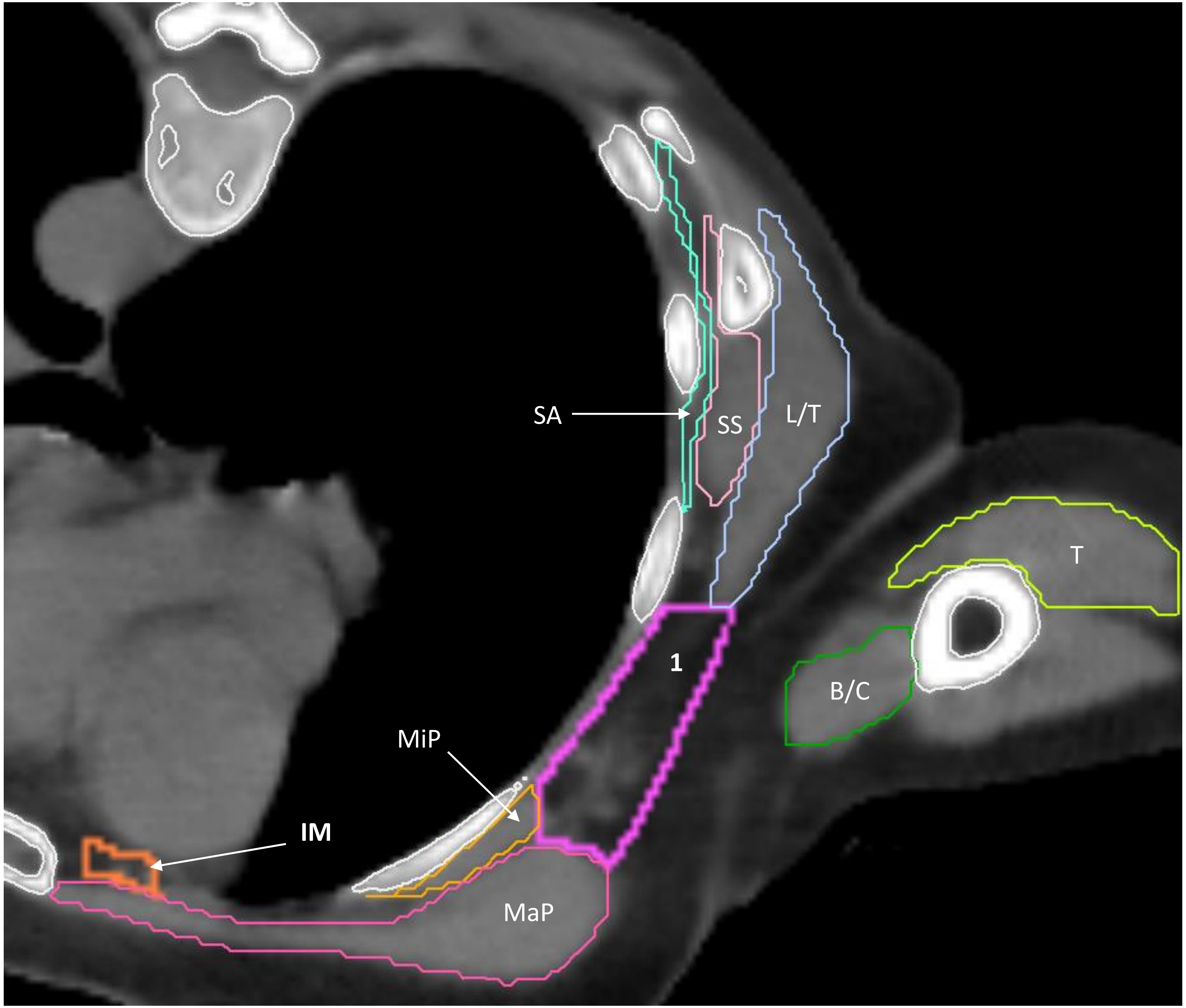
SA
SS
L/T
T
1
B/C
MiP
IM
MaP

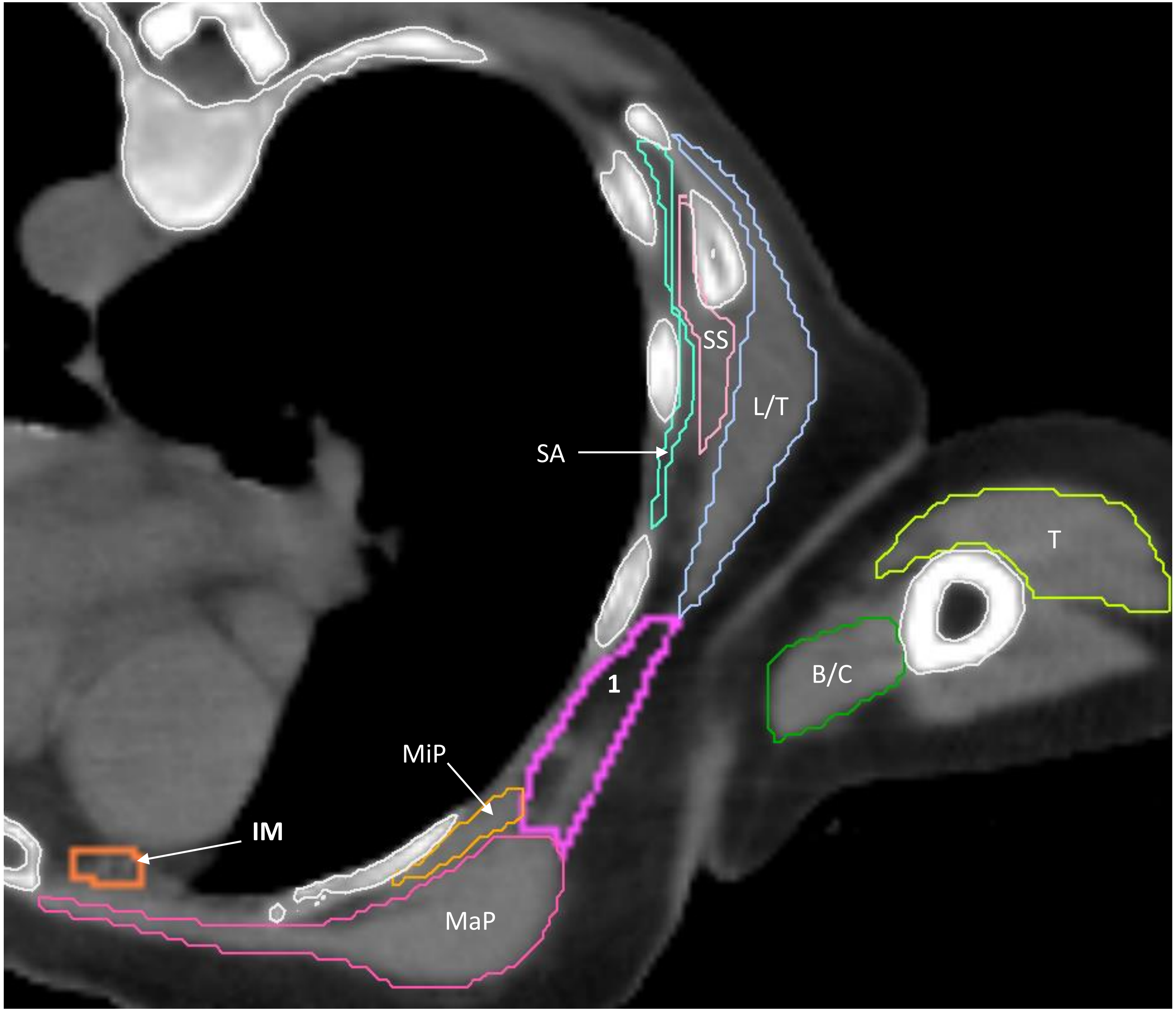

SS
L/T
SA
T
B/C
1
MiP
IM
MaP

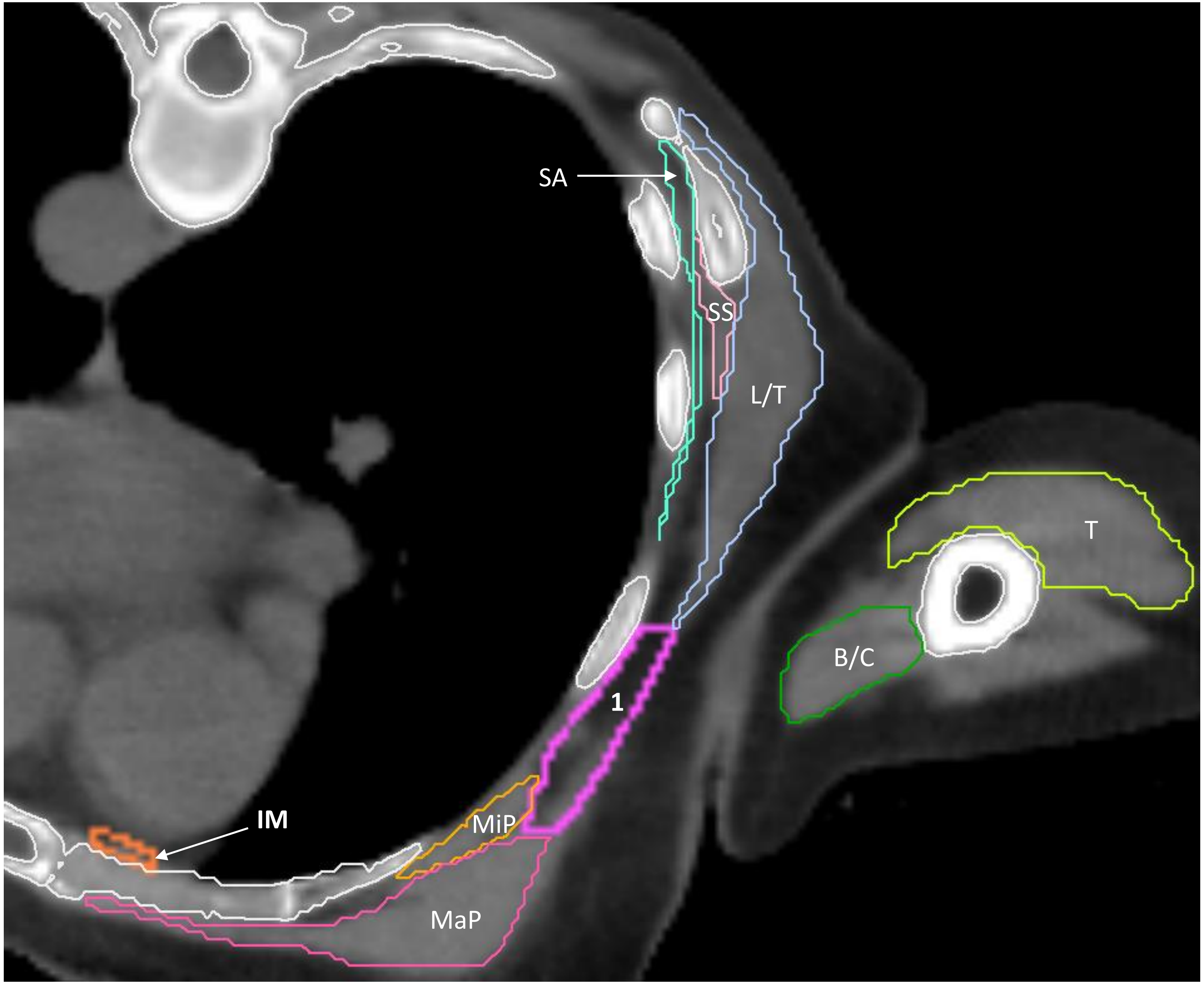
SA
SS
L/T
T
B/C
1
IM
MiP
MaP

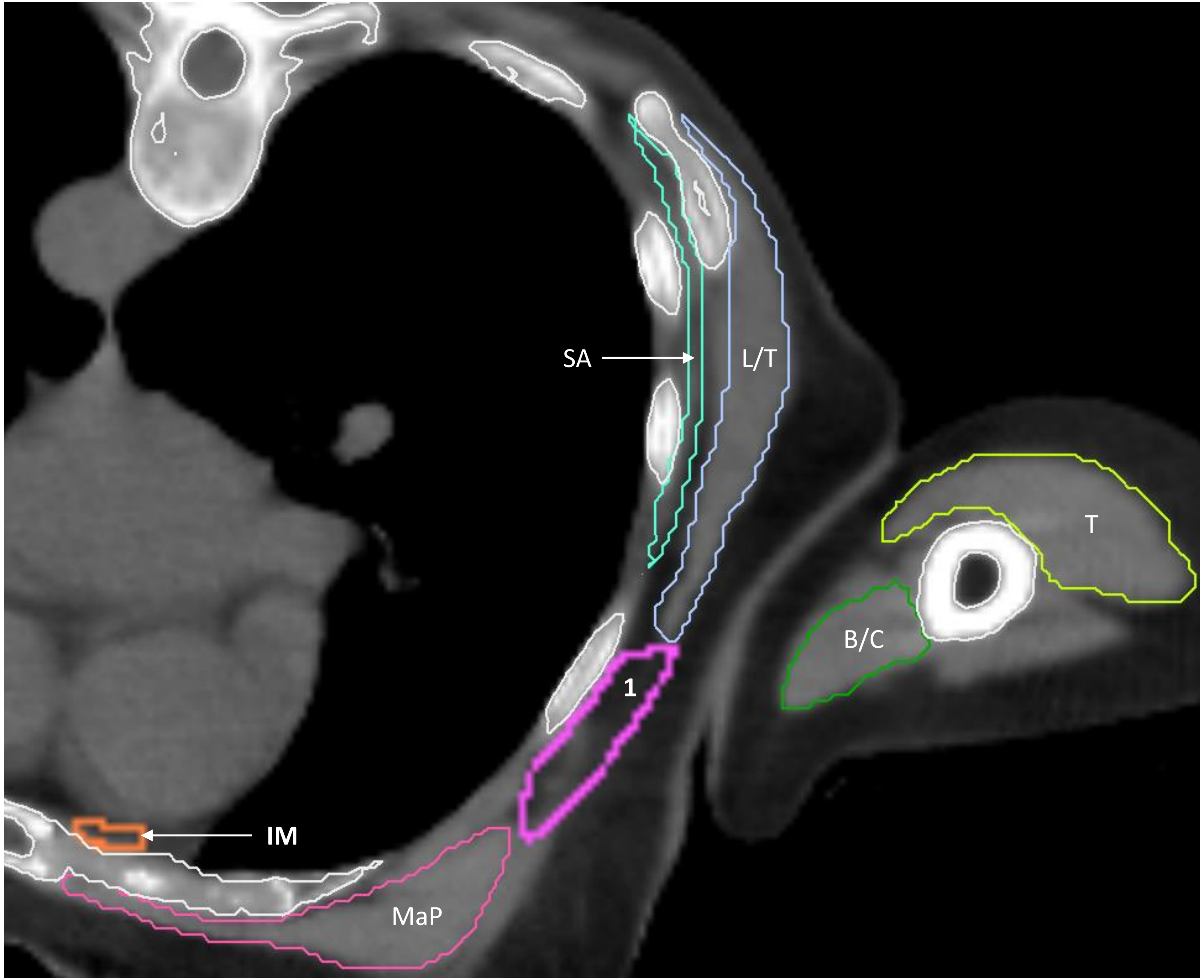
SA
L/T
T
B/C
1
IM
MaP

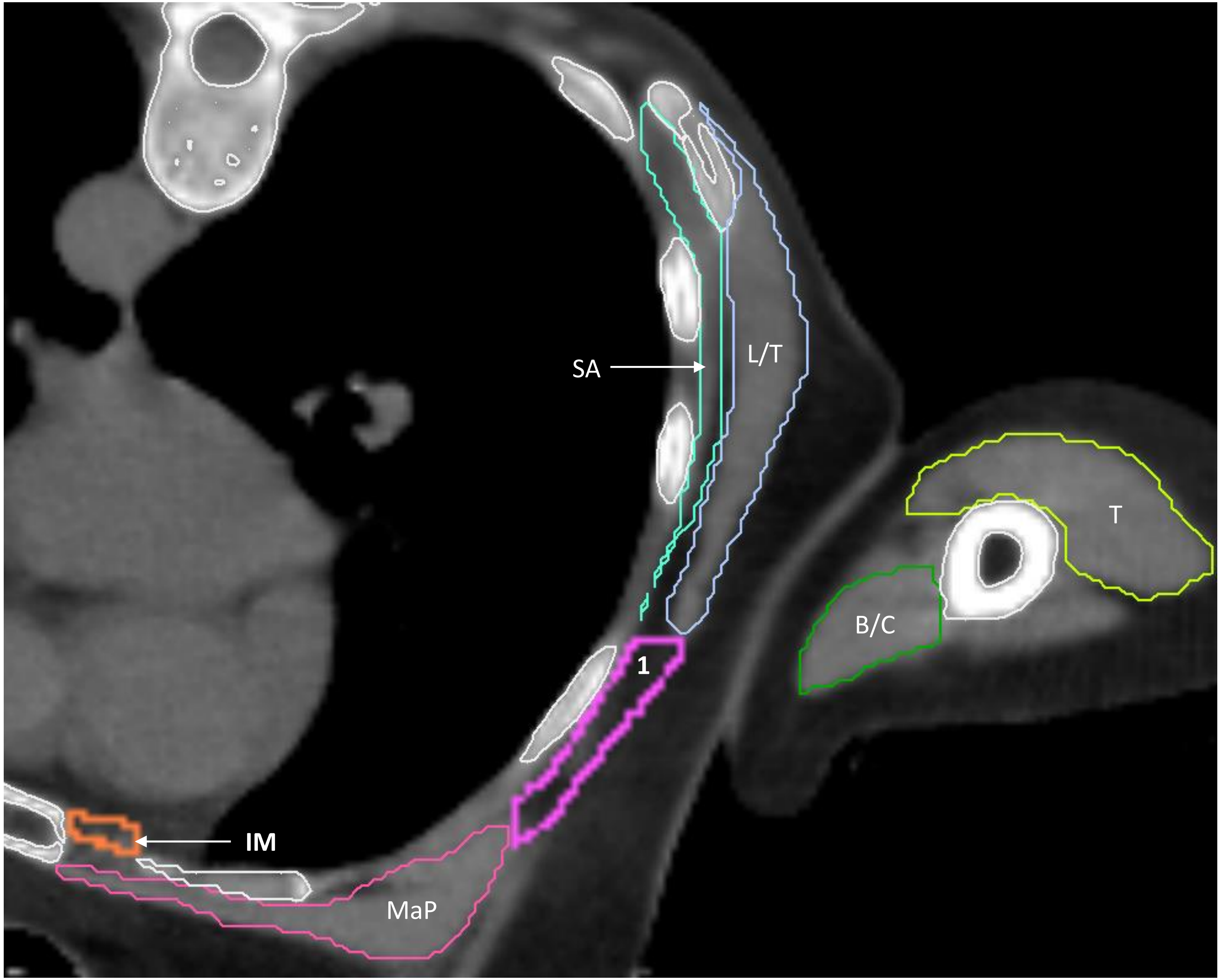

SA
L/T
T
B/C
1
IM
MaP

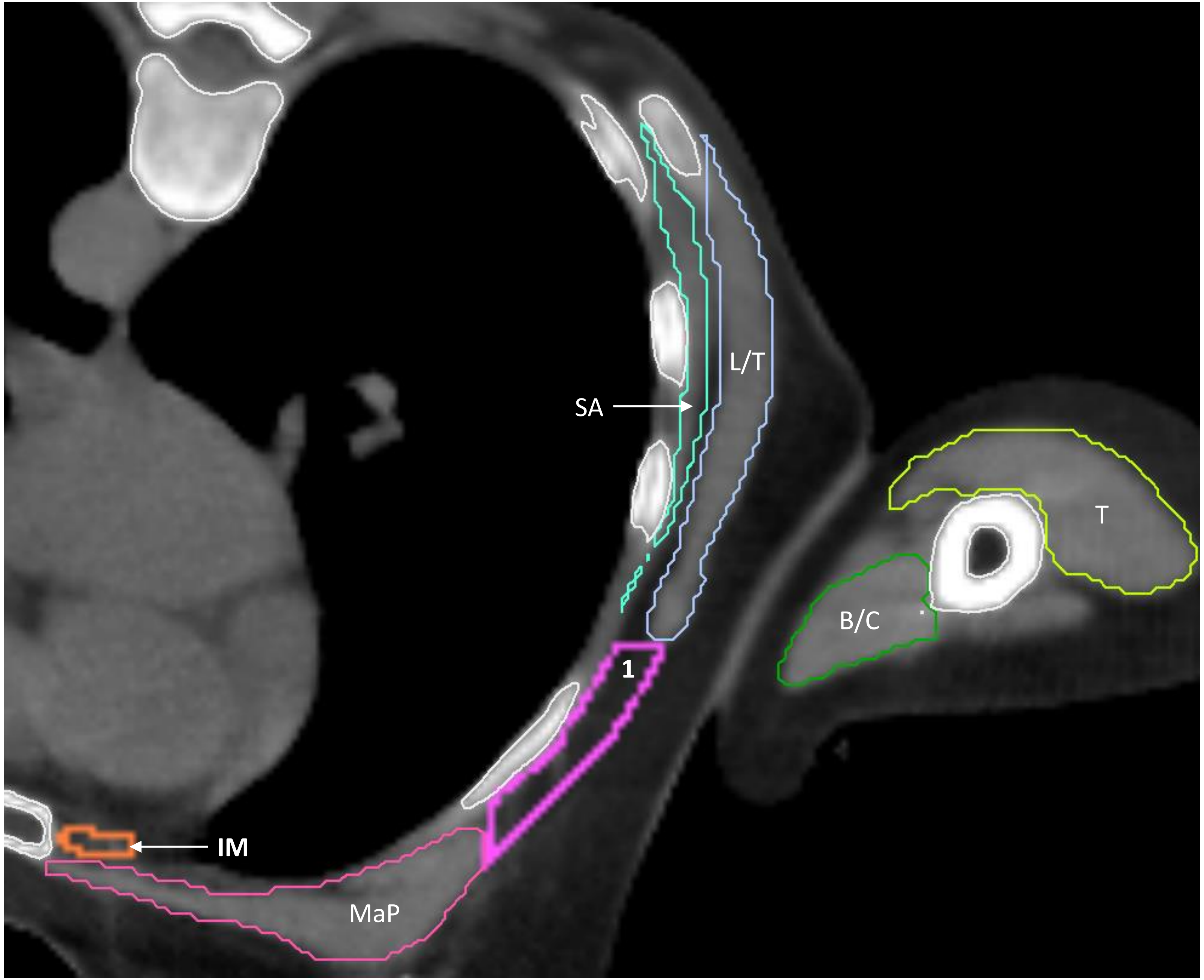
L/T
SA
T
B/C
1
IM
MaP

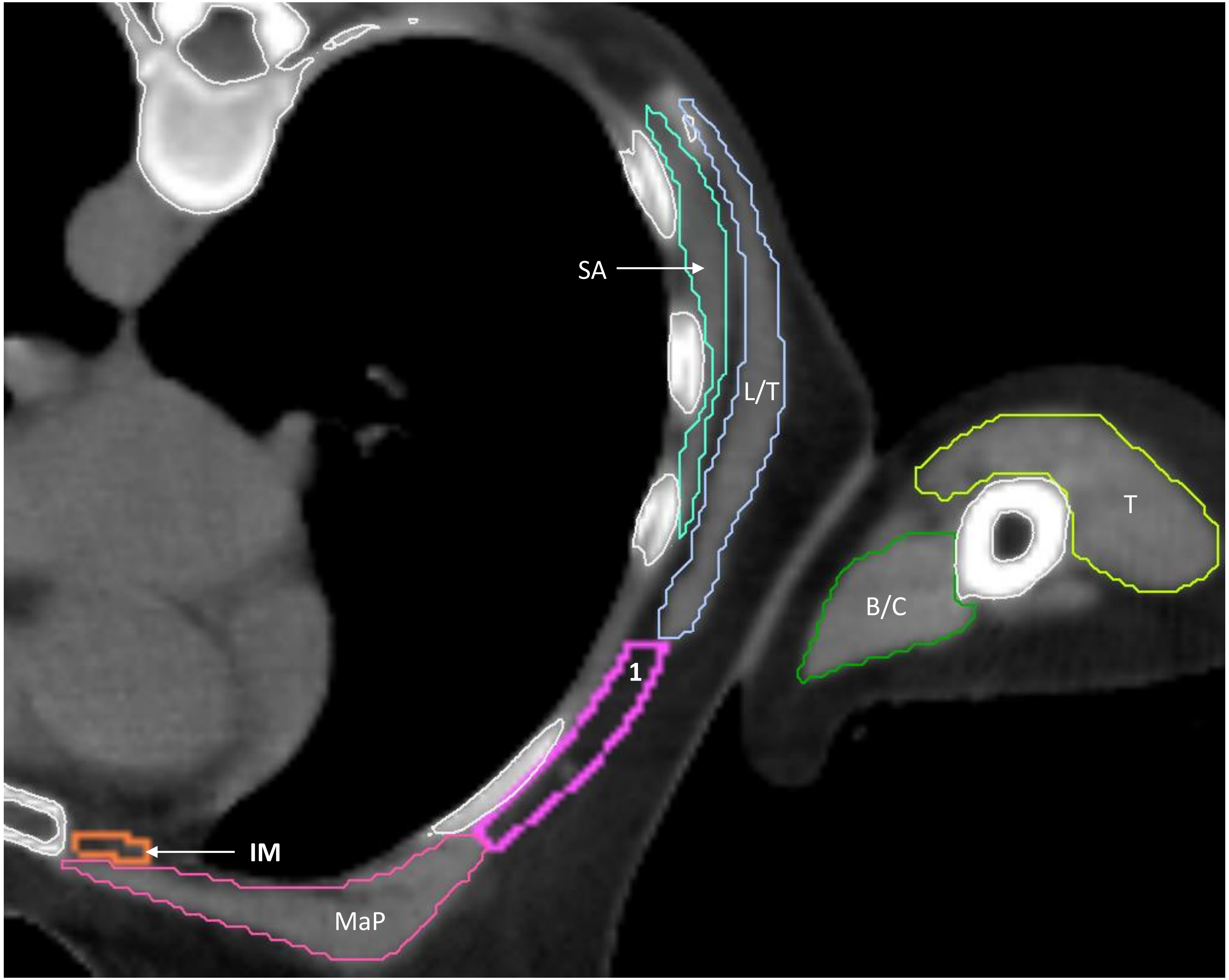
SA
L/T
T
B/C
1
IM
MaP

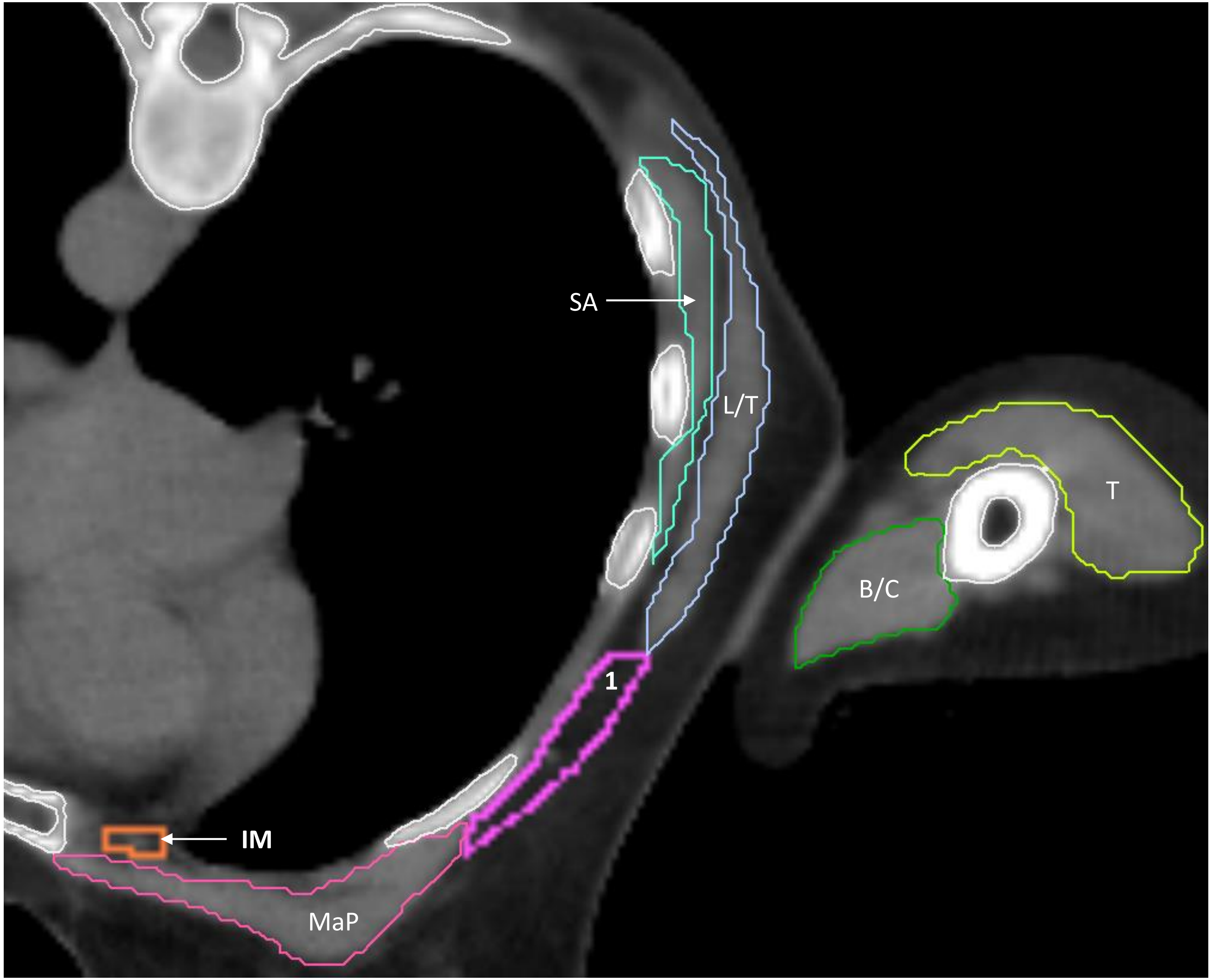

SA
L/T
T
B/C
1
IM
MaP

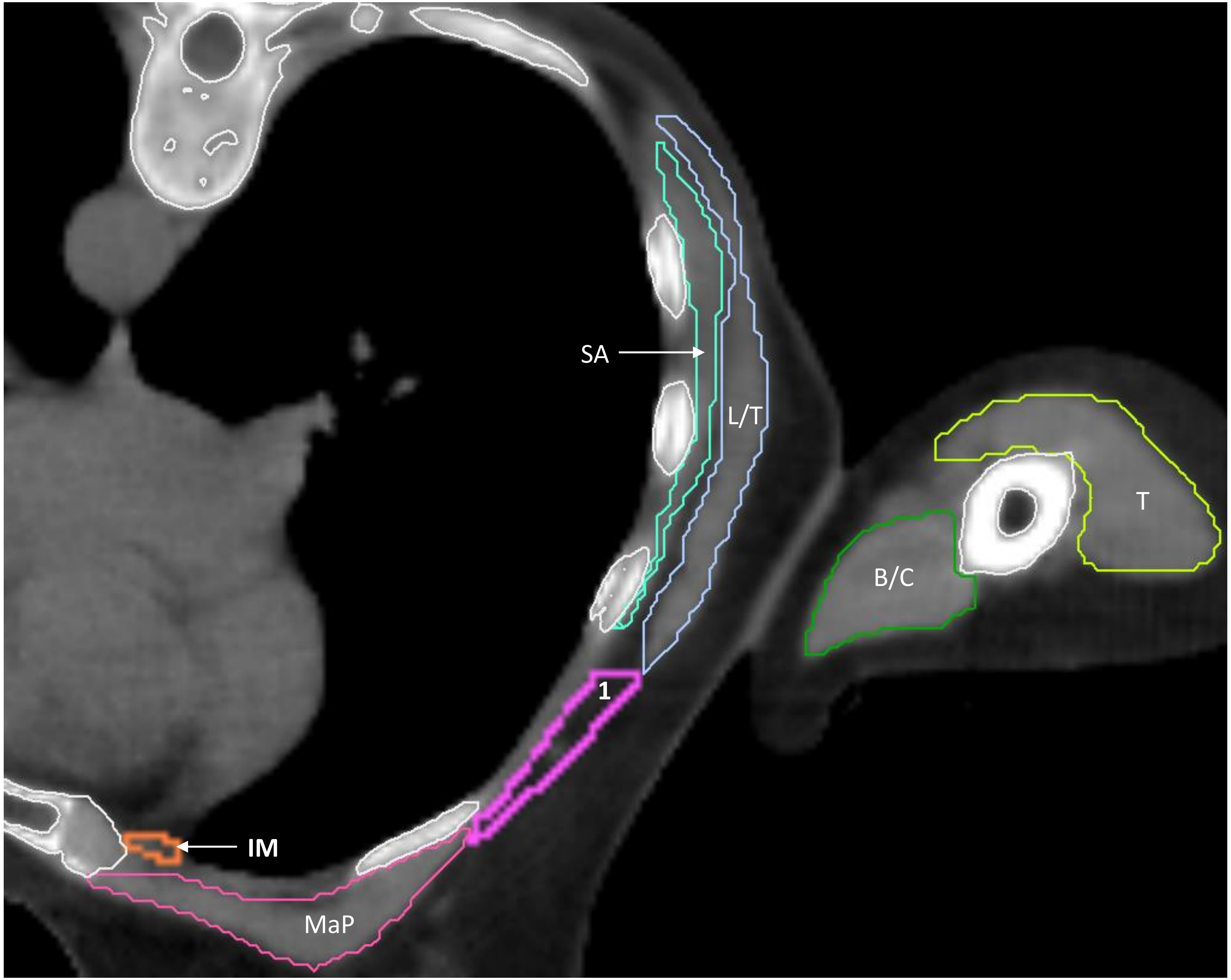
SA
L/T
T
B/C
1
IM
MaP

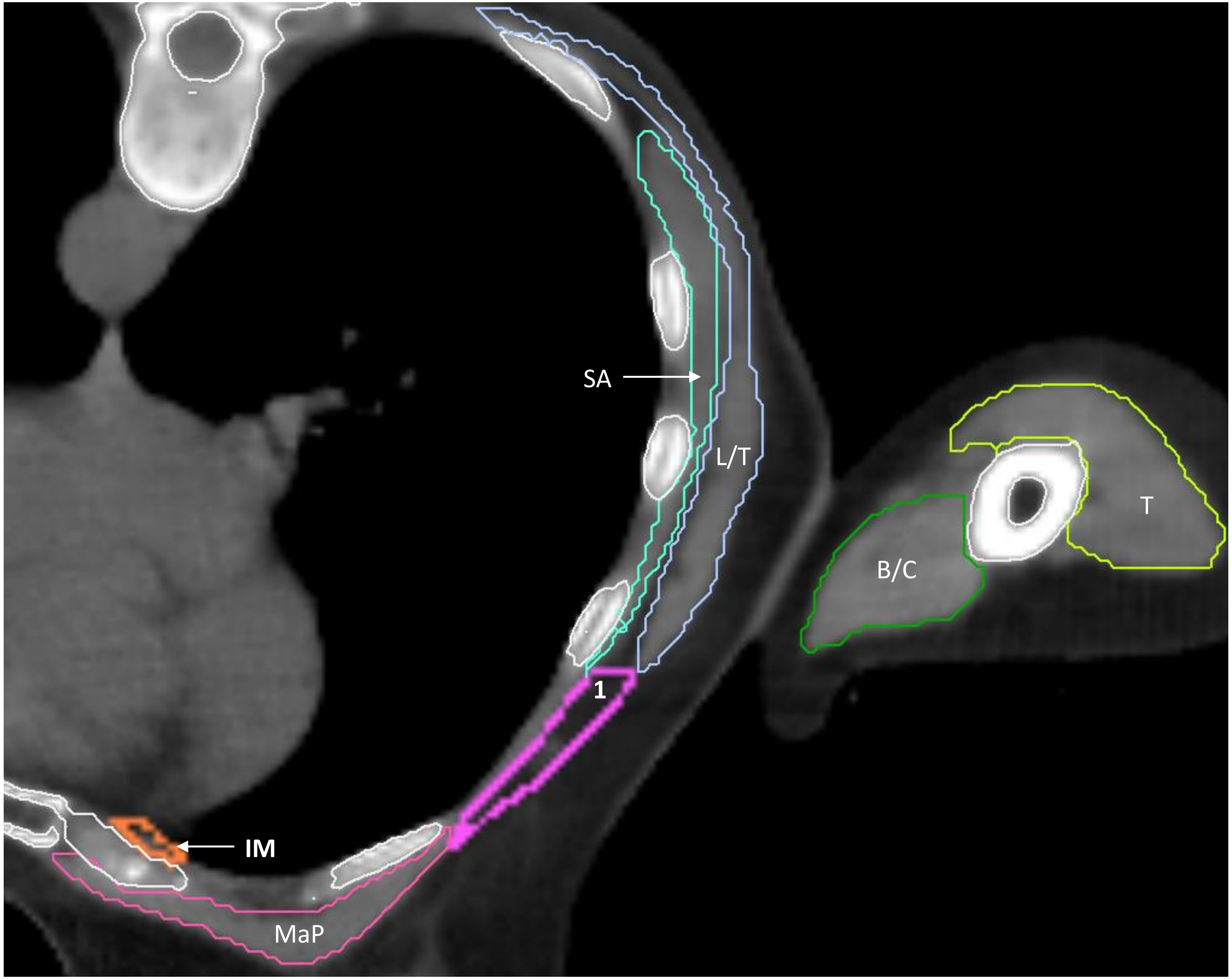
SA
L/T
T
B/C
1
IM
MaP

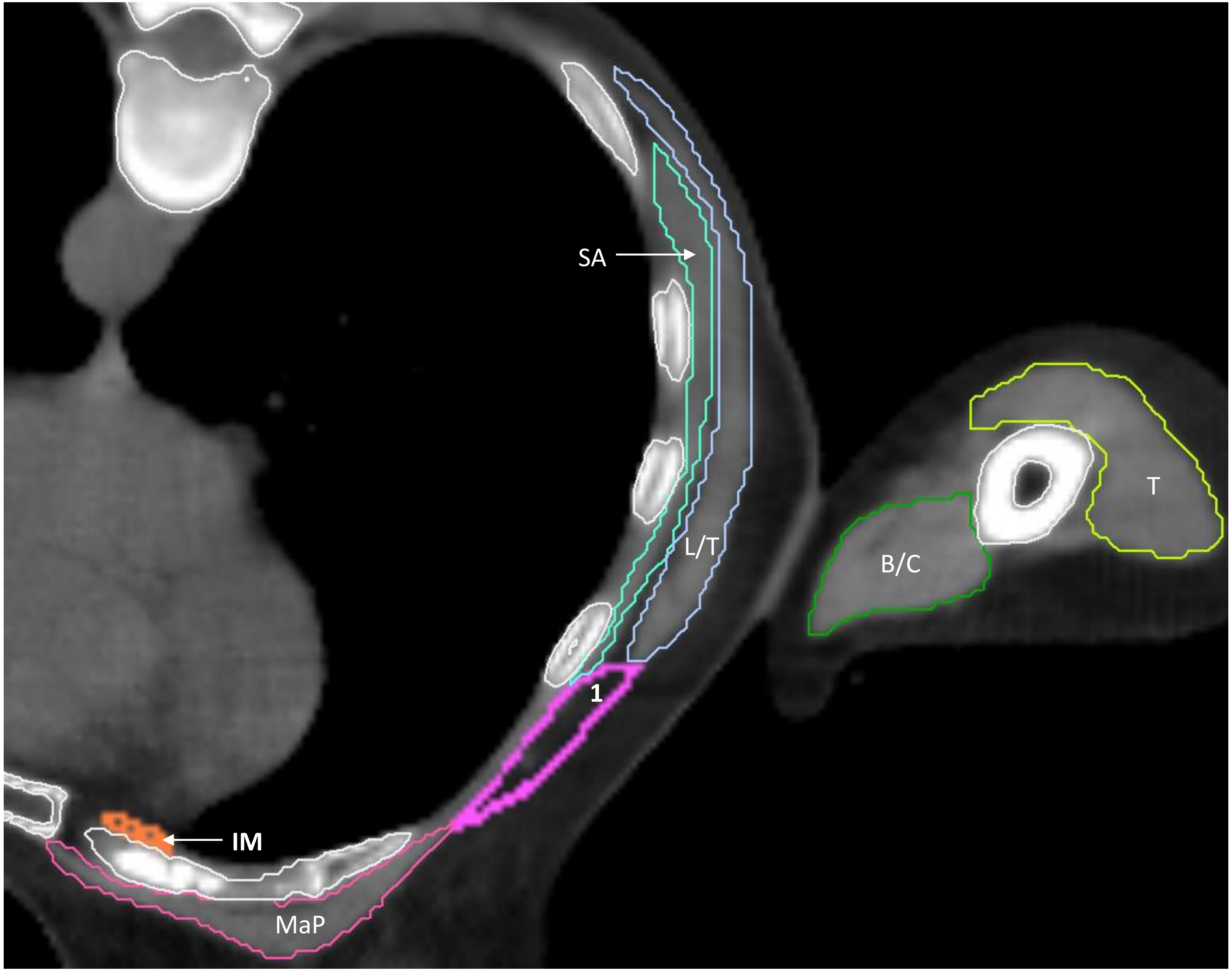
SA
L/T
T
B/C
1
IM
MaP

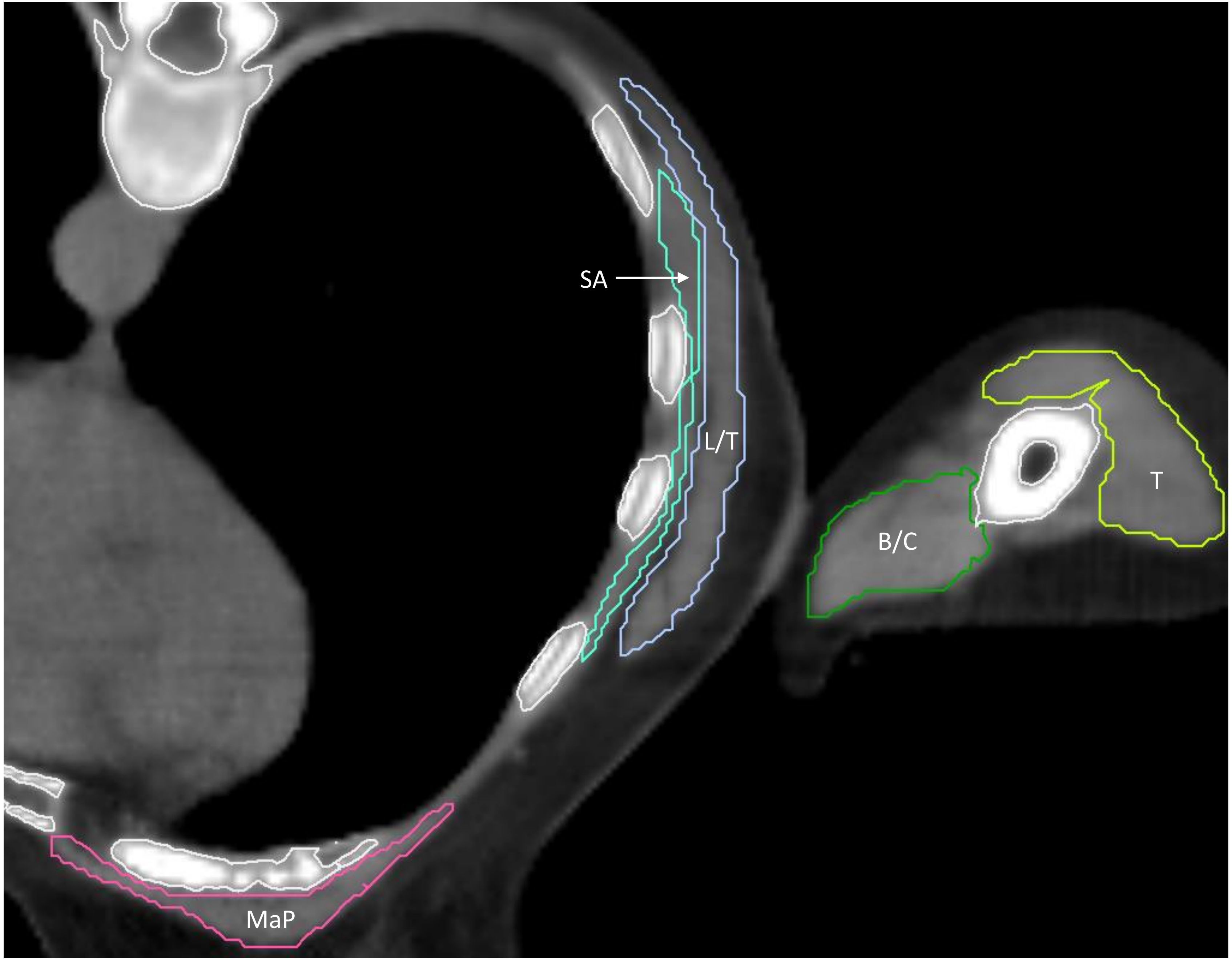
SA
L/T
T
B/C
MaP

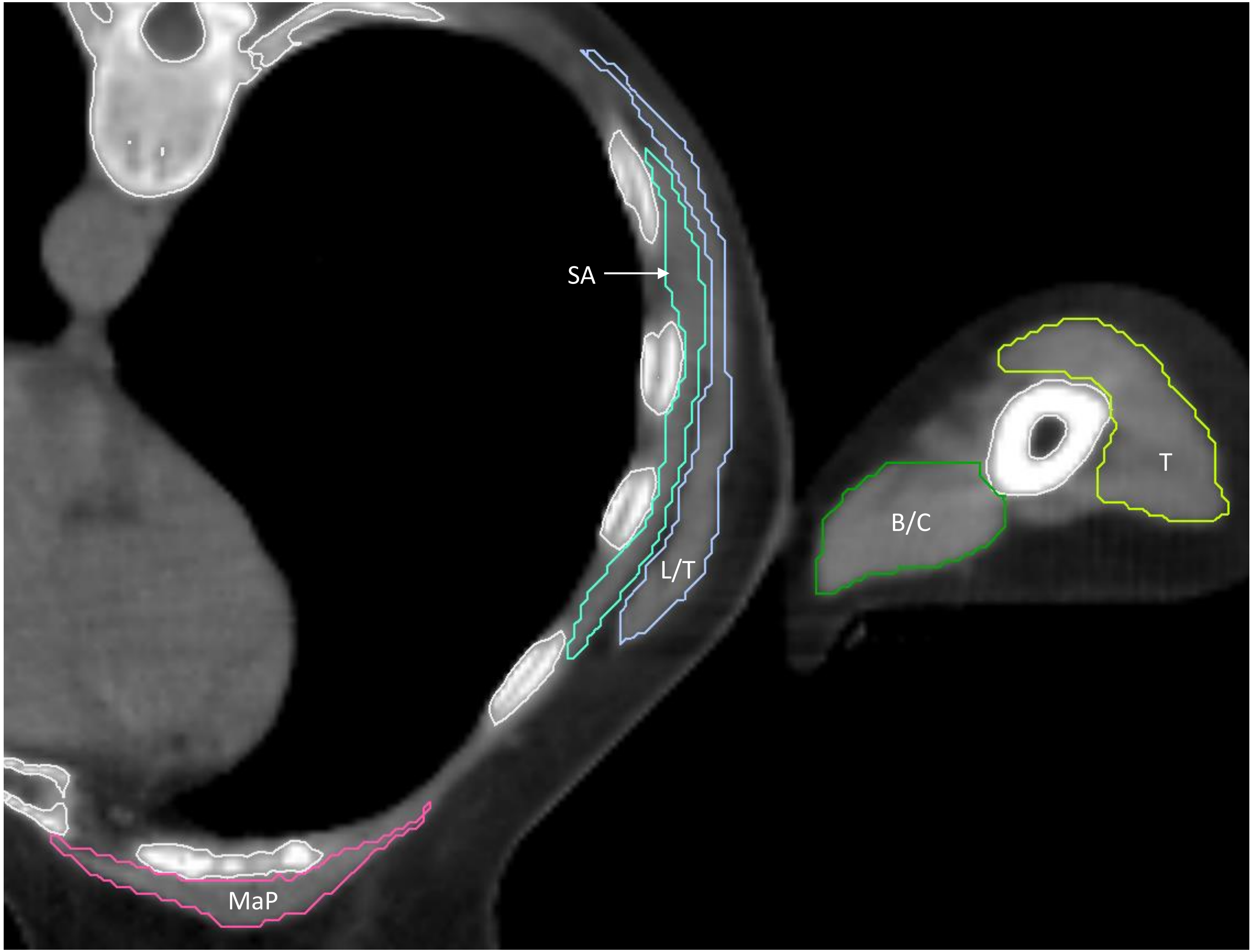
SA
L/T
T
B/C
MaP

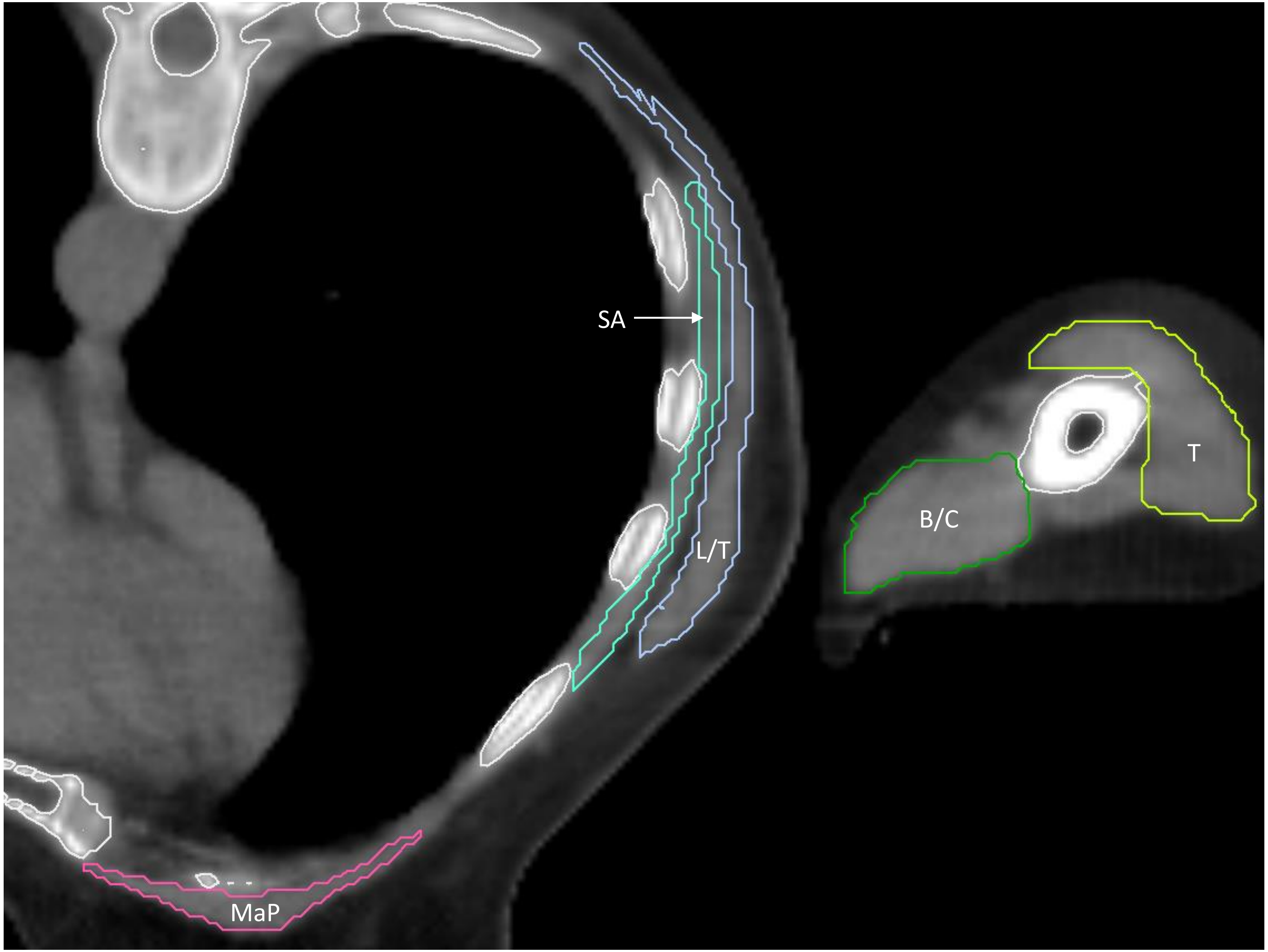
SA
L/T
T
B/C
MaP
