# Supplementary material for: Delineation guidelines for the lymphatic target volumes in ‘prone crawl’ radiotherapy treatment position for breast cancer patients
Source: Sci Rep. 2021 Nov 18;11:22529. doi: 10.1038/s41598-021-01841-y (PMC8602302; doi:10.1038/s41598-021-01841-y)
Supplement: Supplementary file 3 — Supplementary Information 3. [file 41598_2021_1841_MOESM3_ESM.pdf]

# Prone crawl guideline: preparation guide

Linked to manuscript ‘Delineation guidelines for the lymphatic target volumes in ‘prone crawl’ radiotherapy treatment position for breast cancer patients ’

by

Michael E. J. Stouthandel, Françoise Kayser, Vincent Vakaet, Ralph Khoury, Pieter Deseyne, Chris Monten, Max Schoepen, Vincent Remouchamps, Alex De Caluwé, Guillaume Janoray, Wilfried De Neve, Stephane Mazy, Liv Veldeman, Tom Van Hoof.

# Contour colour legend

vein (V)

artery (A)

bones (light grey)

serratus anterior muscle (SA)

infraspinatus muscle (I)

subclavius muscle (SM)

sternocleidomastoid muscle (SCM)

anterior scalene muscle (AS)

biceps/coracobrachial muscle (B/C)

major pectoral muscle (MaP)

minor pectoral muscle (MiP)

lattissimus dorsi/teres major muscle (L/T)

deltoid muscle (D)

teres minor muscle (TM)

subscapular muscle (SS)

triceps muscle (T)

# Locating the vein in level IV (caudal)

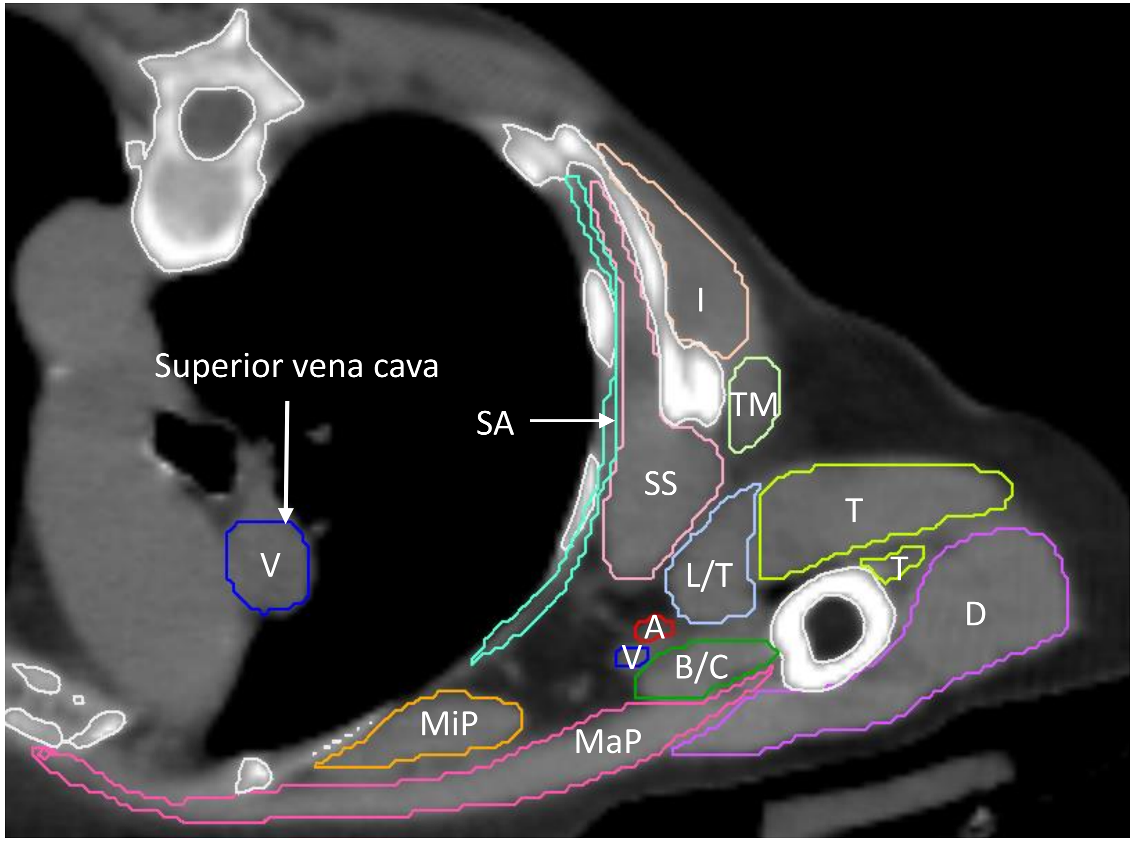


1. Start at the level where the superior vena cava joins the right atrium (caudal) (Slice above). This point is always clearly visible.

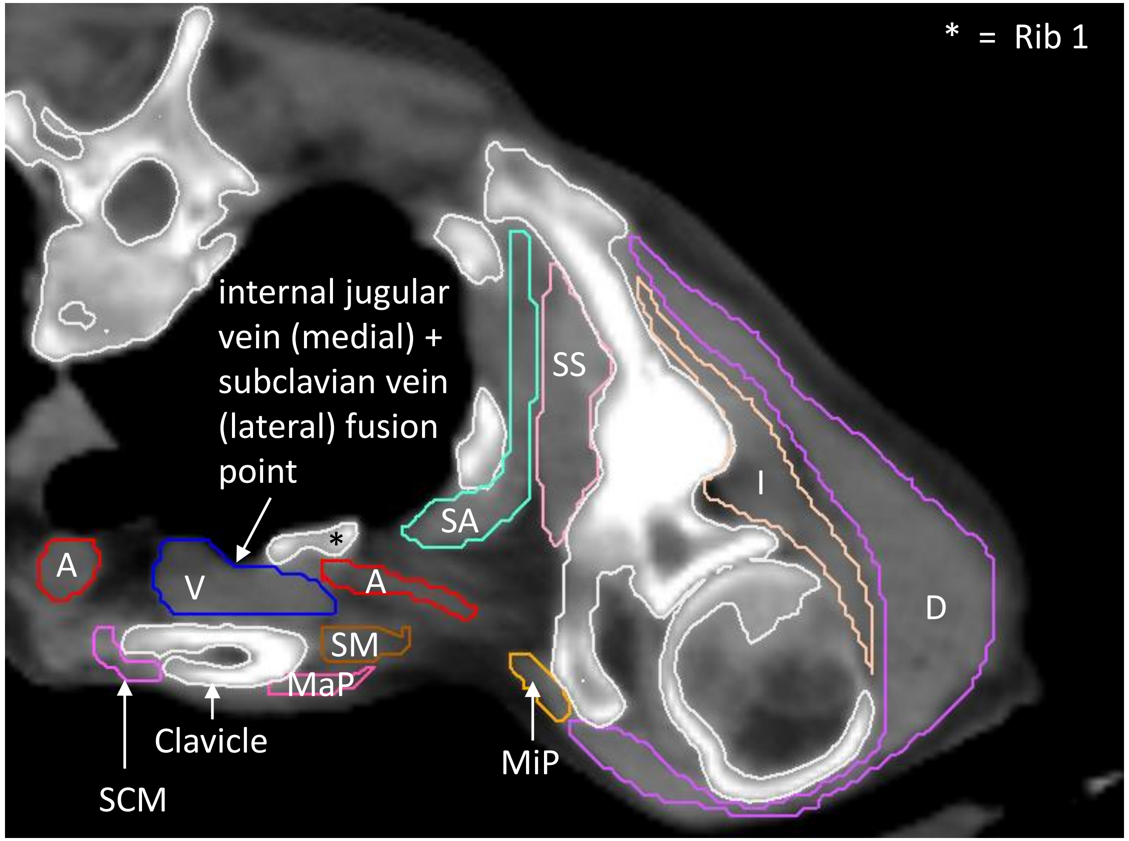


2. Scroll in cranial direction until the subclavian vein and internal jugular vein fuse for the first time (Slice above). This always happens in between the clavicle and the first rib.

# Locating the artery in level IV (caudal)

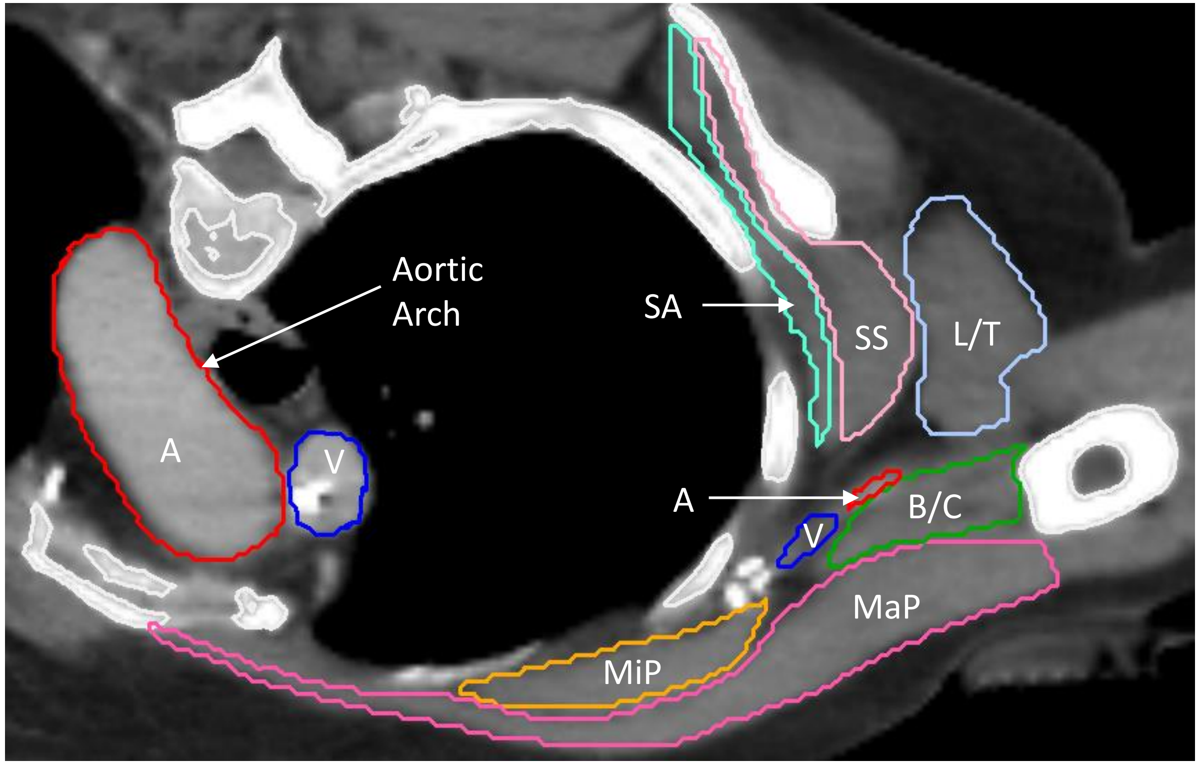


1. Start by locating the aortic arch caudally.

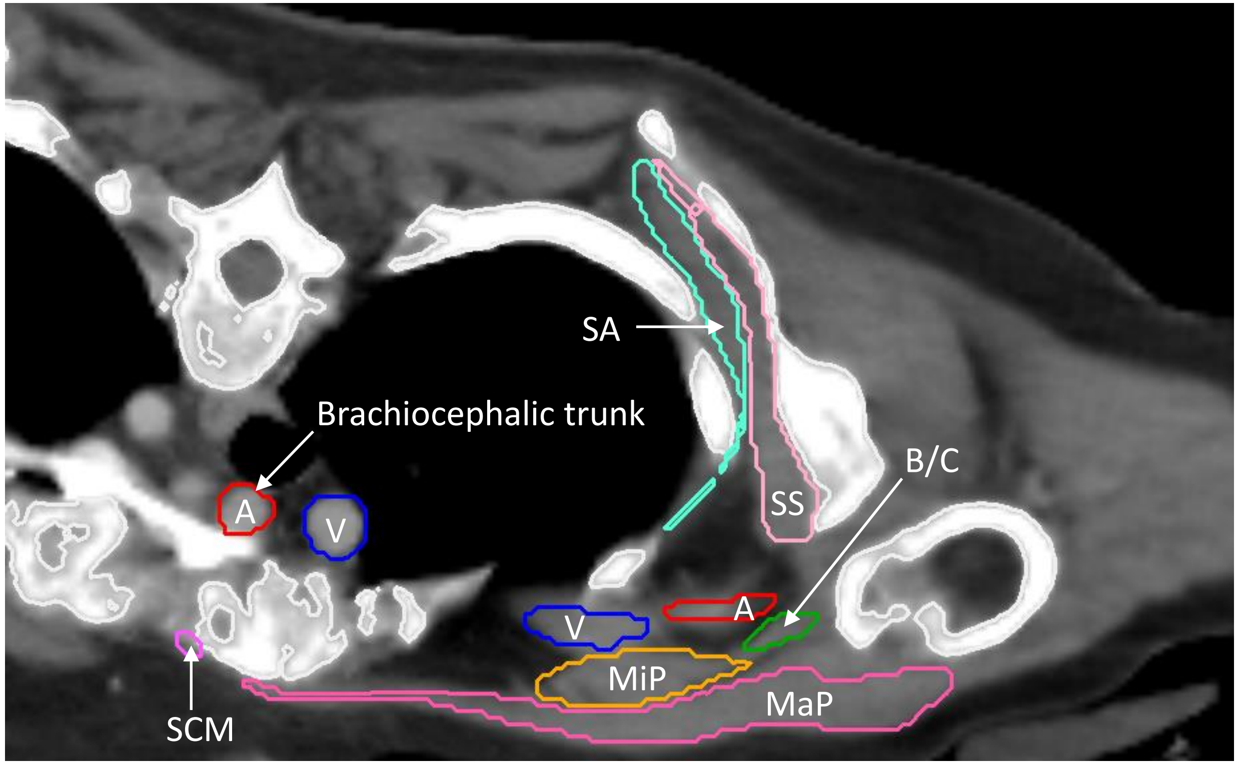


2. Then scroll in cranial direction until finding the braches of the aorta and follow the branch(es) moving towards level IV (brachiocephalic trunk on the right side and subclavian artery + common carotid artery on the left side.

# Locating the artery in level IV

- Trying to follow the course of the artery throughout level IV proved difficult and time consuming, therefore it is not recommended to spend too much time on it. The guidelines were postulated keeping this in mind.
- The following slides show how to distinguish the vein and artery at specific points in level IV, so the guidelines can easily be applied without having to focus on the entire course of the arteries in this level.

# Distinguishing the vein and artery in level IV (cranial)

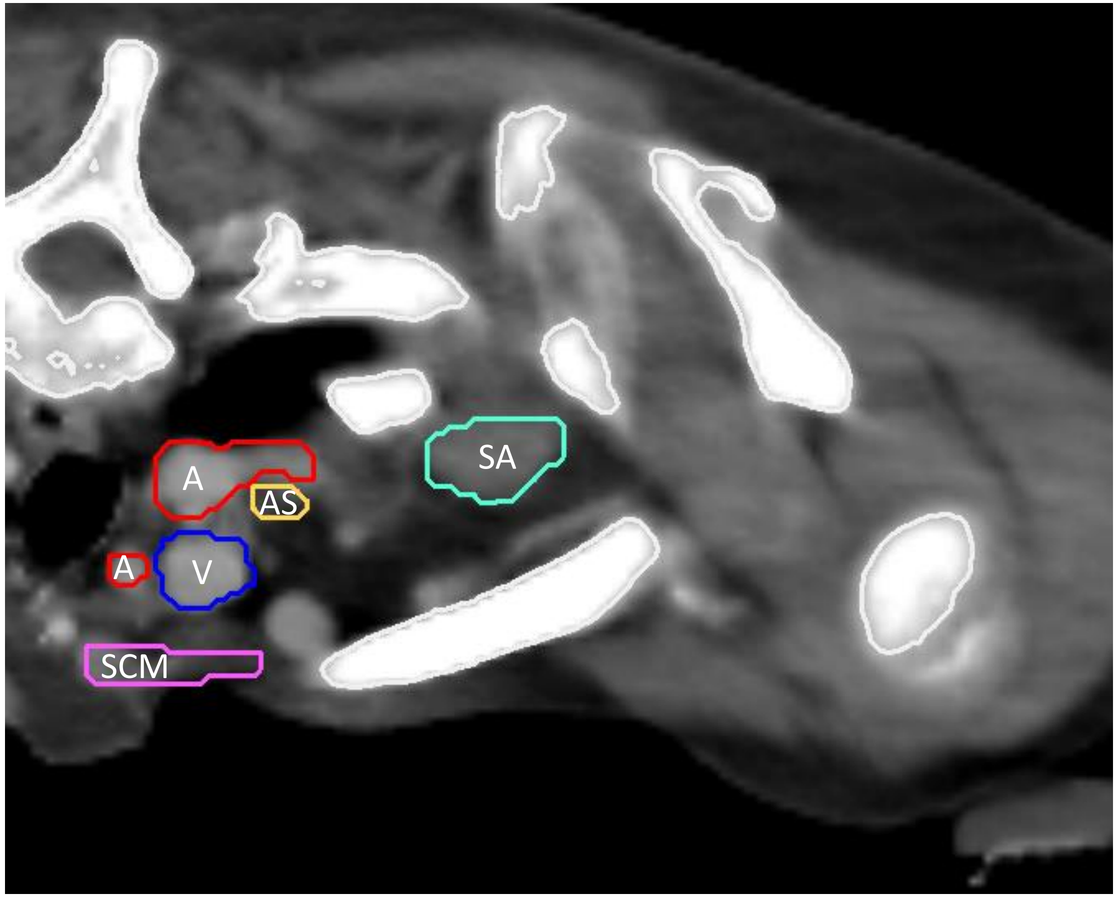


Cranially, the vein can be distinguished by its position relative to the anterior scalene muscle (AS). The subclavian/internal jugular vein always passes ventrally, while the subclavian artery always passes dorsally from the anterior scalene muscle.

# Distinguishing the vein and artery in level IV (most cranial)

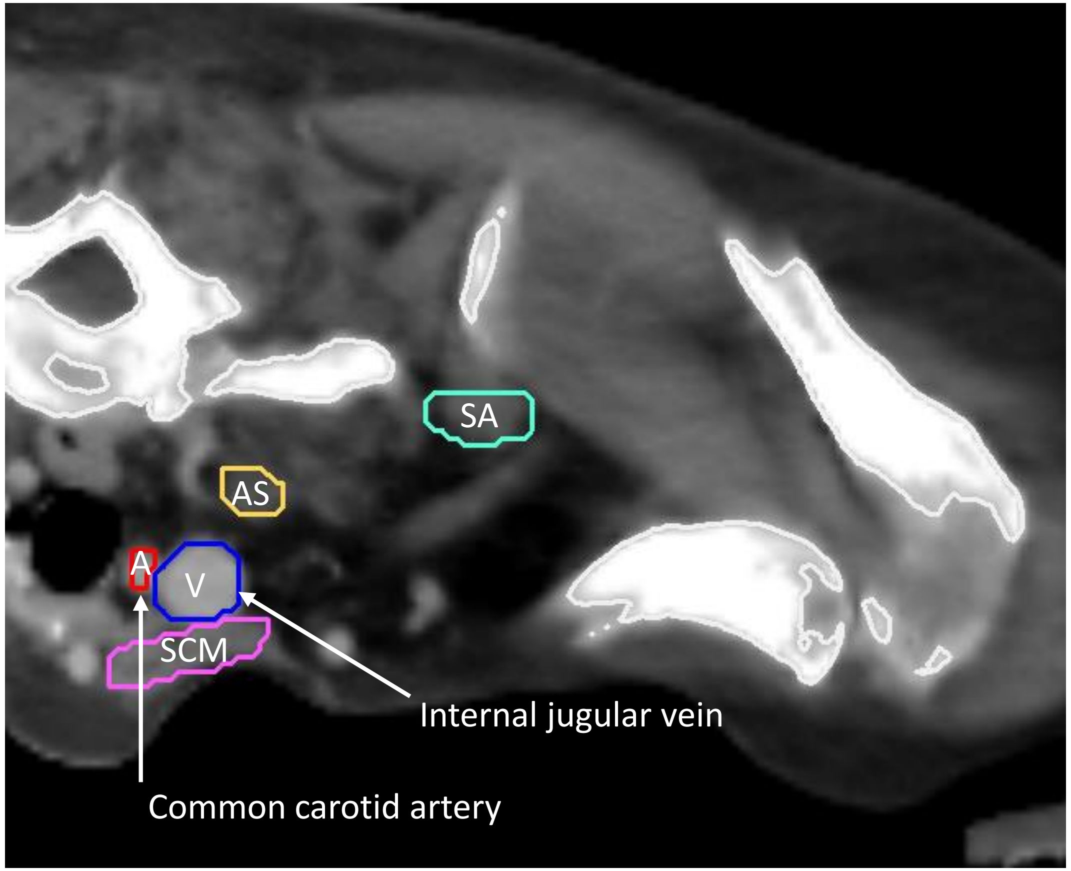


In the most cranial part of level IV, the internal jugular vein will always be located closer to the sternocleidomastoid muscle (SCM). The common carotid artery will always be located medially from the internal jugular vein.

# Locating the anterior scalene muscle (caudal)

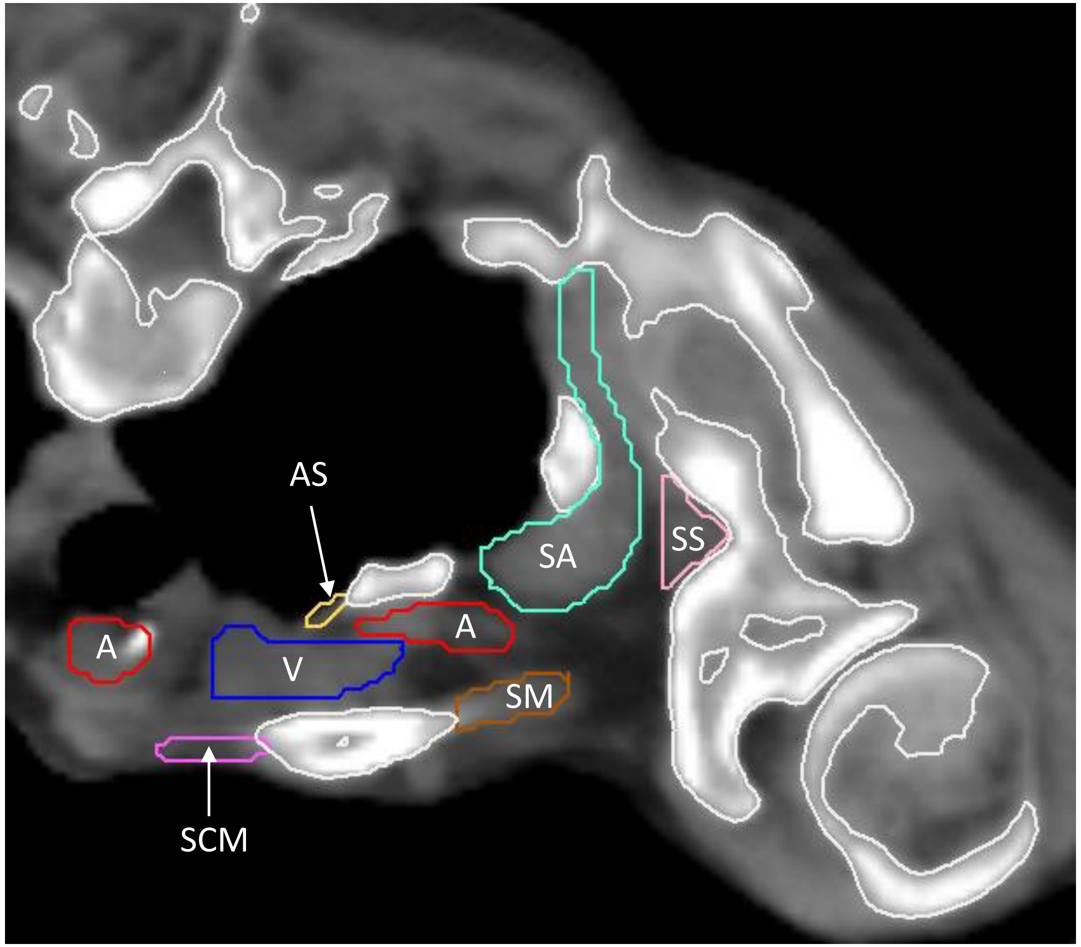


The first caudal slice where the anterior scalene muscle (AS) can be located is closely associated with the fusion point of the subclavian vein and the brachiocephalic vein. If possible, it can be followed to its insertion on the first rib.

For all datasets the first caudal slice containing the anterior scalene muscle was located at, or at most 2 slices more cranial than this fusion point.

# Locating the anterior scalene muscle (cranial)

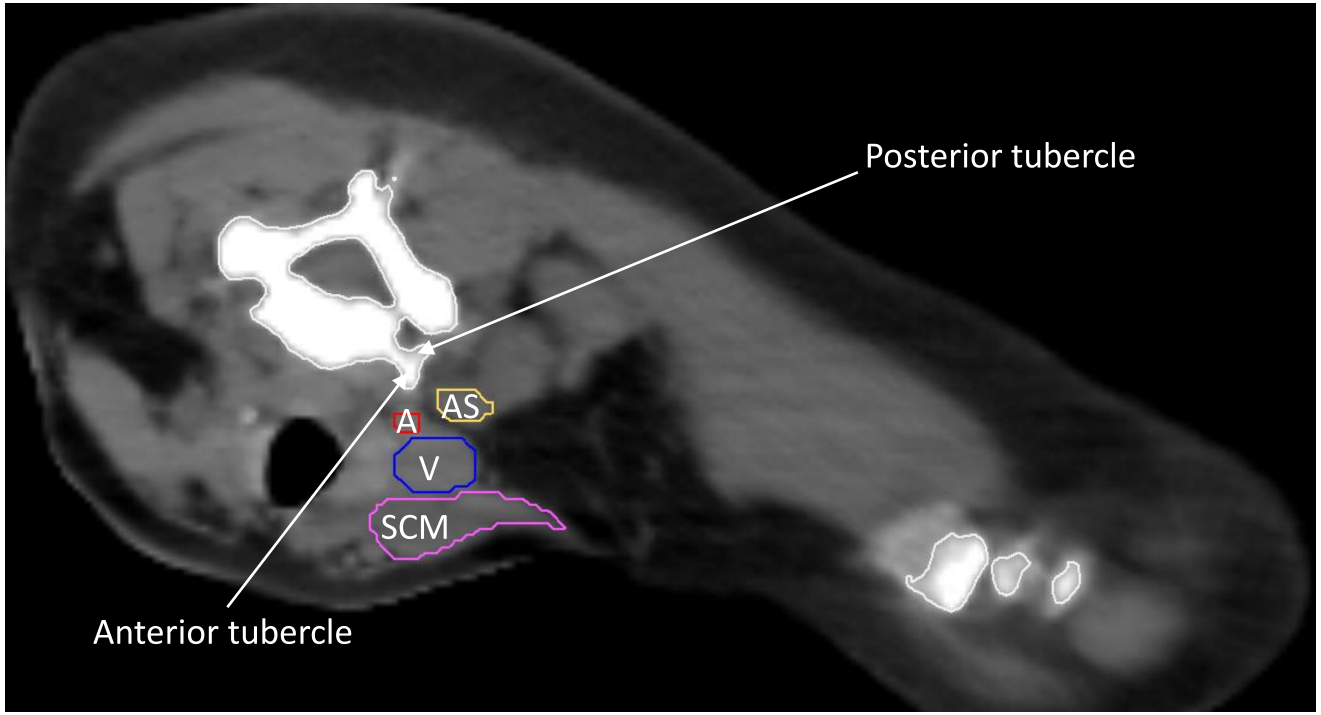


At the cranial level, the anterior scalene muscle (AS) will be located dorsally from the artery and vein.

The anterior scalene muscle originates from the transverse process (anterior tubercle) of the cervical vertebrae (C3-C6).

Keeping the origin (anterior tubercle of C3-C6) and the insertion (cranial side of the first rib, dorsal from the vein) in mind will help to locate the anterior scalene muscle when scrolling through the slices and focusing on these 2 points.

# Distinguishing the vein and artery in level III

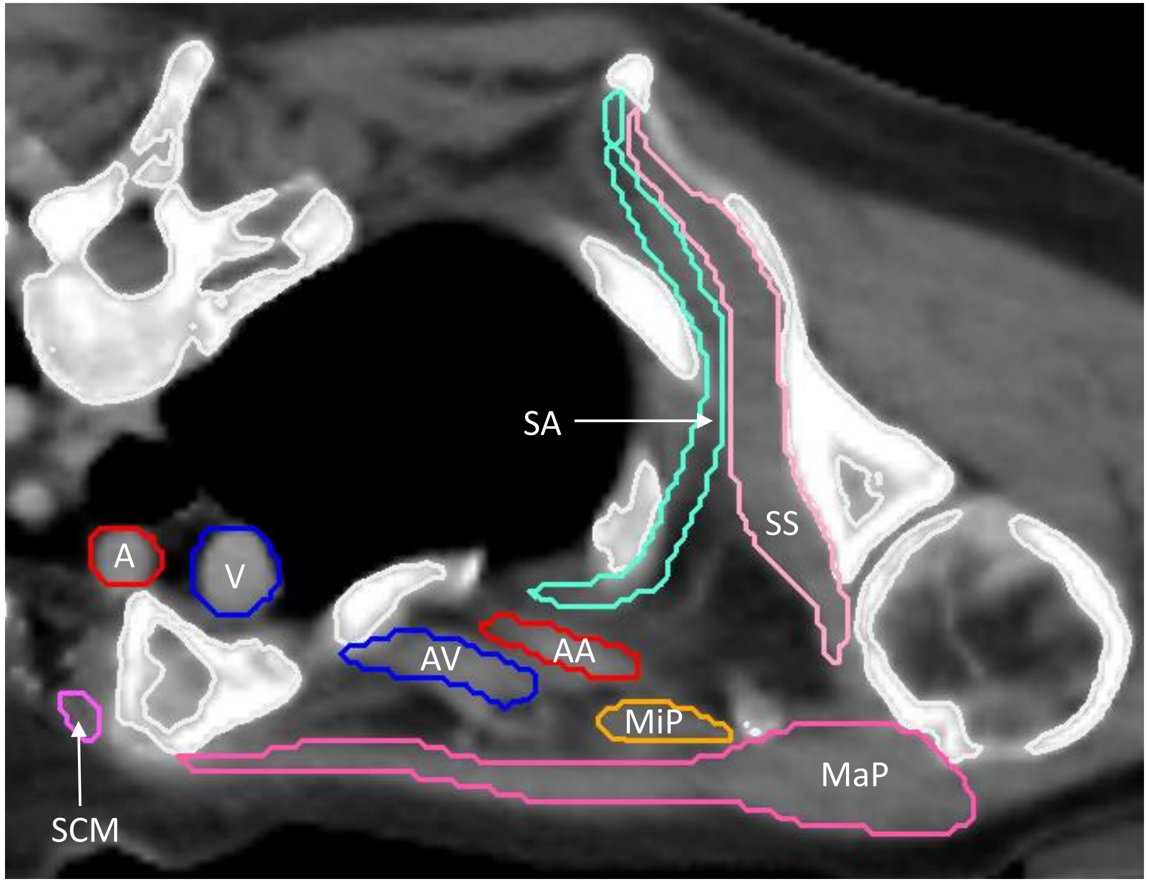


Because of the relation between the vein and the artery in level III, you do not need the location of the artery from level IV:

The axillary artery (AA) will always be located more laterally and more dorsally from the axillary vein (AV) in level III.

The vein can be traced from level IV, from the fusion point of the subclavian vein and the internal jugular vein that is always visible.

# Locating the subclavius muscle

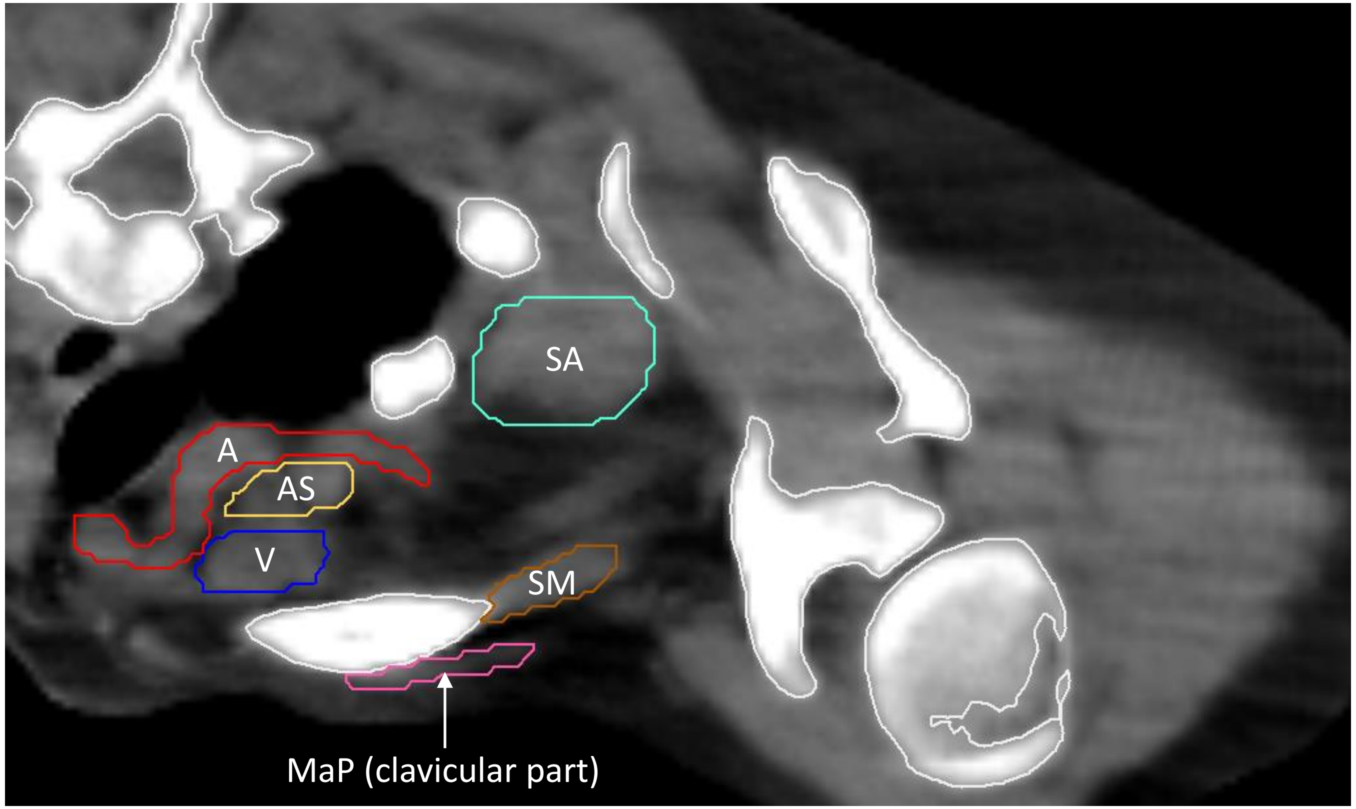


To easily locate the subclavius muscle, follow the major pectoral muscle (MaP) to its clavicular origin (most cranial slice containing MaP), the subclavian muscle is always present in this slice as well.

The subclavian muscle (SM) could be confused with the axillary vein when working too quickly, it is therefore important to locate it, before starting the delineations.

It is located on the lateral part of the clavicle and it will be oriented in dorsolateral direction, spanning 3-5 slices.

Following the origin and insertion of this muscle (like with the anterior scalene muscle) was hardly ever possible, because its origin at the first rib (and cartilage) was very difficult to locate. This doesn't matter however, because the lateral part is the part that is located within the CTV.

# Locating the minor pectoral muscle in level II and III

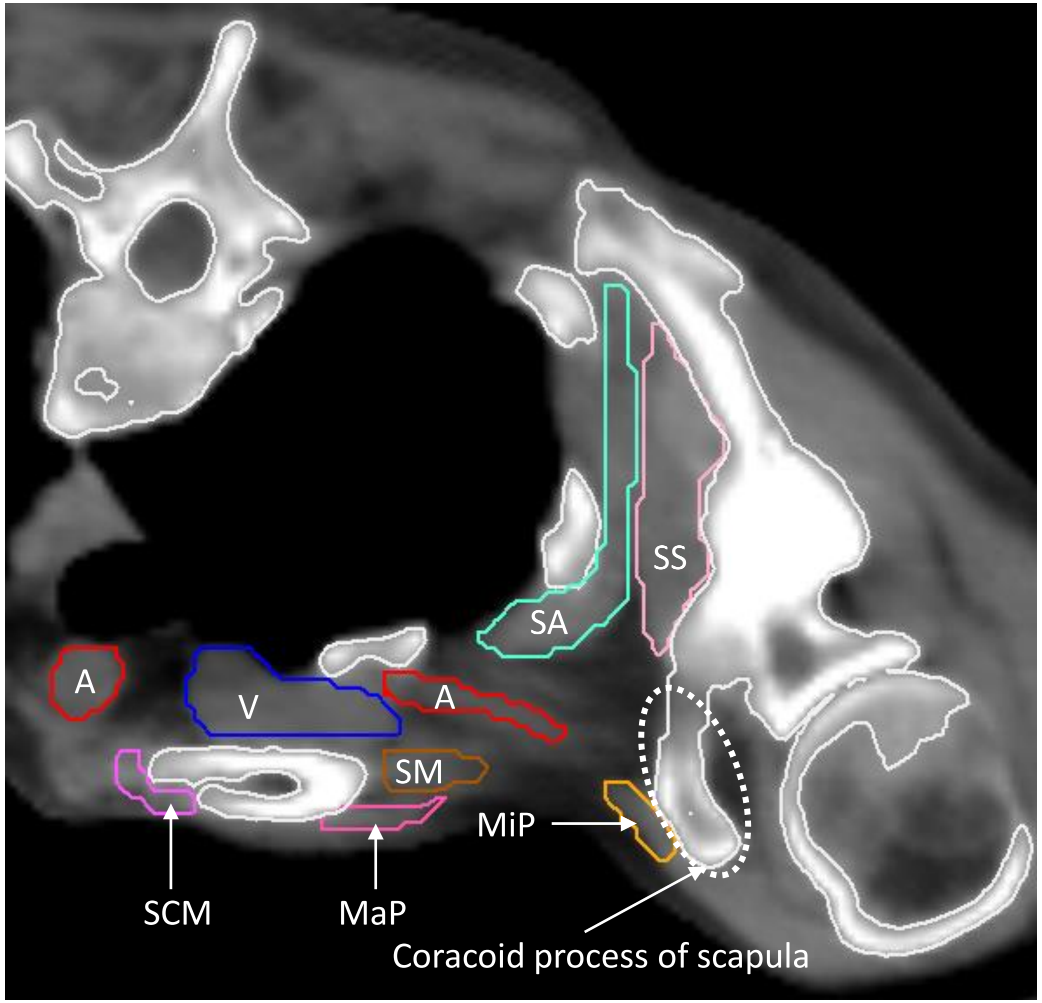


The minor pectoral muscle (MiP) could be mistaken for vascular structures in the most cranial slices when working too quickly, it is therefore important to first locate it before starting the delineation.

The easiest way to locate the MiP is to follow the scapula in cranial direction until encountering the coracoid process (white dotted line), this is where the most cranial part (insertion) of the MiP will be located.

Once the cranial part has been located, it can simply be followed as an uniterrupted structure until reaching its origin on the ribs.

# Distinguishing the artery and vein in level II

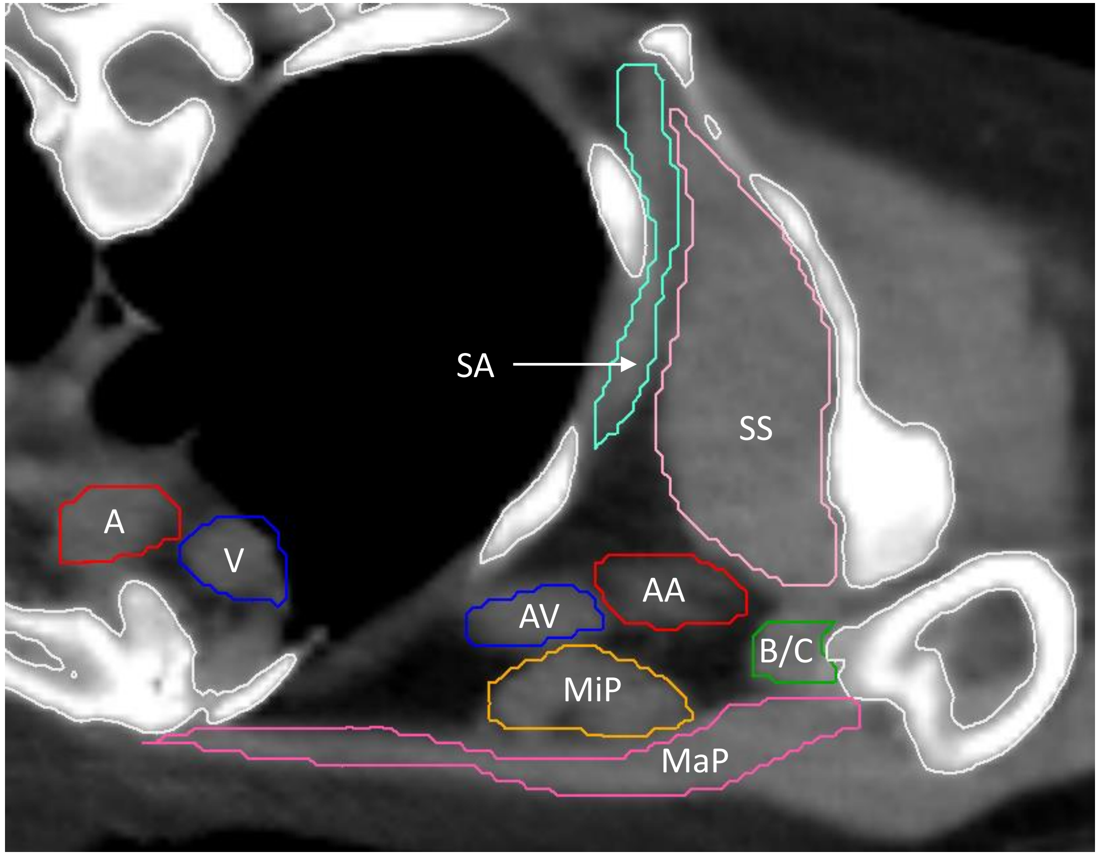


When starting to delineate the vein at level II instead of level III, the same logic can be applied, based on the anatomical relationship between the artery and vein:

The axillary artery (AA) will always be located more laterally and more dorsally than the axillary vein (AV).

This also means that the artery will always cross the medial and lateral border of the minor pectoral muscle (MiP) before the axillary vein does.

# Distinguishing the artery and vein in level I

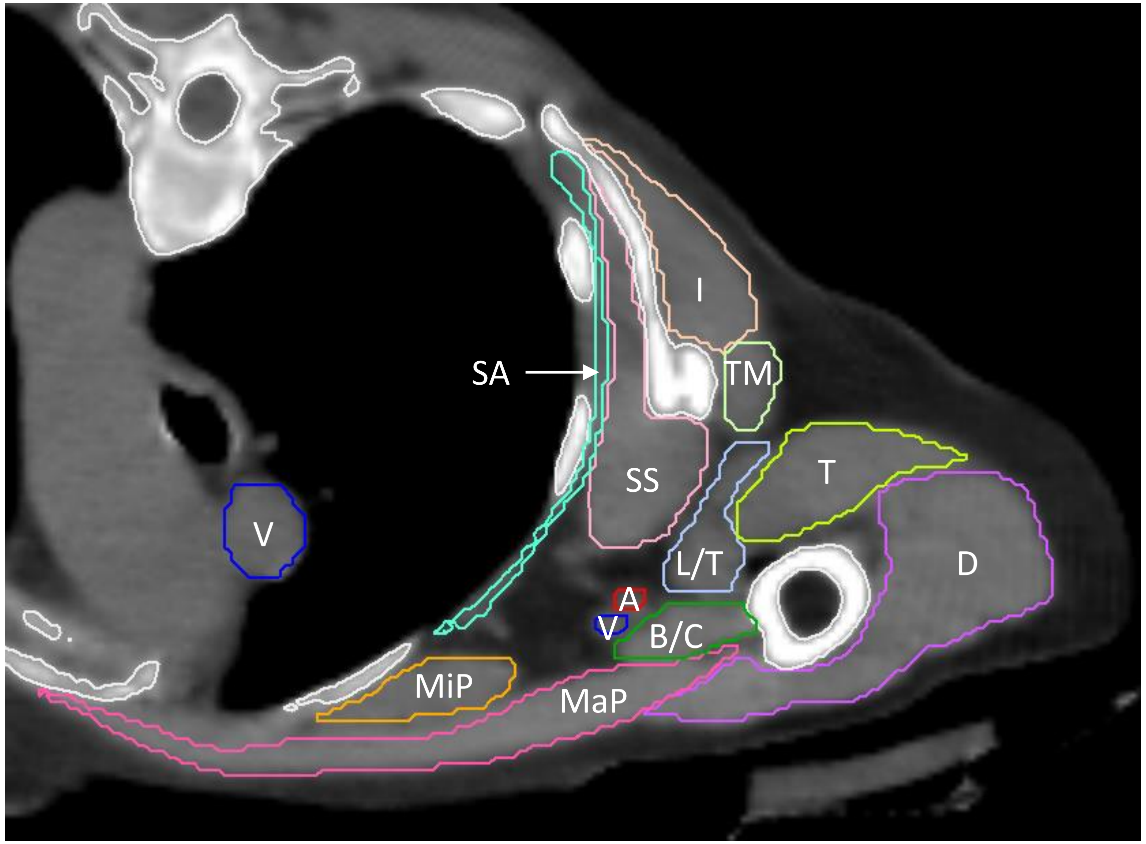


The relationship between the artery and vein remains the same in this level:

The artery will always be located more laterally and more dorsally than the vein.

Starting to locate the vein and artery in level II and following them into level I is easier, because it will not show as many branches in this areas, as it does in level I.

# Distinguishing the artery and vein in level I

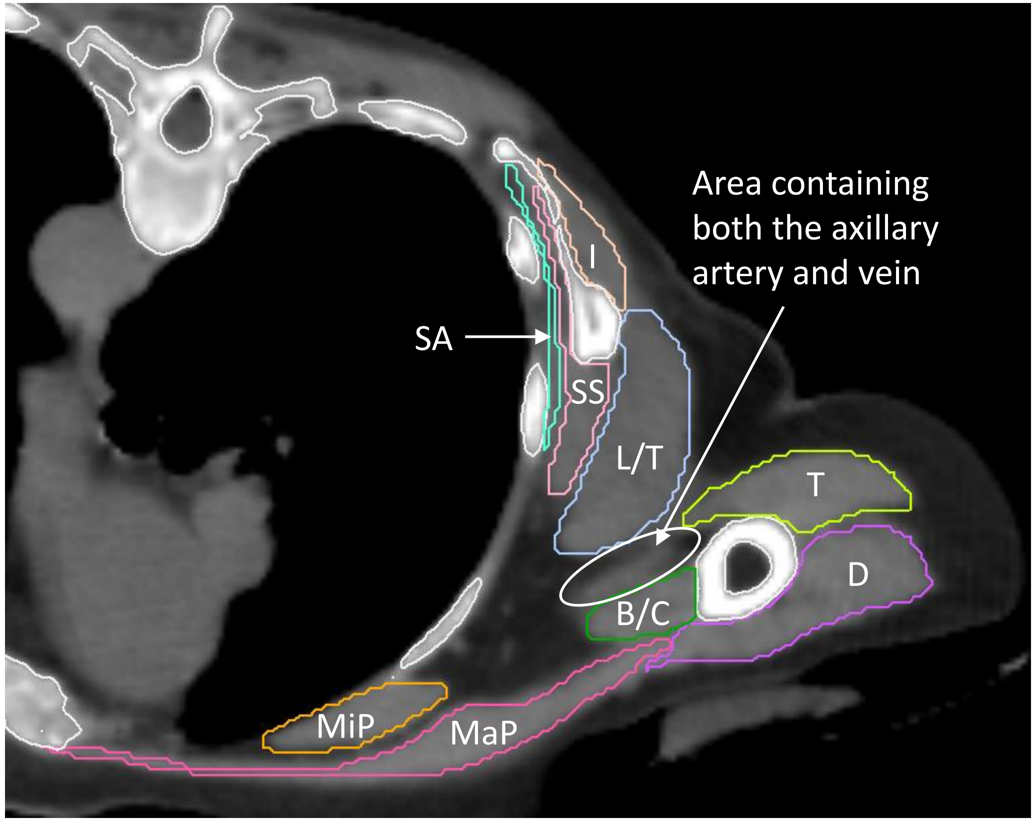


Because of their close proximity to each other and the many branches that emerge in this area, it is usually not possible to follow the artery and vein further than 5-7 slices in caudal direction from the cranial border of level I.

This was taken into account when postulating the guidelines for this level: Only the cranial border uses the vein, the rest of the borders in this level have bony or muscular reference points.

The artery and vein run in close proximity to the biceps/coracobrachial muscle bundle (B/C) in level I, so care should also be taken not to interpret them as part of the muscle volume.

# The biceps/coracobrachial muscle bundle (B/C)

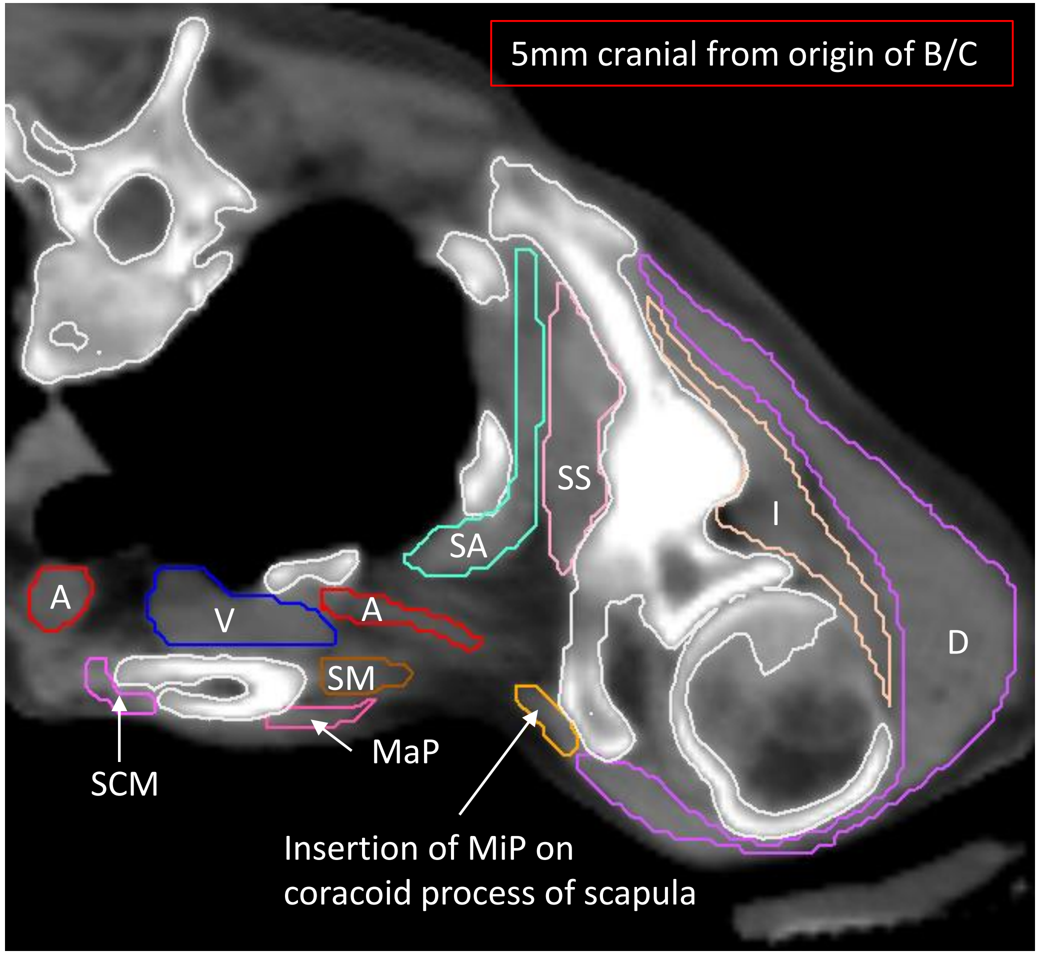


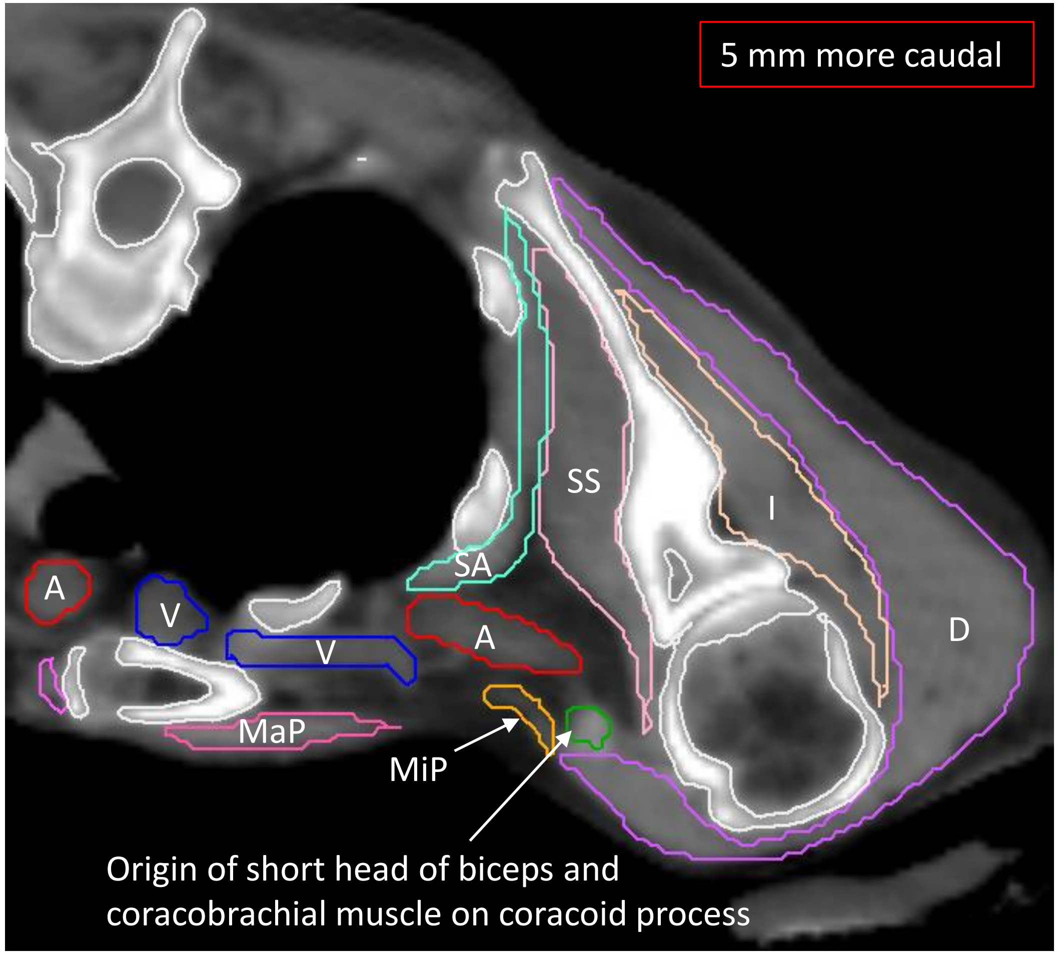

# The biceps/coracobrachial muscle bundle

- The origin of the short head of the biceps and the origin of the coracobrachial muscle are both located on the coracoid process of the scapula, in close proximity to each other.
- They both follow the same trajectory towards the arm and it is difficult/impossible to distinguish the individual muscle bundles on patient CT scans, because of their close proximity.
- Therefore, they are described as a single muscle bundle, to not complicate the guidelines, while still remaining anatomically correct.

# The lattissimus dorsi/teres major bundle (L/T)

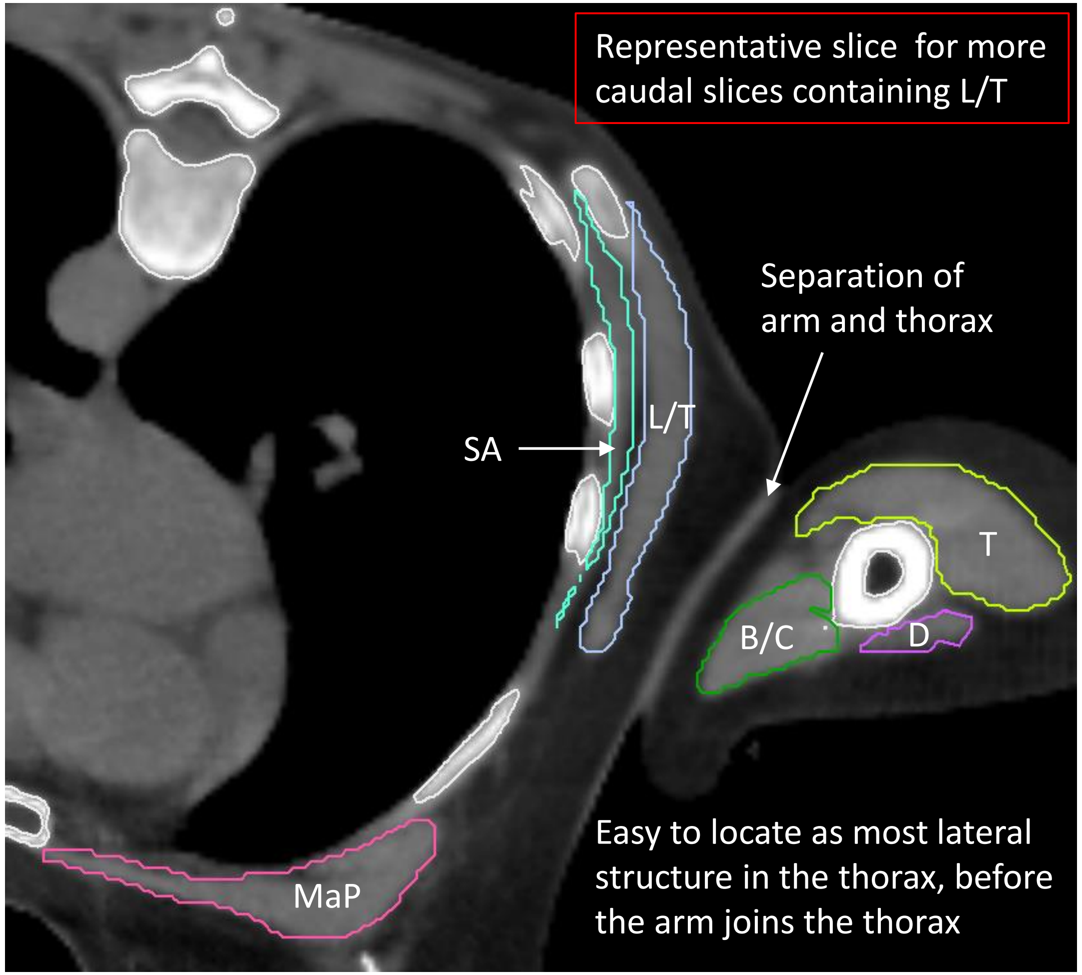


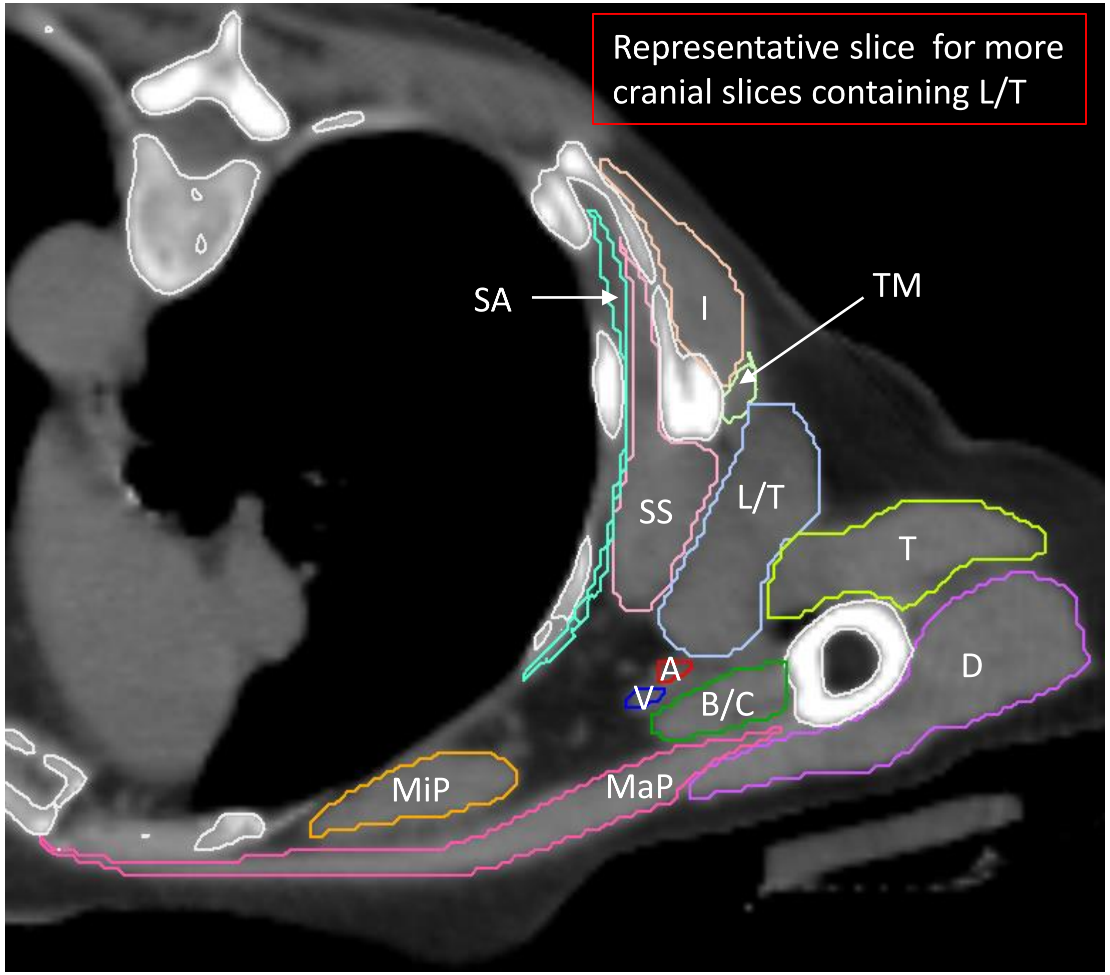

# The lattissimus dorsi/teres major muscle bundle

- Towards the insertion of the lattissimus dorsi and the teres major on the humerus, both muscles run in close proximity to each other.
- The teres major is located in a groove that is formed by the latissimus dorsi tendon, but the different elements of these muscles can not be distinguished on patient CT scans.
- Therefore, they are described as a single muscle bundle, to not complicate the guidelines, while still remaining anatomically correct.

# Identifying the internal mammary vessels

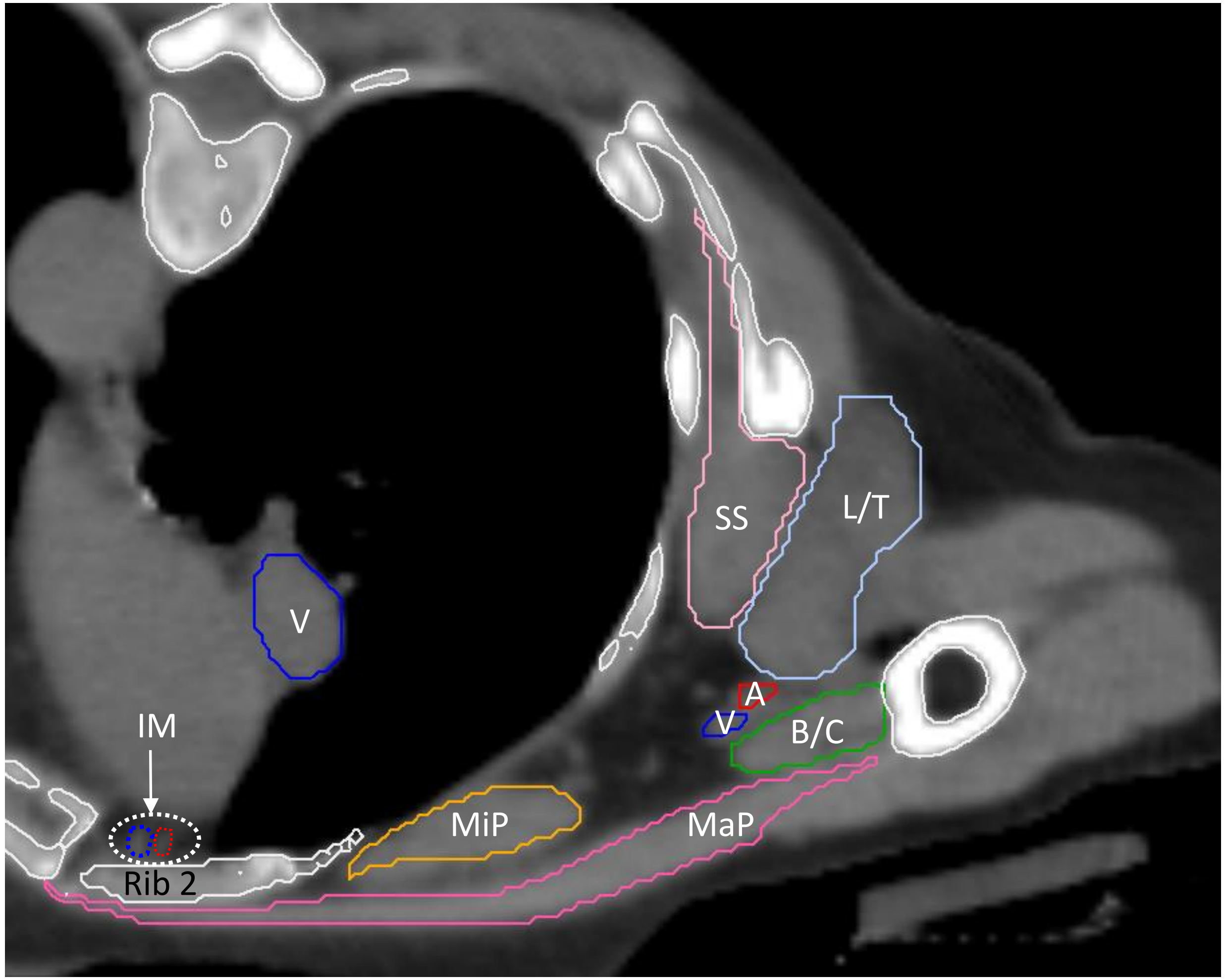


To locate the internal mammary vessels (IM), in case the place where they fuse with the brachiocephalic vein is not clear: locate the point where the second rib attaches to the sternum.

2 vessels can consistently be found here (white dotted outline), located dorsally from the rib and laterally from the sternum: these are the internal mammary vessels.

To locate the internal mammary vein (blue dotted outline), remember that the internal mammary vein is always located medially from the internal mammary artery (red dotted outline).

Once the internal mammary vein is located at this point, it can be traced back to the fusing point with the brachiocephalic vein

# Identifying the internal mammary vein

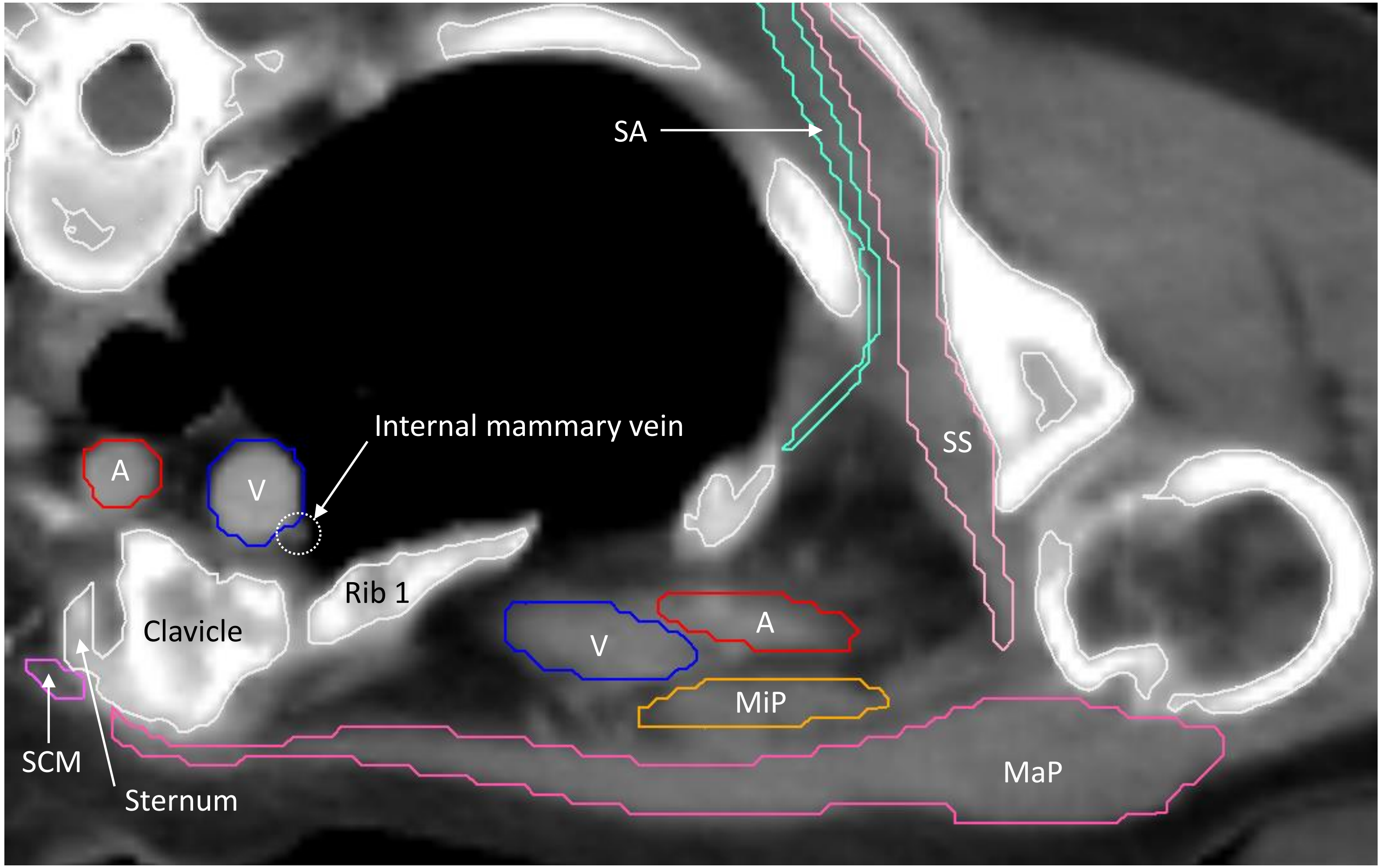


The internal mammary vein always joins the brachiocephalic vein at the ventrolateral part (white dotted lines).

This confluence always appears in the area between where the clavicle attaches to the sternum and the area where the first rib attaches to the sternum.

Combining this knowledge with the location of the internal mammary vein at the level of the second sternocostal joint (previous slide) allows you to trace the length of the internal mammary vein in its cranial aspect.
