# Supplementary material for: Delineation guidelines for the lymphatic target volumes in ‘prone crawl’ radiotherapy treatment position for breast cancer patients
Source: Sci Rep. 2021 Nov 18;11:22529. doi: 10.1038/s41598-021-01841-y (PMC8602302; doi:10.1038/s41598-021-01841-y)
Supplement: Supplementary file 4 — Supplementary Information 4. [file 41598_2021_1841_MOESM4_ESM.pdf]

Supplementary material explanation

Linked to manuscript 'Delineation guidelines for the lymphatic target volumes in 'prone crawl' radiotherapy treatment position for breast cancer patients'

by

Michael E. J. Stouthandel*, Françoise Kayser, Vincent Vakaet, Ralph Khoury, Pieter Deseyne, Chris Monten, Max Schoepen, Vincent Remouchamps, Alex De Caluwé, Guillaume Janoray, Wilfried De Neve, Stephane Mazy, Liv Veldeman, Tom Van Hoof.

PDF titled '*Preparation guide PCP*'.

This file is recommended for people that are new to the prone crawl position. It contains screenshots and explanations that will help you to quickly orient yourself when delineating your first prone crawl dataset. It will teach you how to quickly locate and distinguish the structures that will be required for a prone crawl delineation, using the prone crawl guidelines presented in this manuscript.

PDF titled '*Completely delineated dataset PCP*'

This file shows a 'normal patient' delineated using the prone crawl guidelines, showing a screenshot from every slice that contains a target volume and 3 additional slices without target volume in cranial and caudal direction. Slices are supplied in cranio-caudal order and all levels are depicted at the same time to give a good overview of the lymphatic target volumes that should be obtained and where the different levels start and stop. No additional explanation is given in this file, since this file is meant as a quick overview.

PDF titled '*Application guide PCP*'.

This file will prove useful during the first few delineations in prone crawl position as it explains in detail how to apply the written guidelines. This file contains screenshots that focus on one level at a time and it details how to apply each border separately. Along with explanations for each border and a 3D representation of the CTVs for all the patient CT datasets that were used, this file also shows some rare findings that were only encountered in 1 or 2 out of 9 patient CT datasets. These rare findings had an impact on guideline applicability, so they are described and an alternative guideline is provided to ensure a proper delineation in case these rare findings occur.

Apart from the description of the different levels in the results section of the manuscript and the overview of the guidelines in table 1 of the manuscript, the following (more detailed) description can be used as an alternative. It contains references to the PDF titled 'Application guide PCP', so a certain border can be reviewed in the accompanying screenshot if the textual description alone is not clear.

Level IV

Cranial: Locate the most cranial slice that still contains pleura, add 5 mm in cranial direction. (slide 3)

Caudal: One slice (5mm) below the first caudal slice where the axillary/subclavian vein fuses with the internal jugular vein/brachiocephalic vein. In this slice, the axillary/subclavian vein and the brachiocephalic vein will both be visible, but not yet connected. (slide 4)

- Referring to the first cranial or the first caudal slice that contains a structure is necessary, because it depends on which way you are scrolling through the dataset. (This principle is explained in slide 4 and slide 5 shows the caudal border of level IV.) (slide 5)

Medial: The medial edge of the internal jugular vein and more caudally the medial edge of the subclavian vein and brachiocephalic vein, without margin, excluding the thyroid gland (and the artery if visible). (slide 6)

Lateral: The lateral edge of the anterior scalene muscle. Once the anterior scalene muscle is no longer visible, switch to the lateral edge of the clavicle. (slide 7)

Ventral: The dorsal edge of the sternocleidomastoid muscle, the dorsal edge of the clavicle, or a line connecting the 2 if they are not adjacent. (slide 8)

Dorsal: 5 mm dorsal from the most dorsal point of the vein within this level. If the 5 mm margin extends beyond the ventral edge of the anterior scalene muscle, the artery, the first rib, or the pleura, follow along the contours of these structures until the 5 mm can be respected again, or until reaching the medial/lateral border of the CTV. (slide 9)

Level III

Cranial: The first cranial slice where the subclavian *artery* has first passed the lateral edge of both the clavicle and the first rib. The artery is always the most dorsally located vascular structure in this level. (slide 10)

Caudal: Locate the first cranial slice where the subclavian *vein* crosses the medial edge of the minor pectoral muscle and add 5 mm in caudal direction. (slide 11)

- If the caudal border of level III is located cranially from the caudal border of level IV, the axillary vein limit should be disregarded and the caudal border is extended to the most caudal slice containing level IV. (slide 12)

Medial: The lateral border of level IV, or the lateral border of the clavicle if level IV is no longer present. (slide 13)

Lateral: The lateral edge of the subclavian artery, until it crosses the medial edge of the minor pectoral muscle. When this happens, the lateral border switches to the medial edge of the minor pectoral muscle. (slide 14 and 15)

Ventral: The dorsal edge of the clavicle, the subclavian muscle, the major pectoral muscle, or an imaginary line connecting the major pectoral muscle to the minor pectoral muscle. (slide 16 and 17, left)

- If the major pectoral muscle is not visible in the most cranial slices, a line connecting the ventral edge of the clavicle to the ventral edge of the minor pectoral muscle can be used as ventral border. (slide 17, right)
- In case the subclavius muscle is located within level III and fatty tissue is present between the subclavius muscle and the: include this tissue (in accordance with PROCAB). (slide 18)

Dorsal: Take a 5 mm dorsal margin from the most dorsal part of the vein in this level. If the 5 mm margin extends beyond the ventral edge of the serratus anterior, the ribs or the intercostal muscles, follow along their surface until the 5 mm margin can be respected again, or until reaching the medial or lateral border. (slide 19 and 20)

- When the vein is not yet inside level III (most cranial slices) it is not possible to take the 5 mm dorsal margin from the vein inside level III. In this case, respect the dorsal border of level IV (5 mm dorsal from vein in level IV). (slide 21)

Level II

Cranial: The first cranial slice where the axillary artery crosses the medial edge of the minor pectoral muscle. The axillary artery always crosses the medial edge of the minor pectoral muscle more cranially than the axillary vein. (slide 22, left)

- It is possible that the cranial border of level III and level II coincide. This can happen when the artery spans a long latero-lateral distance in that particular slice. (slide 22, right)

Caudal: The last slice that contains fatty tissue between the pec minor and the ribs/intercostal muscles. (slide 23)

Medial: Medial edge of the minor pectoral muscle, or the point where the minor pectoral muscle attaches to the ribs in the more caudal slices. (slide 24)

Lateral: Lateral edge of minor pectoral muscle, or the medial border of level I if already present. (slide 25)

Ventral: Dorsal edge of the minor pectoral muscle. (slide 26)

Dorsal (cranial): If the vein is located inside level II (cranial), respect a 5 mm margin taken from the most dorsal part of the vein. Exclude the serratus anterior, intercostal muscles and ribs. (slide 27, left)

- When the vein is not yet inside level II (most cranial slices) it is not possible to take the 5 mm dorsal margin from the vein inside level II. In this case, respect the dorsal border of level III (5 mm dorsal from vein in level III).

Dorsal (caudal): From the moment that level I starts (see cranial border level I) and both level I and level II appear in the same slice, the dorsal border of level II follows the dorsal border of level I. This ensures a smooth connection of the dorsal border of level I and level II, making sure no tissue is left out between the medial border of level I, the lateral border of level II and the ribs. (slide 27, right)

Level I

Cranial: Locate the first cranial slice where the axillary vein first crosses the lateral edge of the minor pectoral muscle, and add 5 mm in cranial direction. (slide 28)

Caudal: The point where the 4th rib attaches to the sternum. Stop at the first caudal slice where the 4th rib is no longer visible next to the sternum. (slide 29)

Medial: The lateral edge of the minor pectoral muscle, or the lateral border of level II if still present. The dorsomedial border follows the chest wall to connect the dorsal and lateral border. (slide 30)

Lateral (cranial): In the cranial part of level I, the lateral border is the medial edge of the biceps/coracobrachial muscle bundle. The lateral border reaches until (the level of) the ventral edge of the latissimus dorsi/teres major muscle bundle, or the subscapular muscle bundle dorsally (whichever is located more ventrally). (slide 31, left)

Lateral (caudal): Once the major pectoral muscle is no longer visible as a single object (no longer reaching towards the insertion on the humerus), the lateral border changes to the lateral edge of the major pectoral muscle. Connect this point directly to the most ventral point of the latissimus dorsi/teres major muscle bundle (L/T). In the more caudal slices, follow the curvature of the ribs to make the connection between the major pectoral muscle and the L/T. (slide 31, right)

Ventral: The dorsal edge of the major pectoral muscle. (slide 32)

Dorsal: The ventral edge of the subscapular muscle (slide 33) or the L/T (slide 34). The dorsal border is formed by connecting the dorsal endpoint of the lateral border to the ribs/intercostal muscle/serratus anterior muscle using a straight line in latero-lateral direction. (slide 33 and 34)

- If vascular structures that pass in between the L/T and serratus anterior muscle can clearly be traced to the axillary artery, or the axillary vein and they are located within the target volume, they can be excluded to spare them by adapting the dorsolateral border of level I. When in doubt, the vascular structures could represent lymphatic tissues instead of (branches of) the thoracodorsal vessels and they should be included in the CTV. (slide 35 and 36)

Internal mammary nodes (IM)

Cranial: The cranial border starts 5 mm caudal from the most caudal slice containing level IV. (slide 37)

- The point where the internal mammary vein joins the brachiocephalic vein can be hard to locate. If this point is not identified or not yet present, the CTV in the most cranial slices becomes the space between dorsal edge of the 1st rib, the dorsal edge of the clavicle and the ventral edge of the brachiocephalic vein. (slide 38) (rare finding)

Caudal: Locate the most caudal slice where the 4th rib is still connected to the sternum by the sternocostal joint. Take 5 mm more caudal from this point (the 4th rib is no longer attached to the sternum in this slice). This is the same caudal border as the caudal border of level I. (slide 39)

Medial: For the medial border, a 5 mm margin medial from the internal mammary vein is respected. The clavicle, or sternum should be excluded from the CTV, even if they are located within the 5 mm limit. (slide 40)

Lateral: For the lateral border, a 5 mm margin medial from the internal mammary vein is respected. The first rib and the pleura should be excluded from the CTV, even when they are located within the 5 mm margin. (slide 41)

Ventral: The ventral border of this CTV is formed by the dorsal edge of the clavicle, the ribs, the sternum, or the dorsal edge of the intercostal muscles. (slide 42)

Dorsal: For the dorsal border, a 5 mm margin dorsal from the internal mammary vein is respected. In practice this almost always means that the dorsal borders are the ventral edge of the brachiocephalic vein, the pleura or the pericardium. (slide 43)

Interpectoral nodes (IP)

Cranial: The first cranial slice where the axillary artery crosses the medial edge of the minor pectoral muscle. This is the same cranial border as the one for level II. (slide 44)

- It is possible that the major pectoral muscle is not yet located ventrally from the minor pectoral muscle in the most cranial slices (even though level II is already present in these slices). If this occurs, the cranial border shifts to the first cranial slice where the major pectoral muscle first completely covers the minor pectoral muscle ventrally. (slide 45)

Caudal: The caudal border is the last caudal slice where there is still fatty tissue located between ventral edge of the ribs and the dorsal edge of the minor pectoral muscle. This is the same caudal border as the one used for level II. (slide 46)

Medial: The medial border is the medial edge of the minor pectoral muscle. This is the same medial border as the one used for level II. (slide 47)

- In some slices it can occur that parts of the major and minor pectoral muscle touch, without showing fatty tissue in between them. In this case, the medial edge is the part where no more fatty tissue is located between the major and minor pectoral muscles. (slide 47, right)

Lateral: The lateral border is the lateral edge of the minor pectoral muscle. This is the same lateral border as the lateral border used for level II. (slide 48)

- In some slices it can occur that parts of the major and minor pectoral muscle touch, without showing fatty tissue in between them. In this case, the lateral edge is the part where no more fatty tissue is located between the major and minor pectoral muscles. (slide 48, right)

Ventral: The ventral border is the dorsal edge of the major pectoral muscle. (slide 49)

Dorsal: The dorsal border is the ventral edge of the minor pectoral muscle. (slide 50)
